# TMEM43 promotes pancreatic cancer progression by stabilizing PRPF3 and regulating RAP2B/ERK axis

**DOI:** 10.1186/s11658-022-00321-z

**Published:** 2022-03-08

**Authors:** Junqiang Li, Yang Song, Chao Zhang, Ronglin Wang, Lei Hua, Yongdong Guo, Dongxue Gan, Liaoliao Zhu, Shanshan Li, Peixiang Ma, Cheng Yang, Hong Li, Jing Yang, Jingjie Shi, Xiaonan Liu, Haichuan Su

**Affiliations:** 1 Department of Oncology, Tangdu Hospital, Air Force Medical University, Xi’an, 710038 Shaanxi China; 2 Ambulatory Surgery Center, Xijing Hospital, Air Force Medical University, Xi’an, 710032 Shaanxi China

**Keywords:** Pancreatic cancer, Progression, TMEM43, PRPF3, RAP2B

## Abstract

**Background:**

Transmembrane protein 43 (TMEM43), a member of the transmembrane protein subfamily, plays a critical role in the initiation and development of cancers. However, little is known concerning the biological function and molecular mechanisms of TMEM43 in pancreatic cancer.

**Methods:**

In this study, TMEM43 expression levels were analyzed in pancreatic cancer samples compared with control samples. The relationship of TMEM43 expression and disease-free survival (DFS) and overall survival (OS) were assessed in pancreatic cancer patients. In vitro and in vivo assays were performed to explore the function and role of TMEM43 in pancreatic cancer. Coimmunoprecipitation (co-IP) followed by protein mass spectrometry was applied to analyze the molecular mechanisms of TMEM43 in pancreatic cancer.

**Results:**

We demonstrated that TMEM43 expression level is elevated in pancreatic cancer samples compared with control group, and is correlated with poor DFS and OS in pancreatic cancer patients. Knockdown of TMEM43 inhibited pancreatic cancer progression in vitro, decreased the percentage of S phase, and inhibited the tumorigenicity of pancreatic cancer in vivo. Moreover, we demonstrated that TMEM43 promoted pancreatic cancer progression by stabilizing PRPF3 and regulating the RAP2B/ERK axis.

**Conclusions:**

The present study suggests that TMEM43 contributes to pancreatic cancer progression through the PRPF3/RAP2B/ERK axis, and might be a novel therapeutic target for pancreatic cancer.

**Supplementary Information:**

The online version contains supplementary material available at 10.1186/s11658-022-00321-z.

## Background

Pancreatic cancer, with a 5-year patient survival rate of no more than 5%, is one of the most lethal cancers [1, 2]. Most patients are poorly diagnosed owing to nonspecific symptoms, such as abdominal pain and weight loss [3]. Only 15–20% of the patients with pancreatic cancer have the chance to receive surgery [4]. Radiation, chemotherapy, or their combination are not always effective for patients with pancreatic cancer because of a lack of sensitivity and tumor drug resistance [5]. Therefore, there is an urgent need to discover more new oncogenes and explore the molecular mechanisms responsible for the progression of pancreatic cancer.

Transmembrane protein 43 (TMEM43), a member of the TMEM subfamily, is encoded by a highly conserved gene and is widely expressed in most species, ranging from bacteria to humans [6]. The TMEM43 S358L mutation on chromosome 3p25 is an important cause of arrhythmogenic right ventricular cardiomyopathy (ARVC) as it enhances the NF-κB-TGFβ signal cascade, which can lead to aggressive disease at the early stage, and might later develop into heart disease [7–9]. However, the function and role of TMEM43 in cancer are vague. The upregulation of TMEM43 in brain tumor cells accelerated brain tumor progression through the interaction of TMEM43 with CARD-containing MAGUK protein 3 (CARMA3) in the EGFR-induced NF-κB pathway [10]. To the best of our knowledge, the function and molecular mechanisms of TMEM43 in pancreatic cancer are unclear.

In this study, we demonstrate that TMEM43 expression is closely associated with its clinicopathological characteristics and poor survival outcomes. We also demonstrate that the suppression of TMEM43 could restrain pancreatic cancer growth, migration, and invasion in vitro and in vivo. Our study shows that the TMEM43/PRPF3/RAP2B/ERK axis plays a vital role in regulating cancer progression, and has a potential clinical application value for pancreatic cancer.

## Materials and methods

### Patient samples

All pancreatic cancer samples and peritumoral samples were collected from patients attending the Tangdu Hospital of Air Force Medical University. All patients had a confirmed diagnosis according to the diagnostic criteria of the 2018 revised American Joint Committee on Cancer (AJCC) manual. The study involving the usage of patients’ tissues was performed in accordance with the Declaration of Helsinki and was approved by the Ethics Committee of the Tangdu Hospital of Air Force Medical University (approval no. 202003-093, date: 03.312020). All pancreatic cancer patients gave informed consent to join the experimental study. The pancreatic cancer tissue samples were frozen in liquid nitrogen until use. The data of pancreatic cancer and normal samples from TCGA, GSE62452, GSE16515, GSE28735, GSE15471, and GSE71729 databases are shown in Additional file 1: Tables S6-S10.

### Pancreatic cancer cells and lentivirus infection

In this study, MIAPaCa-2 (RRID: CVCL_0428), Capan-2 (RRID: CVCL_0026) and SW1990 cells (RRID: CVCL_1723) were purchased from Procell Life Science&Technology Company (Wuhan, China). All pancreatic cancer cells underwent short tandem repeat (STR) authentication and were mycoplasma-free. MIAPaCa-2 cells were maintained in Dulbecco’s modified Eagle medium (DMEM)/high glucose containing 10% fetal bovine serum (FBS), 100 U/mL penicillin, and 100 μg/mL streptomycin (HyClone, Utah, USA); SW1990 cells were maintained in complete Leibovitz’s L15 medium (Gibco BRL, Rockville, MD); and Capan-2 cells were cultured in complete McCoy’s 5A medium (HyClone, Utah, USA). The pLent-U6 and pLent-EF1a lentiviral plasmids, pCMV adenovirus plasmid, and packaging plasmids (pMD2G and psPAX2) were purchased from Vigene Biosciences (Jinan, China). For lentivirus packaging, 293 T cells were cultured in 100 mm plates at a concentration of 8 × 10^6^ cells per well. The next day, lentiviral plasmids (12 µg), pMD2G(3.6 µg), psPAX2 (7.2 µg), and 46 µL Lipofectamine 3000 (Invitrogen, California) were added to the 100 mms plate, and the supernatant was collected after 48 h. The supernatant was then filtered using 0.45 µm filter membranes. For cell infection, target cells were cultured in 100 mm plates at a concentration of 2 × 10^6^ per well. Lentivirus was added to the plates, and the medium containing lentivirus was replaced with fresh complete medium after 12 h. After 3 days, positive cells were selected using puromycin. The sequences of different shRNAs used are shown in Additional file 1: Table S1.

### Cell proliferation and colony formation assays

For the cell proliferation assay, tumor cells were cultured in 6-well plates in triplicate at a concentration of 3 × 10^4^ per well. After digestion, cell number in each well was detected using the blood cell counting plate (QIUJING, Shanghai, China). For the colony formation assay, tumor cells (10^3^/well) were seeded into 6-well plates in triplicate. After incubation for 14 days, 95% ethanol was used to fix the cells for 20 min, and then 1% crystal violet was used to perform cell staining for 30 min. The dishes were then washed with running water to visualize colony formation ability. The colonies were counted and the 6-well plates were photographed on a white background (Additional file 2).

### Transwell assays

Invasion assays were performed using transwell chambers coated with Matrigel (BD Biosciences) diluted 1:20 with cell culture medium. The diluted Matrigel was placed into the bottom of the chamber and incubated for 30 min. Migration assays also were performed without Matrigel. Briefly, cells were seeded directly into the Matrigel in 24-well plates at a concentration of 10^5^ cells/chamber, and medium with 10% FBS was added to the lower chamber. After 48 h, 95% ethanol was used to fix the cells for 20 min, and then 1% crystal violet was used to perform cell staining for 30 min. The cells were removed from the top surface of the membrane with a cotton swab. Then, the chamber was washed with running water and five random views were captured to visualize pancreatic cancer migration and invasion.

### Flow cytometry analysis

For flow cytometry, cells (10^6^ cells/well) were seeded into 6-well plates in triplicate and maintained for 24 h, and then cells were digested with trypsin and washed with PBS three times. Then, 70% ethanol was used to fix the cells for 20 min. Finally, the cells were stained with PI/RNase staining buffer (BD, USA) and subjected to flow cytometry for cell cycle detection (BD, FACSCalibur, USA).

### Coimmunoprecipitation (co-IP) and western blotting

All pancreatic cancer cells and tissue samples were lysed using RIPA lysis buffer (Applygen, Beijing, China) with phosphatase and protease inhibitors (Roche, USA). All cell lysates (200 µg protein) were incubated with immunoglobulin (Ig)G or first antibody for 3 h at 4 °C, and then the solution was incubated with protein A/G or anti-flag/HA magnetic beads (Bimake, Texas, USA) overnight. The co-IP complexes were washed five times with phosphate-buffered saline (PBS) and boiled with protein loading buffer. Then the complexes were run on sodium dodecyl sulfate–polyacrylamide gel electrophoresis (SDS-PAGE) to fractionate the proteins, which were then subjected to preformed mass spectrometry (MS) analysis (Mhelix Biotech, Shanghai, China) or immunoblotting. Western blotting was performed as previously described [11]. The primary antibodies are described in Additional file 1: Table S2. Protein bands were detected with enhanced chemiluminescence (ECL) western blotting luminescence reagent (Millipore, USA) using a BIO-RAD ChemiDoc XRS + imaging system (California, USA).

### Immunohistochemistry (IHC) analysis

One tissue microarray for cohort 1 and two tissue microarrays for cohort 2 were purchased from Outdo Biotech Company (Shanghai, China). IHC and tissue microarray (TMA) experimental procedures were performed as described previously [11]. The scores for immunostaining were assessed according to the staining intensity (0, no staining; 1, faint yellow; 2, reddish; and 3, brown) and the stained cells percentage (0, 0–5%; 1, 6–25%; 2, 26–50%; 3, 51–75%; and 4, 76–100%). The overall scores for immunostaining were obtained by multiplying the intensity score by the percentage of stained cells. A staining scores < 6 was described as low expression, while a score  > 6 was described as high expression. The digital images were analyzed using the Panoramic viewer 1.15.3 (3DHistech Ltd., Hungary).

### qRT-PCR

Total RNA was obtained using Trizol Reagent (Ambion, Texas USA) from pancreatic cancer cell samples according to the manufacturer’s protocol. SYBR Green qPCR Master Mix (Servicebio, Wuhan, China) was used to measure the quantity of *TMEM43*, *PRPF3*, and *GAPDH* genes by PCR (Bio-Rad, California, USA). The primer sequences used are described in Additional file 1: Table S3.

### Immunofluorescence assay

Tumor cells were washed twice with PBS (HyClone, Utah, USA), and fixed using 4% paraformaldehyde (Biosharp, Shanghai, China) for 15 min. Then, 0.3% Triton X-100 was used for permeation for 5 min and 5% BSA was used to block for 45 min. The following primary antibodies were used: rabbit anti-PRPF3 (A5482, Proteintech, China) and mouse anti-TMEM43 (SC-365298, Santa, USA), followed by the secondary antibodies Alexa Fluor 647-labeled anti-rabbit (ab150083, abcam, USA) and FITC-labeled anti-mouse (ab6785, abcam, USA). Cell images were captured on a Nikon Eclipse Ti-SR system.

### Label-free quantitative LC/MS proteomics analysis

TMEM43-silenced MIAPaCa-2 cells and control cells were cultured in 100 mm culture dishes in triplicate, respectively. After the density of cells reached 90%, cells were digested using the trypsin and washed three times using PBS. Finally, the cells were analyzed by label-free quantitative liquid chromatography-mass spectrometry (LC/MS) proteomics analysis by Mhelix Biotech Company (Shanghai, China).

### Animal studies

Male nude mice (3–5 weeks old, weight: 16~20 g) were purchased from the Air Force Military Medical University Animal Center (Xi’an, China), and kept in a specific pathogen-free (SPF) environment. Cells (5 × 10^6^) were injected into the right back of the nude mice (*n* = 6 per group). The tumor volumes were measured every three days (volume = longest diameter × shortest diameter^2^ × 0.5). After the experiment, the nude mice were anesthetized and sacrificed with 2% sodium pentobarbital (0.5 mL). The subcutaneous tumors were then excised weighed and imaged. Finally, tumors were fixed with 10% neutralized formalin until use. All nude mice experiments were conducted under an approved protocol by the Ethical Committee of Tangdu Hospital (approval no. 201903-18, date: 2019.03.05).

### Statistical analysis

The experimental data were analyzed with SPSS 19.0. Experimental data are described as mean ± SD. Differences between two samples were analyzed with an unpaired Student’s *t*-test. The correlations between mRNA/protein levels and clinicopathological parameters were analyzed with the χ^2^ test. Prognostic value was assessed with Cox regression analyses. A Kaplan–Meier curve was used to assess the association between mRNA/protein levels and overall survival (OS) and disease-free survival (DFS). All experiments were performed with at least three independent replicates. *p < 0.05, **p < 0.01, and ***p < 0.001 were suggested as statistically significant.

## Results

### *TMEM43* expression level is elevated and associated with poor survival in pancreatic cancer patients

**Fig. 1:**
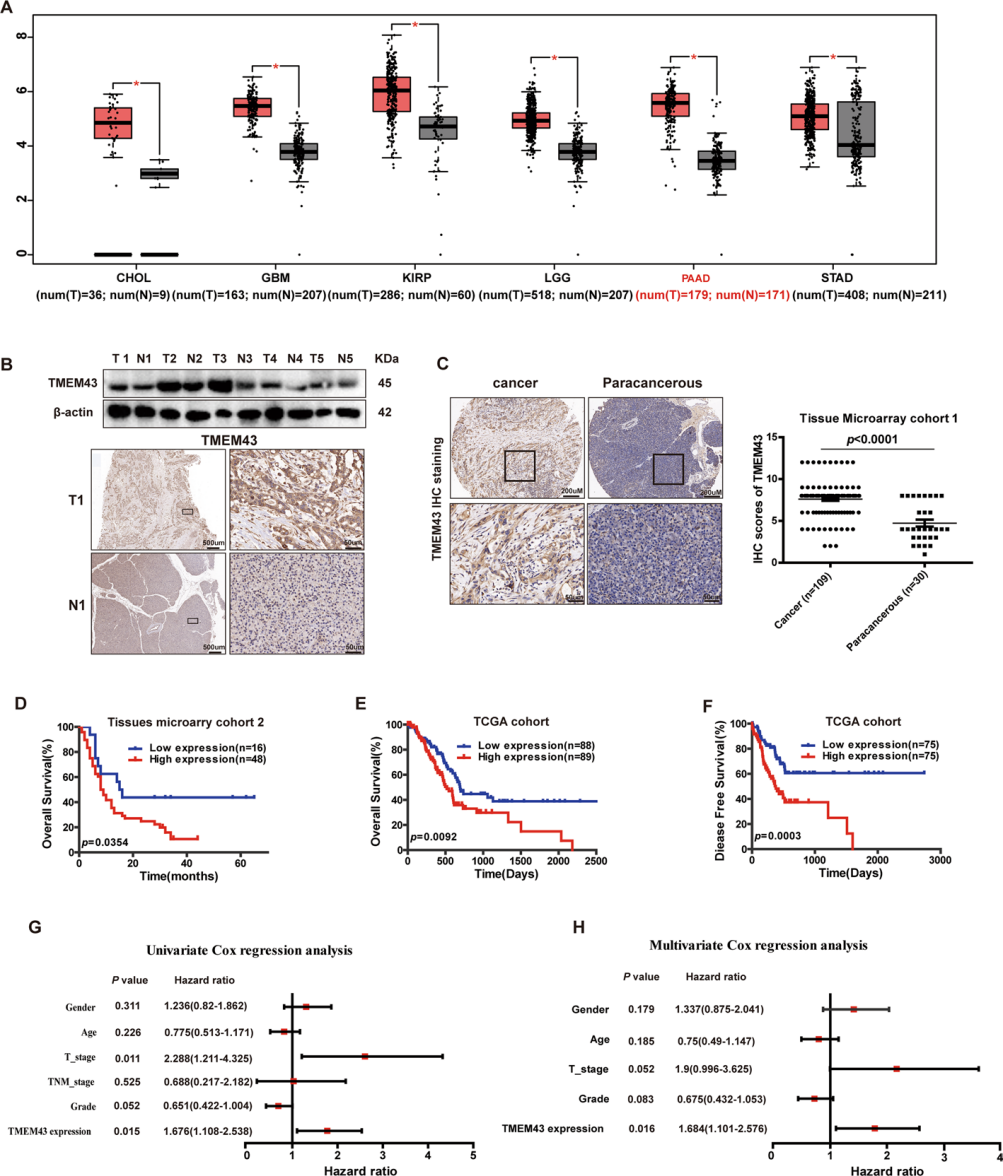
*TMEM43* expression is upregulated and associated with poor survival in pancreatic cancer. **A** *TMEM43* mRNA expression levels in different tumor samples and the corresponding normal samples (http://gepia.cancer-pku.cn/). **B** Western blotting and immunohistochemistry (IHC) assays showed the expression levels of TMEM43 in pancreatic cancer tissue samples and matched normal samples (*n* = 5). Scale bar (left) = 500 μm, scale bar (right) = 50 μm. **C** IHC showing the expression of TMEM43 in pancreatic cancer tissues and normal tissues in TMA cohort 1. Scale bar (left) = 200 μm, scale bar (right) = 50 μm. **D** Kaplan–Meier curve analysis showing the association of TMEM43 protein expression levels with patient survival using pancreatic cancer TMA cohort 2. **E**, **F** Kaplan–Meier curve analysis showing the correlation of TMEM43 mRNA levels with survival using the TCGA dataset. **G**, **H** Univariate and multivariate Cox regression analyses were performed in the TCGA pancreatic cancer dataset. *P < 0.05

First, we examined *TMEM43* mRNA levels in different tumor samples and corresponding control samples using the GEPIA database. *TMEM43* mRNA levels were significantly upregulated in cholangiocarcinoma (CHOL), glioblastoma (GBM), kidney renal papillary cell carcinoma (KIRP), brain lower grade glioma (LGG), pancreatic adenocarcinoma (PAAD), and stomach adenocarcinoma (STAD) samples compared with the corresponding control samples (*p* < 0.05, Fig. 1A). TMEM43 expression was also upregulated in five paired pancreatic cancer tissue samples and peritumoral samples using western blotting, and one sample pair was used to further confirm the result by IHC staining (Fig. 1B). The result of TMA cohort 1 samples from pancreatic cancer patients further confirmed that *TMEM43* expression level was obviously elevated in pancreatic cancer samples compared with control samples using IHC (*p* < 0.0001, Fig. 1C).

To assess the clinical relevance of TMEM43, the association between TMEM43 expression and clinical parameters were analyzed in 64 pancreatic cancer patients from TMA cohort 2. The result showed that TMEM43 expression was obviously correlated with T stage (*p* = 0.047, Table 1). Kaplan–Meier curve analysis was performed to assess the association between the TMEM43 level and survival of pancreatic cancer patients. The results found that patients with pancreatic cancer in TMA cohort 2 (*p* = 0.0354, Fig. 1D) with a high expression level of TMEM43 had worse OS, and patients in the cancer Genome Atlas (TCGA) database with a high expression level of TMEM43 had worse OS (*p* = 0.0092) and DFS (*p* = 0.0003) (Fig. 1E, F). Furthermore, univariate and multivariate analyses were used to analyze the prognostic value of TMEM43 as a biomarker in the TCGA database, and the T stage and TMEM43 expression may have significant prognostic value in pancreatic cancer patients using univariate analysis (*p* = 0.011, *p* = 0.015, respectively, Fig. 1G). TMEM43 was also identified as an independent predictive biomarker for the prognosis in patients with pancreatic cancer by multivariate analysis (*p* = 0.016, Fig. 1H). Taken together, these experimental data suggest that TMEM43 is upregulated in pancreatic cancer, and that TMEM43 may be an independent prognostic marker for pancreatic cancer patients.

**Table 1:** Relationship between the clinicopathological variables and TMEM43 expression in pancreatic cancer

Parameters	Total (*n*)	TMEM43 expression		
		Low expression	High expression	*p*-Value
Gender				
Male	36	9	27	
Female	28	7	21	1.00
Age(years)				
< 60	21	5	16	
≥ 60	43	11	32	0.878
T stage				
T 1–2	13	6	7	
T 3–4	47	9	38	0.047^*^
N stage				
N 0	37	12	25	
N 1–2	23	3	20	0.0919

*p < 0.05

### TMEM43 promotes pancreatic cancer cell proliferation, migration, and invasion in vitro

**Fig. 2:**
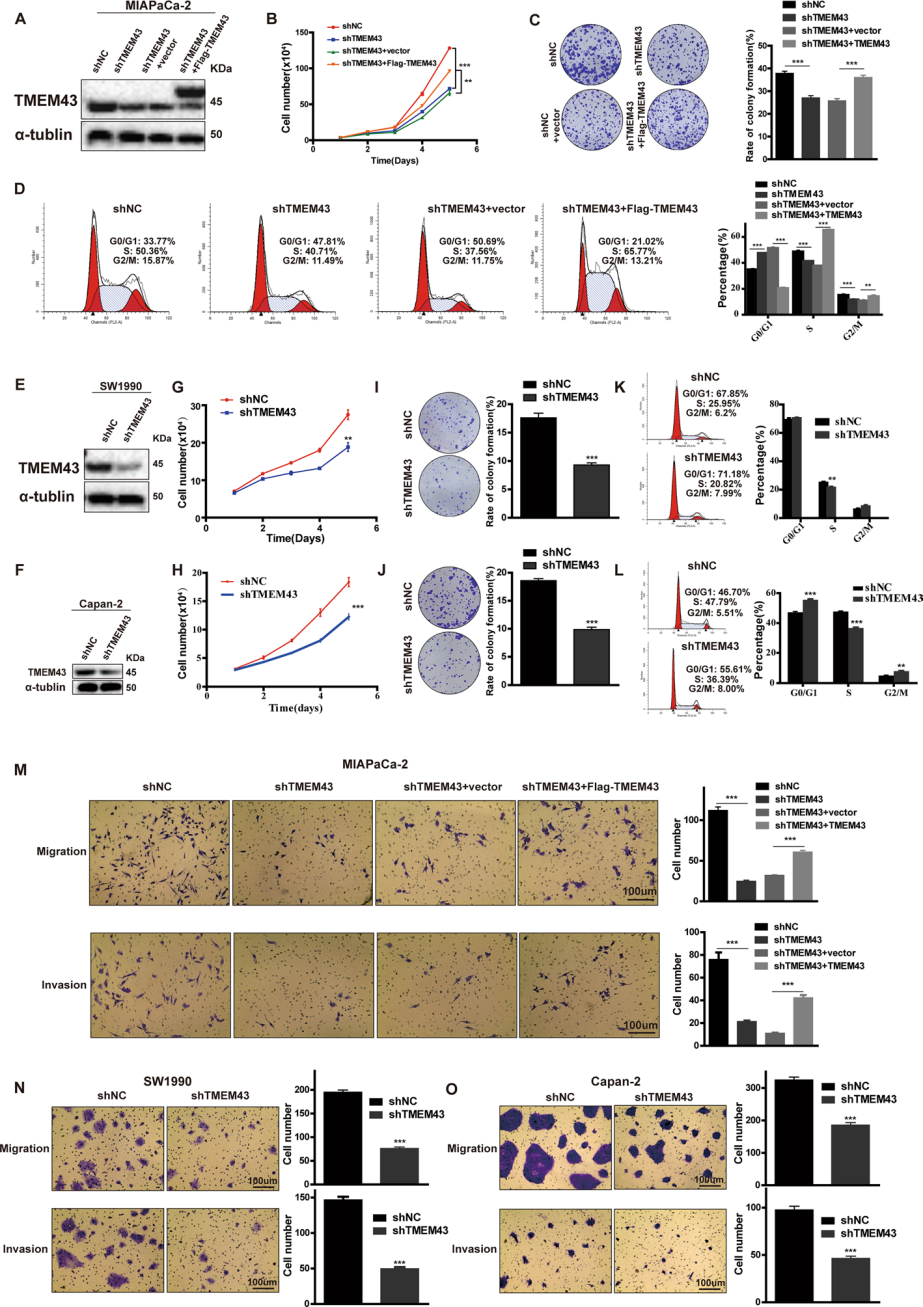
TMEM43 promotes pancreatic cancer proliferation, migration, and invasion in vitro. **A** The protein expression levels of TMEM43 were detected using western blotting in TMEM43-silenced cells and TMEM43-overexpressing MIAPaCa-2 cells. **B**, **C** Cell counting and colony formation assays showed the effects of silencing and overexpressing TMEM43 on MIAPaCa-2 cell proliferation. **D** The effects of silencing and overexpressing TMEM43 on pancreatic cancer cell cycle using flow cytometry assays. **E**, **F** The protein expression levels of TMEM43 were measured in TMEM43-knockdown SW1990, Capan-2 cells, and the corresponding control cells. **G**–**J** Cell counting and colony formation assays were performed in TMEM43-silenced SW1990, Capan-2 cells, and the corresponding control cells. **K**, **L** The cell cycle was analyzed in TMEM43-silenced SW1990, Capan-2 cells, and control cells with flow cytometry assays. **M**–**O** The migration and invasion abilities were detected in TMEM43-silenced MIAPaCa-2, SW1990, Capan-2 cells, TMEM43-overexpressing MIAPaCa-2 cells, and the corresponding control cells using transwell assays. Scale bar = 100 μm. Results are shown as the mean ± SD of three independent replicates. Scale bar = 100 μm. *p < 0.05, **p < 0.01, ***p < 0.001

To assess the function of TMEM43 in pancreatic cancer, we first established stable TMEM43 knockdown in MIAPaCa-2 cells and TMEM43-overexpression in TMEM43-silenced MIAPaCa-2 cells (Fig. 2A). The downregulation of TMEM43 significantly reduced tumor cell proliferation and colony formation abilities in MIAPaCa-2 cells, whereas the upregulation of TMEM43 in TMEM43-silenced MIAPaCa-2 cells and significantly promoted cell proliferation and colony formation abilities (Fig. 2B, C). The percentage of G0/1 phase and G2/M phase obviously increased, and the percentage of S phase was reduced in TMEM43-silenced MIAPaCa-2 cells compared with control cells using flow cytometry. TMEM43-overexpression in TMEM43-silenced MIAPaCa-2 cells had opposite results (Fig. 2D). In addition, TMEM43 was silenced in SW1990 and Capan-2 cells (Fig. 2E, F), and these results also confirmed that the downregulation of TMEM43 significantly reduced pancreatic cancer cell growth and colony formation abilities (Fig. 2G–J). Flow cytometry analysis showed that knockdown of TMEM43 obviously reduced the percentages of S phase in SW1990 and Capan-2 cells (Fig. 2K, L). Furthermore, transwell assays were used to explore the effect of TMEM43 on metastasis, which showed silencing TMEM43 obviously reduced tumor cell migration and invasion abilities in MIAPaCa-2, SW1990, and Capan-2 cells compared with the corresponding control cells (Fig. 2M–O). TMEM43-overexpression significantly promoted cell migration and invasion in TMEM43-silenced MIAPaCa-2 cells compared with the control group (Fig. 2M). Collectively, these results suggest that TMEM43 facilitates the growth, migration, and invasion of pancreatic cancer in vitro.

### TMEM43 accelerates the progression of pancreatic cancer via the RAP2B/ERK signaling pathway

**Fig. 3:**
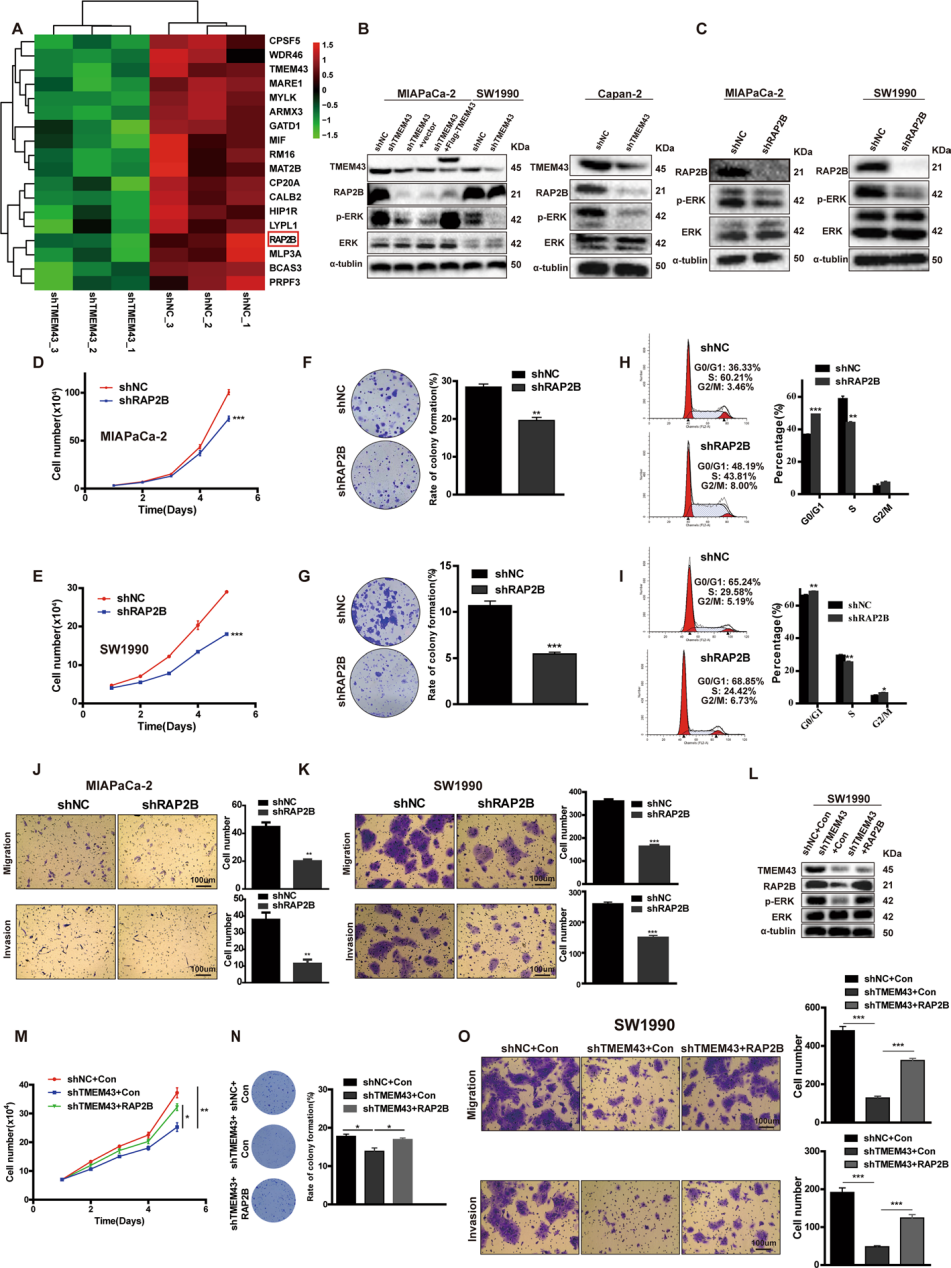
The RAP2B/ERK pathway is essential for TMEM43-mediated pancreatic cancer progression. **A** Heatmap showing proteins with partly differential expression in TMEM43-silenced MIAPaCa-2 cells and control cells. **B** RAP2B, ERK, and p-ERK protein expression levels were detected in TMEM43-silenced, TMEM43-overexpressing cells, and the corresponding control cells. **C** The indicated protein expression levels were measured in RAP2B-silenced cells and control cells. **D**, **E** Cell proliferation abilities were determined in RAP2B-silenced MIAPaCa-2 cells (**D**), SW1990 cells **E**, and the corresponding control cells using the cell counting assay. **F**, **G** Colony formation assays showed the effect of RAP2B expression levels on the growth abilities of MIAPaCa-2 cells **F** and SW1990 cells (**G**). **H**, **I** The cell cycle in RAP2B-silenced MIAPaCa-2 (**H**), SW1990 **I** cells, and the corresponding control cells were detected using flow cytometry assays. **J**, **K** The migration and invasion abilities were detected in RAP2B-knockdown MIAPaCa-2 (**J**), SW1990 cells **K**, and control cells. **L** Western blot showing the protein expression level in SW1990 cells stably transfected with control, shTMEM43, or shTMEM43 plus RAP2B. **M**–**O** Cell counting, colony formation, and transwell assays showing that the suppression of proliferation, migration, and invasion by shTMEM43 in SW1990 cells was partially abolished by RAP2B. Scale bar = 100 μm. Results are shown as the mean ± SD of three independent replicates. *p < 0.05, **p < 0.01, ***p < 0.001

To further identify the molecular mechanisms by which TMEM43 promotes pancreatic cancer progression, differentially expressed proteins mediated by TMEM43 were identified using label-free quantitative proteomics. The results showed that TMEM43 knockdown resulted in the downregulation of 243 proteins (fold change < 0.77) and the upregulation of 648 proteins (fold change > 1.3) (Additional file 1: Table S4). Some differentially expressed proteins are shown in Fig. 3A. Among these proteins, the RAP2B aroused our interest: RAP2B protein expression level was downregulated in TMEM43-knockdown MIAPaCa-2 cells compared with control cells. RAP2B has been reported to be an oncogene that may be correlated with cancer progression by regulating ERK phosphorylation levels [12–14]. TMEM43 also has been reported to promote ERK phosphorylation [10]. Furthermore, we confirmed that TMEM43 knockdown reduced RAP2B and phosphorylated ERK expression levels in MIAPaCa-2, SW1990, and Capan-2 cells compared with the corresponding control cells by western blotting (Fig. 3B). Additionally, overexpression of TMEM43 increased the expression levels of RAP2B and phosphorylated ERK in TMEM43-silenced MIAPaCa-2 cells compared with control cells (Fig. 3B).

To demonstrate the function of RAP2B in pancreatic cancer, it was stably silenced in SW1990 and MIAPaCa-2 cells, and the results show that the expression of phosphorylated ERK in RAP2B-silenced MIAPaCa-2 and SW1990 cells was reduced compared with the corresponding control cells (Fig. 3C). Moreover, cell counting and colony formation assays suggested that RAP2B knockdown reduced the growth and colony formation abilities of MIAPaCa-2 cells (Fig. 3D, F) and SW1990 cells (Fig. 3E, G) compared with control cells. The percentage of G0/1 phase was increased and the percentage of S phase was decreased in RAP2B-knockdown MIAPaCa-2 cells (Fig. 3H). Knockdown of RAP2B in SW1990 cells obviously increased the percentages of G0/1 phase and G2/M phase and decreased the percentage of S phase (Fig.3I). Transwell assays confirmed that RAP2B knockdown inhibited the migration and invasion of SW1990 and MIAPaCa-2 cells (Fig. 3J, K). To confirm that TMEM43 promotes pancreatic cancer progression by RAP2B, we overexpressed RAP2B in TMEM43-silenced SW1990 cells. The results show that RAP2B overexpression rescued the protein expression of RAP2B and p-ERK (Fig. 3L). Cell counting, colony formation, and transwell assays demonstrated that the reduced proliferation, migration, and invasion induced by silenced TMEM43 in SW1990 cells was partially rescued by overexpressing RAP2B (Fig. 3M–O). Taken together, these results suggest that TMEM43 promotes pancreatic cancer progression through the RAP2B/ERK axis.

### TMEM43 mediates PRPF3 protein stability by directly binding to PRPF3

**Fig. 4:**
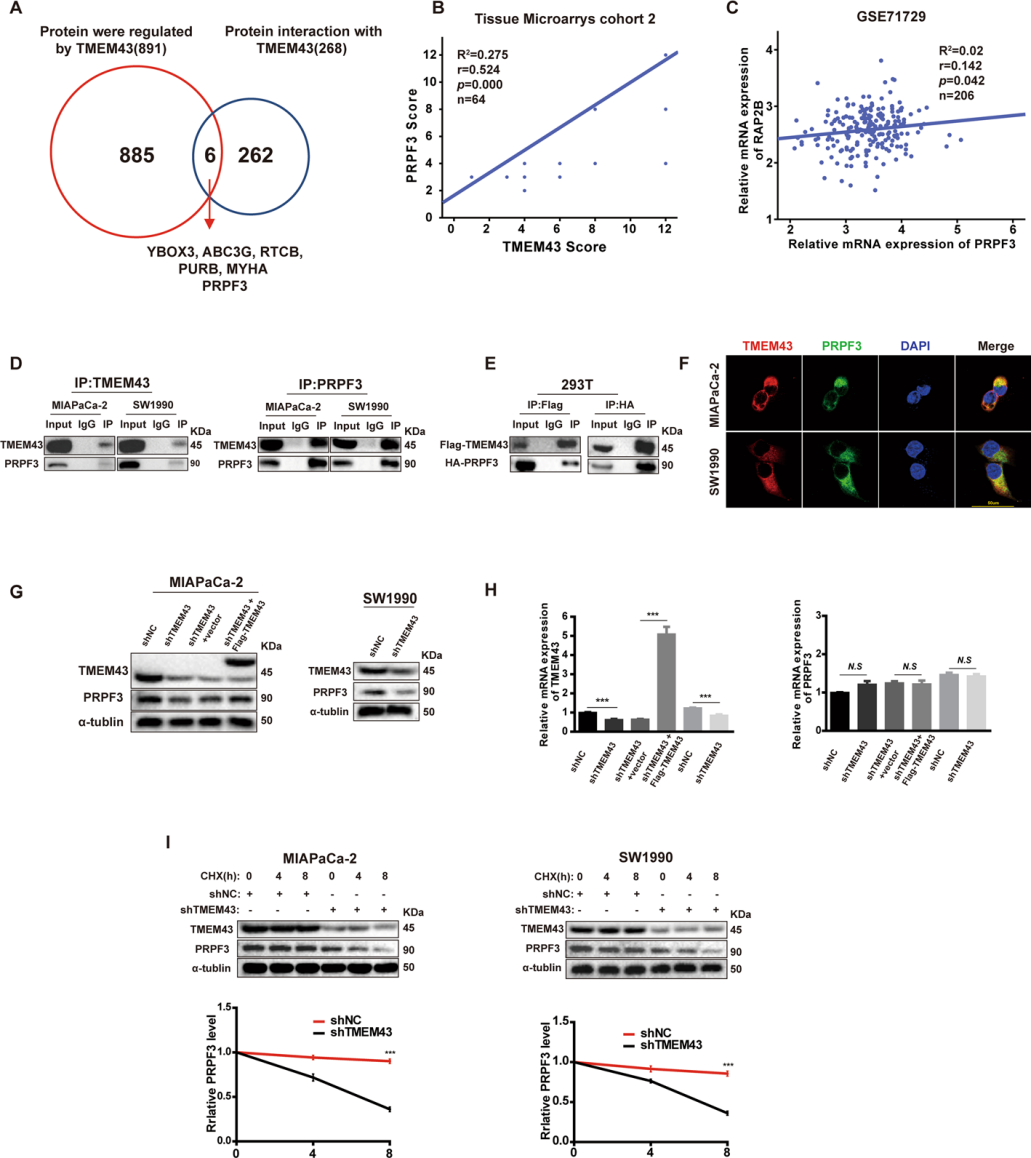
TMEM43 mediates PRPF3 protein stability by directly binding to PRPF3. **A** Venn diagram showing the proteins regulated by TMEM43 and the proteins that directly interact with TMEM43. **B** IHC showing the correlation of TMEM43 protein expression and PRPF3 protein expression. **C** The correlation of PRPF3 mRNA levels with RAP2B mRNA levels in the GSE71729 dataset. **D** Endogenous binding of TMEM43 and PRPF3 was detected using co-IP and western blot assays**. E** Co-IP and western blot assays were used to analyze the exogenous binding of TMEM43 and PRPF3. **F** Confocal immunofluorescence assays detected the location of TMEM43 and PRPF3 in pancreatic cancer cells. **G**, **H** The PRPF3 protein and mRNA levels were detected in TMEM43-silenced MIAPaCa-2, SW1990 cells, overexpressing TMEM43 MIAPaCa-2 cells, and the corresponding control cells. **I** The protein levels of PRPF3 in TMEM43-silenced MIAPaCa-2 and SW1990 cells and the corresponding control cells were detected after treatment with the protein synthesis inhibitor cycloheximide (CHX, 25 µg/mL). Results are shown as the mean ± SD of three independent replicates. *p < 0.05, **p < 0.01, ***p < 0.001

To explore the underlying mechanism by which TMEM43 facilitates pancreatic cancer progression by the RAP2B/ERK signaling pathway, co-IP followed by protein mass spectrometry (MS) was performed to identify the proteins that bind to TMEM43 in MIAPaCa-2 cells. Label-free quantitative proteomics and co-IP protein MS identified six proteins as candidate TMEM43-binding proteins (Additional file 1: Table S5; Fig. 4A). Among them, PRPF3 has been reported to be an oncogene involved in cancer development. Next, we analyzed the association of TMEM43 protein expression level with PRPF3 protein expression level using IHC, and the results suggest that TMEM43 protein levels were significantly positively associated with the PRPF3 protein levels in TMA cohort 2 (Fig. 4B). We also found that the PRPF3 mRNA levels was significantly positively associated with RAP2B mRNA levels in the GSE71729 dataset (Fig. 4C). To further confirm the interaction between TMEM43 and PRPF3, total proteins were immunoprecipitated using anti-TMEM43 or anti-PRPF3 antibodies. TMEM43 and PRPF3 were co-immunoprecipitated in SW1990 and MIAPaCa-2 cells (Fig. 4D). Moreover, the binding of exogenous TMEM43 and PRPF3 was demonstrated using IP/western blotting in HEK293 cells (Fig. 4E). Then, an immunofluorescence assay was performed to determine the expression and localization of TMEM43 and PRPF3 in SW1990 and MIAPaCa-2 cells, and found that TMEM43 and PRPF3 colocalized in the cytoplasm using confocal microscopy (Fig. 4F, Additional file 1: Fig S1).

To further understand the mechanism by which TMEM43 regulates PRPF3, we examined the protein level of PRPF3 and found that it was downregulated in TMEM43-silenced MIAPaCa-2 and SW1990 cells compared with control cells, whereas overexpression of TMEM43 increased PRPF3 protein levels in TMEM43-silenced MIAPaCa-2 cells compared with the control group (Fig. 4G). However, PRPF3 mRNA levels were unchanged after overexpressing or silencing TMEM43 (Fig. 4H). According to the above results, we speculated that TMEM43 may regulate the stability of PRPF3 in pancreatic cancer. To demonstrate our hypothesis, the protein synthesis inhibitor cycloheximide (CHX) was used to treat TMEM43-silenced MIAPaCa-2 and SW1990 cells and the corresponding control cells, and the results showed that PRPF3 was more unstable in TMEM43-knockdown SW1990 and MIAPaCa-2 cells (Fig. 4I). In addition, immunoprecipitation (IP) analysis showed enhanced polyubiquitination of PRPF3 bands upon MG132 treatment in TMEM43-knockdown SW1990 cells (Additional file 1: Fig S2). Taken together, these results suggest that TMEM43 regulates PRPF3 protein stability through the ubiquitination of PRPF3.

### PRPF3 promotes the progression of pancreatic cancer via the RAP2B/ERK signaling pathway

**Fig.5:**
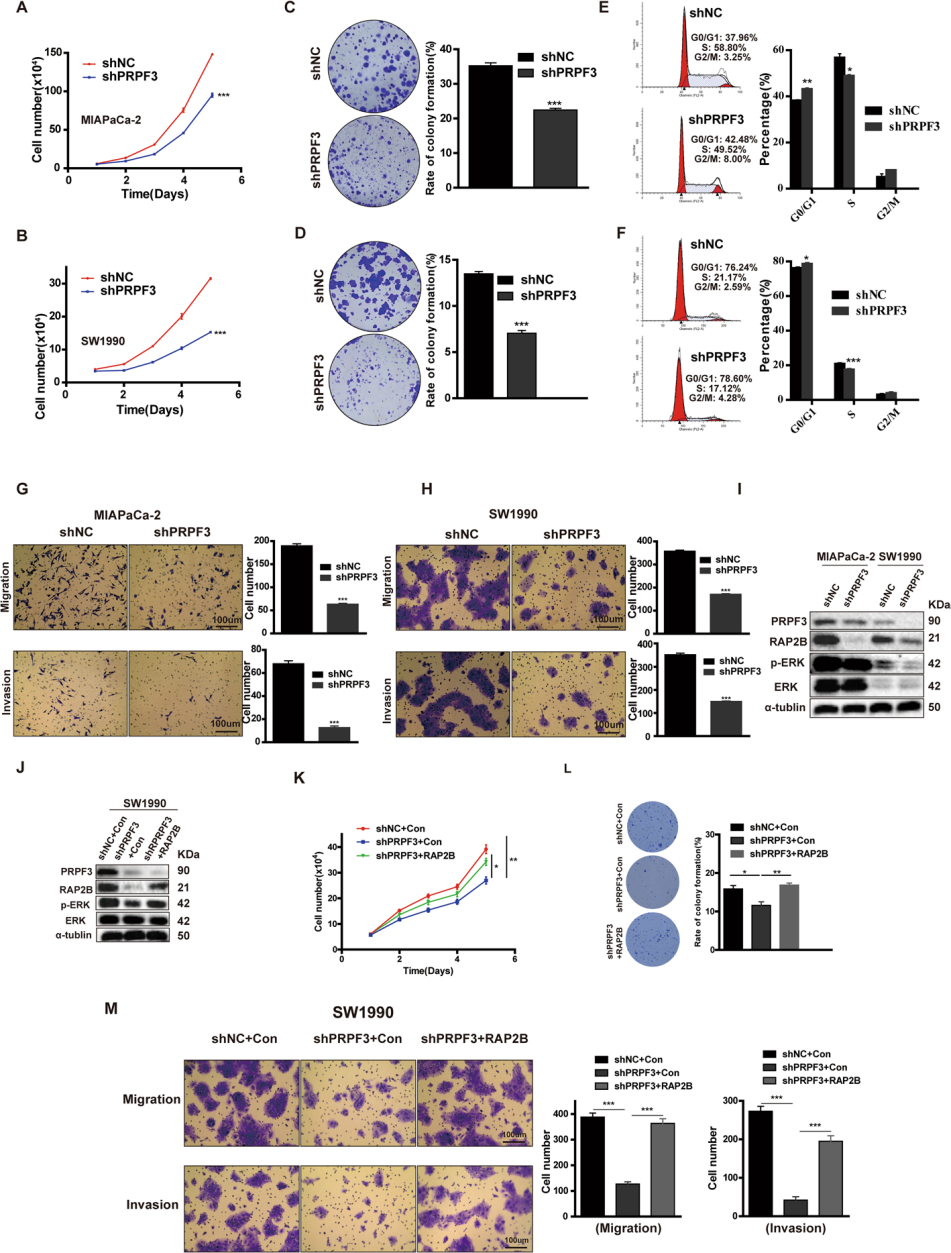
PRPF3 promotes pancreatic cancer proliferation, migration, and invasion in vitro via the RAP2B/ERK pathway. **A**, **B** Cell counting assays showed the effect of PRPF3 on pancreatic cancer cells proliferation in MIAPaCa-2 **A** and SW1990 **B** cells. **C**, **D** Colony formation assays were performed in MIAPaCa-2 (**C**) and SW1990 **D** cells. **E**, **F** The effect of PRPF3 on the cell cycle in MIAPaCa-2 (**E**) and SW1990 **F** cells were detected. **G**, **H** transwell assays were performed to detect the migration and invasion abilities of PRPF3-silenced MIAPaCa-2, and SW1990 cells and control cells. **I** The indicated protein expression levels in PRPF3-knockdown MIAPaCa-2, SW1990 cells, and control cells were detected. **J** Western blot results showing the indicated protein expression levels in SW1990 cells stably transfected with control, shPRPF3, and shPRPF3 + RAP2B. **K**–**M **Cell counting, colony formation, and transwell assays showing that the suppression of proliferation, migration, and invasion by shPRPF3 in SW1990 cells was partially abolished by RAP2B. Scale bar = 100 μm. Results are shown as the mean ± SD of three independent replicates. *p < 0.05, **p < 0.01, ***p < 0.001

To explore the function of PRPF3 in pancreatic cancer, we first established stable PRPF3 knockdown in MIAPaCa-2 and SW1990 cell lines. Cell counting and colony formation assays showed that knockdown of PRPF3 in MIAPaCa-2 and SW1990 cells significantly inhibited growth and colony formation abilities (Fig. 5A–D). Transwell assays demonstrated that PRPF3 knockdown inhibited the migration and invasion abilities of MIAPaCa-2 and SW1990 cells (Fig. 5G, H). Furthermore, the percentage of G0/1 phase was increased and the percentage of S phase was decreased in PRPF3-knockdown MIAPaCa-2 and SW1990 cells compared with control cells by flow cytometry analysis (Fig. 5E, F). Moreover, we demonstrated that the protein expression levels of RAP2B and phosphorylated ERK were decreased in PRPF3-silenced pancreatic cancer cells (Fig. 5I). To confirm that PRPF3 promoted pancreatic cancer progression via RAP2B, we overexpressed RAP2B in PRPF3-silenced SW1990 cells, and found that RAP2B overexpressing rescued RAP2B and p-ERK protein levels (Fig. 5J). Cell counting, colony formation and transwell assays demonstrated that the reduced proliferation, migration, and invasion induced by silencing of PRPF3 in SW1990 cells was partially rescued by overexpressed RAP2B (Fig. 5K–M). Together, our findings suggest that PRPF3 promotes pancreatic cancer (PC) progression via the RAP2B/ERK axis.

### TMEM43 promotes pancreatic cancer growth via the PRPF3/RAP2B axis in vivo

**Fig.6:**
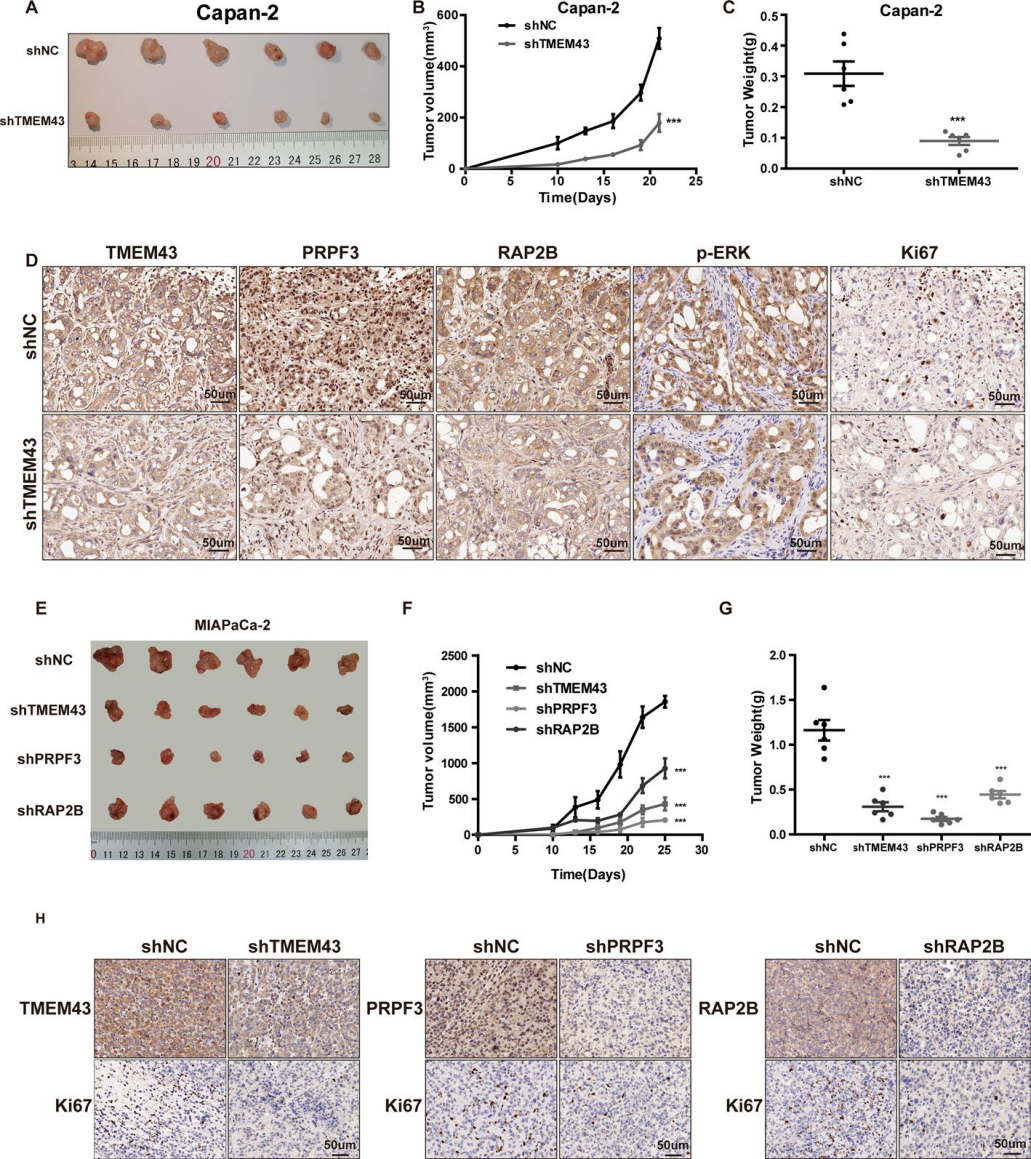
TMEM43 promotes pancreatic cancer growth via the PRPF3/RAP2B axis in vivo. **A** Xenografts from nude mice were obtained and measured. **B** The tumor volume in nude mice was detected and analyzed every three days in the TMEM43-silenced Capan-2 cells group and the control group. **C** Tumor weights were measured and analyzed in the TMEM43-silenced Capan-2 cells group and the control group. **D** IHC detected the expression of the indicated proteins in the two groups. **E** Xenografts from nude mice were obtained and measured after different treatments. **F** The tumor volume in nude mice were detected and analyzed every three days after different treatments. **G** Tumor weights were measured and analyzed after different treatments. **H** IHC detected the expression of the indicated proteins after different treatments. Results are shown as the mean ± SD of three independent replicates. Scale bar = 50 μm *p < 0.05, **p < 0.01, ***p < 0.001

We further assessed the effect of TMEM43 via regulating PRPF3/RAP2B axis on pancreatic cancer cell growth in vivo. First, knockdown of TMEM43 in Capan-2 and control cells were injected into the back of nude mice, and the results suggest that the tumor volume and weight were significantly decreased in TMEM43-silenced Capan-2 cells compared with control cells (Fig. 6A–C). IHC staining confirmed that TMEM43 expression was downregulated in TMEM43-knockdown Capan-2 cell xenografts compared with the control group (Fig. 6D). The expression of PRPF3, RAP2B, p-ERK, and Ki67 was dramatically reduced in TMEM43-silenced Capan-2 cells (Fig. 6D). Moreover, tumor xenografts were also performed to confirm the functions of PRPF3 and RAP2B in vivo, and the results showed that the downregulation of TMEM43, PRPF3, and RAP2B obviously reduced tumor growth in MIAPaCa-2 cells (Fig. 6E–G). The protein level of Ki67 was reduced in TMEM43-knockdown, PRPF3-knockdown, and RAP2B-knockdown MIAPaCa-2 cells compared with control cells (Fig. 6H).

### Clinical significance of the TMEM43/PRPF3/RAP2B axis in pancreatic cancer

**Fig.7:**
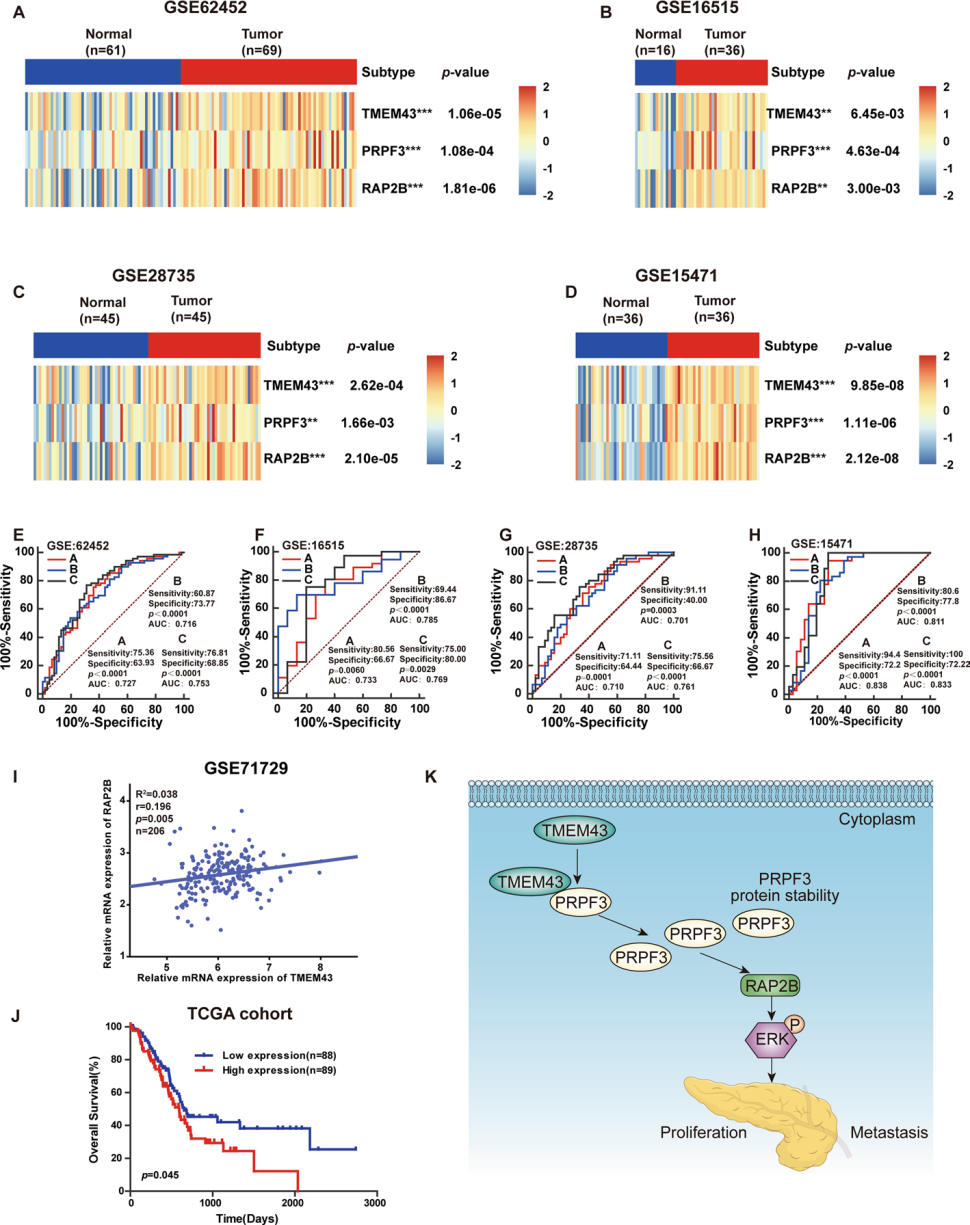
The clinical significance of the TMEM43/PRPF3/RAP2B axis in pancreatic cancer. **A**–**D** The mRNA levels of TMEM43, PRPF3, and RAP2B in pancreatic cancer samples compared with the corresponding control samples in the GSE62452, GSE16515, GSE28735, and GSE15471 datasets. **E**–**H** Receiver operating characteristic (ROC) curves were used to analyze the diagnostic value of TMEM43, PRPF3, and RAP2B. **I** The correlation of TMEM43 and RAP2B was analyzed in the GSE71729 dataset. **J **Kaplan–Meier curve analysis showed the correlation of RAP2B mRNA levels with patient survival using the TCGA dataset. **K** Illustration showing that TMEM43 promotes pancreatic cancer proliferation, migration, and invasion by the PRPF3/RAP2B/ERK axis

To assess the clinical significance of the TMEM43/PRPF3/RAP2B axis in promoting pancreatic cancer progression, TMEM43, PRPF3, and RAP2B expression levels in pancreatic cancer samples were examined and determined to be significantly upregulated compared with the corresponding control group in GSE62452, GSE16515, GSE28735, and GSE15471 datasets (Fig. 7A–D). Receiver operating characteristic (ROC) curve analysis suggested that TMEM43, PRPF3, and RAP2B had good diagnostic value in pancreatic cancer patients according to their prognostic value in the GSE62452, GSE16515, GSE28735, and GSE15471 datasets (Fig. 7E–H). Moreover, TMEM43 mRNA levels were significantly positively associated with RAP2B mRNA expression levels (Fig. 7I, Additional file 1: Fig S3). Kaplan–Meier curve analysis suggested that pancreatic cancer patients with high expression levels of RAP2B had worse OS (Fig. 7J).

## Discussion

Pancreatic cancer is a highly malignant tumor of the digestive system. Most patients are diagnosed at an advanced stage, mainly due to a lack of effective diagnostic markers, and tumors develop resistance to most radiation and chemotherapies [15]. Previous studies have shown that *TMEM43* expression levels are upregulated in high-grade glioma malignancy samples compared with normal control samples and low-grade glioma samples, and higher *TMEM43* expression is associated with poorer survival outcomes. In vitro and in vivo assays showed that knockdown of *TMEM43* inhibited glioma proliferation and metastasis [10]. However, the role of *TMEM43* has not been reported in pancreatic cancer. In our study, we showed that *TMEM43* expression levels were higher in pancreatic cancer samples than in control samples, and pancreatic cancer patients with higher *TMEM43* expression levels had shorter OS and DFS. Moreover, in vitro and in vivo assays showed that knockdown of *TMEM43* inhibited tumor growth, migration, and invasion in MIAPaCa-2, SW1990, and Capan-2 cells. *TMEM43*-overexpression facilitated tumor proliferation, migration, and invasion in MIAPaCa-2 cells in vitro. Taken together, these results suggest that *TMEM43* could be used as a diagnostic biomarker and therapeutic target for patients with pancreatic cancer.

Previous studies indicated that *TMEM43 *facilitated brain tumor progression by mediating *EGFR*-induced *NF-κB* activation and promoting the phosphorylation of *ERK* [10]. To understand the molecular mechanisms of *TMEM43* in pancreatic cancer, we identified differentially expressed proteins in the context of *TMEM43* knockdown using the quantitative technique of label-free protein MS, and found that *RAP2B* is an important downstream target of *TMEM43* in pancreatic cancer. Moreover, pancreatic cancer databases demonstrated that *TMEM43* mRNA level was significantly positively associated with *RAP2B* mRNA expression level; we speculated that *TMEM43* may regulate *RAP2B* expression through mediating *RAP2B* transcription level. *RAP2B*, as a member of the RAS subfamily, is upregulated in many different types of tumors [16]. Previous studies have shown that *RAP2B *plays a vital role in signal transduction, cell adhesion, proliferation, and metastasis in human tumor cells [17, 18]. The *RAP2B/ERK* signaling pathway is one of the important pathways that contributes to cell growth and metastasis in breast cancer, glioma, and hepatocellular carcinoma [12–14]. In addition,* RAP2B* accelerates tumor cell progression through the *PTEN/PI3K/VEGF* signaling pathway in renal cell carcinoma [19]. However, the function of *RAP2B* in pancreatic cancer is vague. In this study, we found that silencing* RAP2B* could reduce the proliferation, migration, and invasion of pancreatic cancer cells via enhancing the phosphorylation of ERK. We further demonstrated that *TMEM43* promoted pancreatic cancer progression via the *RAP2B/ERK* pathway.

To further understand the molecular mechanism by which *TMEM43* mediates the *RAP2B/ERK* pathway, the proteins interacting with *TMEM43* were identified by protein mass spectrometry. *PRPF3* not only interacted with *TMEM43* but could also be regulated by *TMEM43*. IHC also demonstrated that *TMEM43* protein levels ere significantly positively correlated with *PRPF3* protein levels. In addition, *PRPF3* mRNA levels were significantly positively correlated with *RAP2B* mRNA levels using the GSE71729 dataset of pancreatic cancer. We further confirmed the interaction between *TMEM43* and *PRPF3* by western blot and co-IP. Confocal immunofluorescence assays demonstrated that *TMEM43* and *PRPF3* colocalized in pancreatic cancer cell cytoplasm. *PRPF3* protein levels were downregulated in *TMEM43*-knockdown cells compared with control cells, but the mRNA expression levels were not different between *TMEM43*-silenced cells and control cells. Overexpression of *TMEM43* in pancreatic cancer cells was consistent with these results. Our studies further demonstrate that *TMEM43* induced polyubiquitination-mediated proteasomal degradation of *PRPF3*.* PRPF3*, a pre-mRNA processing factor, is an important component of the U4/U6-U5 tri-snRNP complex [20]. At present, we know that the SUMOylation of *PRPF3* is responsible for the formation of the U4/U6U5 tri-snRNP and participates in spliceosome assembly [21], but its role and molecular mechanism in pre-mRNA alternative splicing are poorly understood. Previous studies reported that *PRPF3* and *PRPF3* mutations are associated with autosomal dominant retinitis pigmentosa in Chinese individuals [22–24]. *PRPF3* was reported to be a potential prognostic indicator in hepatocellular carcinoma [25] and promoted the cell growth, migration, and invasion of keratinocyte-derived cutaneous squamous cell carcinoma via the *JAK2/STAT3* pathway [26]. In keeping with this, we confirmed that the expression of *PRPF3* was significantly elevated in pancreatic cancer samples compared with the control group, signifying that it may play an oncogenic role in pancreatic cancer. We demonstrated that *PRPF3* knockdown could significantly inhibit cell growth, migration, invasion, and the* RAP2B/ERK* signaling pathway in pancreatic cancer. Collectively, these results suggest that *TMEM43* mediates pancreatic cancer progression through the *PRPF3/RAP2B/ERK* signaling pathway. However, the molecular mechanism by which *PRPF3* mediates the* RAP2B/ERK* pathway needs to be further explored.

## Conclusions

In summary, our findings identify *TMEM43* as a putative novel oncogene, the expression of which is upregulated in pancreatic cancer. Higher expression of *TMEM43* is associated with poorer OS and DFS outcomes. *TMEM43* mediates the *RAP2B/ERK* pathway by binding to and stabilizing *PRPF3* to promote pancreatic cancer progression (Fig. 7K). Our study provides novel insights into the underlying molecular mechanisms of pancreatic cancer and highlights *TMEM43* as a novel potential prognostic marker and therapeutic target for pancreatic cancer.

## Supplementary Information

**Additional file 1. **Additional tables and figures.

**Additional file 2.** Original data.

## Data Availability

The datasets used in the current study are available from the corresponding author on reasonable request.

## Abbreviations

TMEM43: Transmembrane protein 43
PRPF3: Pre-mRNA processing factor 3
RAP2B: Ras-related protein Rap-2b
TCGA: The Cancer Genome Atlas
ROC: Receiver operating characteristic
SDS–PAGE: Sodium dodecyl sulfate–polyacrylamide gel electrophoresis
PVDF: Polyvinylidene fluoride
IHC: Immunohistochemistry
qRT-PCR: Reverse transcription quantitative real-time PCR
Co-IP: Coimmunoprecipitation
OS: Overall survival
DFS: Disease free survival
