# Correction: TMEM43 promotes pancreatic cancer progression by stabilizing PRPF3 and regulating RAP2B/ERK axis

**DOI:** 10.1186/s11658-023-00419-y

**Published:** 2023-01-18

**Authors:** Junqiang Li, Yang Song, Chao Zhang, Ronglin Wang, Lei Hua, Yongdong Guo, Dongxue Gan, Liaoliao Zhu, Shanshan Li, Peixiang Ma, Cheng Yang, Hong Li, Jing Yang, Jingjie Shi, Xiaonan Liu, Haichuan Su

**Affiliations:** 1 Department of Oncology, Tangdu Hospital, Air Force Medical University, Xi’an, 710038 Shaanxi China; 2 Ambulatory Surgery Center, Xijing Hospital, Air Force Medical University, Xi’an, 710032 Shaanxi China

**Correction: Cellular & Molecular Biology Letters (2022) 27:24** 10.1186/s11658-022-00321-z.

Following publication of the original article [1], the authors identified an error in Fig. 3O. The invasion image in the shTMEM43 + RAP2B group of Fig. 3O was inadvertently placed by mistake. We have double checked the original data and found that the inadvertent errors occurred during picture compilation, and this correction does not change the scientific conclusions of the article. The incorrect and the correct figure is given below.

The incorrect Fig. 3 is:

**Figure:**
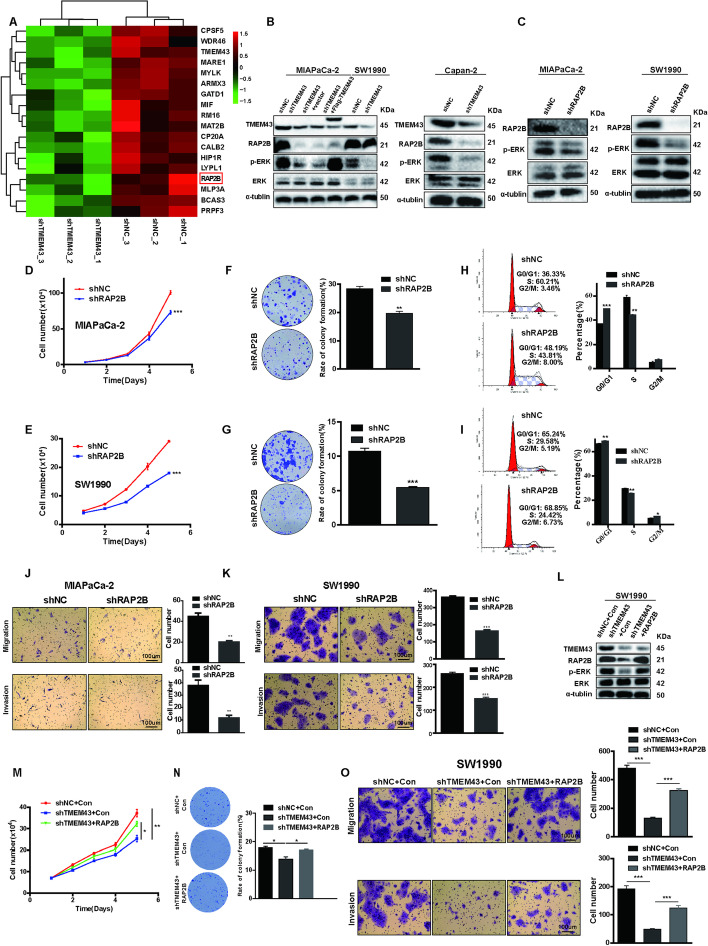
The RAP2B/ERK pathway is essential for TMEM43-mediated pancreatic cancer progression. **A** Heatmap showing proteins with partly differential expression in TMEM43-silenced MIAPaCa-2 cells and control cells. **B** RAP2B, ERK, and p-ERK protein expression levels were detected in TMEM43-silenced, TMEM43-overexpressing cells, and the corresponding control cells. **C** The indicated protein expression levels were measured in RAP2B-silenced cells and control cells. **D**,** E** Cell proliferation abilities were determined in RAP2B-silenced MIAPaCa-2 cells (**D**), SW1990 cells **E**, and the corresponding control cells using the cell counting assay. **F, G** Colony formation assays showed the effect of RAP2B expression levels on the growth abilities of MIAPaCa-2 cells **F** and SW1990 cells (**G**). **H**,** I** The cell cycle in RAP2B-silenced MIAPaCa-2 (**H**), SW1990 **I** cells, and the corresponding control cells were detected using flow cytometry assays. **J**,** K** The migration and invasion abilities were detected in RAP2B-knockdown MIAPaCa-2 (**J**), SW1990 cells **K**, and control cells. **L** Western blot showing the protein expression level in SW1990 cells stably transfected with control, shTMEM43, or shTMEM43 plus RAP2B. **M–O** Cell counting, colony formation, and transwell assays showing that the suppression of proliferation, migration, and invasion by shTMEM43 in SW1990 cells was partially abolished by RAP2B. Scale bar = 100 μm. Results are shown as the mean ± SD of three independent replicates. *p < 0.05, **p < 0.01, ***p < 0.001

The correct Fig. 3 is:

**Fig. 3:**
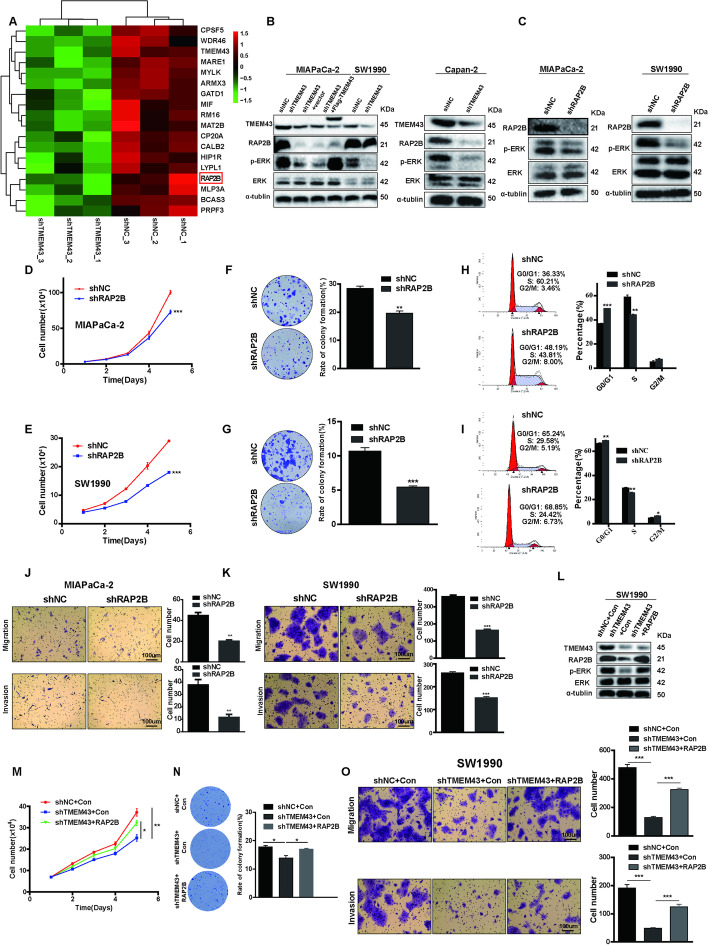
The RAP2B/ERK pathway is essential for TMEM43-mediated pancreatic cancer progression. **A** Heatmap showing proteins with partly differential expression in TMEM43-silenced MIAPaCa-2 cells and control cells. **B** RAP2B, ERK, and p-ERK protein expression levels were detected in TMEM43-silenced, TMEM43-overexpressing cells, and the corresponding control cells. **C** The indicated protein expression levels were measured in RAP2B-silenced cells and control cells. **D**,** E** Cell proliferation abilities were determined in RAP2B-silenced MIAPaCa-2 cells (**D**), SW1990 cells (**E**), and the corresponding control cells using the cell counting assay. **F**,** G** Colony formation assays showed the effect of RAP2B expression levels on the growth abilities of MIAPaCa-2 cells (**F**) and SW1990 cells (**G**). **H**,** I** The cell cycle in RAP2B-silenced MIAPaCa-2 (**H**), SW1990 cells (**I**), and the corresponding control cells were detected using flow cytometry assays. **J, K** The migration and invasion abilities were detected in RAP2B-knockdown MIAPaCa-2 (**J**), SW1990 cells (**K**), and control cells. **L** Western blot showing the protein expression level in SW1990 cells stably transfected with control, shTMEM43, or shTMEM43 plus RAP2B. **M–O** Cell counting, colony formation, and transwell assays showing that the suppression of proliferation, migration, and invasion by shTMEM43 in SW1990 cells was partially abolished by RAP2B. Scale bar = 100 μm. Results are shown as the mean ± SD of three independent replicates. *p < 0.05, **p < 0.01, ***p < 0.001
