# Supplementary material for: TMEM43 promotes pancreatic cancer progression by stabilizing PRPF3 and regulating RAP2B/ERK axis
Source: Cell Mol Biol Lett. 2022 Mar 8;27:24. doi: 10.1186/s11658-022-00321-z (PMC8903684; doi:10.1186/s11658-022-00321-z)
Supplement: Supplementary file 1 — Additional file 1. Additional tables and figures. [file 11658_2022_321_MOESM1_ESM.docx]

Additional file 1: Fig S1


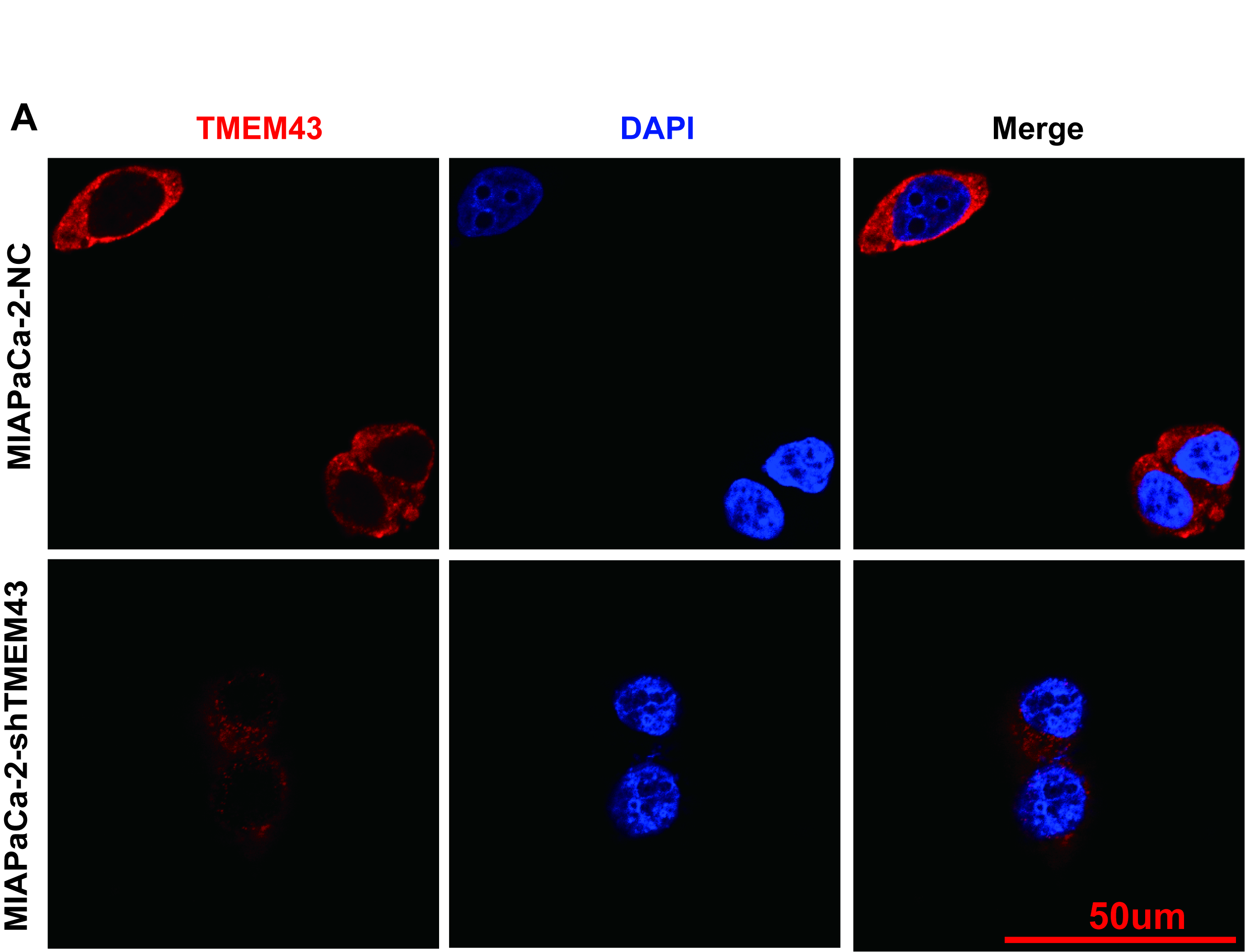


**A.** The expression and localization of TMEM43 in TMEM43-silenced MIAPaCa-2 cells and control cells using immunofluorescence assay (Scale bar **=**50μm).

Additional file 1: Fig S2


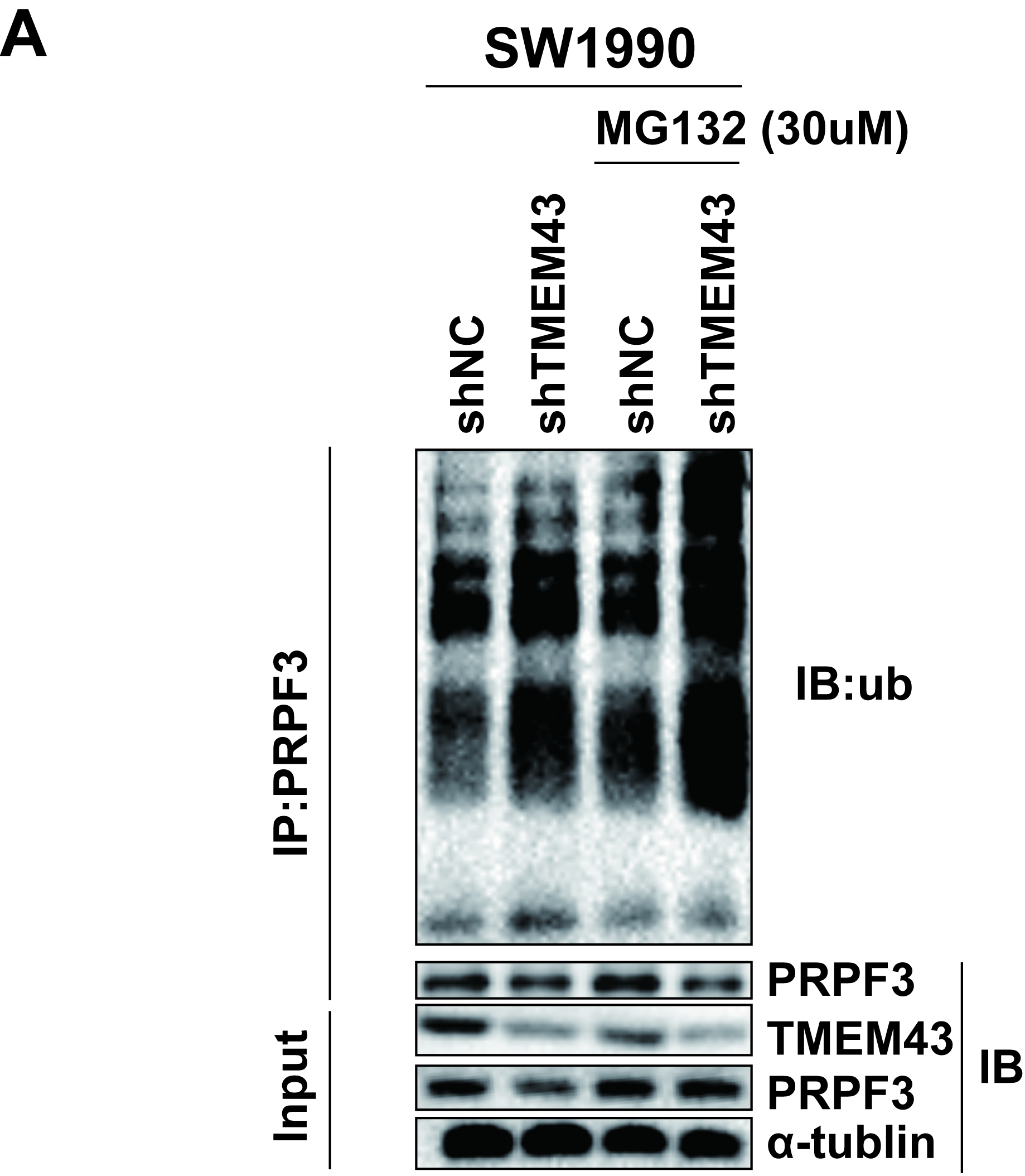


**A.** IP and western blot analysis of the indicated protein expression levels in pretreated with or without MG132 (30 µM).

Additional file 1: Fig S3


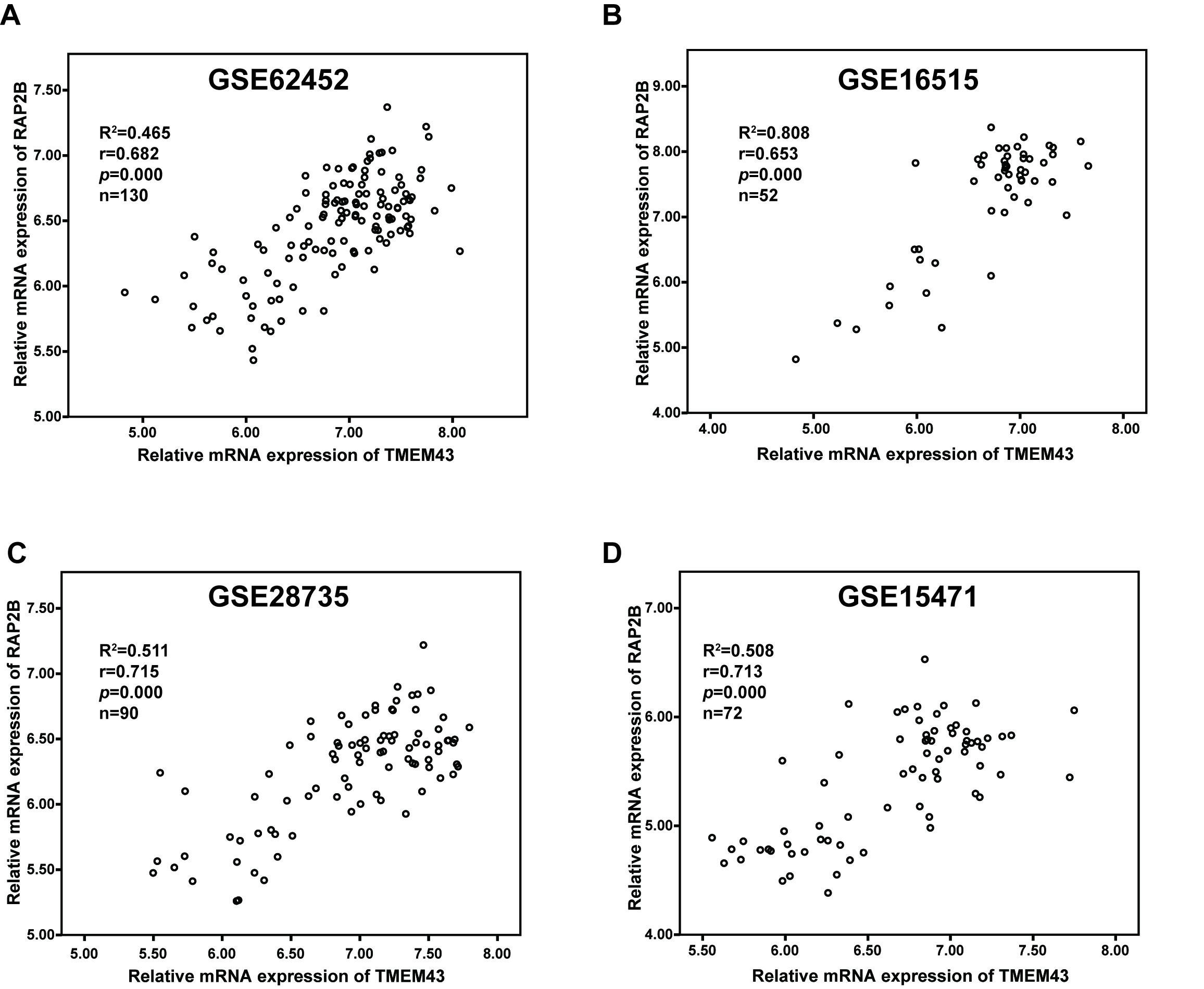


**A-D.** The correlation of TMEM43 and RAP2B was analyzed in the GSE62452, GSE16515, GSE28735, GSE15471 databases.

Additional file 1: Table S1:Knockdown shRNA sequences used in this study

| TRC Number | Squence |
| --- | --- |
| TMEM43-shRNA | GCGAATTATTCCAGTACCAGTTTCAAGAGAACTGGTACTGGAATAATTCGCTTTTTT |
| PRPF3-shRNA | CCGGCAGTTACTCTGGGAGTATATCCTCGAGGATATACTCCCAGAGTAACTGTTTTTG |
| RAP2B-shRNA | CCGGGCCTCGGTAGACGAGCTATTTCTCGAGAAATAGCTCGTCTACCGAGGCTTTG |

Additional file 1: Table S2: Different antibodies used in this study

| Antibody name | Sources |
| --- | --- |
| TMEM43(WB, IHC) | Abcam, ab184164 |
| TMEM43(IF) | Santa,SC-365298 |
| PRPF3(WB)  PRPF3(IHC,IF) | Proteintech, 10106-1-AP  Abclonal, A5482 |
| RAP2B(WB)  RAP2B(IHC)  Ubiquitin | Abclonal, A4071  GeneTex, GTX114702  CST, 3936 |
| Flag  HA  ERK  p-ERK  [α](https://baike.so.com/doc/50483-52891.html)-tublin  β-actin | Sigma, F1804  Proteintech, 51064-2-AP  Proteintech, 16443-1-AP  CST, 4370  Proteintech, 66031-1-Ig  Proteintech, 66009-1-Ig |

Additional file 1: Table S3: Different primer sequences of this study

| Primer Name | Primer sequences |
| --- | --- |
| TMEM43 primers: |  |
| TMEM43 sense primer: | 5'- TCGCTTGTGGTGTCTCCCG-3' |
| TMEM43 antisense prmer: | 5'- TGCTGTTGATGATTTCTGACCTCC-3' |
| PRPF3 primers  PRPF3 sense primer | 5'- ATGCTGACTAAGCTCCAGATCAAAC-3' |
| PRPF3 antisense primer  RAP2B primer | 5'- GCATTGCCGATGAGTCCTG-3' |
| RAP2B sense primer | 5'- GGAAGCTTGTCATCAATGGAAATC-3' |
| RAP2B antisense primer | 5'- TGATGACCCTTTTGGCTCCC-3' |

Additional file 1: Table S4:Label-free quantitative proteomics analysis of differentially expressed proteins in TMEM43-silenced MIAPaCa-2 cells and control cells

| Protein | Symbol | shTMEM43-VS-shNC FC | *p* value |
| --- | --- | --- | --- |
| Q15746 | MYLK | 0.16 | 0.000 |
| Q96AA3 | RFT1 | 0.20 | 0.005 |
| P07992 | ERCC1 | 0.20 | 0.016 |
| A5D8V6 | VP37C | 0.21 | 0.002 |
| O75486 | SUPT3 | 0.22 | 0.001 |
| O14920 | IKKB | 0.23 | 0.025 |
| Q9UH62 | ARMX3 | 0.24 | 0.001 |
| Q99807 | COQ7 | 0.25 | 0.013 |
| Q9H0R4 | HDHD2 | 0.25 | 0.001 |
| Q9H0W8 | SMG9 | 0.25 | 0.005 |
| O14730 | RIOK3 | 0.27 | 0.004 |
| O00458 | IFRD1 | 0.28 | 0.046 |
| Q7Z7H8 | RM10 | 0.28 | 0.000 |
| Q5T440 | CAF17 | 0.30 | 0.002 |
| P52747 | ZN143 | 0.31 | 0.001 |
| Q86TI2 | DPP9 | 0.31 | 0.010 |
| Q16563 | SYPL1 | 0.33 | 0.000 |
| Q86T03 | PP4P1 | 0.33 | 0.000 |
| Q96S97 | MYADM | 0.35 | 0.001 |
| Q8NEY8 | PPHLN | 0.36 | 0.011 |
| P78345 | RPP38 | 0.36 | 0.002 |
| Q5T1C6 | THEM4 | 0.36 | 0.004 |
| Q96EY8 | MMAB | 0.37 | 0.023 |
| Q8NHP6 | MSPD2 | 0.38 | 0.001 |
| O75477 | ERLN1 | 0.38 | 0.018 |
| Q8IZQ5 | SELH | 0.39 | 0.001 |
| P04732 | MT1E | 0.39 | 0.021 |
| Q9UHR4 | BI2L1 | 0.39 | 0.007 |
| P50747 | BPL1 | 0.39 | 0.002 |
| Q9UGP4 | LIMD1 | 0.40 | 0.006 |
| Q96QR8 | PURB | 0.40 | 0.000 |
| Q9NZJ6 | COQ3 | 0.40 | 0.002 |
| Q9H223 | EHD4 | 0.40 | 0.008 |
| Q9HC16 | ABC3G | 0.41 | 0.003 |
| P13473 | LAMP2 | 0.42 | 0.002 |
| Q9BRS2 | RIOK1 | 0.42 | 0.010 |
| P33527 | MRP1 | 0.42 | 0.002 |
| Q96BJ3 | AIDA | 0.42 | 0.003 |
| Q9NQ55 | SSF1 | 0.42 | 0.005 |
| Q6P158 | DHX57 | 0.42 | 0.018 |
| Q96RU2 | UBP28 | 0.43 | 0.005 |
| Q8NE86 | MCU | 0.43 | 0.042 |
| O95249 | GOSR1 | 0.43 | 0.009 |
| P16070 | CD44 | 0.44 | 0.030 |
| Q8NCC3 | PAG15 | 0.44 | 0.009 |
| Q96B49 | TOM6 | 0.45 | 0.035 |
| Q9UID3 | VPS51 | 0.45 | 0.008 |
| Q9NVH1 | DJC11 | 0.45 | 0.000 |
| Q71SY5 | MED25 | 0.46 | 0.001 |
| Q6P587 | FAHD1 | 0.46 | 0.003 |
| O75146 | HIP1R | 0.47 | 0.011 |
| Q5VWZ2 | LYPL1 | 0.47 | 0.024 |
| Q6UW02 | CP20A | 0.48 | 0.009 |
| P22676 | CALB2 | 0.48 | 0.002 |
| Q9NX20 | RM16 | 0.48 | 0.009 |
| Q9BTV4 | TMEM43 | 0.48 | 0.003 |
| Q96II8 | LRCH3 | 0.48 | 0.004 |
| Q8WUU5 | GATD1 | 0.49 | 0.005 |
| Q8WUH6 | TM263 | 0.49 | 0.009 |
| P00352 | AL1A1 | 0.49 | 0.000 |
| Q9NNW5 | WDR6 | 0.49 | 0.017 |
| P49247 | RPIA | 0.49 | 0.027 |
| Q9UPW0 | FOXJ3 | 0.49 | 0.009 |
| Q9BXR0 | TGT | 0.50 | 0.031 |
| Q9P2X0 | DPM3 | 0.50 | 0.009 |
| Q5BKY9 | F133B | 0.50 | 0.000 |
| P11169 | GTR3 | 0.50 | 0.030 |
| Q9BWM7 | SFXN3 | 0.51 | 0.036 |
| O95716 | RAB3D | 0.51 | 0.014 |
| Q16539 | MK14 | 0.51 | 0.006 |
| Q03405 | UPAR | 0.51 | 0.005 |
| Q9ULX6 | AKP8L | 0.51 | 0.043 |
| Q9Y4U1 | MMAC | 0.51 | 0.021 |
| Q7L8L6 | FAKD5 | 0.51 | 0.022 |
| Q6IQ22 | RAB12 | 0.52 | 0.024 |
| O60293 | ZC3H1 | 0.52 | 0.035 |
| Q9NR19 | ACSA | 0.52 | 0.040 |
| P05386 | RLA1 | 0.52 | 0.020 |
| P98179 | RBM3 | 0.53 | 0.004 |
| P22681 | CBL | 0.53 | 0.009 |
| Q9UGP8 | SEC63 | 0.53 | 0.015 |
| Q15125 | EBP | 0.53 | 0.012 |
| Q9BUB7 | TMM70 | 0.53 | 0.012 |
| Q01650 | LAT1 | 0.54 | 0.013 |
| Q9BZL6 | KPCD2 | 0.54 | 0.002 |
| Q14254 | FLOT2 | 0.54 | 0.010 |
| Q8IYL3 | CA174 | 0.55 | 0.022 |
| P83881 | RL36A | 0.55 | 0.027 |
| O95486 | SC24A | 0.55 | 0.014 |
| Q13671 | RIN1 | 0.55 | 0.044 |
| O75208 | COQ9 | 0.55 | 0.001 |
| Q9NXG2 | THUM1 | 0.55 | 0.013 |
| Q9UPU5 | UBP24 | 0.56 | 0.020 |
| P61225 | RAP2B | 0.56 | 0.013 |
| Q8IV38 | ANKY2 | 0.56 | 0.045 |
| Q9H6U6 | BCAS3 | 0.56 | 0.002 |
| Q15691 | MARE1 | 0.56 | 0.001 |
| Q9H492 | MLP3A | 0.56 | 0.009 |
| Q96T83 | SL9A7 | 0.56 | 0.048 |
| Q59GN2 | R39L5 | 0.57 | 0.006 |
| O15533 | TPSN | 0.57 | 0.019 |
| Q96E11 | RRFM | 0.57 | 0.042 |
| P51151 | RAB9A_ | 0.57 | 0.017 |
| Q8WW12 | PCNP | 0.57 | 0.007 |
| Q16222 | UAP1 | 0.57 | 0.011 |
| Q6XZF7 | DNMBP | 0.57 | 0.021 |
| Q4KWH8 | PLCH1 | 0.58 | 0.000 |
| P30046 | DOPD | 0.58 | 0.005 |
| O75376 | NCOR1 | 0.58 | 0.007 |
| Q9NR09 | BIRC6 | 0.58 | 0.012 |
| Q8N3X1 | FNBP4 | 0.58 | 0.005 |
| P32321 | DCTD | 0.58 | 0.000 |
| P82912 | RT11 | 0.58 | 0.027 |
| P12814 | ACTN1 | 0.58 | 0.002 |
| P63000 | RAC1 | 0.59 | 0.002 |
| Q9BZV1 | UBXN6 | 0.59 | 0.001 |
| P48436 | SOX9 | 0.59 | 0.014 |
| P51948 | MAT1 | 0.59 | 0.038 |
| P37235 | HPCL1 | 0.59 | 0.026 |
| Q13541 | 4EBP1 | 0.59 | 0.049 |
| O75663 | TIPRL | 0.59 | 0.010 |
| P07099 | HYEP | 0.59 | 0.005 |
| Q9NW68 | BSDC1 | 0.59 | 0.002 |
| Q8TCD5 | NT5C | 0.59 | 0.003 |
| Q9NWT1 | PK1IP | 0.60 | 0.047 |
| Q96G21 | IMP4 | 0.60 | 0.009 |
| Q13247 | SRSF6 | 0.60 | 0.017 |
| Q13885 | TBB2A | 0.60 | 0.003 |
| Q04206 | TF65 | 0.60 | 0.006 |
| Q9NPA0 | EMC7 | 0.60 | 0.004 |
| Q9BSL1 | UBAC1 | 0.60 | 0.019 |
| Q7Z2W9 | RM21 | 0.61 | 0.001 |
| Q9NZN8 | CNOT2 | 0.61 | 0.043 |
| Q15738 | NSDHL | 0.61 | 0.002 |
| Q8IY17 | PLPL6 | 0.61 | 0.017 |
| Q15942 | ZYX | 0.61 | 0.000 |
| P07203 | GPX1 | 0.61 | 0.017 |
| P60520 | GBRL2 | 0.61 | 0.002 |
| P13797 | PLST | 0.62 | 0.009 |
| P57723 | PCBP4 | 0.62 | 0.009 |
| Q9Y617 | SERC | 0.63 | 0.001 |
| Q9H0L4 | CSTFT | 0.63 | 0.010 |
| P52566 | GDIR2 | 0.63 | 0.005 |
| Q14814 | MEF2D | 0.63 | 0.043 |
| Q9Y5M8 | SRPRB | 0.63 | 0.000 |
| Q9Y547 | IFT25 | 0.63 | 0.011 |
| Q9H488 | OFUT1 | 0.63 | 0.004 |
| Q15392 | DHC24 | 0.63 | 0.012 |
| Q9NRP0 | OSTC | 0.63 | 0.018 |
| Q96DE0 | NUD16 | 0.64 | 0.008 |
| Q9UET6 | TRM7 | 0.64 | 0.016 |
| Q86YS7 | C2CD5 | 0.64 | 0.041 |
| Q6P3W7 | SCYL2 | 0.64 | 0.030 |
| Q8NB37 | GALD1 | 0.64 | 0.011 |
| Q9NZM5 | NOP53 | 0.64 | 0.046 |
| P63167 | DYL1 | 0.64 | 0.034 |
| P51452 | DUS3 | 0.64 | 0.027 |
| P15954 | COX7C | 0.64 | 0.040 |
| P54619 | AAKG1 | 0.65 | 0.047 |
| P49458 | SRP09 | 0.65 | 0.002 |
| Q8NEB9 | PK3C3 | 0.65 | 0.036 |
| Q9Y6Y8 | S23IP | 0.65 | 0.004 |
| Q70CQ2 | UBP34 | 0.66 | 0.044 |
| Q6P1N0 | C2D1A | 0.66 | 0.031 |
| Q96SI9 | STRBP | 0.66 | 0.005 |
| Q9H7D7 | WDR26 | 0.66 | 0.021 |
| P41236 | IPP2 | 0.66 | 0.007 |
| Q6WKZ4 | RFIP1 | 0.66 | 0.019 |
| P62913 | RL11 | 0.67 | 0.007 |
| P46109 | CRKL | 0.67 | 0.018 |
| Q9NXJ5 | PGPI | 0.67 | 0.012 |
| P26006 | ITA3 | 0.68 | 0.024 |
| O94874 | UFL1 | 0.68 | 0.002 |
| Q13907 | IDI1 | 0.68 | 0.007 |
| Q8N5C6 | SRBD1 | 0.68 | 0.022 |
| Q9BYD1 | RM13 | 0.68 | 0.024 |
| Q99590 | SCAFB | 0.68 | 0.031 |
| Q9H2U2 | IPYR2 | 0.68 | 0.000 |
| P17676 | CEBPB | 0.68 | 0.035 |
| Q9UIJ7 | KAD3 | 0.68 | 0.007 |
| Q9P206 | K1522 | 0.69 | 0.011 |
| Q8N2K0 | ABD12 | 0.69 | 0.033 |
| O94915 | FRYL | 0.69 | 0.013 |
| Q86X55 | CARM1 | 0.69 | 0.002 |
| Q8IV08 | PLD3 | 0.69 | 0.041 |
| P30825 | CTR1 | 0.69 | 0.018 |
| P23229 | ITA6 | 0.69 | 0.029 |
| A0AV96 | RBM47 | 0.70 | 0.000 |
| Q9UJA5 | TRM6 | 0.70 | 0.005 |
| Q6ZRV2 | FA83H | 0.70 | 0.047 |
| Q58FF8 | H90B2 | 0.70 | 0.022 |
| P49902 | 5NTC | 0.70 | 0.006 |
| Q9BU61 | NDUF3 | 0.70 | 0.026 |
| Q9H773 | DCTP1 | 0.70 | 0.013 |
| O43395 | PRPF3 | 0.71 | 0.021 |
| O43809 | CPSF5 | 0.71 | 0.003 |
| Q9NZL9 | MAT2B | 0.71 | 0.018 |
| P14174 | MIF | 0.71 | 0.019 |
| O15213 | WDR46 | 0.71 | 0.015 |
| P46976 | GLYG | 0.71 | 0.035 |
| P84098 | RL19 | 0.71 | 0.001 |
| P62888 | RL30 | 0.71 | 0.022 |
| O43592 | XPOT | 0.71 | 0.008 |
| Q13148 | TADBP | 0.71 | 0.048 |
| P49720 | PSB3 | 0.72 | 0.013 |
| P19447 | ERCC3 | 0.72 | 0.050 |
| Q9Y6K9 | NEMO | 0.72 | 0.033 |
| Q14139 | UBE4A | 0.72 | 0.044 |
| P07108 | ACBP | 0.73 | 0.035 |
| O95478 | NSA2 | 0.73 | 0.006 |
| O00566 | MPP10 | 0.73 | 0.045 |
| O00148 | DX39A | 0.73 | 0.038 |
| Q96BH1 | RNF25 | 0.73 | 0.032 |
| P52298 | NCBP2 | 0.73 | 0.043 |
| Q02750 | MP2K1 | 0.73 | 0.002 |
| Q6GMV2 | SMYD5 | 0.73 | 0.044 |
| P48960 | CD97 | 0.74 | 0.042 |
| Q14197 | ICT1 | 0.74 | 0.049 |
| P46940 | IQGA1 | 0.74 | 0.001 |
| Q92974 | ARHG2 | 0.74 | 0.011 |
| Q06265 | EXOS9 | 0.74 | 0.049 |
| P11166 | GTR1 | 0.74 | 0.014 |
| Q9UBQ0 | VPS29 | 0.74 | 0.006 |
| Q14258 | TRI25 | 0.74 | 0.004 |
| O95825 | QORL1 | 0.75 | 0.028 |
| O15144 | ARPC2 | 0.75 | 0.013 |
| P09382 | LEG1 | 0.75 | 0.004 |
| P78406 | RAE1L | 0.75 | 0.010 |
| Q5JS54 | PSMG4 | 0.75 | 0.007 |
| Q9Y3Q8 | T22D4 | 0.75 | 0.030 |
| Q14376 | GALE | 0.75 | 0.029 |
| Q5VIR6 | VPS53 | 0.75 | 0.018 |
| Q12962 | TAF10 | 0.75 | 0.028 |
| P31483 | TIA1 | 0.75 | 0.038 |
| P18085 | ARF4 | 0.75 | 0.044 |
| P54132 | BLM | 0.75 | 0.007 |
| Q9NUL7 | DDX28 | 0.76 | 0.030 |
| P0DPD7 | EFMT4 | 0.76 | 0.047 |
| Q8TC12 | RDH11 | 0.76 | 0.006 |
| P32969 | RL9 | 0.76 | 0.006 |
| Q92934 | BAD | 0.76 | 0.008 |
| Q9Y2V2 | CHSP1 | 0.76 | 0.031 |
| P62829 | RL23 | 0.76 | 0.007 |
| O75494 | SRS10 | 0.77 | 0.012 |
| Q9H3P7 | GCP60 | 1.30 | 0.007 |
| Q9NZ01 | TECR | 1.30 | 0.013 |
| P15374 | UCHL3 | 1.30 | 0.002 |
| P01111 | RASN | 1.30 | 0.023 |
| P49419 | AL7A1 | 1.30 | 0.000 |
| Q8N1G0 | ZN687 | 1.30 | 0.015 |
| Q5JPE7 | NOMO2 | 1.30 | 0.004 |
| P11387 | TOP1 | 1.30 | 0.009 |
| P61619 | S61A1 | 1.30 | 0.036 |
| Q8NFF5 | FAD1 | 1.30 | 0.005 |
| Q96EY1 | DNJA3 | 1.30 | 0.015 |
| P30043 | BLVRB | 1.30 | 0.000 |
| Q9Y2I8 | WDR37 | 1.30 | 0.001 |
| Q00653 | NFKB2 | 1.30 | 0.033 |
| O00273 | DFFA | 1.30 | 0.008 |
| Q14562 | DHX8 | 1.30 | 0.021 |
| O75439 | MPPB | 1.31 | 0.005 |
| Q8IZ83 | A16A1 | 1.31 | 0.049 |
| P30740 | ILEU | 1.31 | 0.005 |
| O43399 | TPD54 | 1.31 | 0.002 |
| O94973 | AP2A2 | 1.31 | 0.000 |
| P53794 | SC5A3 | 1.31 | 0.004 |
| Q9Y4B6 | DCAF1 | 1.31 | 0.032 |
| P62861 | RS30 | 1.31 | 0.042 |
| Q9H4M9 | EHD1 | 1.31 | 0.004 |
| P27824 | CALX | 1.32 | 0.001 |
| Q5SSJ5 | HP1B3 | 1.32 | 0.006 |
| Q14573 | ITPR3 | 1.32 | 0.006 |
| P06865 | HEXA | 1.32 | 0.006 |
| Q9NRX1 | PNO1 | 1.32 | 0.033 |
| Q96EP5 | DAZP1 | 1.32 | 0.010 |
| O75694 | NU155 | 1.32 | 0.011 |
| P36954 | RPB9 | 1.32 | 0.022 |
| O15382 | BCAT2 | 1.32 | 0.014 |
| O14828 | SCAM3 | 1.32 | 0.014 |
| Q92769 | HDAC2 | 1.32 | 0.005 |
| Q9NPD3 | EXOS4 | 1.32 | 0.038 |
| Q16836 | HCDH | 1.32 | 0.019 |
| Q13724 | MOGS | 1.32 | 0.004 |
| Q6IQ49 | SDE2 | 1.32 | 0.037 |
| O43237 | DC1L2 | 1.32 | 0.013 |
| A6NIH7 | U119B | 1.32 | 0.025 |
| Q9Y3B9 | RRP15 | 1.32 | 0.020 |
| O60568 | PLOD3 | 1.32 | 0.000 |
| P26641 | EF1G | 1.32 | 0.001 |
| P49736 | MCM2 | 1.32 | 0.001 |
| P11182 | ODB2 | 1.32 | 0.003 |
| P63172 | DYLT1 | 1.32 | 0.005 |
| Q8NI36 | WDR36 | 1.32 | 0.000 |
| Q9Y305 | ACOT9 | 1.33 | 0.038 |
| Q09028 | RBBP4 | 1.33 | 0.004 |
| P36957 | ODO2 | 1.33 | 0.042 |
| P62942 | FKB1A | 1.33 | 0.001 |
| Q96FZ2 | HMCES | 1.33 | 0.015 |
| Q8IWA4 | MFN1 | 1.33 | 0.043 |
| P50416 | CPT1A | 1.33 | 0.003 |
| P34896 | GLYC | 1.34 | 0.003 |
| P61513 | RL37A | 1.34 | 0.016 |
| Q8NAV1 | PR38A | 1.34 | 0.003 |
| Q08378 | GOGA3 | 1.34 | 0.005 |
| P82930 | RT34 | 1.34 | 0.002 |
| P27361 | MK03 | 1.34 | 0.018 |
| Q9BZG8 | DPH1 | 1.34 | 0.009 |
| P07355 | ANXA2 | 1.34 | 0.007 |
| Q86U86 | PB1 | 1.34 | 0.011 |
| O00629 | IMA3 | 1.34 | 0.005 |
| O94906 | PRP6 | 1.34 | 0.000 |
| P18859 | ATP5J | 1.34 | 0.012 |
| Q9Y3I0 | RTCB | 1.35 | 0.001 |
| Q9H2G2 | SLK | 1.35 | 0.007 |
| P0DP25 | CALM3 | 1.35 | 0.017 |
| Q96ST2 | IWS1 | 1.35 | 0.003 |
| Q9Y613 | FHOD1 | 1.35 | 0.007 |
| O43252 | PAPS1 | 1.35 | 0.005 |
| P62701 | RS4X | 1.35 | 0.012 |
| P40222 | TXLNA | 1.35 | 0.017 |
| Q9NV31 | IMP3 | 1.35 | 0.034 |
| P17931 | LEG3 | 1.35 | 0.015 |
| Q969V3 | NCLN | 1.35 | 0.029 |
| Q14008 | CKAP5 | 1.35 | 0.001 |
| Q13043 | STK4 | 1.35 | 0.010 |
| Q9BRP1 | PDD2L | 1.35 | 0.040 |
| Q9UKY7 | CDV3 | 1.36 | 0.005 |
| Q02809 | PLOD1 | 1.36 | 0.006 |
| Q6KC79 | NIPBL | 1.36 | 0.033 |
| O43172 | PRP4 | 1.36 | 0.031 |
| Q9NZM1 | MYOF | 1.36 | 0.001 |
| O95671 | ASML | 1.36 | 0.001 |
| Q16775 | GLO2 | 1.36 | 0.046 |
| Q9HB65 | ELL3 | 1.36 | 0.048 |
| P30520 | PURA2 | 1.36 | 0.028 |
| Q9P2J5 | SYLC | 1.36 | 0.002 |
| Q9H993 | ARMT1 | 1.36 | 0.014 |
| Q15382 | RHEB | 1.36 | 0.029 |
| O14907 | TX1B3 | 1.36 | 0.020 |
| O60610 | DIAP1 | 1.37 | 0.015 |
| O00193 | SMAP | 1.37 | 0.034 |
| Q9BSH4 | TACO1 | 1.37 | 0.038 |
| Q8WTT2 | NOC3L | 1.37 | 0.020 |
| Q13617 | CUL2 | 1.37 | 0.006 |
| Q9Y2S7 | PDIP2 | 1.37 | 0.032 |
| Q9P2E9 | RRBP1 | 1.37 | 0.001 |
| Q9NPH2 | INO1 | 1.37 | 0.009 |
| Q8WXX5 | DNJC9 | 1.37 | 0.035 |
| Q53GQ0 | DHB12 | 1.37 | 0.001 |
| Q07960 | RHG01 | 1.37 | 0.014 |
| Q9P2R7 | SUCB1 | 1.37 | 0.019 |
| O75367 | H2AY | 1.38 | 0.013 |
| Q9BQA1 | MEP50 | 1.38 | 0.001 |
| Q96EB6 | SIR1 | 1.38 | 0.002 |
| Q8WYA6 | CTBL1 | 1.38 | 0.004 |
| Q14320 | FA50A | 1.38 | 0.044 |
| Q7Z2W4 | ZCCHV | 1.38 | 0.008 |
| Q9UG63 | ABCF2 | 1.38 | 0.001 |
| Q8IYB7 | DI3L2 | 1.38 | 0.001 |
| Q9Y2T2 | AP3M1 | 1.38 | 0.016 |
| P19623 | SPEE | 1.38 | 0.006 |
| Q9Y2Z4 | SYYM | 1.38 | 0.016 |
| P33992 | MCM5 | 1.39 | 0.005 |
| O75351 | VPS4B | 1.39 | 0.004 |
| Q7Z5H4 | VN1R5 | 1.39 | 0.034 |
| Q9H9B1 | EHMT1 | 1.39 | 0.004 |
| Q7L2J0 | MEPCE | 1.39 | 0.018 |
| Q86XI2 | CNDG2 | 1.39 | 0.010 |
| Q96RQ3 | MCCA | 1.39 | 0.010 |
| Q9HD33 | RM47 | 1.39 | 0.016 |
| Q9Y3Z3 | SAMH1 | 1.39 | 0.003 |
| Q14CX7 | NAA25 | 1.39 | 0.029 |
| O00244 | ATOX1 | 1.39 | 0.000 |
| P23588 | IF4B | 1.39 | 0.021 |
| Q8NFH4 | NUP37 | 1.39 | 0.024 |
| O43709 | BUD23 | 1.39 | 0.028 |
| P49721 | PSB2 | 1.39 | 0.003 |
| Q9UM54 | MYO6 | 1.39 | 0.003 |
| P52732 | KIF11 | 1.40 | 0.047 |
| Q70UQ0 | IKIP | 1.40 | 0.043 |
| O00743 | PPP6 | 1.40 | 0.012 |
| P50613 | CDK7 | 1.40 | 0.004 |
| O75323 | NIPS2 | 1.40 | 0.011 |
| Q96EU6 | RRP36 | 1.40 | 0.046 |
| Q9HCC0 | MCCB | 1.40 | 0.001 |
| A6NDG6 | PGP | 1.40 | 0.018 |
| P08579 | RU2B | 1.40 | 0.006 |
| O95793 | STAU1 | 1.40 | 0.004 |
| Q9P0J7 | KCMF1 | 1.41 | 0.005 |
| Q99497 | PARK7 | 1.41 | 0.000 |
| Q9Y3C6 | PPIL1 | 1.41 | 0.008 |
| Q96CU9 | FXRD1 | 1.41 | 0.008 |
| Q8IVM0 | CCD50 | 1.41 | 0.044 |
| Q96HE7 | ERO1A | 1.41 | 0.002 |
| P31040 | SDHA | 1.41 | 0.003 |
| O75947 | ATP5H | 1.42 | 0.009 |
| Q14997 | PSME4 | 1.42 | 0.004 |
| P08243 | ASNS | 1.42 | 0.001 |
| Q7Z4H3 | HDDC2 | 1.42 | 0.044 |
| Q96S52 | PIGS | 1.42 | 0.001 |
| P06737 | PYGL | 1.42 | 0.010 |
| P50453 | SPB9 | 1.42 | 0.001 |
| O60869 | EDF1 | 1.42 | 0.006 |
| Q5SWX8 | ODR4 | 1.42 | 0.033 |
| Q99729 | ROAA | 1.42 | 0.045 |
| Q5W111 | SPRY7 | 1.43 | 0.003 |
| Q9UBF2 | COPG2 | 1.43 | 0.016 |
| Q9BYC5 | FUT8 | 1.43 | 0.049 |
| Q5VTR2 | BRE1A | 1.43 | 0.002 |
| Q02818 | NUCB1 | 1.43 | 0.006 |
| A0A0U1RRL7 | MMPOS | 1.43 | 0.048 |
| Q86W42 | THOC6 | 1.43 | 0.006 |
| Q96CS3 | FAF2 | 1.43 | 0.037 |
| Q15785 | TOM34 | 1.43 | 0.000 |
| P61106 | RAB14 | 1.43 | 0.020 |
| Q86UU0 | BCL9L | 1.44 | 0.006 |
| Q6EEV6 | SUMO4 | 1.44 | 0.018 |
| Q13642 | FHL1 | 1.44 | 0.030 |
| P11177 | ODPB | 1.44 | 0.028 |
| P16403 | H12 | 1.44 | 0.024 |
| Q8NCN5 | PDPR | 1.44 | 0.034 |
| Q7L9L4 | MOB1B | 1.44 | 0.028 |
| Q5VV42 | CDKAL | 1.44 | 0.029 |
| Q9BZ67 | FRMD8 | 1.44 | 0.001 |
| P16455 | MGMT | 1.44 | 0.008 |
| Q8N4Q1 | MIA40 | 1.44 | 0.011 |
| P09211 | GSTP1 | 1.44 | 0.001 |
| Q15102 | PA1B3 | 1.45 | 0.001 |
| Q9H910 | JUPI2 | 1.45 | 0.002 |
| P26358 | DNMT1 | 1.45 | 0.003 |
| O95391 | SLU7 | 1.45 | 0.019 |
| O96000 | NDUBA | 1.45 | 0.024 |
| Q9BRJ2 | RM45 | 1.45 | 0.003 |
| Q96PU8 | QKI | 1.45 | 0.004 |
| Q9NX55 | HYPK | 1.45 | 0.003 |
| Q9NZD2 | GLTP | 1.45 | 0.024 |
| Q9BXJ9 | NAA15 | 1.45 | 0.003 |
| A6NDU8 | CE051 | 1.45 | 0.034 |
| Q14061 | COX17 | 1.45 | 0.024 |
| Q96BN8 | OTUL | 1.45 | 0.036 |
| Q9Y324 | FCF1 | 1.45 | 0.011 |
| O00186 | STXB3 | 1.46 | 0.016 |
| Q9NR46 | SHLB2 | 1.46 | 0.025 |
| P15586 | GNS | 1.46 | 0.001 |
| Q8NEJ9 | NGDN | 1.46 | 0.008 |
| Q96HN2 | SAHH3 | 1.46 | 0.010 |
| P57076 | CF298 | 1.46 | 0.046 |
| O60942 | MCE1 | 1.46 | 0.037 |
| Q14669 | TRIPC | 1.46 | 0.004 |
| P42765 | THIM | 1.46 | 0.002 |
| Q8N884 | CGAS | 1.46 | 0.001 |
| P23381 | SYWC | 1.46 | 0.001 |
| P0DMV9 | HS71B | 1.46 | 0.001 |
| Q9ULU4 | PKCB1 | 1.46 | 0.036 |
| Q9BPX5 | ARP5L | 1.47 | 0.042 |
| Q9BT73 | PSMG3 | 1.47 | 0.027 |
| Q9GZT4 | SRR | 1.47 | 0.042 |
| Q96AB3 | ISOC2 | 1.47 | 0.033 |
| P67870 | CSK2B | 1.47 | 0.033 |
| Q8WWV3 | RT4I1 | 1.47 | 0.008 |
| P47985 | UCRI | 1.47 | 0.025 |
| Q9UK76 | JUPI1 | 1.47 | 0.007 |
| Q3LXA3 | TKFC | 1.47 | 0.000 |
| Q9HB71 | CYBP | 1.47 | 0.004 |
| Q96DF8 | ESS2 | 1.47 | 0.025 |
| P47895 | AL1A3 | 1.47 | 0.000 |
| P08240 | SRPRA | 1.47 | 0.014 |
| P63208 | SKP1 | 1.47 | 0.020 |
| Q03154 | ACY1 | 1.47 | 0.000 |
| Q9Y6E2 | BZW2 | 1.48 | 0.006 |
| Q13472 | TOP3A | 1.48 | 0.047 |
| Q9Y4A5 | TRRAP | 1.48 | 0.015 |
| Q9NQS7 | INCE | 1.48 | 0.019 |
| O95202 | LETM1 | 1.48 | 0.011 |
| P50542 | PEX5 | 1.48 | 0.039 |
| P13798 | ACPH | 1.48 | 0.001 |
| Q9HCK8 | CHD8 | 1.48 | 0.013 |
| Q86VI3 | IQGA3 | 1.48 | 0.008 |
| P17655 | CAN2 | 1.48 | 0.000 |
| Q9P016 | THYN1 | 1.49 | 0.027 |
| Q9BTZ2 | DHRS4 | 1.49 | 0.000 |
| P35580 | MYH10 | 1.49 | 0.005 |
| Q3SXM5 | HSDL1 | 1.49 | 0.043 |
| O94905 | ERLN2 | 1.49 | 0.001 |
| P49748 | ACADV | 1.49 | 0.003 |
| P62070 | RRAS2 | 1.49 | 0.000 |
| O75348 | VATG1 | 1.49 | 0.048 |
| Q9UBX3 | DIC | 1.49 | 0.005 |
| P63272 | SPT4H | 1.50 | 0.032 |
| Q6PJG6 | BRAT1 | 1.50 | 0.044 |
| Q13586 | STIM1 | 1.50 | 0.003 |
| Q96DV4 | RM38 | 1.50 | 0.009 |
| P30049 | ATPD | 1.50 | 0.036 |
| O94916 | NFAT5 | 1.50 | 0.001 |
| P33993 | MCM7 | 1.50 | 0.000 |
| Q9Y2X7 | GIT1 | 1.50 | 0.010 |
| P48553 | TPC10 | 1.50 | 0.003 |
| Q9UI12 | VATH | 1.50 | 0.004 |
| Q7Z7K6 | CENPV | 1.50 | 0.025 |
| P22830 | HEMH | 1.50 | 0.016 |
| Q9H9T3 | ELP3 | 1.50 | 0.012 |
| Q14318 | FKBP8 | 1.50 | 0.003 |
| Q9Y2I1 | NISCH | 1.50 | 0.023 |
| P10253 | LYAG | 1.50 | 0.003 |
| Q9BRP8 | PYM1 | 1.50 | 0.002 |
| Q9NX18 | SDHF2 | 1.50 | 0.027 |
| Q8TB61 | S35B2 | 1.51 | 0.007 |
| P35244 | RFA3 | 1.51 | 0.002 |
| Q96K76 | UBP47 | 1.51 | 0.022 |
| P04844 | RPN2 | 1.51 | 0.017 |
| P33991 | MCM4 | 1.51 | 0.000 |
| P40926 | MDHM | 1.51 | 0.034 |
| P41227 | NAA10 | 1.51 | 0.006 |
| Q15813 | TBCE | 1.51 | 0.014 |
| Q8NBQ5 | DHB11 | 1.51 | 0.006 |
| Q08623 | HDHD1 | 1.51 | 0.024 |
| Q86Y37 | CACL1 | 1.51 | 0.004 |
| Q9UIW2 | PLXA1 | 1.51 | 0.022 |
| O14646 | CHD1 | 1.52 | 0.015 |
| O75818 | RPP40 | 1.52 | 0.013 |
| Q00403 | TF2B | 1.52 | 0.004 |
| Q16595 | FRDA | 1.52 | 0.003 |
| Q96GA7 | SDSL | 1.52 | 0.001 |
| Q9H0U3 | MAGT1 | 1.52 | 0.050 |
| P25440 | BRD2 | 1.52 | 0.010 |
| Q2TAY7 | SMU1 | 1.52 | 0.007 |
| Q9H9A5 | CNO10 | 1.52 | 0.025 |
| Q14181 | DPOA2 | 1.52 | 0.012 |
| Q9Y6V7 | DDX49 | 1.53 | 0.000 |
| Q96IX5 | ATPMD | 1.53 | 0.005 |
| Q969S9 | RRF2M | 1.53 | 0.013 |
| P32322 | P5CR1 | 1.54 | 0.009 |
| Q9H4L7 | SMRCD | 1.54 | 0.003 |
| Q86TB9 | PATL1 | 1.54 | 0.002 |
| Q75N03 | HAKAI | 1.54 | 0.019 |
| O75962 | TRIO | 1.54 | 0.023 |
| Q68E01 | INT3 | 1.54 | 0.003 |
| Q92890 | UFD1 | 1.54 | 0.003 |
| Q9NR31 | SAR1A | 1.55 | 0.002 |
| Q8NBT2 | SPC24 | 1.55 | 0.014 |
| P53007 | TXTP | 1.55 | 0.002 |
| P30260 | CDC27 | 1.55 | 0.008 |
| P46013 | KI67 | 1.55 | 0.001 |
| Q13393 | PLD1 | 1.55 | 0.044 |
| Q9HCU5 | PREB | 1.55 | 0.003 |
| P10321 | HLAC | 1.56 | 0.015 |
| Q14012 | KCC1A | 1.56 | 0.011 |
| O14662 | STX16 | 1.56 | 0.016 |
| Q7LBC6 | KDM3B | 1.56 | 0.002 |
| P35080 | PROF2 | 1.56 | 0.005 |
| Q2M389 | WASC4 | 1.56 | 0.009 |
| P16401 | H15 | 1.56 | 0.002 |
| Q7L1T6 | NB5R4 | 1.57 | 0.021 |
| Q15836 | VAMP3 | 1.57 | 0.006 |
| Q9H479 | FN3K | 1.57 | 0.011 |
| O15121 | DEGS1 | 1.57 | 0.012 |
| Q5TAQ9 | DCAF8 | 1.57 | 0.019 |
| P42696 | RBM34 | 1.57 | 0.024 |
| P24534 | EF1B | 1.57 | 0.010 |
| O14672 | ADA10 | 1.57 | 0.021 |
| Q9NY93 | DDX56 | 1.57 | 0.000 |
| Q9BVL4 | SELO | 1.57 | 0.049 |
| P34059 | GALNS | 1.57 | 0.042 |
| P35270 | SPRE | 1.58 | 0.041 |
| Q9H0U6 | RM18 | 1.58 | 0.002 |
| Q96EC8 | YIPF6 | 1.58 | 0.023 |
| Q9NZJ7 | MTCH1 | 1.58 | 0.001 |
| P68402 | PA1B2 | 1.58 | 0.011 |
| Q4U2R6 | RM51 | 1.58 | 0.011 |
| Q9Y4F5 | C170B | 1.59 | 0.014 |
| Q99719 | Septin-5 | 1.59 | 0.041 |
| Q96FZ7 | CHMP6 | 1.59 | 0.037 |
| Q5JTZ9 | SYAM | 1.59 | 0.005 |
| Q9NRF8 | PYRG2 | 1.59 | 0.042 |
| P16989 | YBOX3 | 1.59 | 0.000 |
| Q9UBR2 | CATZ | 1.60 | 0.022 |
| Q13643 | FHL3 | 1.60 | 0.024 |
| Q9H0S4 | DDX47 | 1.60 | 0.012 |
| Q9BUK6 | MSTO1 | 1.60 | 0.001 |
| Q9UBB4 | ATX10 | 1.61 | 0.020 |
| P51649 | SSDH | 1.61 | 0.030 |
| Q96PC5 | MIA2 | 1.61 | 0.006 |
| P52292 | IMA1 | 1.61 | 0.000 |
| P35250 | RFC2 | 1.61 | 0.018 |
| Q9BVK6 | TMED9 | 1.61 | 0.022 |
| Q9NVZ3 | NECP2 | 1.61 | 0.019 |
| P13693 | TCTP | 1.61 | 0.001 |
| Q9NZ32 | ARP10 | 1.61 | 0.008 |
| A4D1P6 | WDR91 | 1.61 | 0.047 |
| Q13206 | DDX10 | 1.62 | 0.041 |
| Q9UI30 | TR112 | 1.62 | 0.031 |
| P60604 | UB2G2 | 1.62 | 0.000 |
| Q9NRV9 | HEBP1 | 1.62 | 0.002 |
| Q5U5Q3 | MEX3C | 1.62 | 0.017 |
| Q9P260 | RELCH | 1.63 | 0.001 |
| O00411 | RPOM | 1.63 | 0.007 |
| P36915 | GNL1 | 1.63 | 0.010 |
| Q9H446 | RWDD1 | 1.63 | 0.006 |
| Q96GD4 | AURKB | 1.63 | 0.019 |
| O43708 | MAAI | 1.63 | 0.018 |
| Q9UHD9 | UBQL2 | 1.63 | 0.003 |
| P52434 | RPAB3 | 1.63 | 0.028 |
| Q13185 | CBX3 | 1.64 | 0.004 |
| Q96CP2 | FWCH2 | 1.64 | 0.002 |
| Q8IXQ5 | KLHL7 | 1.64 | 0.006 |
| Q9GZZ1 | NAA50 | 1.64 | 0.008 |
| O95140 | MFN2 | 1.64 | 0.028 |
| Q5TC12 | ATPF1 | 1.64 | 0.033 |
| P12931 | SRC | 1.64 | 0.022 |
| Q12834 | CDC20 | 1.64 | 0.015 |
| Q7L1Q6 | BZW1 | 1.64 | 0.007 |
| Q9Y2L5 | TPPC8 | 1.64 | 0.014 |
| Q9H0F6 | SHRPN | 1.64 | 0.034 |
| P09104 | ENOG | 1.64 | 0.038 |
| Q07065 | CKAP4 | 1.65 | 0.001 |
| Q16540 | RM23 | 1.65 | 0.030 |
| Q9Y3A2 | UTP11 | 1.65 | 0.046 |
| P82933 | RT09 | 1.65 | 0.008 |
| P35249 | RFC4 | 1.65 | 0.001 |
| Q9BQ61 | TRIR | 1.65 | 0.017 |
| Q5VW32 | BROX | 1.65 | 0.007 |
| Q8IYD1 | ERF3B | 1.65 | 0.043 |
| Q9BV86 | NTM1A | 1.66 | 0.006 |
| O94822 | LTN1 | 1.66 | 0.011 |
| Q06124 | PTN11 | 1.66 | 0.003 |
| P60983 | GMFB | 1.66 | 0.013 |
| Q10570 | CPSF1 | 1.66 | 0.003 |
| O75964 | ATP5L | 1.66 | 0.008 |
| Q9GZT6 | CC90B | 1.67 | 0.042 |
| Q13011 | ECH1 | 1.67 | 0.001 |
| Q86U44 | MTA70 | 1.67 | 0.009 |
| O75794 | CD123 | 1.67 | 0.000 |
| Q04837 | SSBP | 1.67 | 0.002 |
| Q15398 | DLGP5 | 1.67 | 0.042 |
| Q9HC36 | MRM3 | 1.68 | 0.004 |
| P36639 | 8ODP | 1.68 | 0.027 |
| Q9BY42 | RTF2 | 1.68 | 0.000 |
| P10155 | RO60 | 1.68 | 0.043 |
| Q8NFJ5 | RAI3 | 1.69 | 0.001 |
| P60228 | EIF3E | 1.69 | 0.002 |
| Q9BTE6 | AASD1 | 1.69 | 0.024 |
| O95801 | TTC4 | 1.69 | 0.015 |
| Q9BV19 | CA050 | 1.69 | 0.008 |
| P60763 | RAC3 | 1.69 | 0.004 |
| P36873 | PP1G | 1.69 | 0.021 |
| Q8WVX9 | FACR1 | 1.69 | 0.047 |
| Q92905 | CSN5 | 1.69 | 0.043 |
| O94766 | B3GA3 | 1.69 | 0.004 |
| Q13610 | PWP1 | 1.70 | 0.021 |
| Q8IVD9 | NUDC3 | 1.70 | 0.003 |
| Q9UBB6 | NCDN | 1.70 | 0.000 |
| Q5VWQ8 | DAB2P | 1.70 | 0.009 |
| Q13573 | SNW1 | 1.70 | 0.009 |
| P27986 | P85A | 1.70 | 0.016 |
| O14981 | BTAF1 | 1.70 | 0.024 |
| Q96FJ2 | DYL2 | 1.71 | 0.004 |
| P49366 | DHYS | 1.71 | 0.001 |
| P52630 | STAT2 | 1.71 | 0.049 |
| Q96FQ6 | S10AG | 1.72 | 0.013 |
| Q96GG9 | DCNL1 | 1.72 | 0.023 |
| Q9Y399 | RT02 | 1.72 | 0.006 |
| Q5VTE6 | ANGE2 | 1.72 | 0.008 |
| P41567 | EIF1 | 1.72 | 0.030 |
| Q86U38 | NOP9 | 1.72 | 0.004 |
| O75122 | CLAP2 | 1.72 | 0.038 |
| P35914 | HMGCL | 1.73 | 0.008 |
| Q14289 | FAK2 | 1.73 | 0.015 |
| Q9NRY2 | SOSSC | 1.73 | 0.004 |
| Q7L2H7 | EIF3M | 1.73 | 0.029 |
| O00767 | ACOD | 1.73 | 0.024 |
| O95155 | UBE4B | 1.73 | 0.010 |
| P09496 | CLCA | 1.74 | 0.006 |
| Q14165 | MLEC | 1.75 | 0.019 |
| P12532 | KCRU | 1.75 | 0.002 |
| P49840 | GSK3A | 1.75 | 0.019 |
| Q14019 | COTL1 | 1.75 | 0.030 |
| P23258 | TBG1 | 1.75 | 0.003 |
| Q709C8 | VP13C | 1.75 | 0.020 |
| Q9ULX9 | MAFF | 1.75 | 0.027 |
| Q9BQC3 | DPH2 | 1.75 | 0.005 |
| Q16763 | UBE2S | 1.76 | 0.012 |
| Q6IMN6 | CAPR2 | 1.76 | 0.003 |
| Q9HD45 | TM9S3 | 1.76 | 0.012 |
| Q969Y2 | GTPB3 | 1.76 | 0.017 |
| P35611 | ADDA | 1.77 | 0.002 |
| Q13613 | MTMR1 | 1.77 | 0.030 |
| Q9UJX3 | APC7 | 1.77 | 0.001 |
| Q9BSJ2 | GCP2 | 1.78 | 0.008 |
| P55039 | DRG2 | 1.78 | 0.006 |
| Q7L2E3 | DHX30 | 1.78 | 0.010 |
| Q4G176 | ACSF3 | 1.78 | 0.000 |
| P67812 | SC11A | 1.78 | 0.008 |
| Q9Y3B2 | EXOS1 | 1.78 | 0.008 |
| Q9NX40 | OCAD1 | 1.78 | 0.000 |
| P67775 | PP2AA | 1.79 | 0.027 |
| P49069 | CAMLG | 1.79 | 0.032 |
| P40937 | RFC5 | 1.79 | 0.009 |
| P29034 | S10A2 | 1.80 | 0.001 |
| Q96KB5 | TOPK | 1.80 | 0.012 |
| Q9Y5J9 | TIM8B | 1.80 | 0.023 |
| Q9HAN9 | NMNA1 | 1.80 | 0.032 |
| O14976 | GAK | 1.81 | 0.003 |
| Q96RE7 | NACC1 | 1.81 | 0.046 |
| Q9BQP7 | MGME1 | 1.82 | 0.033 |
| Q9BVG9 | PTSS2 | 1.82 | 0.043 |
| Q6SPF0 | SAMD1 | 1.82 | 0.047 |
| P23610 | HAP40 | 1.83 | 0.046 |
| O94966 | UBP19 | 1.83 | 0.023 |
| Q9Y241 | HIG1A | 1.83 | 0.005 |
| Q92990 | GLMN | 1.83 | 0.000 |
| Q3KQU3 | MA7D1 | 1.84 | 0.000 |
| Q9Y314 | NOSIP | 1.84 | 0.025 |
| P0CG12 | DERPC | 1.84 | 0.011 |
| Q5TA45 | INT11 | 1.84 | 0.002 |
| O43424 | GRID2 | 1.85 | 0.016 |
| P05166 | PCCB | 1.85 | 0.034 |
| Q9NX46 | ARHL2 | 1.86 | 0.017 |
| Q9Y2Z9 | COQ6 | 1.87 | 0.037 |
| Q49AR2 | CE022 | 1.87 | 0.010 |
| Q9BZX2 | UCK2 | 1.87 | 0.033 |
| P07858 | CATB | 1.88 | 0.003 |
| O95197 | RTN3 | 1.88 | 0.009 |
| Q9H7E9 | CH033 | 1.88 | 0.000 |
| O15160 | RPAC1 | 1.88 | 0.001 |
| P62899 | RL31 | 1.88 | 0.006 |
| O95372 | LYPA2 | 1.88 | 0.005 |
| Q99541 | PLIN2 | 1.89 | 0.000 |
| Q9ULE6 | PALD | 1.89 | 0.002 |
| P09488 | GSTM1 | 1.89 | 0.001 |
| Q3B7T1 | EDRF1 | 1.89 | 0.039 |
| P31321 | KAP1 | 1.90 | 0.007 |
| P04818 | TYSY | 1.90 | 0.000 |
| Q3B726 | RPA43 | 1.90 | 0.034 |
| Q02252 | MMSA | 1.90 | 0.029 |
| Q9H074 | PAIP1 | 1.90 | 0.000 |
| P41214 | EIF2D | 1.90 | 0.023 |
| O15554 | KCNN4 | 1.90 | 0.017 |
| Q00169 | PIPNA | 1.91 | 0.002 |
| O95834 | EMAL2 | 1.91 | 0.003 |
| P11388 | TOP2A | 1.91 | 0.000 |
| P08648 | ITA5 | 1.92 | 0.039 |
| Q9UNN8 | EPCR | 1.92 | 0.021 |
| Q9Y446 | PKP3 | 1.92 | 0.029 |
| Q13325 | IFIT5 | 1.93 | 0.013 |
| Q9NPQ8 | RIC8A | 1.93 | 0.002 |
| Q8WWC4 | MAIP1 | 1.93 | 0.017 |
| Q96TC7 | RMD3 | 1.94 | 0.002 |
| P31350 | RIR2 | 1.94 | 0.000 |
| O60443 | GSDME | 1.95 | 0.005 |
| P00846 | ATP6 | 1.95 | 0.005 |
| O76075 | DFFB | 1.95 | 0.006 |
| Q53EL6 | PDCD4 | 1.96 | 0.000 |
| Q8IXJ6 | SIR2 | 1.97 | 0.009 |
| Q9BVA0 | KTNB1 | 1.97 | 0.011 |
| Q9NVP2 | ASF1B | 1.97 | 0.048 |
| Q96HQ2 | C2AIL | 1.97 | 0.004 |
| O43156 | TTI1 | 1.98 | 0.001 |
| P07205 | PGK2 | 1.98 | 0.006 |
| O14773 | TPP1 | 1.98 | 0.000 |
| Q9C0B1 | FTO | 1.99 | 0.007 |
| Q14146 | URB2 | 1.99 | 0.004 |
| Q9UEY8 | ADDG | 1.99 | 0.007 |
| Q96LD4 | TRI47 | 2.00 | 0.001 |
| P10619 | PPGB | 2.01 | 0.003 |
| Q9BUR4 | TCAB1 | 2.02 | 0.004 |
| O15127 | SCAM2 | 2.02 | 0.031 |
| Q16626 | MEA1 | 2.03 | 0.003 |
| Q6P1M0 | S27A4 | 2.03 | 0.008 |
| Q8WTS1 | ABHD5 | 2.04 | 0.002 |
| P43307 | SSRA | 2.04 | 0.046 |
| P25815 | S100P | 2.04 | 0.050 |
| Q14353 | GAMT | 2.05 | 0.005 |
| Q9Y2P8 | RCL1 | 2.05 | 0.033 |
| Q03164 | KMT2A | 2.05 | 0.036 |
| O60888 | CUTA | 2.05 | 0.009 |
| Q9Y426 | C2CD2 | 2.05 | 0.020 |
| Q6P1Q9 | MET2B | 2.05 | 0.009 |
| P22033 | MUTA | 2.06 | 0.011 |
| P39210 | MPV17 | 2.06 | 0.007 |
| O75843 | AP1G2 | 2.06 | 0.038 |
| Q9UJ68 | MSRA | 2.08 | 0.014 |
| Q99575 | POP1 | 2.09 | 0.016 |
| Q9Y6H1 | CHCH2 | 2.09 | 0.000 |
| Q9BW27 | NUP85 | 2.09 | 0.000 |
| O75170 | PP6R2 | 2.10 | 0.004 |
| Q7Z422 | SZRD1 | 2.10 | 0.000 |
| P04183 | KITH | 2.10 | 0.009 |
| P28799 | GRN | 2.11 | 0.006 |
| Q8NCE2 | MTMRE | 2.11 | 0.038 |
| Q9HC07 | TM165 | 2.12 | 0.024 |
| Q9H1K1 | ISCU | 2.13 | 0.009 |
| Q9Y2H0 | DLGP4 | 2.13 | 0.023 |
| Q16850 | CP51A | 2.13 | 0.008 |
| Q9H201 | EPN3 | 2.14 | 0.039 |
| Q5JSZ5 | PRC2B | 2.14 | 0.045 |
| Q9H7B2 | RPF2 | 2.15 | 0.003 |
| Q9UHJ6 | SHPK | 2.15 | 0.019 |
| P50750 | CDK9 | 2.15 | 0.017 |
| Q9Y2S6 | TMA7 | 2.16 | 0.004 |
| Q9NZ43 | USE1 | 2.16 | 0.000 |
| P09497 | CLCB | 2.17 | 0.000 |
| Q03135 | CAV1 | 2.18 | 0.034 |
| O95456 | PSMG1 | 2.19 | 0.015 |
| Q15043 | S39AE | 2.19 | 0.025 |
| Q8N5K1 | CISD2 | 2.19 | 0.050 |
| Q9BT67 | NFIP1 | 2.20 | 0.004 |
| Q13685 | AAMP | 2.21 | 0.002 |
| Q9BTX1 | NDC1 | 2.21 | 0.002 |
| P35610 | SOAT1 | 2.21 | 0.001 |
| Q5PRF9 | SMAG2 | 2.21 | 0.014 |
| Q96SU4 | OSBL9 | 2.22 | 0.003 |
| O43294 | TGFI1 | 2.22 | 0.022 |
| Q96IR7 | HPDL | 2.22 | 0.012 |
| Q9P000 | COMD9 | 2.23 | 0.003 |
| P03886 | NU1M | 2.23 | 0.044 |
| Q9NXV2 | KCTD5 | 2.24 | 0.048 |
| P11117 | PPAL | 2.25 | 0.005 |
| Q7L7V1 | DHX32 | 2.26 | 0.009 |
| Q9NV56 | MRGBP | 2.26 | 0.001 |
| Q9BRG1 | VPS25 | 2.26 | 0.004 |
| Q8N6N3 | CA052 | 2.27 | 0.001 |
| Q01167 | FOXK2 | 2.27 | 0.013 |
| P38432 | COIL | 2.28 | 0.016 |
| P13498 | CY24A | 2.28 | 0.008 |
| Q15742 | NAB2 | 2.28 | 0.003 |
| Q9BZG1 | RAB34 | 2.28 | 0.001 |
| Q96PU4 | UHRF2 | 2.30 | 0.001 |
| Q5T200 | ZC3HD | 2.30 | 0.015 |
| Q15773 | MLF2 | 2.32 | 0.001 |
| Q9UMR2 | DD19B | 2.33 | 0.002 |
| O15397 | IPO8 | 2.33 | 0.008 |
| Q9BRT8 | CBWD1 | 2.34 | 0.008 |
| P82673 | RT35 | 2.35 | 0.001 |
| Q13505 | MTX1 | 2.37 | 0.010 |
| Q7Z7A3 | CTU1 | 2.44 | 0.041 |
| Q9NSI6 | BRWD1 | 2.44 | 0.004 |
| Q9H467 | CUED2 | 2.45 | 0.017 |
| Q96EI5 | TCAL4 | 2.45 | 0.006 |
| Q9NQW6 | ANLN | 2.48 | 0.015 |
| Q9H4M3 | FBX44 | 2.51 | 0.021 |
| Q92968 | PEX13 | 2.61 | 0.044 |
| Q9NVH2 | INT7 | 2.62 | 0.033 |
| Q9H1D9 | RPC6 | 2.63 | 0.022 |
| O75330 | HMMR | 2.65 | 0.003 |
| Q14527 | HLTF | 2.66 | 0.044 |
| P14635 | CCNB1 | 2.67 | 0.001 |
| Q9BTA9 | WAC | 2.67 | 0.009 |
| Q9Y587 | AP4S1 | 2.69 | 0.007 |
| P42858 | HD | 2.72 | 0.010 |
| Q9GZT8 | NIF3L | 2.72 | 0.001 |
| Q03518 | TAP1 | 2.73 | 0.005 |
| O00255 | MEN1 | 2.77 | 0.001 |
| Q8IWT6 | LRC8A | 2.79 | 0.009 |
| O95235 | KI20A | 2.80 | 0.024 |
| Q9Y3E7 | CHMP3 | 2.82 | 0.008 |
| Q9Y237 | PIN4 | 2.85 | 0.001 |
| Q9H8G2 | CAAP1 | 2.89 | 0.001 |
| P01011 | AACT | 3.06 | 0.000 |
| Q9BW19 | KIFC1 | 3.08 | 0.005 |
| Q8TAF3 | WDR48 | 3.12 | 0.019 |
| P05161 | ISG15 | 3.13 | 0.001 |
| Q9UKV5 | AMFR | 3.16 | 0.000 |
| Q92759 | TF2H4 | 3.20 | 0.008 |
| Q5TDH0 | DDI2 | 3.31 | 0.006 |
| Q9H6V9 | LDAH | 3.32 | 0.001 |
| P61962 | DCAF7 | 3.34 | 0.006 |
| Q0VGL1 | LTOR4 | 3.34 | 0.038 |
| Q9NVI1 | FANCI | 3.36 | 0.002 |
| Q9NXR1 | NDE1 | 3.39 | 0.023 |
| Q9Y672 | ALG6 | 3.42 | 0.027 |
| Q641Q2 | WAC2A | 3.43 | 0.010 |
| Q9NZN4 | EHD2 | 3.44 | 0.011 |
| Q15345 | LRC41 | 3.44 | 0.001 |
| P35221 | CTNA1 | 3.47 | 0.021 |
| O00762 | UBE2C | 3.52 | 0.001 |
| Q9Y244 | POMP | 3.57 | 0.037 |
| Q9BQ70 | TCF25 | 3.66 | 0.005 |
| Q9Y666 | S12A7 | 3.80 | 0.001 |
| Q06136 | KDSR | 3.80 | 0.000 |
| Q96T88 | UHRF1 | 3.89 | 0.001 |
| O15460 | P4HA2 | 3.91 | 0.039 |
| Q9NVT9 | ARMC1 | 3.93 | 0.004 |
| Q9Y2G5 | OFUT2 | 4.20 | 0.002 |
| P13521 | SCG2 | 4.31 | 0.001 |
| Q96HR9 | REEP6 | 4.35 | 0.040 |
| Q13535 | ATR | 4.38 | 0.002 |
| Q92520 | FAM3C | 4.60 | 0.000 |
| Q06481 | APLP2 | 4.93 | 0.007 |
| Q9NRZ9 | HELLS | 4.96 | 0.004 |
| P49137 | MAPK2 | 5.46 | 0.024 |
| P33552 | CKS2 | 6.65 | 0.000 |
| P06703 | S10A6 | 7.19 | 0.000 |
| Q6WCQ1 | MPRIP | 14.69 | 0.001 |

Additional file 1: Table S5: The CO-IP proteins of TMEM43 in MIAPaCa-2 cells using Mass Spectrometry proteomics analysis

| Protein IDs | Peptide counts | Sequence coverage [%] | Score |
| --- | --- | --- | --- |
| sp\|Q9BQG0\|MBB1A_HUMAN | 3 | 2.4 | 21.225 |
| sp\|Q00341\|VIGLN_HUMAN | 3 | 2.8 | 21.677 |
| sp\|P09874\|PARP1_HUMAN | 3 | 3.4 | 17.05 |
| sp\|Q15020\|SART3_HUMAN | 3 | 3.7 | 20.81 |
| sp\|Q01804\|OTUD4_HUMAN | 3 | 3.9 | 18.964 |
| sp\|P11216\|PYGB_HUMAN | 3 | 5 | 22.645 |
| sp\|Q08J23\|NSUN2_HUMAN | 3 | 5.5 | 19.593 |
| sp\|P78362\|SRPK2_HUMAN | 3 | 6.2 | 16.596 |
| sp\|O15234\|CASC3_HUMAN | 3 | 6.4 | 20.288 |
| sp\|Q71RC2\|LARP4_HUMAN | 3 | 6.5 | 21.211 |
| sp\|Q9UBU9\|NXF1_HUMAN | 3 | 6.5 | 18.829 |
| sp\|Q96T37\|RBM15_HUMAN | 3 | 6.8 | 17.352 |
| sp\|O15371\|EIF3D_HUMAN | 3 | 6.9 | 20.78 |
| sp\|Q6UN15\|FIP1_HUMAN | 3 | 8.2 | 20.445 |
| sp\|Q52LJ0\|FA98B_HUMAN | 3 | 8.3 | 19.726 |
| sp\|P08621\|RU17_HUMAN | 3 | 8.5 | 22.236 |
| sp\|Q9NUD5\|ZCHC3_HUMAN | 3 | 8.7 | 19.575 |
| sp\|Q9BY44\|EIF2A_HUMAN | 3 | 8.9 | 19.984 |
| sp\|Q9UKM9\|RALY_HUMAN | 3 | 10.1 | 17.925 |
| sp\|Q8ND56\|LS14A_HUMAN | 3 | 10.2 | 17.611 |
| sp\|Q99873\|ANM1_human | 3 | 10.2 | 19.596 |
| sp\|P82650\|RT22_HUMAN | 3 | 10.3 | 18.881 |
| sp\|Q02878\|RL6_HUMAN | 3 | 10.8 | 19.324 |
| sp\|Q9Y295\|DRG1_HUMAN | 3 | 11.4 | 22.243 |
| sp\|P00558\|PGK1_HUMAN | 3 | 12 | 18.176 |
| sp\|Q13595\|TRA2A_HUMAN | 3 | 12.1 | 17.562 |
| sp\|O00303\|EIF3F_HUMAN | 3 | 12.3 | 25.871 |
| sp\|O43251\|RFOX2_HUMAN | 3 | 13.1 | 20.906 |
| sp\|Q06830\|PRDX1_HUMAN | 3 | 15.1 | 18.798 |
| sp\|P06730\|IF4E_HUMAN | 3 | 15.2 | 20.279 |
| sp\|P48047\|ATPO_HUMAN | 3 | 16.9 | 18.933 |
| sp\|Q86U42\|PABP2_HUMAN | 3 | 18 | 24.363 |
| sp\|P84103\|SRSF3_HUMAN | 3 | 18.3 | 22.284 |
| sp\|P62273\|RS29_HUMAN | 1 | 19.6 | 10.032 |
| sp\|Q86V81\|THOC4_HUMAN | 3 | 21.4 | 25.695 |
| sp\|P60866\|RS20_HUMAN | 3 | 23.5 | 35.191 |
| sp\|P61353\|RL27_HUMAN | 3 | 23.5 | 18.255 |
| sp\|P62851\|RS25_HUMAN | 3 | 24 | 19.239 |
| sp\|P62937\|PPIA_HUMAN | 3 | 24.2 | 24.729 |
| sp\|Q5JNZ5\|RS26L_HUMAN | 3 | 24.3 | 19.857 |
| sp\|P53680\|AP2S1_HUMAN | 3 | 24.6 | 21.405 |
| sp\|P62847\|RS24_HUMAN | 3 | 26.3 | 22.242 |
| sp\|Q99878\|H2A1J_HUMAN | 3 | 30.5 | 18.203 |
| sp\|P46776\|RL27A_HUMAN | 3 | 31.1 | 23.582 |
| sp\|P62316\|SMD2_HUMAN | 3 | 32.2 | 18.072 |
| sp\|O43395\|PRPF3_HUMAN | 3 | 35.3 | 132.56 |
| sp\|P37108\|SRP14_HUMAN | 3 | 41.2 | 19.408 |
| sp\|Q9BTV4\|TMEM43_HUMAN | 4 | 56.6 | 100.36 |
| sp\|Q9UL18\|AGO1_HUMAN | 4 | 5.6 | 13.275 |
| sp\|P27816\|MAP4_HUMAN | 4 | 6.2 | 30.147 |
| sp\|Q9BQ39\|DDX50_HUMAN | 4 | 6.9 | 21.262 |
| sp\|P17066\|HSP76_HUMAN | 4 | 7.2 | 13.715 |
| sp\|P55884\|EIF3B_HUMAN | 4 | 7.2 | 23.901 |
| sp\|P0DMV8\|HS71A_HUMAN | 4 | 8 | 6.2148 |
| sp\|Q9NUL3\|STAU2_HUMAN | 4 | 8.6 | 25.312 |
| sp\|Q96CW1\|AP2M1_HUMAN | 4 | 9 | 24.435 |
| sp\|Q9BZE4\|NOG1_HUMAN | 4 | 9 | 24.932 |
| sp\|Q14498\|RBM39_HUMAN | 4 | 10 | 31.272 |
| sp\|O43148\|MCES_HUMAN | 4 | 10.3 | 28.575 |
| sp\|Q13283\|G3BP1_HUMAN | 4 | 11.8 | 28.316 |
| sp\|Q96GA3\|LTV1_HUMAN | 4 | 11.8 | 37.13 |
| sp\|P61978\|HNRPK_HUMAN | 4 | 11.9 | 26.062 |
| sp\|Q07955\|SRSF1_HUMAN | 4 | 12.9 | 24.981 |
| sp\|P46777\|RL5_HUMAN | 4 | 13.1 | 26.896 |
| sp\|Q9UNQ2\|DIM1_HUMAN | 4 | 13.1 | 22.907 |
| sp\|P06733\|ENOA_HUMAN | 4 | 15.9 | 26.227 |
| sp\|Q16629\|SRSF7_HUMAN | 4 | 16 | 29.574 |
| sp\|P22087\|FBRL_HUMAN | 4 | 17.4 | 25.191 |
| sp\|O75569\|PRKRA_HUMAN | 4 | 18.2 | 25.654 |
| sp\|P60174\|TPIS_HUMAN | 4 | 20.3 | 25.357 |
| sp\|P05198\|IF2A_HUMAN | 4 | 22.2 | 29.232 |
| sp\|P83731\|RL24_HUMAN | 4 | 22.3 | 23.309 |
| sp\|P42766\|RL35_HUMAN | 4 | 22.8 | 50.779 |
| sp\|P08865\|RSSA_HUMAN | 4 | 23.1 | 26.958 |
| sp\|P46783\|RS10_HUMAN | 4 | 23.6 | 27.112 |
| sp\|P04406\|G3P_HUMAN | 4 | 24.2 | 31.317 |
| sp\|P62269\|RS18_HUMAN | 4 | 28.3 | 26.616 |
| sp\|P30050\|RL12_HUMAN | 4 | 33.9 | 25.687 |
| sp\|P23528\|COF1_HUMAN | 4 | 38.6 | 39.849 |
| sp\|P42677\|RS27_HUMAN | 4 | 40.5 | 29.359 |
| sp\|P62805\|H4_HUMAN | 4 | 43.7 | 26.44 |
| sp\|O43432\|IF4G3_HUMAN | 5 | 3.7 | 18.162 |
| sp\|O15027\|SC16A_HUMAN | 5 | 4.6 | 29.671 |
| sp\|O43143\|DHX15_HUMAN | 5 | 7.3 | 32.882 |
| sp\|Q9UKV8\|AGO2_HUMAN | 5 | 7.6 | 33.162 |
| sp\|P13639\|EF2_HUMAN | 5 | 7.9 | 33.616 |
| sp\|Q14444\|CAPR1_HUMAN | 5 | 8.5 | 35.224 |
| sp\|Q9BRZ2\|TRI56_HUMAN | 5 | 10.5 | 40.384 |
| sp\|P15311\|EZRI_HUMAN | 5 | 11.4 | 33.692 |
| sp\|Q8WXF1\|PSPC1_HUMAN | 5 | 12.8 | 23.638 |
| sp\|P07437\|TBB5_HUMAN | 5 | 13.5 | 6.2377 |
| sp\|O14979\|HNRDL_HUMAN | 5 | 13.6 | 32.98 |
| sp\|P49411\|EFTU_HUMAN | 5 | 14.4 | 31.929 |
| sp\|P19474\|RO52_HUMAN | 5 | 14.9 | 32.913 |
| sp\|P38159\|RBMX_HUMAN | 5 | 15.1 | 31.962 |
| sp\|P55795\|HNRH2_HUMAN | 5 | 15.6 | 15.157 |
| sp\|P52597\|HNRPF_HUMAN | 5 | 16.1 | 21.588 |
| sp\|Q9UMS4\|PRP19_HUMAN | 5 | 17.5 | 32.417 |
| sp\|Q9BY77\|PDIP3_HUMAN | 5 | 18.8 | 33.619 |
| sp\|P36542\|ATPG_HUMAN | 5 | 19.1 | 32.591 |
| sp\|P68104\|EF1A1_HUMAN | 5 | 19.9 | 60.989 |
| sp\|P20042\|IF2B_HUMAN | 5 | 20.4 | 34.675 |
| sp\|P62917\|RL8_HUMAN | 5 | 21.4 | 36.187 |
| sp\|P06748\|NPM_HUMAN | 5 | 22.1 | 42.336 |
| sp\|Q92734\|TFG_HUMAN | 5 | 24.8 | 39.077 |
| sp\|Q96AG4\|LRC59_HUMAN | 5 | 26.1 | 37.881 |
| sp\|O60814\|H2B1K_HUMAN | 5 | 26.2 | 32.477 |
| sp\|P62906\|RL10A_HUMAN | 5 | 26.3 | 37.641 |
| sp\|P18124\|RL7_HUMAN | 5 | 26.6 | 37.52 |
| sp\|Q14011\|CIRBP_HUMAN | 5 | 34.3 | 33.753 |
| sp\|Q3MHD2\|LSM12_HUMAN | 5 | 40.5 | 33.85 |
| sp\|P08708\|RS17_HUMAN | 5 | 48.9 | 46.331 |
| sp\|P25398\|RS12_HUMAN | 5 | 50.8 | 62.067 |
| sp\|P63173\|RL38_HUMAN | 5 | 52.9 | 32.846 |
| sp\|Q8NDV7\|TNR6A_HUMAN | 6 | 4.5 | 35.74 |
| sp\|Q9HAU5\|RENT2_HUMAN | 6 | 6.2 | 37.711 |
| sp\|Q9Y5A9\|YTHD2_HUMAN | 6 | 11.9 | 18.941 |
| sp\|Q01844\|EWS_HUMAN | 6 | 12.3 | 58.697 |
| sp\|P19525\|E2AK2_HUMAN | 6 | 13.2 | 50.029 |
| sp\|Q8N684\|CPSF7_HUMAN | 6 | 18.7 | 44.303 |
| sp\|Q92804\|RBP56_HUMAN | 6 | 19.1 | 33.023 |
| sp\|Q13895\|BYST_HUMAN | 6 | 19.2 | 40.259 |
| sp\|P62424\|RL7A_HUMAN | 6 | 19.9 | 37.442 |
| sp\|Q15366\|PCBP2_HUMAN | 6 | 25.2 | 38.238 |
| sp\|Q13242\|SRSF9_HUMAN | 6 | 25.3 | 35.149 |
| sp\|P31942\|HNRH3_HUMAN | 6 | 26.9 | 113.05 |
| sp\|P40429\|RL13A_HUMAN | 6 | 30 | 44.543 |
| sp\|Q13151\|ROA0_HUMAN | 6 | 31.1 | 59.458 |
| sp\|P62263\|RS14_HUMAN | 6 | 33.1 | 49.031 |
| sp\|Q96QR8\|PURB_HUMAN | 6 | 33.3 | 35.789 |
| sp\|P46782\|RS5_HUMAN | 6 | 36.8 | 43.774 |
| sp\|P63104\|1433Z_HUMAN | 6 | 38.4 | 37.052 |
| sp\|P35268\|RL22_HUMAN | 6 | 47.7 | 42.795 |
| sp\|P62266\|RS23_HUMAN | 6 | 49 | 36.921 |
| sp\|Q9Y262\|EIF3L_HUMAN | 7 | 12.4 | 43.388 |
| sp\|Q09161\|NCBP1_HUMAN | 7 | 13.4 | 47.476 |
| sp\|Q9P258\|RCC2_HUMAN | 7 | 15.1 | 43.489 |
| sp\|Q15424\|SAFB1_HUMAN | 7 | 15.3 | 49.73 |
| sp\|P10515\|ODP2_HUMAN | 7 | 16.2 | 61.921 |
| sp\|P05783\|K1C18_HUMAN | 7 | 17.4 | 32.718 |
| sp\|P25705\|ATPA_HUMAN | 7 | 18.6 | 55.019 |
| sp\|P10809\|CH60_HUMAN | 7 | 18.7 | 53.101 |
| sp\|Q8IUX4\|ABC3F_HUMAN | 7 | 21.7 | 32.581 |
| sp\|Q14240\|IF4A2_HUMAN | 7 | 22.1 | 35.707 |
| sp\|Q96AK3\|ABC3D_HUMAN | 7 | 22.3 | 17.591 |
| sp\|P68371\|TBB4B_HUMAN | 7 | 22.7 | 52.839 |
| sp\|P12236\|ADT3_HUMAN | 7 | 22.8 | 6.241 |
| sp\|Q9UN81\|LORF1_HUMAN | 7 | 22.8 | 45.319 |
| sp\|Q9UH17\|ABC3B_HUMAN | 7 | 24.3 | 48.91 |
| sp\|P41091\|IF2G_HUMAN | 7 | 24.8 | 47.25 |
| sp\|P62995\|TRA2B_HUMAN | 7 | 28.1 | 52.306 |
| sp\|P39019\|RS19_HUMAN | 7 | 37.9 | 44.621 |
| sp\|P61254\|RL26_HUMAN | 7 | 40 | 56.031 |
| sp\|P62244\|RS15A_HUMAN | 7 | 51.5 | 44.802 |
| sp\|Q14152\|EIF3A_HUMAN | 8 | 7 | 64.308 |
| sp\|Q99613\|EIF3C_HUMAN | 8 | 8.4 | 49.427 |
| sp\|Q9H0D6\|XRN2_HUMAN | 8 | 13.8 | 60.341 |
| sp\|Q92945\|FUBP2_HUMAN | 8 | 15.2 | 52.823 |
| sp\|P38646\|GRP75_HUMAN | 8 | 15.2 | 55.315 |
| sp\|O75534\|CSDE1_HUMAN | 8 | 15.3 | 50.005 |
| sp\|P11021\|BIP_HUMAN | 8 | 16.1 | 47.808 |
| sp\|P39023\|RL3_HUMAN | 8 | 21.6 | 58.163 |
| sp\|Q14103\|HNRPD_HUMAN | 8 | 23.7 | 54.264 |
| sp\|P36578\|RL4_HUMAN | 8 | 26.2 | 56.135 |
| sp\|P27635\|RL10_HUMAN | 8 | 27.1 | 57.513 |
| sp\|Q9Y3F4\|STRAP_HUMAN | 8 | 29.7 | 52.767 |
| sp\|P18621\|RL17_HUMAN | 8 | 34.8 | 69.078 |
| sp\|P05388\|RLA0_HUMAN | 8 | 36.3 | 52.529 |
| sp\|P63244\|RACK1_HUMAN | 8 | 38.5 | 58.877 |
| sp\|P62750\|RL23A_HUMAN | 8 | 45.5 | 66.482 |
| sp\|P35580\|MYH10_HUMAN | 9 | 4.9 | 16.482 |
| sp\|Q12797\|ASPH_HUMAN | 9 | 14.1 | 58.876 |
| sp\|Q9NYF8\|BCLF1_HUMAN | 9 | 14.1 | 59.908 |
| sp\|Q92615\|LAR4B_HUMAN | 9 | 16 | 64.452 |
| sp\|P07900\|HS90A_HUMAN | 9 | 17.5 | 23.065 |
| sp\|P35637\|FUS_HUMAN | 9 | 18.6 | 245.45 |
| sp\|P38919\|IF4A3_HUMAN | 9 | 22.4 | 57.131 |
| sp\|P07910\|HNRPC_HUMAN | 9 | 24.8 | 59.275 |
| sp\|Q96I24\|FUBP3_HUMAN | 9 | 26.7 | 64.417 |
| sp\|P16402\|H13_HUMAN | 9 | 27.6 | 9.0843 |
| sp\|P10412\|H14_HUMAN | 9 | 27.9 | 63.46 |
| sp\|P63261\|ACTG_HUMAN | 9 | 29.1 | 65.609 |
| sp\|P05141\|ADT2_HUMAN | 9 | 29.2 | 58.641 |
| sp\|P14618\|KPYM_HUMAN | 9 | 30.1 | 60.694 |
| sp\|P67809\|YBOX1_HUMAN | 9 | 41.4 | 28.947 |
| sp\|P62280\|RS11_HUMAN | 9 | 43.7 | 60.378 |
| sp\|P62249\|RS16_HUMAN | 9 | 45.2 | 81.665 |
| sp\|P62081\|RS7_HUMAN | 9 | 52.1 | 59.321 |
| sp\|Q00610\|CLH1_HUMAN | 10 | 8.8 | 64.85 |
| sp\|Q99700\|ATX2_HUMAN | 10 | 11.7 | 70.456 |
| sp\|Q14157\|UBP2L_HUMAN | 10 | 15.3 | 77.065 |
| sp\|P11940\|PABP1_HUMAN | 10 | 18.7 | 101.07 |
| sp\|P16989\|YBOX3_HUMAN | 10 | 32.8 | 201.95 |
| sp\|P31943\|HNRH1_HUMAN | 10 | 33.6 | 95.412 |
| sp\|P15880\|RS2_HUMAN | 10 | 40.3 | 82.398 |
| sp\|O95782\|AP2A1_HUMAN | 11 | 13.8 | 72.022 |
| sp\|O43390\|HNRPR_HUMAN | 11 | 19.7 | 50.664 |
| sp\|P11142\|HSP7C_HUMAN | 11 | 20.7 | 82.145 |
| sp\|P14866\|HNRPL_HUMAN | 11 | 22.6 | 79.877 |
| sp\|Q8NCA5\|FA98A_HUMAN | 11 | 27.6 | 168.14 |
| sp\|Q9NUQ6\|SPS2L_HUMAN | 11 | 27.6 | 81.636 |
| sp\|P68363\|TBA1B_HUMAN | 11 | 33.9 | 9.869 |
| sp\|Q9BQE3\|TBA1C_HUMAN | 11 | 34.1 | 151.68 |
| sp\|P62753\|RS6_HUMAN | 11 | 36.5 | 82.944 |
| sp\|P62241\|RS8_HUMAN | 11 | 54.3 | 89.075 |
| sp\|Q9NR30\|DDX21_HUMAN | 12 | 21.8 | 83.774 |
| sp\|Q5T9A4\|ATD3B_HUMAN | 12 | 22.2 | 87.021 |
| sp\|Q9NVI7\|ATD3A_HUMAN | 12 | 22.4 | 19.974 |
| sp\|Q9Y6M1\|IF2B2_HUMAN | 12 | 25 | 104.33 |
| sp\|Q12905\|ILF2_HUMAN | 12 | 37.7 | 80.181 |
| sp\|Q8NC51\|PAIRB_HUMAN | 12 | 39.7 | 78.856 |
| sp\|Q15717\|ELAV1_HUMAN | 12 | 43.9 | 92.462 |
| sp\|P46781\|RS9_HUMAN | 12 | 45.4 | 105.18 |
| sp\|P62277\|RS13_HUMAN | 12 | 55.6 | 105.79 |
| sp\|Q7Z739\|YTHD3_HUMAN | 13 | 19.7 | 38.798 |
| sp\|Q06787\|FMR1_HUMAN | 13 | 26.3 | 86.654 |
| sp\|Q07666\|KHDR1_HUMAN | 13 | 33.4 | 93.4 |
| sp\|Q9H2U1\|DHX36_HUMAN | 14 | 19.4 | 121.99 |
| sp\|P08238\|HS90B_HUMAN | 14 | 21 | 102.62 |
| sp\|P52272\|HNRPM_HUMAN | 14 | 22.5 | 123.18 |
| sp\|O43290\|SNUT1_HUMAN | 14 | 24.2 | 97.865 |
| sp\|P26599\|PTBP1_HUMAN | 14 | 36.2 | 208.47 |
| sp\|Q9UN86\|G3BP2_HUMAN | 14 | 39 | 174.86 |
| sp\|Q9HC16\|ABC3G_HUMAN | 14 | 44.5 | 113.21 |
| sp\|Q96HS1\|PGAM5_HUMAN | 14 | 48.8 | 96.237 |
| sp\|P23396\|RS3_HUMAN | 14 | 60.1 | 103.28 |
| sp\|P51991\|ROA3_HUMAN | 15 | 28 | 145.04 |
| sp\|P08670\|VIME_HUMAN | 15 | 37.6 | 98.706 |
| sp\|P09651\|ROA1_HUMAN | 15 | 41.7 | 102.86 |
| sp\|P61247\|RS3A_HUMAN | 15 | 46.2 | 111.15 |
| sp\|Q00839\|HNRPU_HUMAN | 16 | 19.3 | 158.66 |
| sp\|Q12906\|ILF3_HUMAN | 16 | 21.9 | 129.68 |
| sp\|P04259\|K2C6B_HUMAN | 16 | 22.7 | 19.644 |
| sp\|P43243\|MATR3_HUMAN | 16 | 24.4 | 156.16 |
| sp\|P26196\|DDX6_HUMAN | 16 | 40.6 | 123.19 |
| sp\|Q9Y224\|RTRAF_HUMAN | 16 | 72.1 | 119.45 |
| sp\|Q9BYK8\|HELZ2_HUMAN | 17 | 9.3 | 116.99 |
| sp\|Q04637\|IF4G1_HUMAN | 17 | 15.5 | 122.36 |
| sp\|Q9Y2W1\|TR150_HUMAN | 17 | 20.1 | 135.63 |
| sp\|P63010\|AP2B1_HUMAN | 17 | 20.7 | 111.57 |
| sp\|Q96PK6\|RBM14_HUMAN | 17 | 31.1 | 174 |
| sp\|Q9BYJ9\|YTHD1_HUMAN | 17 | 32.6 | 150.13 |
| sp\|Q9BUJ2\|HNRL1_HUMAN | 18 | 28.6 | 161.75 |
| sp\|Q15233\|NONO_HUMAN | 18 | 37.8 | 144.34 |
| sp\|Q9Y3I0\|RTCB_HUMAN | 18 | 39.6 | 156.64 |
| sp\|O00425\|IF2B3_HUMAN | 18 | 39.7 | 141.37 |
| sp\|P22626\|ROA2_HUMAN | 19 | 43.9 | 164.45 |
| sp\|P19338\|NUCL_HUMAN | 20 | 29.2 | 160.4 |
| sp\|Q7Z417\|NUFP2_HUMAN | 20 | 38.6 | 199.21 |
| sp\|Q92841\|DDX17_HUMAN | 21 | 30.7 | 95.958 |
| sp\|P51116\|FXR2_HUMAN | 21 | 46.5 | 210.25 |
| sp\|Q6PKG0\|LARP1_HUMAN | 22 | 24.8 | 167.49 |
| sp\|P23246\|SFPQ_HUMAN | 22 | 35.4 | 317.21 |
| sp\|O60506\|HNRPQ_HUMAN | 22 | 36.1 | 248.11 |
| sp\|Q9HCE1\|MOV10_HUMAN | 23 | 28.2 | 183.88 |
| sp\|P12956\|XRCC6_HUMAN | 23 | 41.7 | 165.92 |
| sp\|P13010\|XRCC5_HUMAN | 23 | 47.4 | 187.78 |
| sp\|Q9NZB2\|F120A_HUMAN | 24 | 31.8 | 187.92 |
| sp\|Q14694\|UBP10_HUMAN | 25 | 47.7 | 220.65 |
| sp\|Q8WWM7\|ATX2L_HUMAN | 26 | 28.1 | 239.55 |
| sp\|Q13310\|PABP4_HUMAN | 26 | 34 | 191.46 |
| sp\|P17844\|DDX5_HUMAN | 26 | 38.3 | 195.55 |
| sp\|P48634\|PRC2A_HUMAN | 28 | 20.9 | 206.6 |
| sp\|Q08211\|DHX9_HUMAN | 30 | 32.3 | 250.28 |
| sp\|P35579\|MYH9_HUMAN | 31 | 21.6 | 236.1 |
| sp\|P51114\|FXR1_HUMAN | 31 | 57.5 | 323.31 |
| sp\|O00571\|DDX3X_HUMAN | 31 | 57.7 | 323.31 |
| sp\|Q9Y520\|PRC2C_HUMAN | 37 | 16.4 | 287.58 |
| sp\|Q92900\|RENT1_HUMAN | 41 | 45.6 | 323.31 |

Additional file 1: Table S6: The database of TCGA

| Sample | TMEM43 levels | RAP2B levels | RFS | OS | OS.time |
| --- | --- | --- | --- | --- | --- |
| TCGA-2J-AAB9-01A | 26.6542547 | 8.7104268 | 521 | 1 | 627 |
| TCGA-2J-AABI-01A | 21.3525935 | 5.9146796 | 728 | 0 | 969 |
| TCGA-2J-AABP-01A | 23.6092222 | 10.4733939 | 463 | 0 | 463 |
| TCGA-2J-AABR-01A | 29.6964369 | 10.3240513 | 327 | 0 | 438 |
| TCGA-2J-AABT-01A | 46.1042783 | 7.9478957 | 319 | 0 | 319 |
| TCGA-2L-AAQJ-01A | 26.4378454 | 10.7719402 |  | 1 | 394 |
| TCGA-3A-A9IB-01A | 42.3985540 | 16.5096762 |  | 1 | 224 |
| TCGA-3A-A9IC-01A | 24.3810414 | 10.1611140 |  | 1 | 738 |
| TCGA-3A-A9IH-01A | 26.0193716 | 12.3857763 | 763 | 0 | 1021 |
| TCGA-3A-A9IL-01A | 15.9276283 | 8.3459012 | 2741 | 0 | 2741 |
| TCGA-3A-A9IN-01A | 18.2717787 | 3.2212679 | 2084 | 0 | 2084 |
| TCGA-3A-A9IR-01A | 10.7494183 | 0.7149310 | 1542 | 0 | 1542 |
| TCGA-3A-A9IV-01A | 10.8270603 | 3.6407681 | 967 | 0 | 1103 |
| TCGA-F2-A44G-01A | 34.8883774 | 10.2230379 | 214 | 1 | 233 |
| TCGA-FB-A4P5-01A | 32.5782651 | 7.7133533 | 4 | 1 | 179 |
| TCGA-FB-A545-01A | 32.6645357 | 13.9536274 | 378 | 1 | 732 |
| TCGA-FB-A78T-01A | 30.1768706 | 10.9901842 | 1 | 1 | 375 |
| TCGA-FB-AAPS-01A | 38.0479797 | 15.3096086 | 228 | 0 | 228 |
| TCGA-FB-AAPU-01A | 18.9577401 | 13.6777550 |  | 1 | 381 |
| TCGA-FB-AAQ2-01A | 64.5169046 | 8.5487635 | 92 | 1 | 153 |
| TCGA-FB-AAQ3-01A | 25.2049847 | 11.4012872 |  | 1 | 31 |
| TCGA-H6-8124-01A | 48.5476950 | 11.6075427 | 259 | 0 | 392 |
| TCGA-H6-A45N-01A | 28.9172585 | 9.5523795 | 363 | 1 | 397 |
| TCGA-HV-A5A4-01A | 27.4126339 | 9.9524919 | 232 | 0 | 232 |
| TCGA-HV-A5A5-01A | 24.4416170 | 10.1464705 | 289 | 0 | 289 |
| TCGA-HV-A5A6-01A | 36.5435654 | 10.9350211 | 1511 | 1 | 2036 |
| TCGA-HV-AA8X-01A | 17.3298900 | 8.7679181 | 375 | 1 | 532 |
| TCGA-HZ-7919-01A | 39.5122772 | 11.5670389 | 20 | 1 | 593 |
| TCGA-HZ-7922-01A | 34.2183735 | 11.8569778 | 4 | 0 | 4 |
| TCGA-HZ-7924-01A | 26.1358328 | 5.6477999 | 831 | 0 | 840 |
| TCGA-HZ-8003-01A | 26.8552776 | 9.8159762 | 21 | 1 | 596 |
| TCGA-HZ-8315-01A | 38.0839635 | 7.6684975 | 28 | 1 | 299 |
| TCGA-HZ-8317-01A | 26.9844492 | 6.5306270 | 16 | 1 | 378 |
| TCGA-HZ-8636-01A | 35.1488565 | 16.4484123 | 5 | 1 | 545 |
| TCGA-HZ-8637-01A | 21.7623164 | 10.6059535 | 0 | 1 | 517 |
| TCGA-HZ-8638-01A | 19.8649370 | 6.7247605 | 91 | 1 | 151 |
| TCGA-HZ-A49G-01A | 26.3295268 | 9.0746739 | 660 | 0 | 660 |
| TCGA-HZ-A49H-01A | 26.8552776 | 11.0628430 | 491 | 0 | 491 |
| TCGA-HZ-A77O-01A | 24.3213256 | 10.1352218 | 98 | 1 | 160 |
| TCGA-HZ-A77Q-01A | 34.4443872 | 8.9879014 | 33 | 0 | 33 |
| TCGA-IB-7644-01A | 30.6001404 | 10.1464705 | 306 | 1 | 394 |
| TCGA-IB-7645-01A | 37.8512723 | 11.3762643 | 1210 | 1 | 1502 |
| TCGA-IB-7649-01A | 30.7288607 | 8.9687415 | 282 | 1 | 467 |
| TCGA-IB-7651-01A | 40.1162740 | 7.8985339 | 492 | 1 | 603 |
| TCGA-IB-7652-01A | 36.5435654 | 10.8302711 | 724 | 0 | 1116 |
| TCGA-IB-7885-01A | 27.7459956 | 13.5307729 | 977 | 0 | 1257 |
| TCGA-IB-7887-01A | 26.2233448 | 13.5356335 |  | 1 | 110 |
| TCGA-IB-7888-01A | 33.6053346 | 7.8604209 | 427 | 1 | 1332 |
| TCGA-IB-7889-01A | 32.4409280 | 8.7358561 | 352 | 1 | 481 |
| TCGA-IB-7891-01A | 29.5627058 | 10.1962874 | 525 | 1 | 913 |
| TCGA-IB-7897-01A | 38.7254070 | 7.9970939 | 397 | 1 | 486 |
| TCGA-IB-8126-01A | 27.6872922 | 5.6664886 | 83 | 0 | 462 |
| TCGA-IB-A5SQ-01A | 27.1559715 | 11.9752962 | 197 | 1 | 219 |
| TCGA-IB-A5SS-01A | 40.7399980 | 9.5632693 | 66 | 1 | 460 |
| TCGA-IB-A5ST-01A | 30.3993127 | 9.0566671 | 8 | 0 | 635 |
| TCGA-IB-AAUN-01A | 41.9043953 | 9.6446814 | 131 | 1 | 144 |
| TCGA-IB-AAUO-01A | 29.9022121 | 12.8542246 | 211 | 1 | 239 |
| TCGA-IB-AAUS-01A | 29.7197776 | 11.0835486 | 225 | 0 | 225 |
| TCGA-IB-AAUW-01A | 31.6433263 | 6.0945007 | 185 | 1 | 230 |
| TCGA-L1-A7W4-01A | 32.8631351 | 14.6147566 | 129 | 1 | 278 |
| TCGA-LB-A7SX-01A | 20.8052192 | 8.0639399 | 127 | 1 | 393 |
| TCGA-LB-A8F3-01A | 14.6364702 | 3.5108772 | 379 | 0 | 379 |
| TCGA-LB-A9Q5-01A | 22.6267710 | 9.3451968 | 290 | 1 | 313 |
| TCGA-M8-A5N4-01A | 36.6544030 | 11.3182588 | 416 | 0 | 584 |
| TCGA-PZ-A5RE-01A | 29.0910039 | 9.0289877 | 393 | 1 | 470 |
| TCGA-Q3-AA2A-01A | 25.7916166 | 12.3043657 | 95 | 0 | 95 |
| TCGA-RB-A7B8-01A | 25.5516844 | 15.0851630 | 449 | 1 | 466 |
| TCGA-RL-AAAS-01A | 29.4475503 | 8.9248799 | 9 | 0 | 9 |
| TCGA-S4-A8RO-01A | 27.8255652 | 19.5666749 | 291 | 0 | 525 |
| TCGA-S4-A8RP-01A | 26.0674295 | 10.4241404 |  | 1 | 702 |
| TCGA-US-A774-01A | 25.3672163 | 8.3438879 | 366 | 1 | 695 |
| TCGA-US-A779-01A | 18.9577401 | 7.5689318 |  | 1 | 511 |
| TCGA-US-A77J-01A | 28.1885211 | 6.8608436 |  | 1 | 568 |
| TCGA-XD-AAUG-01A | 41.7613692 | 6.1621061 | 420 | 0 | 420 |
| TCGA-XD-AAUH-01A | 35.9455469 | 7.8070722 | 87 | 0 | 395 |
| TCGA-XD-AAUI-01A | 32.0612550 | 12.8198019 | 175 | 1 | 366 |
| TCGA-XN-A8T5-01A | 31.6692847 | 7.8785055 | 242 | 0 | 720 |
| TCGA-YH-A8SY-01A | 27.0798102 | 12.6081061 | 388 | 0 | 388 |
| TCGA-YY-A8LH-01A | 14.6687113 | 5.6152193 | 2016 | 0 | 2016 |
| TCGA-Z5-AAPL-01A | 21.3199813 | 8.3416149 | 467 | 0 | 467 |
| TCGA-2J-AAB1-01A | 29.4945777 | 7.5824944 |  | 1 | 66 |
| TCGA-2J-AAB4-01A | 24.6799237 | 11.6740245 | 729 | 0 | 729 |
| TCGA-2J-AAB6-01A | 34.3773095 | 24.1446443 | 169 | 1 | 293 |
| TCGA-2J-AAB8-01A | 29.6746696 | 8.5153267 | 80 | 0 | 80 |
| TCGA-2J-AABA-01A | 48.1526919 | 6.7930404 | 160 | 1 | 607 |
| TCGA-2J-AABE-01A | 30.7288607 | 7.7553800 | 676 | 0 | 676 |
| TCGA-2J-AABF-01A | 24.8633402 | 10.7080838 | 443 | 1 | 691 |
| TCGA-2J-AABH-01A | 27.7657367 | 12.4702656 | 1287 | 0 | 1287 |
| TCGA-2J-AABK-01A | 19.3431074 | 9.6176135 | 484 | 0 | 484 |
| TCGA-2J-AABO-01A | 25.8989520 | 12.1519383 | 439 | 0 | 440 |
| TCGA-2J-AABU-01A | 29.8553747 | 8.9901356 | 154 | 1 | 277 |
| TCGA-2J-AABV-01A | 13.2552465 | 4.7417243 | 126 | 1 | 652 |
| TCGA-2L-AAQA-01A | 28.7026214 | 16.8206215 |  | 1 | 143 |
| TCGA-2L-AAQE-01A | 28.8524741 | 8.3716337 |  | 1 | 684 |
| TCGA-2L-AAQI-01A | 20.3027795 | 11.7847874 |  | 1 | 103 |
| TCGA-2L-AAQL-01A | 20.8937276 | 12.0822728 |  | 1 | 292 |
| TCGA-2L-AAQM-01A | 20.3434500 | 3.8982760 | 914 | 0 | 1383 |
| TCGA-3A-A9I5-01A | 13.7010976 | 9.0867646 | 1794 | 0 | 1794 |
| TCGA-3A-A9I7-01A | 25.2727746 | 8.6378682 | 1309 | 0 | 1323 |
| TCGA-3A-A9I9-01A | 28.8740135 | 9.0940349 | 513 | 1 | 634 |
| TCGA-3A-A9IJ-01A | 8.8235966 | 2.0852178 | 1854 | 0 | 1854 |
| TCGA-3A-A9IO-01A | 7.1787517 | 3.1415317 | 1942 | 0 | 1942 |
| TCGA-3A-A9IS-01A | 11.6813947 | 3.5914731 | 998 | 0 | 998 |
| TCGA-3A-A9IU-01A | 28.0237325 | 13.0595543 | 371 | 1 | 458 |
| TCGA-3A-A9IX-01A | 38.3586063 | 9.4389686 | 901 | 0 | 1037 |
| TCGA-3A-A9IZ-01A | 28.3116895 | 11.2874119 | 109 | 1 | 308 |
| TCGA-3A-A9J0-01A | 39.6005362 | 10.1849930 | 363 | 0 | 743 |
| TCGA-3E-AAAY-01A | 24.3658649 | 8.1682487 | 987 | 0 | 2285 |
| TCGA-3E-AAAZ-01A | 36.7258038 | 7.2764010 | 1600 | 1 | 2182 |
| TCGA-F2-6879-01A | 40.4307357 | 8.2836690 | 183 | 1 | 334 |
| TCGA-F2-6880-01A | 14.6092317 | 2.8834684 | 295 | 0 | 295 |
| TCGA-F2-7273-01A | 44.5012411 | 9.3890055 | 256 | 1 | 592 |
| TCGA-F2-7276-01A | 37.0889918 | 7.8805182 |  | 1 | 216 |
| TCGA-F2-A44H-01A | 24.8001591 | 10.0579774 | 586 | 0 | 586 |
| TCGA-F2-A7TX-01A | 20.4742925 | 10.5847157 |  | 1 | 95 |
| TCGA-F2-A8YN-01A | 26.6542547 | 8.0055098 | 517 | 0 | 517 |
| TCGA-FB-A4P6-01A | 38.2025027 | 5.2281657 | 620 | 0 | 767 |
| TCGA-FB-A5VM-01A | 30.1995806 | 13.7010976 | 177 | 1 | 498 |
| TCGA-FB-A7DR-01A | 30.6247385 | 13.0181426 | 166 | 1 | 353 |
| TCGA-FB-AAPP-01A | 8.9857220 | 5.3542084 | 150 | 1 | 485 |
| TCGA-FB-AAPQ-01A | 16.9499158 | 13.2145055 |  | 1 | 1130 |
| TCGA-FB-AAPY-01A | 21.7300961 | 8.7358561 |  | 1 | 1059 |
| TCGA-FB-AAPZ-01A | 36.3314998 | 10.5566054 | 716 | 0 | 716 |
| TCGA-FB-AAQ0-01A | 27.3549832 | 10.3448384 |  | 1 | 473 |
| TCGA-FB-AAQ1-01A | 24.1160511 | 11.3289929 |  | 1 | 123 |
| TCGA-FB-AAQ6-01A | 16.7150416 | 6.7121484 | 213 | 1 | 244 |
| TCGA-H8-A6C1-01A | 27.1960662 | 11.8756785 | 455 | 0 | 671 |
| TCGA-HV-A5A3-01A | 38.6002204 | 11.7737389 |  | 1 | 128 |
| TCGA-HV-A7OL-01A | 17.1825729 | 9.2312645 | 123 | 0 | 252 |
| TCGA-HV-A7OP-01A | 13.5834328 | 2.5426937 | 342 | 0 | 978 |
| TCGA-HV-AA8V-01A | 32.2504376 | 9.9442675 | 526 | 0 | 920 |
| TCGA-HZ-7289-01A | 19.6995237 | 15.6291084 | 228 | 1 | 661 |
| TCGA-HZ-7918-01A | 31.0534571 | 12.5352801 | 542 | 0 | 969 |
| TCGA-HZ-7920-01A | 36.3623514 | 6.2917721 | 0 | 1 | 236 |
| TCGA-HZ-7923-01A | 35.8754627 | 8.5356999 | 8 | 0 | 314 |
| TCGA-HZ-7925-01A | 42.9573211 | 9.6313617 | 364 | 1 | 614 |
| TCGA-HZ-7926-01A | 36.2969565 | 11.6446538 | 340 | 1 | 518 |
| TCGA-HZ-8001-01A | 25.3349547 | 8.7726984 | 706 | 0 | 706 |
| TCGA-HZ-8002-01A | 42.0505052 | 8.0660530 | 24 | 1 | 366 |
| TCGA-HZ-8005-01A | 59.6293726 | 10.4273484 |  | 1 | 120 |
| TCGA-HZ-8519-01A | 33.1267931 | 7.1841008 | 454 | 0 | 454 |
| TCGA-HZ-A49I-01A | 35.8754627 | 9.4814041 | 0 | 1 | 308 |
| TCGA-HZ-A4BH-01A | 39.3416585 | 9.2646166 | 194 | 0 | 194 |
| TCGA-HZ-A4BK-01A | 23.4965757 | 10.0316661 | 657 | 0 | 657 |
| TCGA-HZ-A77P-01A | 37.8064296 | 6.9140838 | 330 | 0 | 330 |
| TCGA-HZ-A8P1-01A | 13.8940261 | 5.7049233 | 7 | 0 | 7 |
| TCGA-HZ-A9TJ-01A | 29.4699167 | 7.3657462 | 555 | 0 | 603 |
| TCGA-HZ-A9TJ-06A | 29.0479559 | 14.0641077 |  | 0 | 603 |
| TCGA-IB-7646-01A | 38.5608752 | 9.6824864 | 132 | 1 | 145 |
| TCGA-IB-7654-01A | 27.5077334 | 7.7271708 |  | 1 | 476 |
| TCGA-IB-7886-01A | 32.0612550 | 6.3863266 |  | 1 | 123 |
| TCGA-IB-7890-01A | 52.5838052 | 11.9029126 | 519 | 1 | 598 |
| TCGA-IB-7893-01A | 44.7707800 | 9.9662521 | 80 | 1 | 117 |
| TCGA-IB-8127-01A | 36.1536204 | 10.2053065 | 486 | 0 | 522 |
| TCGA-IB-A5SO-01A | 31.7476322 | 9.6473582 | 276 | 1 | 365 |
| TCGA-IB-A5SP-01A | 19.7479013 | 6.4765722 | 300 | 0 | 482 |
| TCGA-IB-A6UF-01A | 56.8765109 | 8.3241746 | 593 | 0 | 666 |
| TCGA-IB-A6UG-01A | 33.8484570 | 5.8762835 |  | 1 | 41 |
| TCGA-IB-A7LX-01A | 34.0605280 | 14.7668022 | 230 | 1 | 250 |
| TCGA-IB-A7M4-01A | 38.2025027 | 16.0982878 | 483 | 0 | 483 |
| TCGA-IB-AAUM-01A | 21.2307066 | 5.2234052 | 8 | 0 | 8 |
| TCGA-IB-AAUP-01A | 37.5427258 | 9.7968493 | 431 | 0 | 431 |
| TCGA-IB-AAUQ-01A | 38.0074810 | 8.8663154 | 107 | 1 | 183 |
| TCGA-IB-AAUR-01A | 30.3461278 | 9.1933907 | 318 | 0 | 338 |
| TCGA-IB-AAUT-01A | 26.2957248 | 9.9609435 | 287 | 0 | 287 |
| TCGA-IB-AAUU-01A | 28.9609578 | 11.2426153 | 245 | 0 | 245 |
| TCGA-IB-AAUV-01A | 41.4793892 | 12.4381853 | 404 | 0 | 404 |
| TCGA-OE-A75W-01A | 19.6340030 | 7.9826750 | 146 | 1 | 267 |
| TCGA-Q3-A5QY-01A | 22.5747563 | 10.4063509 | 416 | 0 | 416 |
| TCGA-RB-AA9M-01A | 26.4378454 | 8.2277882 | 258 | 0 | 286 |
| TCGA-S4-A8RM-01A | 21.3420308 | 9.1414610 | 737 | 0 | 737 |
| TCGA-US-A776-01A | 9.5523795 | 15.1634916 | 844 | 0 | 1216 |
| TCGA-US-A77E-01A | 33.6648446 | 7.9663234 | 166 | 1 | 430 |
| TCGA-US-A77G-01A | 27.1172469 | 10.8044266 |  | 1 | 12 |
| TCGA-XD-AAUL-01A | 36.1168875 | 8.9879014 | 498 | 0 | 498 |
| TCGA-XN-A8T3-01A | 36.2638892 | 10.8562639 | 461 | 0 | 951 |
| TCGA-YB-A89D-01A | 27.9647990 | 9.8432929 | 350 | 0 | 350 |

Additional file 1: Table S7: The database of GSE62452

| GSE62452 | TMEM43  levles | PRPF3 levles | RAP2B levles | Type |
| --- | --- | --- | --- | --- |
| GSM1527106 | 5.4874 | 5.92698 | 5.84493 | Normal |
| GSM1527108 | 6.42293 | 5.86149 | 6.52514 | Normal |
| GSM1527110 | 7.32409 | 6.11247 | 6.66902 | Normal |
| GSM1527112 | 6.83761 | 6.13117 | 6.25212 | Normal |
| GSM1527114 | 6.86136 | 6.06173 | 6.08812 | Normal |
| GSM1527116 | 7.44177 | 5.81261 | 6.73404 | Normal |
| GSM1527118 | 6.29857 | 5.78688 | 6.02069 | Normal |
| GSM1527120 | 7.5966 | 6.35864 | 6.51084 | Normal |
| GSM1527122 | 7.40165 | 6.09864 | 6.39549 | Normal |
| GSM1527124 | 7.25041 | 5.86263 | 6.43021 | Normal |
| GSM1527126 | 5.99973 | 5.86792 | 5.92492 | Normal |
| GSM1527128 | 7.12045 | 6.38794 | 6.77597 | Normal |
| GSM1527130 | 6.04802 | 5.8279 | 5.75482 | Normal |
| GSM1527132 | 6.28921 | 6.04693 | 6.44677 | Normal |
| GSM1527134 | 7.03508 | 5.8738 | 6.91107 | Normal |
| GSM1527136 | 5.61877 | 5.94566 | 5.73882 | Normal |
| GSM1527138 | 6.17885 | 6.31231 | 5.68471 | Normal |
| GSM1527140 | 6.32219 | 6.0397 | 5.89907 | Normal |
| GSM1527142 | 7.0615 | 6.16735 | 6.64097 | Normal |
| GSM1527144 | 7.04077 | 6.09754 | 6.2667 | Normal |
| GSM1527146 | 7.04875 | 6.11135 | 6.25197 | Normal |
| GSM1527148 | 7.27744 | 5.8279 | 6.428 | Normal |
| GSM1527150 | 5.47303 | 5.88979 | 5.68248 | Normal |
| GSM1527152 | 6.60675 | 6.02628 | 6.33872 | Normal |
| GSM1527154 | 6.21115 | 6.0292 | 6.10071 | Normal |
| GSM1527156 | 7.37882 | 6.07249 | 6.60899 | Normal |
| GSM1527158 | 6.07041 | 6.46315 | 5.43295 | Normal |
| GSM1527160 | 5.50012 | 5.58537 | 6.37816 | Normal |
| GSM1527162 | 6.23729 | 5.96009 | 5.65384 | Normal |
| GSM1527164 | 5.67733 | 5.93231 | 5.76874 | Normal |
| GSM1527166 | 7.19703 | 6.23857 | 7.00951 | Normal |
| GSM1527168 | 6.74136 | 5.99776 | 6.52689 | Normal |
| GSM1527170 | 6.45565 | 5.99717 | 5.99157 | Normal |
| GSM1527172 | 7.5247 | 6.02283 | 6.64867 | Normal |
| GSM1527174 | 6.8236 | 6.45589 | 6.34477 | Normal |
| GSM1527176 | 6.33914 | 6.11782 | 5.73205 | Normal |
| GSM1527178 | 6.9273 | 6.17613 | 6.14637 | Normal |
| GSM1527180 | 6.93177 | 5.95687 | 6.64481 | Normal |
| GSM1527182 | 6.85842 | 6.23245 | 6.63713 | Normal |
| GSM1527184 | 7.46667 | 6.1221 | 6.60064 | Normal |
| GSM1527186 | 6.06404 | 6.0822 | 5.84703 | Normal |
| GSM1527188 | 5.74636 | 6.08272 | 5.65756 | Normal |
| GSM1527190 | 6.55801 | 6.15112 | 6.30675 | Normal |
| GSM1527192 | 7.2569 | 5.9456 | 6.45687 | Normal |
| GSM1527194 | 6.77178 | 6.15011 | 6.65446 | Normal |
| GSM1527195 | 6.60503 | 6.61473 | 6.45966 | Normal |
| GSM1527197 | 6.89426 | 5.73516 | 6.89854 | Normal |
| GSM1527201 | 5.11817 | 5.25022 | 5.89784 | Normal |
| GSM1527203 | 7.21025 | 5.91961 | 7.12643 | Normal |
| GSM1527206 | 6.75198 | 5.90123 | 5.81 | Normal |
| GSM1527208 | 4.82398 | 5.64747 | 5.95177 | Normal |
| GSM1527211 | 6.49079 | 6.4553 | 6.5917 | Normal |
| GSM1527214 | 5.76529 | 5.98771 | 6.12953 | Normal |
| GSM1527217 | 5.97172 | 5.86603 | 6.04539 | Normal |
| GSM1527221 | 7.18535 | 6.09119 | 6.27133 | Normal |
| GSM1527222 | 6.67252 | 6.07999 | 6.28091 | Normal |
| GSM1527224 | 7.74338 | 5.93689 | 7.22064 | Normal |
| GSM1527226 | 5.66974 | 5.37849 | 6.17485 | Normal |
| GSM1527229 | 6.5479 | 6.15583 | 5.8099 | Normal |
| GSM1527231 | 8.07037 | 6.14924 | 6.26727 | Normal |
| GSM1527233 | 6.11382 | 6.08635 | 6.31984 | Normal |
| GSM1527105 | 7.38687 | 6.24805 | 6.50834 | Tumor |
| GSM1527107 | 7.14679 | 6.19484 | 6.83214 | Tumor |
| GSM1527109 | 6.94582 | 6.26972 | 6.788 | Tumor |
| GSM1527111 | 7.56255 | 6.09293 | 6.44994 | Tumor |
| GSM1527113 | 6.84152 | 6.02502 | 6.76797 | Tumor |
| GSM1527115 | 7.14174 | 5.97558 | 6.61201 | Tumor |
| GSM1527117 | 7.58538 | 6.03842 | 6.40325 | Tumor |
| GSM1527119 | 6.57782 | 6.32537 | 6.71348 | Tumor |
| GSM1527121 | 6.75172 | 6.20494 | 6.54829 | Tumor |
| GSM1527123 | 6.9548 | 6.1705 | 6.64976 | Tumor |
| GSM1527125 | 7.06006 | 6.33354 | 6.54542 | Tumor |
| GSM1527127 | 6.41485 | 6.17445 | 6.21237 | Tumor |
| GSM1527129 | 7.49421 | 6.05596 | 6.42446 | Tumor |
| GSM1527131 | 6.77964 | 6.75869 | 6.90808 | Tumor |
| GSM1527133 | 6.76852 | 6.08544 | 6.62655 | Tumor |
| GSM1527135 | 7.54764 | 5.97177 | 6.70587 | Tumor |
| GSM1527137 | 6.16806 | 6.01101 | 6.27547 | Tumor |
| GSM1527139 | 6.05945 | 6.00053 | 5.5207 | Tumor |
| GSM1527141 | 7.14963 | 6.12762 | 6.88476 | Tumor |
| GSM1527143 | 6.75537 | 6.32811 | 6.27336 | Tumor |
| GSM1527145 | 7.26522 | 6.36822 | 6.53609 | Tumor |
| GSM1527147 | 7.06753 | 6.218 | 6.62464 | Tumor |
| GSM1527149 | 7.16432 | 6.15439 | 6.70714 | Tumor |
| GSM1527151 | 6.92618 | 6.01974 | 6.51703 | Tumor |
| GSM1527153 | 7.36636 | 6.39819 | 7.36977 | Tumor |
| GSM1527155 | 7.30436 | 5.86909 | 6.62359 | Tumor |
| GSM1527157 | 7.24123 | 6.62782 | 6.12709 | Tumor |
| GSM1527159 | 7.46704 | 6.32653 | 6.59551 | Tumor |
| GSM1527161 | 7.31421 | 6.02532 | 7.02305 | Tumor |
| GSM1527163 | 7.2908 | 6.20205 | 7.0183 | Tumor |
| GSM1527165 | 7.31098 | 6.20586 | 6.8744 | Tumor |
| GSM1527167 | 7.57271 | 6.22511 | 6.66653 | Tumor |
| GSM1527169 | 6.97301 | 6.04905 | 6.56203 | Tumor |
| GSM1527171 | 6.5745 | 6.07641 | 6.84462 | Tumor |
| GSM1527173 | 7.58585 | 6.49948 | 6.65696 | Tumor |
| GSM1527175 | 7.11852 | 6.11365 | 6.50034 | Tumor |
| GSM1527177 | 7.09219 | 6.13306 | 6.70497 | Tumor |
| GSM1527179 | 6.8979 | 6.15334 | 6.48567 | Tumor |
| GSM1527181 | 7.17488 | 6.13829 | 6.9562 | Tumor |
| GSM1527183 | 7.41623 | 6.12343 | 7.03816 | Tumor |
| GSM1527185 | 5.68129 | 6.06733 | 6.25862 | Tumor |
| GSM1527187 | 7.68701 | 6.18596 | 6.82575 | Tumor |
| GSM1527189 | 7.35811 | 6.24486 | 6.33043 | Tumor |
| GSM1527191 | 7.5035 | 5.92183 | 6.77445 | Tumor |
| GSM1527193 | 7.02454 | 6.05867 | 6.90258 | Tumor |
| GSM1527196 | 6.77209 | 6.26695 | 6.70041 | Tumor |
| GSM1527198 | 7.98809 | 6.34735 | 6.75065 | Tumor |
| GSM1527199 | 7.29446 | 6.28655 | 6.36144 | Tumor |
| GSM1527200 | 7.60553 | 6.16713 | 6.68129 | Tumor |
| GSM1527202 | 7.30188 | 6.54987 | 6.71906 | Tumor |
| GSM1527204 | 7.57197 | 5.8246 | 6.46007 | Tumor |
| GSM1527205 | 6.92713 | 6.22596 | 6.51982 | Tumor |
| GSM1527207 | 7.54849 | 6.65752 | 6.53568 | Tumor |
| GSM1527209 | 6.88157 | 6.11231 | 6.6582 | Tumor |
| GSM1527210 | 7.38305 | 6.83432 | 6.52749 | Tumor |
| GSM1527212 | 7.48096 | 5.83824 | 6.83385 | Tumor |
| GSM1527213 | 7.20112 | 6.228 | 6.97833 | Tumor |
| GSM1527215 | 6.94661 | 6.07396 | 6.34559 | Tumor |
| GSM1527216 | 6.24313 | 6.07974 | 5.88901 | Tumor |
| GSM1527218 | 6.91862 | 7.10383 | 6.57811 | Tumor |
| GSM1527219 | 7.00551 | 6.10028 | 6.77845 | Tumor |
| GSM1527220 | 7.41095 | 6.79505 | 6.51384 | Tumor |
| GSM1527223 | 7.05704 | 6.16046 | 6.53244 | Tumor |
| GSM1527225 | 7.76842 | 6.1412 | 7.14318 | Tumor |
| GSM1527227 | 5.39961 | 5.27116 | 6.08208 | Tumor |
| GSM1527228 | 6.43564 | 6.0766 | 6.31214 | Tumor |
| GSM1527230 | 7.69789 | 6.09608 | 6.89 | Tumor |
| GSM1527232 | 7.82603 | 6.35796 | 6.577 | Tumor |
| GSM1527234 | 6.55036 | 6.23239 | 6.2195 | Tumor |

Additional file 1: Table S8: The database of GSE16515

| GSE15615 | Type | TMEM43  levels | PRPF3 levels | RAP2B levels |
| --- | --- | --- | --- | --- |
| GSM414924 | Pancreatic Sample 6-Tumor | 6.851218082 | 8.101188 | 7.708923 |
| GSM414925 | Pancreatic Sample 7-Tumor | 7.228650366 | 8.090642 | 7.82985 |
| GSM414926 | Pancreatic Sample 1-Tumor | 7.28271779 | 8.041659 | 8.094412 |
| GSM414927 | Pancreatic Sample 16-Tumor | 6.091541171 | 6.78136 | 5.834661 |
| GSM414929 | Pancreatic Sample 53-Tumor | 7.049031698 | 8.254084 | 7.684 |
| GSM414931 | Pancreatic Sample 9-Tumor | 6.858664869 | 7.969243 | 7.797337 |
| GSM414933 | Pancreatic Sample 11-Tumor | 5.98669535 | 8.538771 | 7.825319 |
| GSM414935 | Pancreatic Sample 12-Tumor | 6.941781242 | 7.529821 | 7.303902 |
| GSM414936 | Pancreatic Sample 13-Tumor | 7.319039816 | 7.585714 | 8.06255 |
| GSM414937 | Pancreatic Sample 54-Tumor | 6.973553879 | 7.430453 | 8.075586 |
| GSM414939 | Pancreatic Sample 15-Tumor | 7.031632288 | 8.32328 | 7.895686 |
| GSM414941 | Pancreatic Sample 14-Tumor | 7.077376536 | 7.563768 | 7.22033 |
| GSM414943 | Pancreatic Sample 17-Tumor | 7.033092576 | 8.251719 | 7.959635 |
| GSM414944 | Pancreatic Sample 20-Tumor | 7.586652169 | 8.494656 | 8.155274 |
| GSM414945 | Pancreatic Sample 5-Tumor | 5.977279923 | 7.552285 | 6.502606 |
| GSM414946 | Pancreatic Sample 4-Tumor | 7.660388167 | 8.412782 | 7.780244 |
| GSM414948 | Pancreatic Sample 19-Tumor | 6.857732233 | 7.132371 | 7.750473 |
| GSM414949 | Pancreatic Sample 21-Tumor | 7.005933264 | 7.105385 | 7.72176 |
| GSM414950 | Pancreatic Sample 22-Tumor | 6.868976845 | 7.516488 | 7.774567 |
| GSM414951 | Pancreatic Sample 23-Tumor | 6.794935663 | 7.627169 | 8.052351 |
| GSM414952 | Pancreatic Sample 24-Tumor | 6.889929788 | 7.580447 | 7.649472 |
| GSM414954 | Pancreatic Sample 25-Tumor | 6.55228484 | 8.0268 | 7.547563 |
| GSM414956 | Pancreatic Sample 30-Tumor | 6.876424857 | 7.855491 | 7.928962 |
| GSM414958 | Pancreatic Sample 26-Tumor | 6.866166169 | 7.790511 | 8.054161 |
| GSM414959 | Pancreatic Sample 28-Tumor | 7.312882955 | 8.018478 | 7.535353 |
| GSM414960 | Pancreatic Sample 32-Tumor | 6.622930351 | 7.430453 | 7.798979 |
| GSM414961 | Pancreatic Sample 29-Tumor | 7.012149614 | 7.176921 | 7.568209 |
| GSM414962 | Pancreatic Sample 31-Tumor | 6.01920151 | 6.621319 | 6.505891 |
| GSM414964 | Pancreatic Sample -Tumor | 6.848247353 | 7.863567 | 7.857255 |
| GSM414965 | Pancreatic Sample 33-Tumor | 6.998449397 | 7.93251 | 7.634279 |
| GSM414967 | Pancreatic Sample 27-Tumor | 7.45001524 | 8.645658 | 7.026321 |
| GSM414968 | Pancreatic Sample 34-Tumor | 6.788685711 | 7.208478 | 7.606418 |
| GSM414969 | Pancreatic Sample 35-Tumor | 6.590923827 | 7.584211 | 7.882297 |
| GSM414971 | Pancreatic Sample 36-Tumor | 7.090906345 | 7.063934 | 7.887749 |
| GSM414973 | Pancreatic Sample 37-Tumor | 7.036640695 | 7.23936 | 8.221603 |
| GSM414974 | Pancreatic Sample 38-Tumor | 6.721987779 | 7.428779 | 7.09399 |
| GSM414928 | Pancreatic Sample 16-Normal | 6.177369703 | 6.934281 | 6.293824 |
| GSM414930 | Pancreatic Sample 53-Normal | 5.739240553 | 7.433794 | 5.937501 |
| GSM414932 | Pancreatic Sample 9-Normal | 7.140829771 | 7.484622 | 7.550362 |
| GSM414934 | Pancreatic Sample 11-Normal | 6.882856993 | 7.253611 | 7.44802 |
| GSM414938 | Pancreatic Sample 54-Normal | 6.718019065 | 7.263034 | 6.098874 |
| GSM414940 | Pancreatic Sample 15-Normal | 6.648681141 | 7.565293 | 7.942749 |
| GSM414942 | Pancreatic Sample 14-Normal | 6.240505149 | 7.44791 | 5.304633 |
| GSM414947 | Pancreatic Sample 4-Normal | 5.732540561 | 7.266787 | 5.644625 |
| GSM414953 | Pancreatic Sample 24-Normal | 7.317141235 | 7.47978 | 7.95503 |
| GSM414955 | Pancreatic Sample 25-Normal | 7.013155152 | 7.634448 | 7.554205 |
| GSM414957 | Pancreatic Sample 30-Normal | 6.02989455 | 7.0268 | 6.344592 |
| GSM414963 | Pancreatic Sample 31-Normal | 6.848560349 | 7.145677 | 7.068026 |
| GSM414966 | Pancreatic Sample 33-Normal | 5.413289427 | 6.52904 | 5.278976 |
| GSM414970 | Pancreatic Sample 35-Normal | 6.718704106 | 7.150763 | 8.370891 |
| GSM414972 | Pancreatic Sample 36-Normal | 4.826675591 | 6.1152 | 4.8212 |
| GSM414975 | Pancreatic Sample 38-Normal | 5.229684048 | 6.885086 | 5.373764 |

Additional file 1: Table S9: The database of GSE28735

| GSE28735 | type | TMEM43 levels | PRPF3 evels | RAP2B evels |
| --- | --- | --- | --- | --- |
| GSM711905 | human pancreatic nontumor tissue, patient sample 1 | 5.52832 | 6.06964 | 5.56468 |
| GSM711907 | human pancreatic nontumor tissue, patient sample 2 | 6.49105 | 6.00671 | 6.45256 |
| GSM711909 | human pancreatic nontumor tissue, patient sample 3 | 7.42536 | 6.27433 | 6.54073 |
| GSM711911 | human pancreatic nontumor tissue, patient sample 4 | 6.91756 | 6.27943 | 6.1329 |
| GSM711913 | human pancreatic nontumor tissue, patient sample 5 | 6.93854 | 6.18555 | 5.94324 |
| GSM711915 | human pancreatic nontumor tissue, patient sample 6 | 7.57169 | 5.98197 | 6.57504 |
| GSM711917 | human pancreatic nontumor tissue, patient sample 7 | 6.35509 | 5.93667 | 5.80388 |
| GSM711919 | human pancreatic nontumor tissue, patient sample 8 | 7.71355 | 6.51864 | 6.28835 |
| GSM711921 | human pancreatic nontumor tissue, patient sample 9 | 7.50335 | 6.24584 | 6.28294 |
| GSM711923 | human pancreatic nontumor tissue, patient sample 10 | 7.35205 | 5.99005 | 6.34705 |
| GSM711925 | human pancreatic nontumor tissue, patient sample 11 | 6.05661 | 6.0255 | 5.74955 |
| GSM711927 | human pancreatic nontumor tissue, patient sample 12 | 7.21386 | 6.55316 | 6.52093 |
| GSM711929 | human pancreatic nontumor tissue, patient sample 13 | 6.10697 | 5.99263 | 5.55862 |
| GSM711931 | human pancreatic nontumor tissue, patient sample 14 | 6.34037 | 6.19062 | 6.23233 |
| GSM711933 | human pancreatic nontumor tissue, patient sample 15 | 7.11243 | 6.01115 | 6.75662 |
| GSM711935 | human pancreatic nontumor tissue, patient sample 16 | 5.65211 | 6.12021 | 5.51651 |
| GSM711937 | human pancreatic nontumor tissue, patient sample 17 | 6.23455 | 6.45896 | 5.47584 |
| GSM711939 | human pancreatic nontumor tissue, patient sample 18 | 6.38475 | 6.17429 | 5.77126 |
| GSM711941 | human pancreatic nontumor tissue, patient sample 19 | 7.15114 | 6.32441 | 6.49076 |
| GSM711943 | human pancreatic nontumor tissue, patient sample 20 | 7.12086 | 6.22263 | 6.07519 |
| GSM711945 | human pancreatic nontumor tissue, patient sample 21 | 7.15242 | 6.24829 | 6.0301 |
| GSM711947 | human pancreatic nontumor tissue, patient sample 22 | 7.40212 | 5.96932 | 6.30788 |
| GSM711949 | human pancreatic nontumor tissue, patient sample 23 | 5.49826 | 6.02895 | 5.47499 |
| GSM711951 | human pancreatic nontumor tissue, patient sample 24 | 6.68094 | 6.16451 | 6.12209 |
| GSM711953 | human pancreatic nontumor tissue, patient sample 25 | 6.26128 | 6.1583 | 5.7774 |
| GSM711955 | human pancreatic nontumor tissue, patient sample 26 | 7.48353 | 6.2162 | 6.45806 |
| GSM711957 | human pancreatic nontumor tissue, patient sample 27 | 6.11859 | 6.61472 | 5.26616 |
| GSM711959 | human pancreatic nontumor tissue, patient sample 28 | 5.54903 | 5.70829 | 6.24117 |
| GSM711961 | human pancreatic nontumor tissue, patient sample 29 | 6.30436 | 6.10257 | 5.41854 |
| GSM711963 | human pancreatic nontumor tissue, patient sample 30 | 5.72699 | 6.08928 | 5.60276 |
| GSM711965 | human pancreatic nontumor tissue, patient sample 31 | 7.27393 | 6.36169 | 6.89901 |
| GSM711967 | human pancreatic nontumor tissue, patient sample 32 | 6.80278 | 6.14562 | 6.38553 |
| GSM711969 | human pancreatic nontumor tissue, patient sample 33 | 6.51008 | 6.14111 | 5.75864 |
| GSM711971 | human pancreatic nontumor tissue, patient sample 34 | 7.63797 | 6.16902 | 6.48757 |
| GSM711973 | human pancreatic nontumor tissue, patient sample 35 | 6.89005 | 6.61986 | 6.1994 |
| GSM711975 | human pancreatic nontumor tissue, patient sample 36 | 6.40251 | 6.2471 | 5.59812 |
| GSM711977 | human pancreatic nontumor tissue, patient sample 37 | 7.00502 | 6.2986 | 6.00233 |
| GSM711979 | human pancreatic nontumor tissue, patient sample 38 | 7.00222 | 6.06831 | 6.46862 |
| GSM711981 | human pancreatic nontumor tissue, patient sample 42 | 6.9438 | 6.3699 | 6.45307 |
| GSM711983 | human pancreatic nontumor tissue, patient sample 43 | 7.57158 | 6.2772 | 6.45128 |
| GSM711985 | human pancreatic nontumor tissue, patient sample 44 | 6.12982 | 6.20762 | 5.72132 |
| GSM711987 | human pancreatic nontumor tissue, patient sample 45 | 5.7848 | 6.24521 | 5.4116 |
| GSM711989 | human pancreatic nontumor tissue, patient sample 39 | 6.62776 | 6.29483 | 6.06264 |
| GSM711991 | human pancreatic nontumor tissue, patient sample 40 | 7.38347 | 6.0838 | 6.31449 |
| GSM711993 | human pancreatic nontumor tissue, patient sample 41 | 6.84644 | 6.30882 | 6.44661 |
| GSM711904 | human pancreatic tumor tissue, patient sample 1 | 7.49906 | 6.41414 | 6.34136 |
| GSM711906 | human pancreatic tumor tissue, patient sample 2 | 7.24062 | 6.34764 | 6.71928 |
| GSM711908 | human pancreatic tumor tissue, patient sample 3 | 7.04156 | 6.42431 | 6.6829 |
| GSM711910 | human pancreatic tumor tissue, patient sample 4 | 7.67804 | 6.23851 | 6.22892 |
| GSM711912 | human pancreatic tumor tissue, patient sample 5 | 6.91879 | 6.18171 | 6.61259 |
| GSM711914 | human pancreatic tumor tissue, patient sample 6 | 7.23155 | 6.11449 | 6.48717 |
| GSM711916 | human pancreatic tumor tissue, patient sample 7 | 7.70417 | 6.17001 | 6.30671 |
| GSM711918 | human pancreatic tumor tissue, patient sample 8 | 6.64408 | 6.4742 | 6.51824 |
| GSM711922 | human pancreatic tumor tissue, patient sample 10 | 7.0372 | 6.3076 | 6.49338 |
| GSM711924 | human pancreatic tumor tissue, patient sample 11 | 7.1493 | 6.47472 | 6.39656 |
| GSM711926 | human pancreatic tumor tissue, patient sample 12 | 6.47043 | 6.3214 | 6.02734 |
| GSM711928 | human pancreatic tumor tissue, patient sample 13 | 7.58555 | 6.18266 | 6.20088 |
| GSM711930 | human pancreatic tumor tissue, patient sample 14 | 6.8676 | 6.94426 | 6.68023 |
| GSM711932 | human pancreatic tumor tissue, patient sample 15 | 6.83684 | 6.20963 | 6.47125 |
| GSM711934 | human pancreatic tumor tissue, patient sample 16 | 7.64453 | 6.11931 | 6.48916 |
| GSM711936 | human pancreatic tumor tissue, patient sample 17 | 6.23643 | 6.15879 | 6.05758 |
| GSM711938 | human pancreatic tumor tissue, patient sample 18 | 6.10411 | 6.17049 | 5.25992 |
| GSM711940 | human pancreatic tumor tissue, patient sample 19 | 7.23381 | 6.27173 | 6.72723 |
| GSM711942 | human pancreatic tumor tissue, patient sample 20 | 6.83344 | 6.48873 | 6.05641 |
| GSM711944 | human pancreatic tumor tissue, patient sample 21 | 7.35891 | 6.5239 | 6.43126 |
| GSM711946 | human pancreatic tumor tissue, patient sample 22 | 7.17009 | 6.35616 | 6.40454 |
| GSM711948 | human pancreatic tumor tissue, patient sample 23 | 7.25173 | 6.31416 | 6.53205 |
| GSM711950 | human pancreatic tumor tissue, patient sample 24 | 6.99849 | 6.15451 | 6.32123 |
| GSM711952 | human pancreatic tumor tissue, patient sample 25 | 7.4619 | 6.54919 | 7.21897 |
| GSM711954 | human pancreatic tumor tissue, patient sample 26 | 7.40854 | 6.02451 | 6.47256 |
| GSM711956 | human pancreatic tumor tissue, patient sample 27 | 7.33397 | 6.76831 | 5.92663 |
| GSM711958 | human pancreatic tumor tissue, patient sample 28 | 7.57218 | 6.46946 | 6.40558 |
| GSM711960 | human pancreatic tumor tissue, patient sample 29 | 7.42041 | 6.14856 | 6.84263 |
| GSM711962 | human pancreatic tumor tissue, patient sample 30 | 7.38 | 6.34324 | 6.8348 |
| GSM711964 | human pancreatic tumor tissue, patient sample 31 | 7.4059 | 6.36332 | 6.72455 |
| GSM711966 | human pancreatic tumor tissue, patient sample 32 | 7.67947 | 6.3567 | 6.47112 |
| GSM711968 | human pancreatic tumor tissue, patient sample 33 | 7.04523 | 6.17729 | 6.42851 |
| GSM711970 | human pancreatic tumor tissue, patient sample 34 | 6.64301 | 6.22021 | 6.63569 |
| GSM711972 | human pancreatic tumor tissue, patient sample 35 | 7.68954 | 6.62823 | 6.49603 |
| GSM711974 | human pancreatic tumor tissue, patient sample 36 | 7.21073 | 6.28034 | 6.28412 |
| GSM711976 | human pancreatic tumor tissue, patient sample 37 | 7.17313 | 6.25265 | 6.52516 |
| GSM711978 | human pancreatic tumor tissue, patient sample 38 | 6.98725 | 6.2817 | 6.37669 |
| GSM711980 | human pancreatic tumor tissue, patient sample 42 | 7.26566 | 6.29309 | 6.79304 |
| GSM711982 | human pancreatic tumor tissue, patient sample 43 | 7.51557 | 6.2656 | 6.87215 |
| GSM711984 | human pancreatic tumor tissue, patient sample 44 | 5.73028 | 6.22928 | 6.10065 |
| GSM711986 | human pancreatic tumor tissue, patient sample 45 | 7.79557 | 6.31112 | 6.58845 |
| GSM711988 | human pancreatic tumor tissue, patient sample 39 | 7.45171 | 6.37308 | 6.09822 |
| GSM711990 | human pancreatic tumor tissue, patient sample 40 | 7.60696 | 6.08024 | 6.66584 |
| GSM711992 | human pancreatic tumor tissue, patient sample 41 | 7.11233 | 6.22492 | 6.72108 |
| GSM711920 | human pancreatic tumor tissue, patient sample 9 | 6.81983 | 6.36599 | 6.34346 |

Additional file 1: Table S10: The database of GSE15471

| GSE15471 | type | TMEM43 levels | PRPF3 levels | RAP2B levels |
| --- | --- | --- | --- | --- |
| GSM388077 | N30162_rep | 6.383717 | 7.831897 | 6.119043 |
| GSM388079 | N40728_rep | 5.731052 | 6.323948 | 4.689408 |
| GSM388081 | N41027_rep | 5.91264 | 5.748753 | 4.769036 |
| GSM388082 | N30057 | 5.628976 | 6.114569 | 4.656658 |
| GSM388083 | N30068 | 6.85729 | 7.614825 | 5.666384 |
| GSM388084 | N30277 | 5.745887 | 6.070366 | 4.857765 |
| GSM388085 | N30308 | 7.098805 | 7.383335 | 5.781277 |
| GSM388086 | N30364 | 6.877939 | 6.814464 | 4.981201 |
| GSM388087 | N30582 | 6.618505 | 7.160744 | 5.166749 |
| GSM388088 | N30617 | 6.871885 | 7.225465 | 5.081715 |
| GSM388089 | N40645 | 5.982126 | 6.607736 | 5.597949 |
| GSM388090 | N40656 | 6.473578 | 6.985764 | 4.753649 |
| GSM388091 | N40726 | 7.368384 | 7.805455 | 5.830772 |
| GSM388092 | N40730 | 6.257786 | 6.826538 | 4.864127 |
| GSM388093 | N40741 | 5.848502 | 6.521867 | 4.778084 |
| GSM388094 | N40836 | 6.25858 | 6.286907 | 4.383818 |
| GSM388095 | N40843 | 5.675025 | 6.687042 | 4.784571 |
| GSM388096 | N40875 | 6.922111 | 7.111709 | 5.431223 |
| GSM388097 | N40892 | 7.7489 | 7.395632 | 6.060736 |
| GSM388098 | N40899 | 5.897921 | 6.88017 | 4.784115 |
| GSM388099 | N40975 | 6.380371 | 6.599826 | 5.081061 |
| GSM388100 | N40977 | 5.99254 | 6.764054 | 4.950378 |
| GSM388101 | N51084 | 6.205707 | 6.162296 | 4.998961 |
| GSM388102 | N51091 | 6.116786 | 6.759626 | 4.759359 |
| GSM388103 | N51176 | 6.332268 | 6.58652 | 4.823322 |
| GSM388104 | N51292 | 6.810444 | 7.64951 | 5.968751 |
| GSM388105 | N51294 | 7.033743 | 7.057365 | 5.924572 |
| GSM388106 | N51308 | 6.214348 | 7.145827 | 4.874295 |
| GSM388107 | N51315 | 6.391398 | 7.060039 | 4.68437 |
| GSM388108 | N51572 | 6.311536 | 6.311759 | 4.550704 |
| GSM388109 | N51628 | 6.038931 | 6.101564 | 4.742237 |
| GSM388110 | N51677 | 6.027858 | 6.642118 | 4.537844 |
| GSM388111 | N51681 | 5.556512 | 6.190573 | 4.890655 |
| GSM388112 | N51721 | 5.983033 | 6.651604 | 4.493687 |
| GSM388113 | N51722 | 6.013064 | 5.938193 | 4.829903 |
| GSM388114 | N51783 | 6.860837 | 8.045989 | 5.793341 |
| GSM388116 | T30162_rep | 6.677979 | 7.546902 | 6.044747 |
| GSM388118 | T40728_rep | 6.853095 | 7.63045 | 5.834941 |
| GSM388120 | T41027_rep | 7.085614 | 7.309078 | 5.680545 |
| GSM388121 | T30057 | 7.224882 | 7.850325 | 5.804127 |
| GSM388122 | T30068 | 7.721887 | 7.260542 | 5.444593 |
| GSM388123 | T30277 | 7.151611 | 6.696029 | 5.29569 |
| GSM388124 | T30308 | 6.694161 | 7.510964 | 5.795238 |
| GSM388125 | T30364 | 7.163692 | 7.422154 | 5.774435 |
| GSM388126 | T30582 | 6.830688 | 7.164378 | 5.441814 |
| GSM388127 | T30617 | 6.911389 | 6.949921 | 5.495478 |
| GSM388128 | T40645 | 7.313575 | 7.460084 | 5.820565 |
| GSM388129 | T40656 | 6.723892 | 7.518284 | 6.070644 |
| GSM388130 | T40726 | 7.303755 | 7.898407 | 5.469842 |
| GSM388131 | T40730 | 6.848763 | 7.210859 | 5.780097 |
| GSM388132 | T40741 | 6.325637 | 7.216885 | 5.651416 |
| GSM388133 | T40836 | 6.917455 | 7.37008 | 6.027924 |
| GSM388134 | T40843 | 6.714713 | 6.928452 | 5.477591 |
| GSM388135 | T40875 | 7.1795 | 7.326757 | 5.550934 |
| GSM388136 | T40892 | 7.154257 | 7.430525 | 6.127045 |
| GSM388137 | T40899 | 6.844595 | 7.376788 | 6.529163 |
| GSM388138 | T40975 | 6.983412 | 7.633829 | 5.688875 |
| GSM388139 | T40977 | 6.959636 | 7.716022 | 6.10426 |
| GSM388140 | T51084 | 7.177163 | 6.876504 | 5.262405 |
| GSM388141 | T51091 | 7.12654 | 7.425674 | 5.761054 |
| GSM388142 | T51176 | 7.012659 | 7.342568 | 5.849297 |
| GSM388143 | T51292 | 7.190897 | 7.580378 | 5.723956 |
| GSM388144 | T51294 | 6.902279 | 8.078588 | 5.872296 |
| GSM388145 | T51308 | 6.235323 | 6.969843 | 5.395687 |
| GSM388146 | T51315 | 7.002227 | 7.718709 | 5.897801 |
| GSM388147 | T51572 | 6.930812 | 7.527002 | 5.612503 |
| GSM388148 | T51628 | 6.771494 | 7.087794 | 5.520412 |
| GSM388149 | T51677 | 6.800134 | 7.577846 | 6.094534 |
| GSM388150 | T51681 | 7.096591 | 7.705049 | 5.865176 |
| GSM388151 | T51721 | 7.091776 | 7.592108 | 5.746788 |
| GSM388152 | T51722 | 6.884842 | 7.552041 | 5.779813 |
| GSM388153 | T51783 | 6.813906 | 7.039613 | 5.177333 |

Additional file 1: Table S10: The database of GSE71729

| ID | Type | TMEM43 levels | PRPF3 levels | RAP2B levels |
| --- | --- | --- | --- | --- |
| T1 | Tumor | 5.492317 | 3.991034 | 2.92758 |
| T2 | Tumor | 7.038919 | 3.921762 | 2.287501 |
| T3 | Tumor | 6.459566 | 3.632507 | 3.808664 |
| T4 | Tumor | 6.428852 | 3.597704 | 2.499975 |
| T5 | Tumor | 5.308028 | 3.013843 | 1.948106 |
| T6 | Tumor | 5.215343 | 3.566723 | 1.75163 |
| T7 | Tumor | 5.624541 | 2.270155 | 2.545861 |
| T8 | Tumor | 5.388186 | 2.845216 | 1.988828 |
| T9 | Tumor | 6.382972 | 3.053504 | 2.55258 |
| T10 | Tumor | 6.991261 | 3.093234 | 2.081878 |
| T11 | Tumor | 5.767761 | 3.083766 | 2.078406 |
| T12 | Tumor | 6.025574 | 3.014305 | 2.270744 |
| T13 | Tumor | 6.77035 | 2.928443 | 2.224821 |
| T14 | Tumor | 5.917926 | 3.290431 | 2.48688 |
| T15 | Tumor | 6.818078 | 2.907759 | 2.206053 |
| T16 | Tumor | 5.668 | 2.492571 | 2.313944 |
| T17 | Tumor | 5.467312 | 3.051552 | 2.468513 |
| T18 | Tumor | 6.175028 | 3.123308 | 2.247081 |
| T19 | Tumor | 5.297006 | 3.199846 | 2.320104 |
| T20 | Tumor | 5.480224 | 3.085266 | 1.599669 |
| T21 | Tumor | 5.931882 | 3.504276 | 2.563008 |
| T22 | Tumor | 5.795958 | 2.616852 | 1.729482 |
| T23 | Tumor | 5.260434 | 2.206053 | 2.367465 |
| T24 | Tumor | 6.050422 | 3.526852 | 1.514896 |
| T25 | Tumor | 6.761157 | 3.111653 | 2.103874 |
| T26 | Tumor | 6.217483 | 3.115134 | 2.797216 |
| T27 | Tumor | 5.28601 | 2.856095 | 2.786955 |
| T28 | Tumor | 5.625824 | 2.136861 | 2.443319 |
| T29 | Tumor | 6.178944 | 3.160692 | 2.724394 |
| T30 | Tumor | 5.523527 | 2.389322 | 2.597182 |
| T31 | Tumor | 5.859992 | 3.659382 | 2.505957 |
| T32 | Tumor | 5.91321 | 2.808994 | 2.760535 |
| T33 | Tumor | 6.498048 | 2.847683 | 2.38023 |
| T34 | Tumor | 6.012618 | 2.79271 | 2.739014 |
| T35 | Tumor | 5.899549 | 2.45751 | 2.409308 |
| T36 | Tumor | 6.386678 | 2.747429 | 2.689246 |
| T37 | Tumor | 5.554451 | 2.504343 | 2.432916 |
| T38 | Tumor | 6.488706 | 3.353815 | 2.724776 |
| T39 | Tumor | 6.245588 | 3.040566 | 2.436249 |
| T40 | Tumor | 5.835246 | 4.043003 | 2.659836 |
| T41 | Tumor | 5.790661 | 3.205403 | 2.32388 |
| T42 | Tumor | 6.358574 | 3.004401 | 2.82169 |
| T43 | Tumor | 5.848485 | 3.919087 | 2.49273 |
| T44 | Tumor | 5.558034 | 3.545521 | 2.082441 |
| T45 | Tumor | 6.169457 | 2.700683 | 2.457793 |
| T46 | Tumor | 6.836686 | 3.359765 | 1.939639 |
| T47 | Tumor | 6.08244 | 2.827529 | 2.89605 |
| T48 | Tumor | 6.457275 | 2.682261 | 2.927748 |
| T49 | Tumor | 5.983826 | 2.957472 | 2.800364 |
| T50 | Tumor | 6.062415 | 2.675682 | 2.410994 |
| T51 | Tumor | 6.101817 | 2.890472 | 2.72191 |
| T52 | Tumor | 6.003339 | 3.261006 | 2.589613 |
| T53 | Tumor | 5.692737 | 2.675406 | 2.842043 |
| T54 | Tumor | 5.477629 | 2.20809 | 2.323594 |
| T55 | Tumor | 6.499046 | 3.197566 | 2.551941 |
| T56 | Tumor | 5.748847 | 3.355308 | 2.003263 |
| T57 | Tumor | 6.635239 | 2.639283 | 2.727244 |
| T58 | Tumor | 5.708542 | 2.677132 | 2.258717 |
| T59 | Tumor | 6.101817 | 2.780062 | 2.302527 |
| T60 | Tumor | 5.190148 | 3.0968 | 1.941728 |
| T61 | Tumor | 5.933283 | 2.079304 | 2.256465 |
| T62 | Tumor | 6.277891 | 3.185941 | 2.633693 |
| T63 | Tumor | 6.170608 | 3.069034 | 2.800754 |
| T64 | Tumor | 6.179675 | 3.543734 | 2.745751 |
| T65 | Tumor | 6.662798 | 2.85391 | 3.120464 |
| T66 | Tumor | 6.457692 | 3.302325 | 2.948294 |
| T67 | Tumor | 5.753549 | 3.012776 | 2.506455 |
| T68 | Tumor | 6.325192 | 3.312588 | 2.78849 |
| T69 | Tumor | 6.885384 | 3.03309 | 3.005473 |
| T70 | Tumor | 6.404406 | 3.757697 | 2.510867 |
| T71 | Tumor | 5.368833 | 3.901734 | 2.057654 |
| T72 | Tumor | 6.403527 | 3.625545 | 2.509375 |
| T73 | Tumor | 5.491689 | 3.408056 | 2.216232 |
| T74 | Tumor | 6.459112 | 3.441347 | 2.971646 |
| T75 | Tumor | 6.524755 | 3.467164 | 3.04952 |
| T76 | Tumor | 5.368298 | 3.443385 | 2.316944 |
| T77 | Tumor | 6.284325 | 3.371798 | 2.134127 |
| T78 | Tumor | 6.582824 | 4.165929 | 2.858958 |
| T79 | Tumor | 6.606207 | 3.797697 | 3.249843 |
| T80 | Tumor | 5.279655 | 3.67523 | 1.976087 |
| T81 | Tumor | 6.184648 | 3.665014 | 2.772129 |
| T82 | Tumor | 6.521325 | 3.833387 | 2.904286 |
| T83 | Tumor | 6.932132 | 3.88752 | 2.735641 |
| T84 | Tumor | 6.626144 | 3.424239 | 2.545098 |
| T85 | Tumor | 5.259015 | 3.873106 | 3.467754 |
| T86 | Tumor | 7.403532 | 3.093944 | 2.62958 |
| T87 | Tumor | 6.295417 | 3.448676 | 2.820703 |
| T88 | Tumor | 5.91909 | 3.842846 | 3.481148 |
| T89 | Tumor | 5.663969 | 4.403176 | 2.686683 |
| T90 | Tumor | 6.549312 | 3.13441 | 3.034832 |
| T91 | Tumor | 6.052885 | 3.259335 | 2.902794 |
| T92 | Tumor | 5.498689 | 3.937118 | 2.908182 |
| T93 | Tumor | 6.029164 | 3.3002 | 2.575625 |
| T94 | Tumor | 6.034786 | 3.621319 | 2.648119 |
| T95 | Tumor | 6.270909 | 3.600174 | 2.832294 |
| T96 | Tumor | 6.279182 | 3.48502 | 2.682261 |
| T97 | Tumor | 5.774101 | 3.585521 | 2.952297 |
| T98 | Tumor | 5.810275 | 3.195634 | 2.613605 |
| T99 | Tumor | 6.061997 | 3.314357 | 3.169619 |
| T100 | Tumor | 6.597857 | 3.883643 | 2.638515 |
| T101 | Tumor | 5.450354 | 3.213613 | 2.123154 |
| T102 | Tumor | 6.371678 | 3.660279 | 2.843104 |
| T103 | Tumor | 6.164835 | 3.643227 | 3.139003 |
| T104 | Tumor | 6.044825 | 3.660279 | 2.271961 |
| T105 | Tumor | 6.227947 | 3.830053 | 3.003894 |
| T106 | Tumor | 6.530583 | 3.96709 | 3.069513 |
| T107 | Tumor | 5.923098 | 3.526683 | 2.647168 |
| T108 | Tumor | 6.891303 | 3.418155 | 2.783003 |
| T109 | Tumor | 6.303905 | 3.642353 | 2.813185 |
| T110 | Tumor | 6.008591 | 3.347465 | 2.866027 |
| T111 | Tumor | 5.412849 | 3.024815 | 2.484239 |
| T112 | Tumor | 5.996485 | 3.32598 | 2.84723 |
| T113 | Tumor | 5.77737 | 3.448235 | 2.929332 |
| T114 | Tumor | 5.765647 | 4.106182 | 2.515808 |
| T115 | Tumor | 6.087669 | 3.276437 | 2.996263 |
| T116 | Tumor | 6.016997 | 3.373293 | 2.955758 |
| T117 | Tumor | 5.971492 | 3.930373 | 2.437333 |
| T118 | Tumor | 6.015877 | 3.471087 | 3.01764 |
| T119 | Tumor | 5.635021 | 4.295182 | 2.54 |
| T120 | Tumor | 5.945377 | 3.767413 | 2.44022 |
| T121 | Tumor | 5.562919 | 3.20556 | 2.362566 |
| T122 | Tumor | 5.883049 | 3.442266 | 2.728259 |
| T123 | Tumor | 5.420714 | 3.779123 | 2.53862 |
| T124 | Tumor | 6.38619 | 3.492118 | 2.544395 |
| T125 | Tumor | 6.156268 | 3.364724 | 2.220493 |
| T126 | Tumor | 5.610539 | 3.358494 | 2.786678 |
| T127 | Tumor | 5.561258 | 4.006347 | 2.674392 |
| T128 | Tumor | 5.409384 | 2.92669 | 1.677076 |
| T129 | Tumor | 6.340903 | 3.366885 | 2.193863 |
| T130 | Tumor | 5.96765 | 3.534535 | 2.414258 |
| T131 | Tumor | 5.856482 | 3.883989 | 2.673606 |
| T132 | Tumor | 6.205599 | 3.642896 | 2.332479 |
| T133 | Tumor | 6.788336 | 3.403648 | 2.624333 |
| T134 | Tumor | 6.21221 | 3.722548 | 2.657398 |
| T135 | Tumor | 5.999126 | 3.576277 | 2.335417 |
| T136 | Tumor | 6.071476 | 3.93807 | 2.807738 |
| T137 | Tumor | 6.594938 | 4.04372 | 2.765423 |
| T138 | Tumor | 6.048496 | 3.198193 | 2.574403 |
| T139 | Tumor | 6.553261 | 3.049661 | 2.563689 |
| T140 | Tumor | 6.372154 | 4.077443 | 2.237415 |
| T141 | Tumor | 6.398846 | 3.226857 | 2.554246 |
| T142 | Tumor | 6.674077 | 3.823514 | 2.312703 |
| T143 | Tumor | 5.557737 | 3.775076 | 2.210987 |
| T144 | Tumor | 5.801374 | 3.32812 | 2.922555 |
| T145 | Tumor | 5.482325 | 4.174829 | 2.114997 |
| T146 | Tumor | 5.802045 | 3.357711 | 2.543167 |
| T147 | Tumor | 5.973046 | 2.949602 | 2.675258 |
| T148 | Tumor | 6.583291 | 3.435902 | 2.717549 |
| T149 | Tumor | 5.317284 | 2.563555 | 2.006232 |
| T150 | Tumor | 5.439423 | 3.053367 | 2.611328 |
| T151 | Tumor | 5.757891 | 3.042129 | 3.034003 |
| T152 | Tumor | 5.296703 | 3.334947 | 2.716434 |
| T153 | Tumor | 6.141179 | 3.017465 | 2.237958 |
| T154 | Tumor | 6.82856 | 3.985465 | 2.952826 |
| T155 | Tumor | 6.629798 | 3.989455 | 2.286468 |
| T156 | Tumor | 7.135039 | 3.759615 | 2.726347 |
| T157 | Tumor | 8.011924 | 3.415819 | 2.838195 |
| T158 | Tumor | 6.728375 | 3.986546 | 2.676821 |
| T159 | Tumor | 6.293737 | 3.469493 | 2.504207 |
| T160 | Tumor | 7.276027 | 3.833078 | 2.960349 |
| T161 | Tumor | 7.226591 | 3.881457 | 2.465702 |
| T162 | Tumor | 7.976751 | 4.352036 | 2.619176 |
| T163 | Tumor | 6.284325 | 3.869275 | 2.660692 |
| T164 | Tumor | 6.30171 | 3.511116 | 2.468112 |
| T165 | Tumor | 7.213199 | 3.999944 | 2.516293 |
| T166 | Tumor | 6.350036 | 4.074882 | 2.156209 |
| T167 | Tumor | 6.569644 | 2.758014 | 2.413975 |
| T168 | Tumor | 4.75651 | 4.860978 | 2.492006 |
| T169 | Tumor | 4.889702 | 5.065992 | 2.405961 |
| T170 | Tumor | 6.416605 | 3.885916 | 2.561108 |
| T171 | Tumor | 6.020035 | 3.434258 | 2.660692 |
| T172 | Tumor | 6.283102 | 3.414637 | 2.673048 |
| T173 | Tumor | 6.329566 | 3.712577 | 2.400789 |
| T174 | Tumor | 6.610223 | 3.565434 | 2.285885 |
| T175 | Tumor | 6.406258 | 3.951608 | 2.738863 |
| T176 | Tumor | 6.203578 | 3.637868 | 2.797384 |
| T177 | Tumor | 6.297539 | 3.705587 | 3.425157 |
| T178 | Tumor | 6.233926 | 3.834874 | 2.392338 |
| T179 | Tumor | 5.932934 | 4.003654 | 2.972476 |
| T180 | Tumor | 5.819356 | 3.337478 | 2.916986 |
| T181 | Tumor | 5.308602 | 3.285804 | 2.790095 |
| T182 | Tumor | 5.672958 | 2.792985 | 2.582227 |
| T183 | Tumor | 5.423748 | 3.727989 | 2.243857 |
| T184 | Tumor | 6.595408 | 3.490692 | 2.374919 |
| T185 | Tumor | 5.812332 | 3.854874 | 2.822227 |
| T186 | Tumor | 5.941627 | 3.011458 | 3.139794 |
| T187 | Tumor | 5.536136 | 3.755125 | 2.693742 |
| T188 | Tumor | 5.548885 | 3.458459 | 2.563689 |
| T189 | Tumor | 6.596899 | 4.315373 | 2.925952 |
| T190 | Tumor | 5.580634 | 4.041955 | 2.715325 |
| T191 | Tumor | 6.551008 | 3.539507 | 2.612406 |
| T192 | Tumor | 5.514317 | 3.580913 | 2.852389 |
| T193 | Tumor | 5.709902 | 2.742297 | 2.09781 |
| T194 | Tumor | 6.685966 | 3.621846 | 2.874885 |
| T195 | Tumor | 6.200158 | 4.451664 | 2.976625 |
| T196 | Tumor | 6.496605 | 3.869947 | 2.480335 |
| T197 | Tumor | 5.940564 | 4.081221 | 2.320665 |
| T198 | Tumor | 6.219217 | 4.808591 | 2.289286 |
| T199 | Tumor | 5.150641 | 2.492888 | 3.410224 |
| T200 | Tumor | 6.123445 | 2.112637 | 2.749961 |
| T201 | Tumor | 5.245 | 3.087193 | 2.212401 |
| T202 | Tumor | 6.017763 | 3.668737 | 2.966501 |
| T203 | Tumor | 5.387347 | 3.654441 | 2.346825 |
| T204 | Tumor | 5.405941 | 3.426144 | 2.404689 |
| T205 | Tumor | 5.057183 | 3.417496 | 3.172252 |
| T206 | Tumor | 6.439297 | 3.203479 | 2.692553 |
