# Supplementary material for: TMEM43 promotes pancreatic cancer progression by stabilizing PRPF3 and regulating RAP2B/ERK axis
Source: Cell Mol Biol Lett. 2022 Mar 8;27:24. doi: 10.1186/s11658-022-00321-z (PMC8903684; doi:10.1186/s11658-022-00321-z)

**MIAPaCa-2 cells STR report**


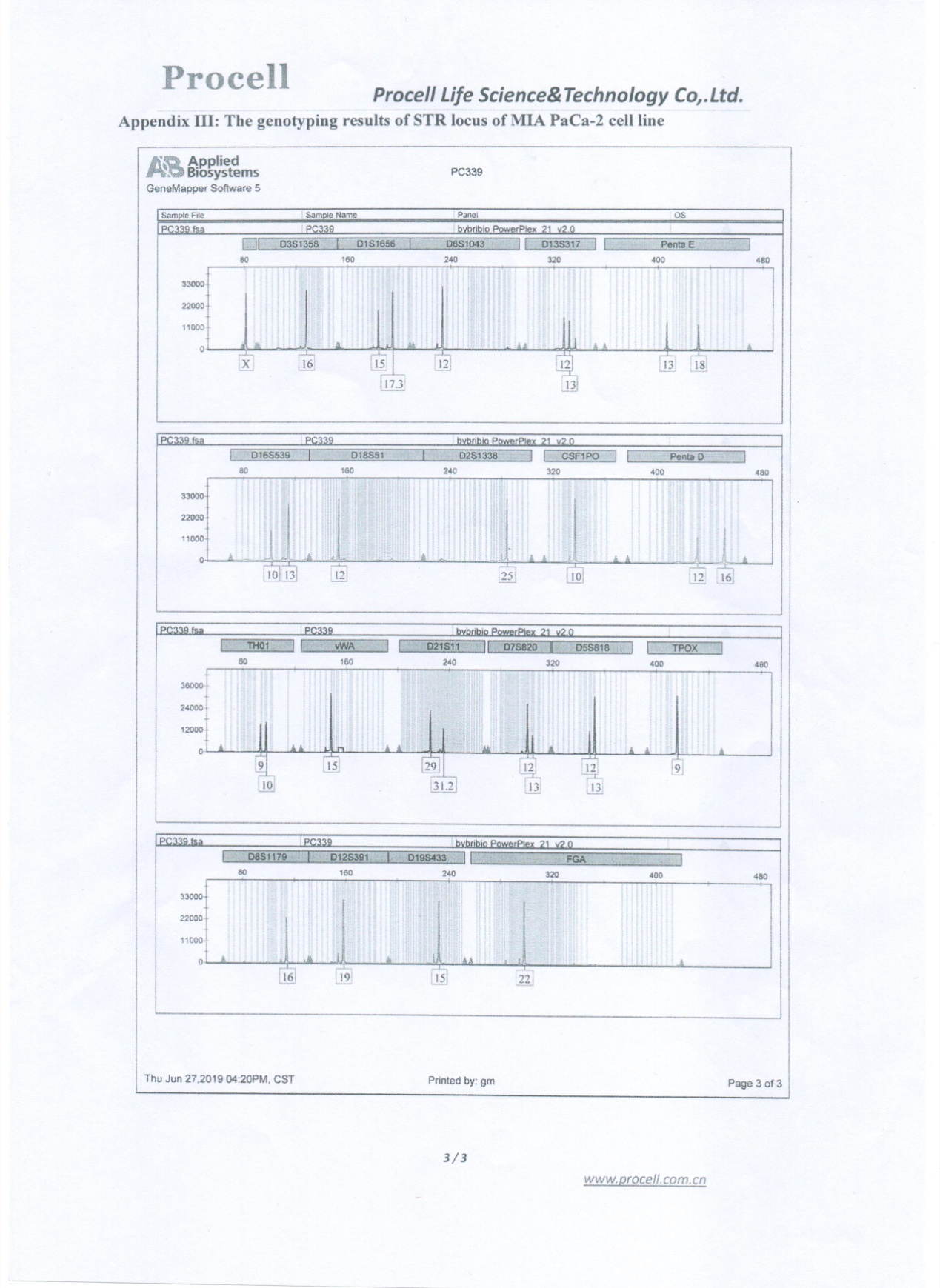


**SW1990 cells STR report**


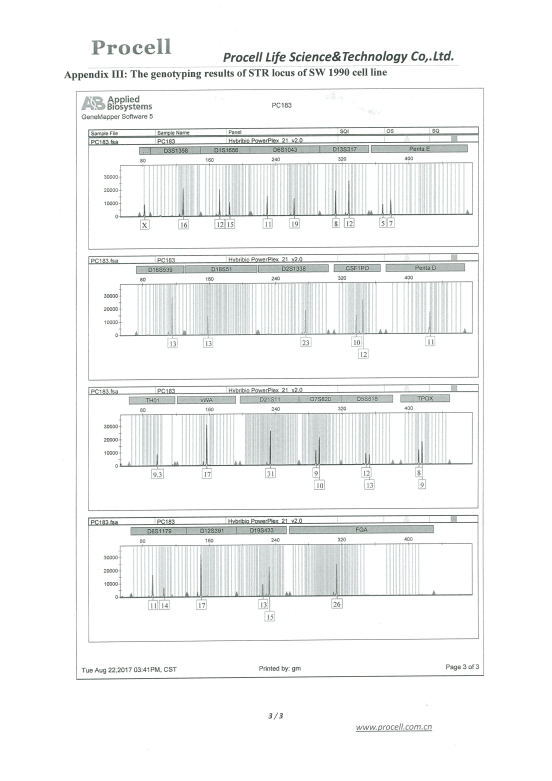
**Capan-2 cells STR report**


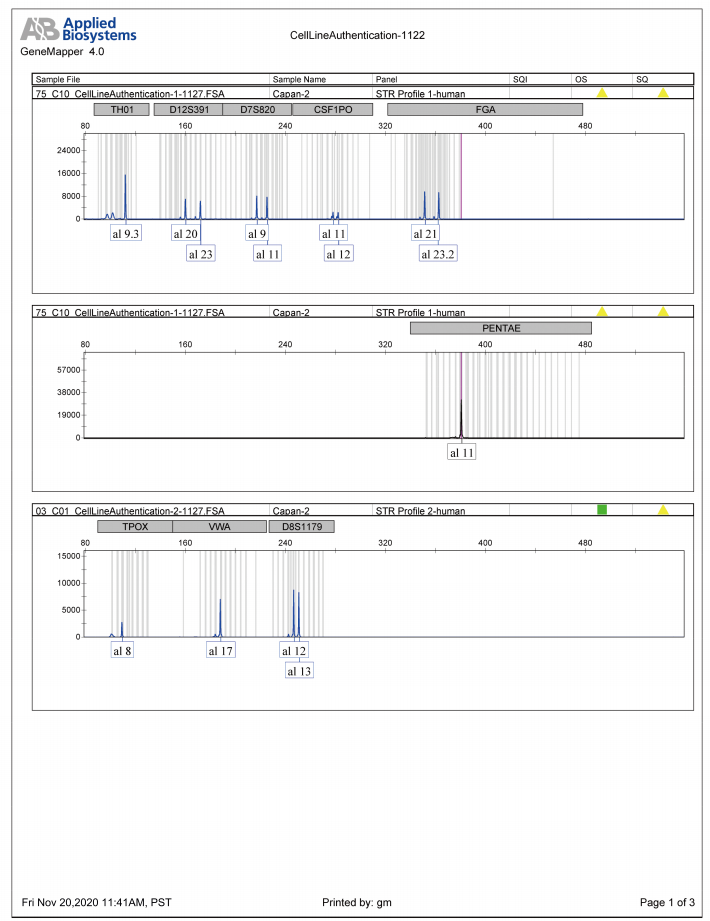


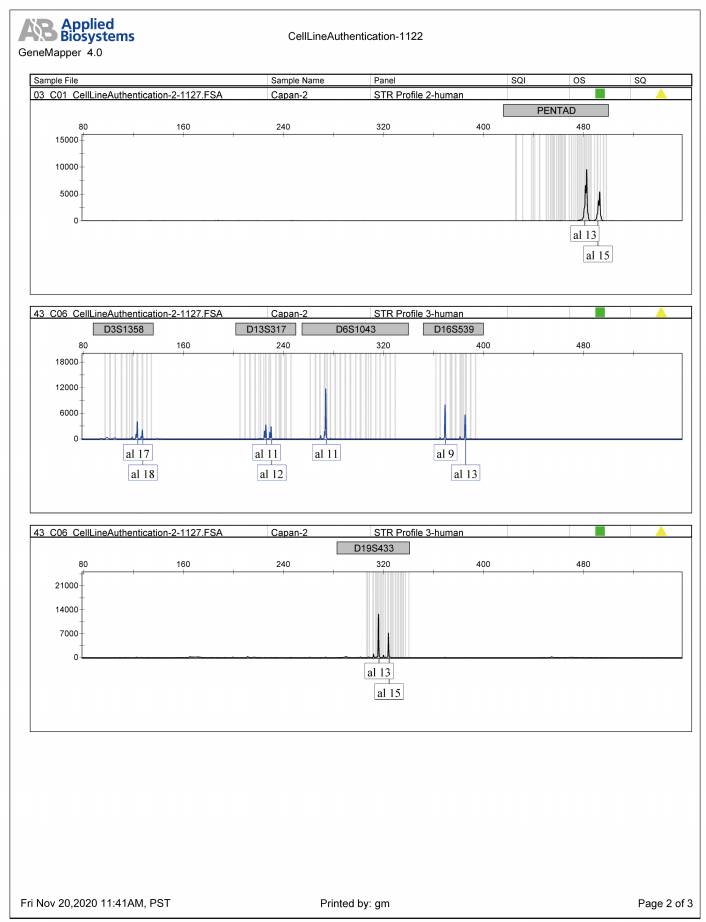


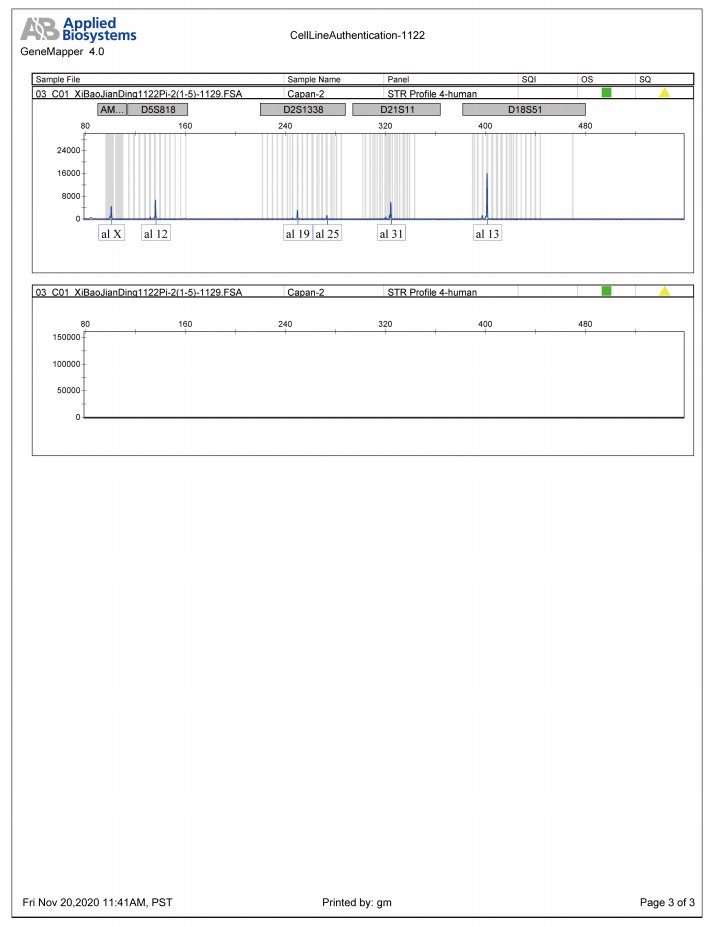


The original data of qRT-PCR

| ID | Gapdh(ct value) | TMEM43(ct value) | PRPF3(ct value) |
| --- | --- | --- | --- |
| MIAPaCa-2-NC-1 | 12.60 | 19.25 | 19.78 |
| MIAPaCa-2-NC-2 | 12.53 | 19.24 | 19.79 |
| MIAPaCa-2-NC-3 | 12.02 | 19.29 | 19.58 |
| MIAPaCa-2-NC-4 | 11.85 | 19.03 | 19.48 |
| MIAPaCa-2-NC-5 | 12.20 | 18.94 | 19.40 |
| MIAPaCa-2-NC-6 | 12.27 | 18.85 | 19.41 |
| MIAPaCa-2-NC-7 | 12.00 | 19.19 | 19.52 |
| MIAPaCa-2-NC-8 | 12.61 | 19.27 | 19.68 |
| MIAPaCa-2-NC-9 | 12.25 | 19.10 | 19.62 |
| MIAPaCa-2-shTMEM43-1 | 13.04 | 20.62 | 20.05 |
| MIAPaCa-2-shTMEM43-2 | 13.02 | 20.78 | 20.04 |
| MIAPaCa-2-shTMEM43-3 | 13.44 | 20.69 | 20.04 |
| MIAPaCa-2-shTMEM43-4 | 13.07 | 20.75 | 20.28 |
| MIAPaCa-2-shTMEM43-5 | 13.10 | 20.77 | 20.18 |
| MIAPaCa-2-shTMEM43-6 | 12.93 | 20.63 | 20.18 |
| MIAPaCa-2-shTMEM43-7 | 13.07 | 20.57 | 20.11 |
| MIAPaCa-2-shTMEM43-8 | 12.96 | 20.30 | 20.05 |
| MIAPaCa-2-shTMEM43-9 | 13.06 | 20.49 | 20.20 |
| MIAPaCa-2-shTMEM43+vector-1 | 13.02 | 20.58 | 19.96 |
| MIAPaCa-2-shTMEM43+vector-2 | 13.19 | 20.63 | 20.14 |
| MIAPaCa-2-shTMEM43+vector-3 | 13.09 | 20.65 | 20.15 |
| MIAPaCa-2-shTMEM43+vector-4 | 13.28 | 20.83 | 20.28 |
| MIAPaCa-2-shTMEM43+vector-5 | 13.22 | 20.78 | 20.31 |
| MIAPaCa-2-shTMEM43+vector-6 | 13.58 | 20.84 | 20.30 |
| MIAPaCa-2-shTMEM43+vector-7 | 13.11 | 20.56 | 20.23 |
| MIAPaCa-2-shTMEM43+vector-8 | 13.16 | 20.71 | 20.28 |
| MIAPaCa-2-shTMEM43+vector-9 | 13.10 | 20.55 | 20.18 |
| MIAPaCa-2-shTMEM43+Flag-TMEM43-1 | 13.21 | 17.52 | 20.02 |
| MIAPaCa-2-shTMEM43+Flag-TMEM43-2 | 13.17 | 17.55 | 20.02 |
| MIAPaCa-2-shTMEM43+Flag-TMEM43-3 | 13.04 | 17.38 | 19.92 |
| MIAPaCa-2-shTMEM43+Flag-TMEM43-4 | 12.86 | 17.37 | 20.05 |
| MIAPaCa-2-shTMEM43+Flag-TMEM43-5 | 12.92 | 17.50 | 20.04 |
| MIAPaCa-2-shTMEM43+Flag-TMEM43-6 | 12.82 | 17.47 | 19.88 |
| MIAPaCa-2-shTMEM43+Flag-TMEM43-7 | 12.99 | 17.70 | 20.23 |
| MIAPaCa-2-shTMEM43+Flag-TMEM43-8 | 13.03 | 17.78 | 20.16 |
| MIAPaCa-2-shTMEM43+Flag-TMEM43-9 | 13.08 | 17.69 | 20.18 |
| SW1990-NC-1 | 13.18 | 19.70 | 20.09 |
| SW1990-NC-2 | 13.17 | 19.80 | 20.02 |
| SW1990-NC-3 | 13.16 | 19.67 | 19.93 |
| SW1990-NC-4 | 13.27 | 19.71 | 19.99 |
| SW1990-NC-5 | 13.04 | 19.61 | 19.79 |
| SW1990-NC-6 | 12.80 | 19.53 | 19.69 |
| SW1990-NC-7 | 13.09 | 19.69 | 19.73 |
| SW1990-NC-8 | 13.07 | 19.66 | 19.93 |
| SW1990-NC-9 | 13.17 | 19.60 | 19.74 |
| SW1990-shTMEM43-1 | 13.35 | 20.57 | 20.14 |
| SW1990-shTMEM43-2 | 13.20 | 20.54 | 20.22 |
| SW1990-shTMEM43-3 | 13.33 | 20.44 | 20.17 |
| SW1990-shTMEM43-4 | 13.42 | 20.36 | 20.10 |
| SW1990-shTMEM43-5 | 13.16 | 20.33 | 20.10 |
| SW1990-shTMEM43-6 | 13.10 | 20.26 | 19.99 |
| SW1990-shTMEM43-7 | 13.36 | 20.27 | 20.05 |
| SW1990-shTMEM43-8 | 13.24 | 20.31 | 20.03 |
| SW1990-shTMEM43-9 | 13.18 | 20.14 | 19.86 |

The original data of western blots

Fig 1B-TMEM43


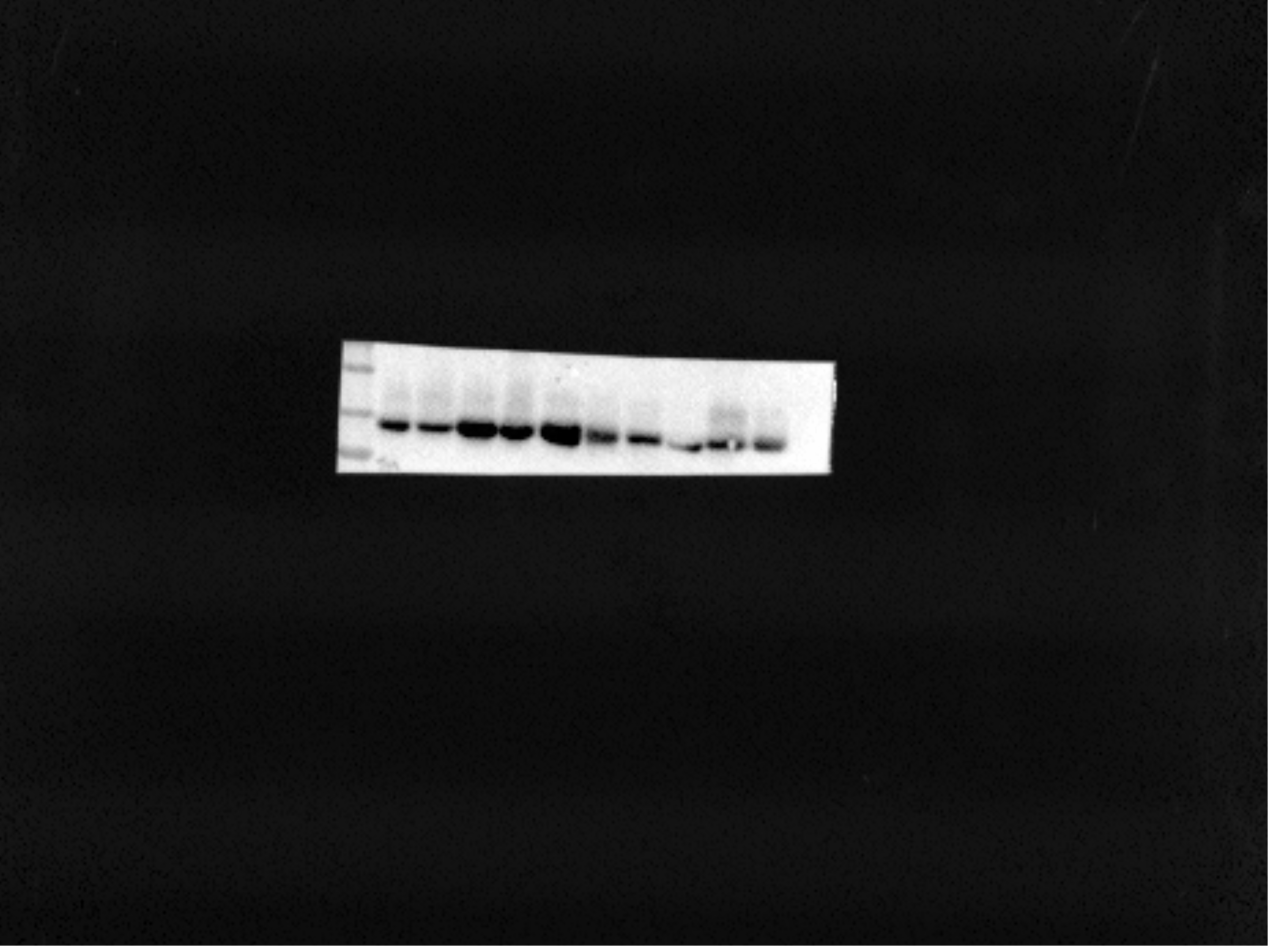


Fig 1B-β-actin


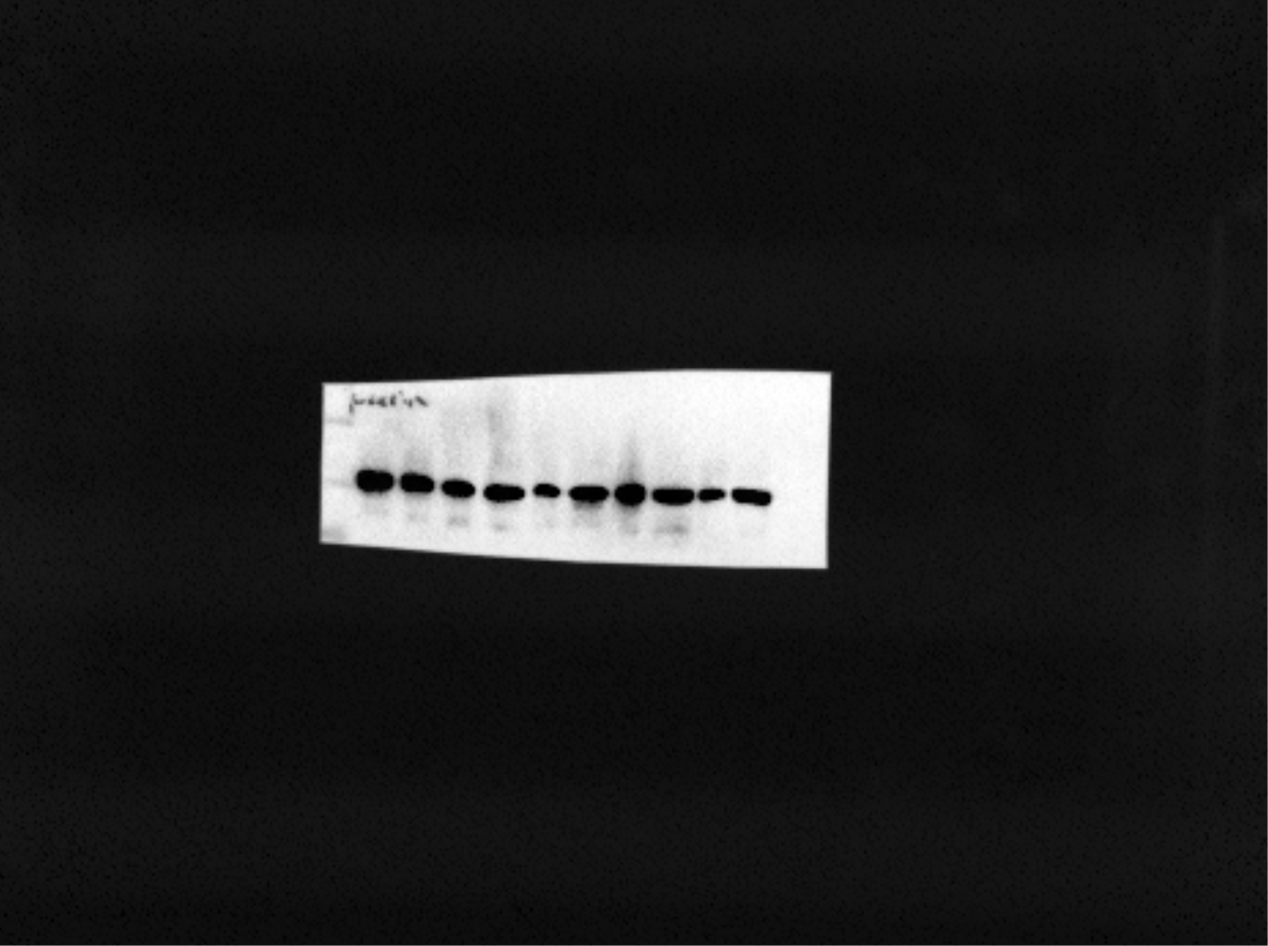


Fig 2A-TMEM43


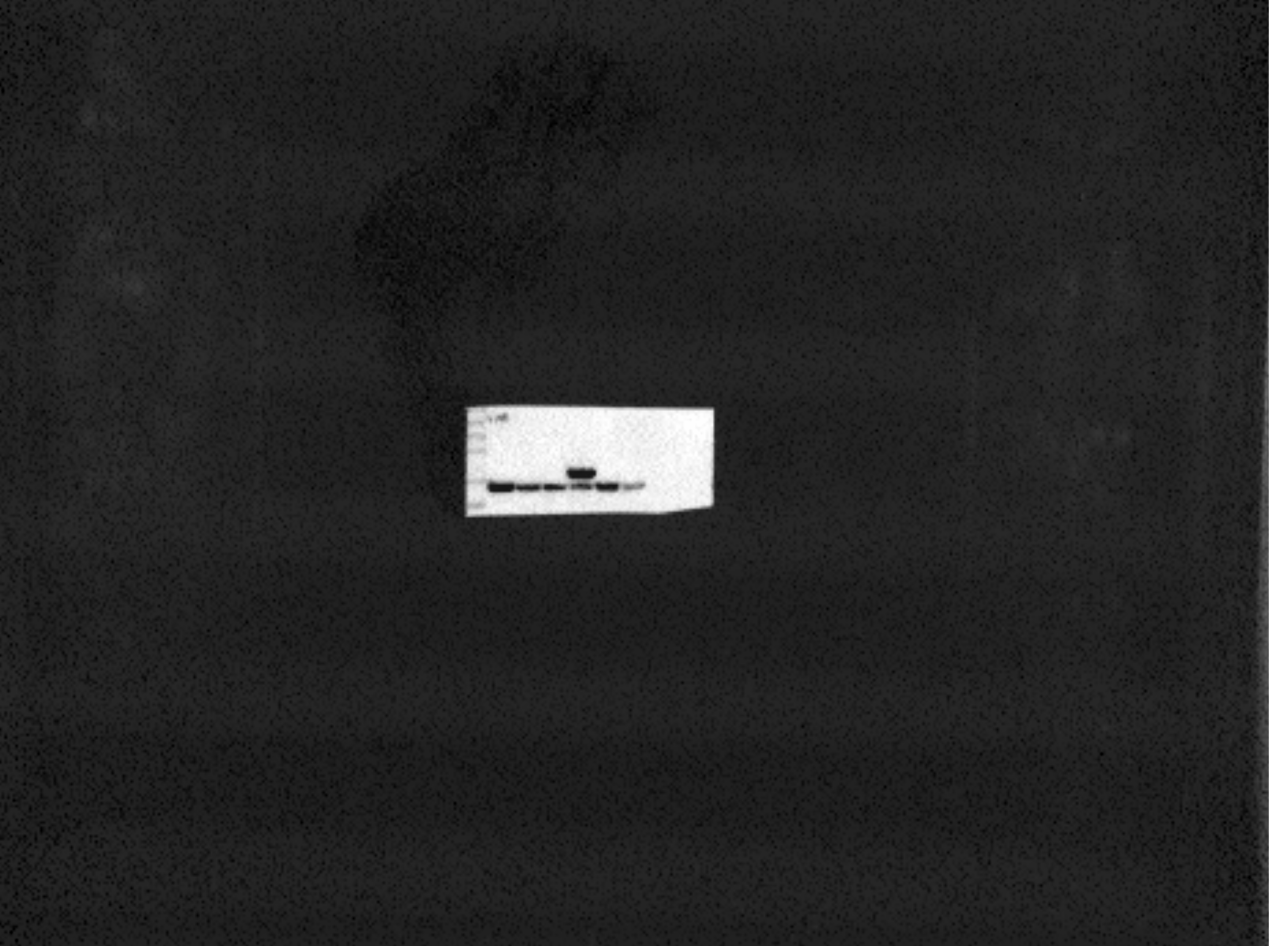


Fig 2A-α-tublin


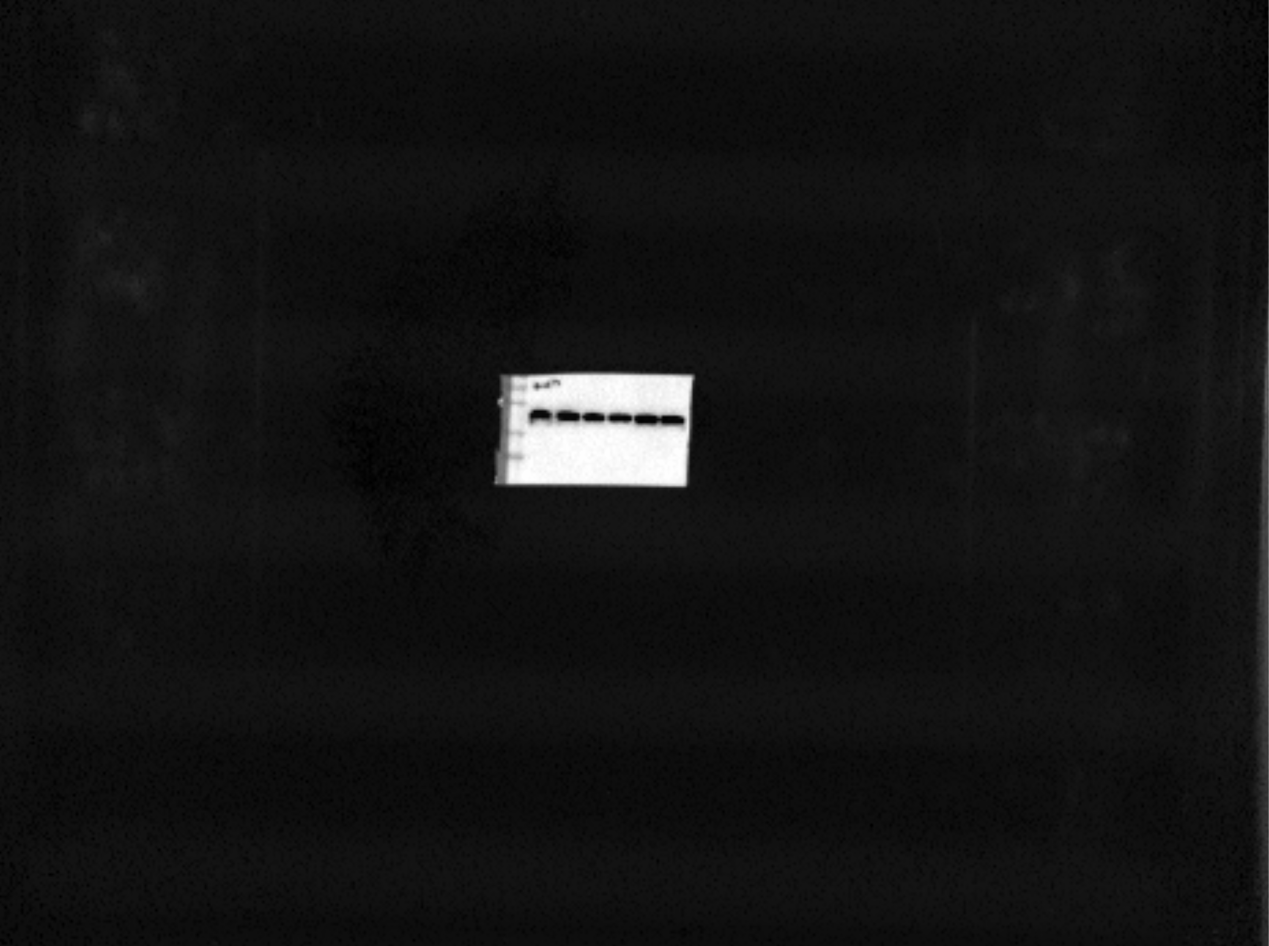


Fig 2E-TMEM43


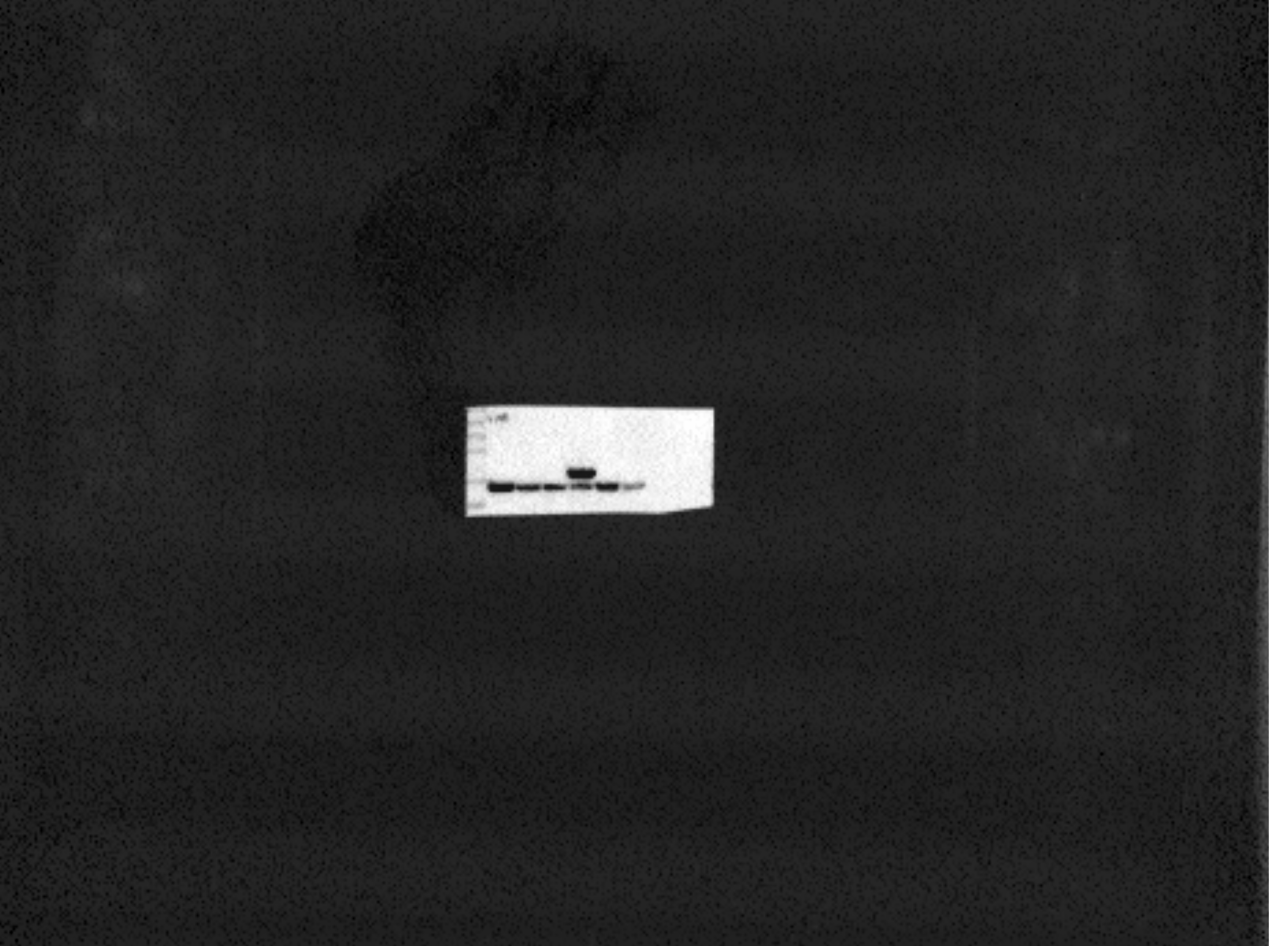


Fig2E-α-tublin


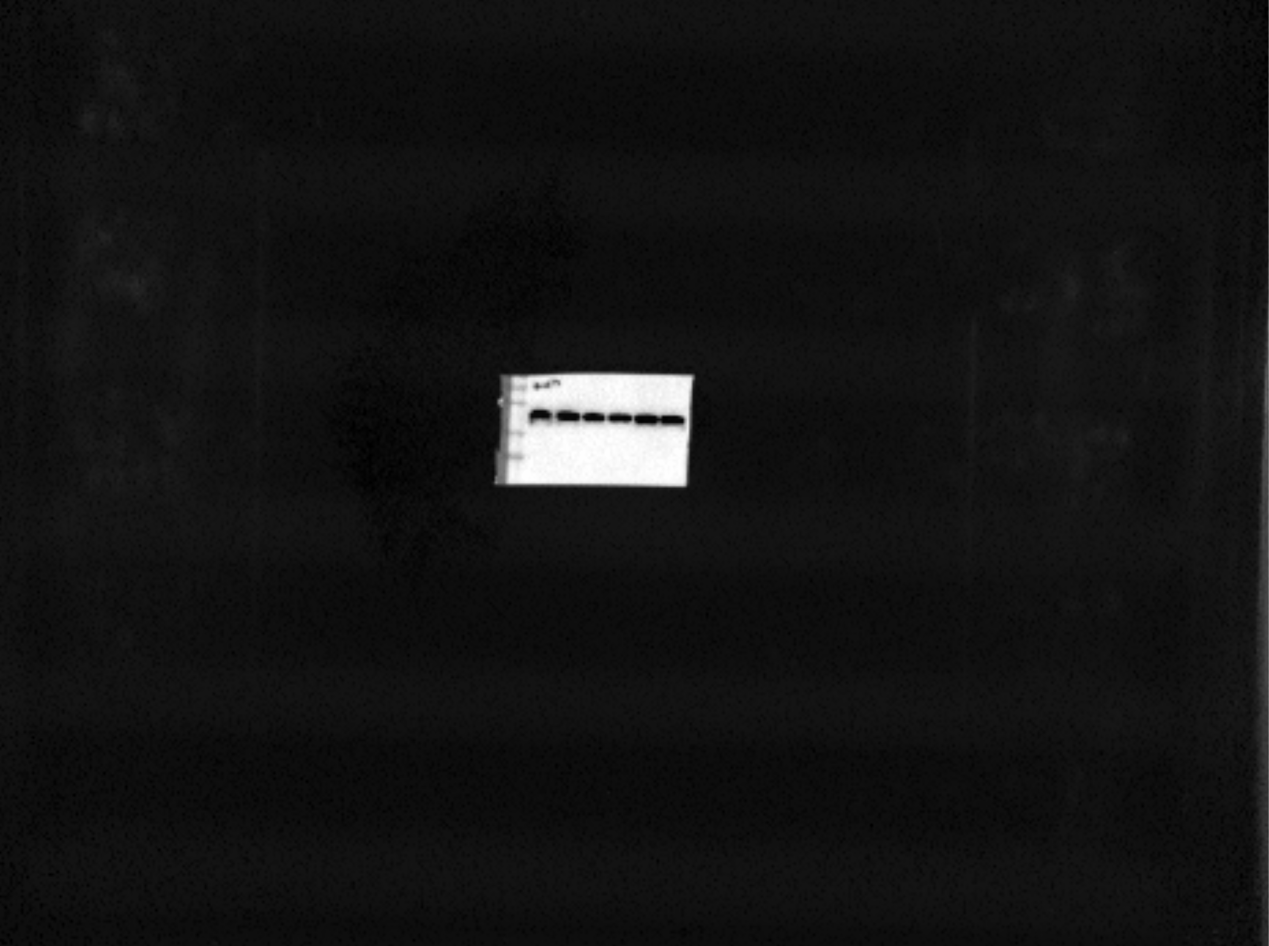


Fig 2F-TMEM43


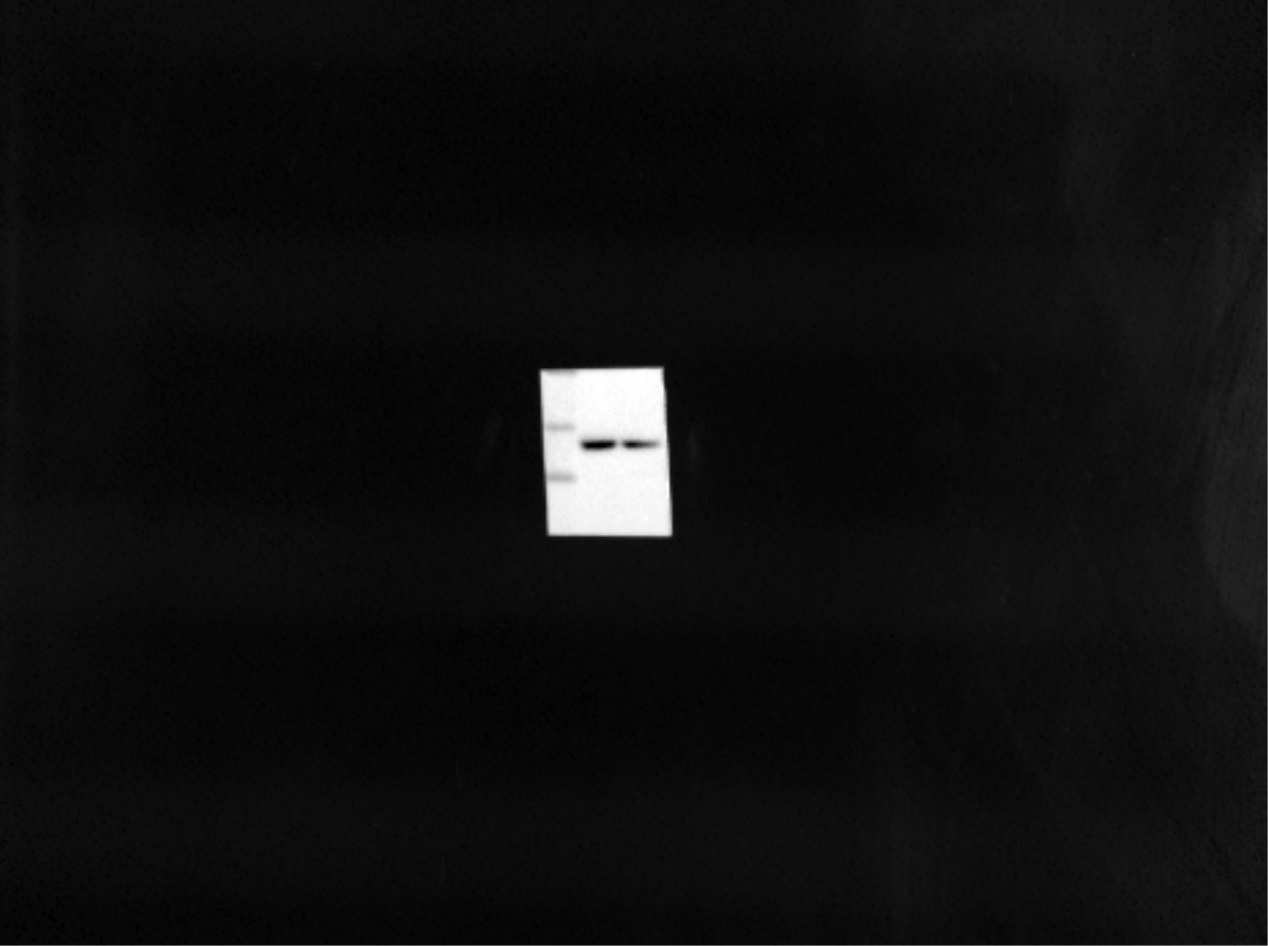


Fig 2F-α-tublin


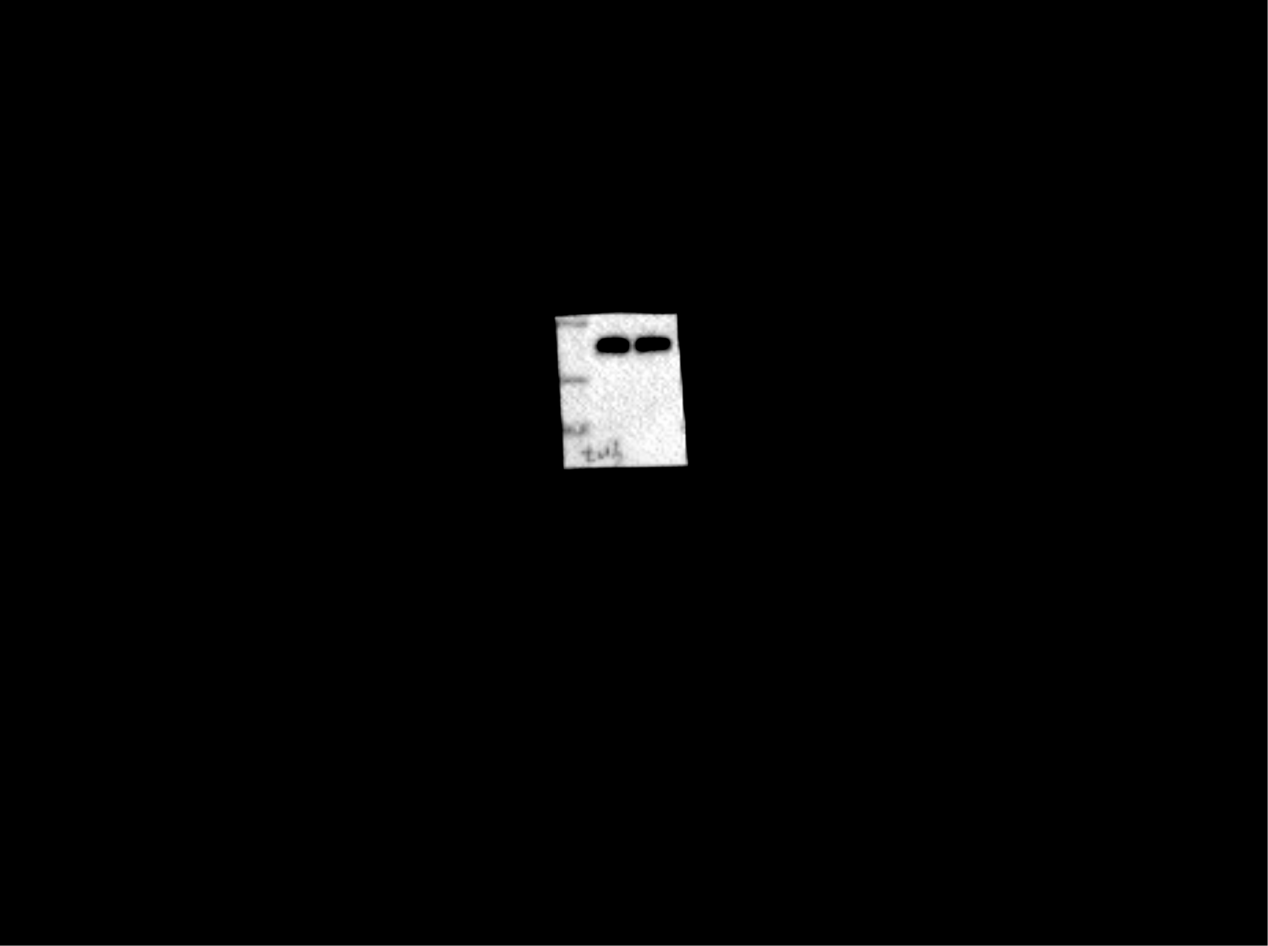


Fig 3B (MIAPaCa-2, SW1990)-TMEM43


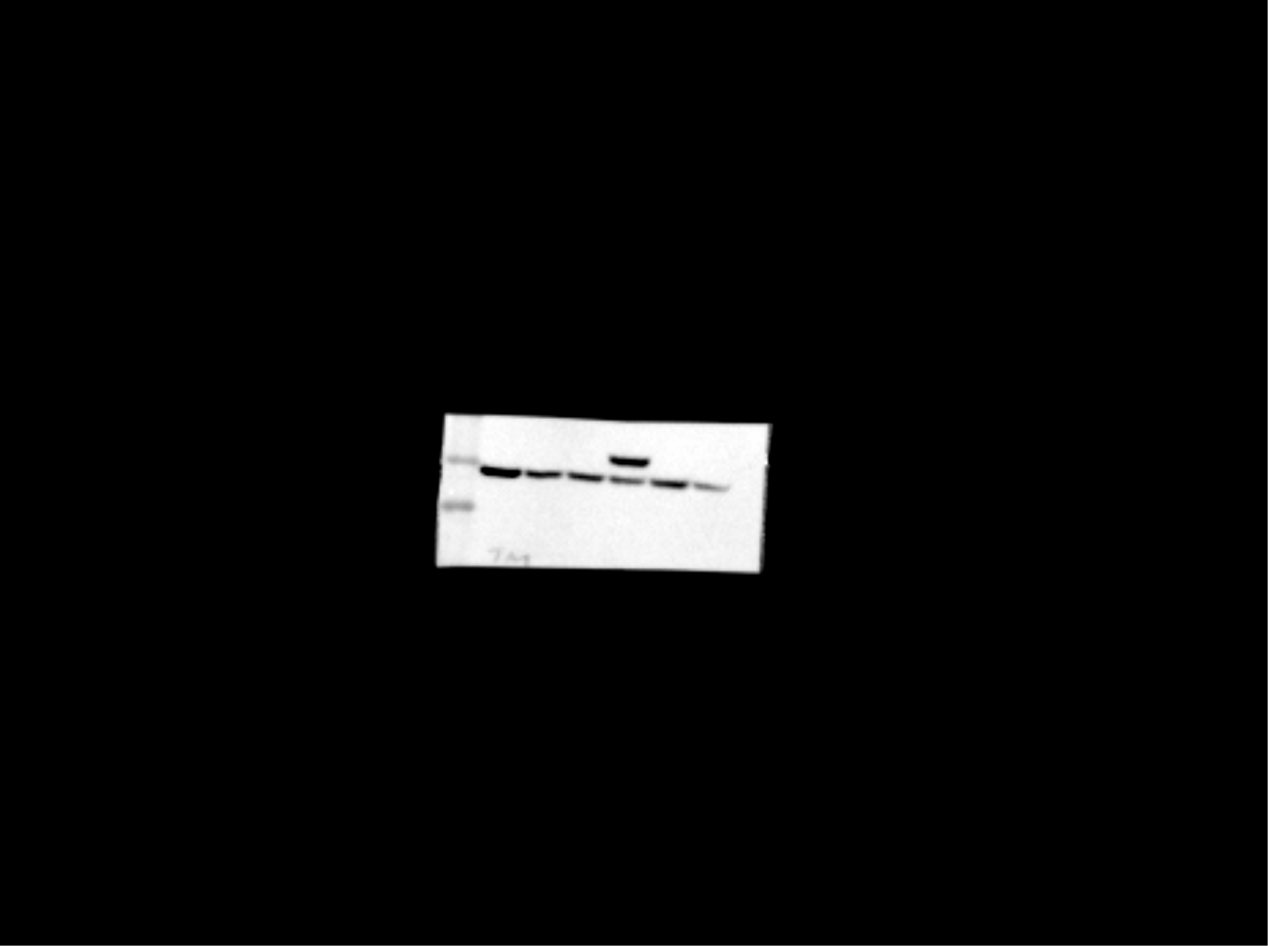


Fig 3B (MIAPaCa-2, SW1990)-RAP2B


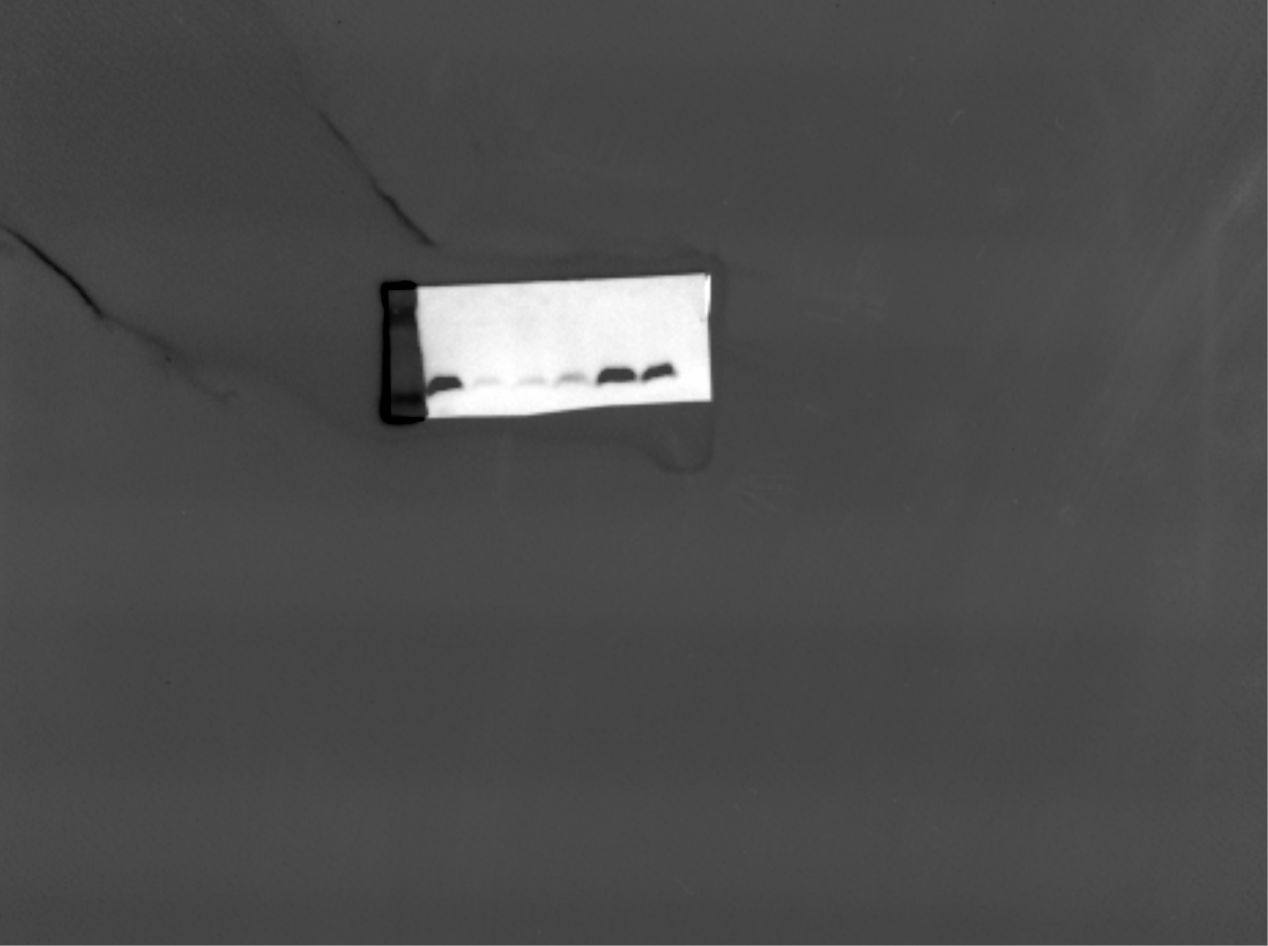


Fig 3B (MIAPaCa-2, SW1990)-pERK


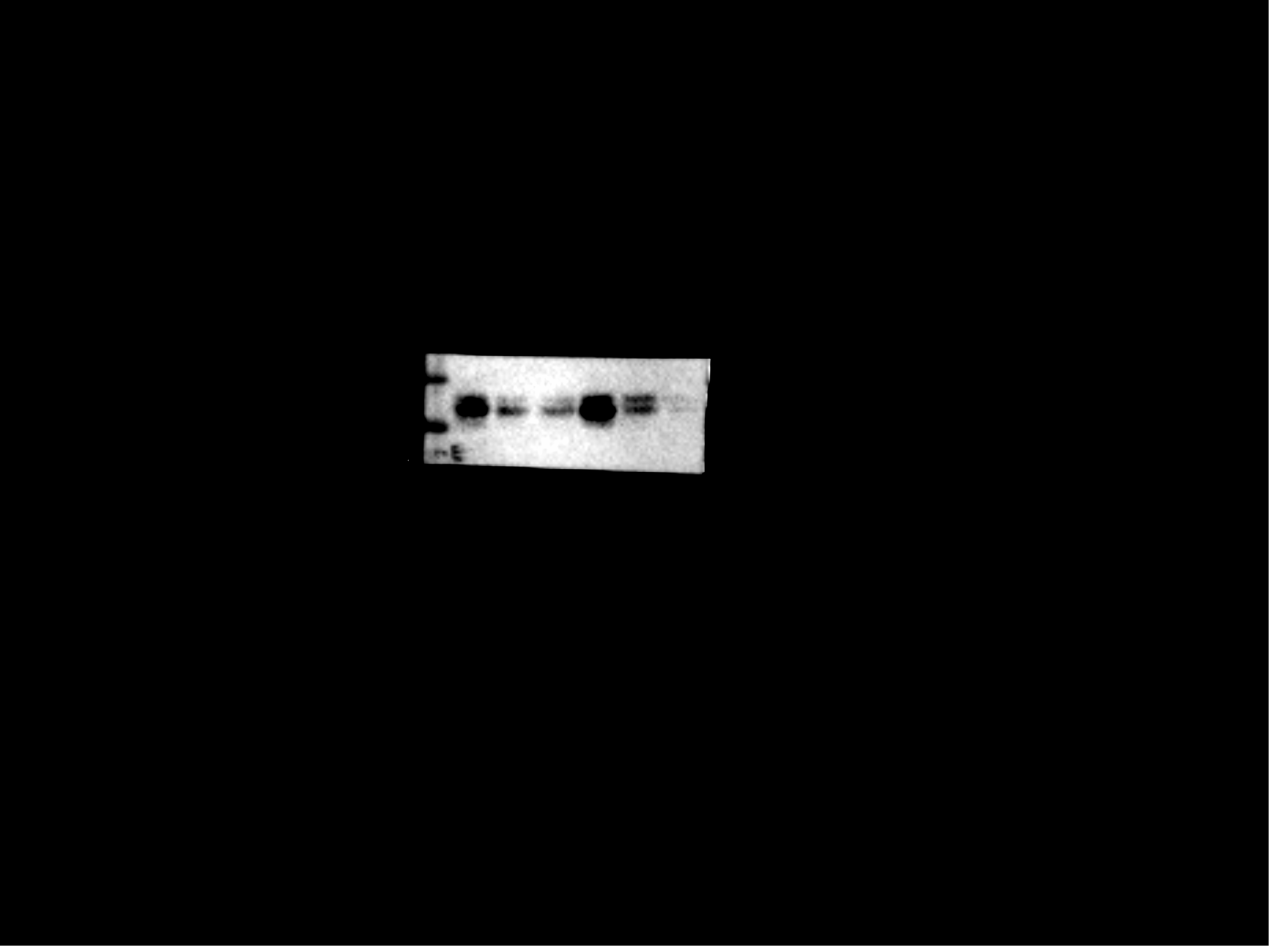


Fig 3B (MIAPaCa-2, SW1990)-ERK


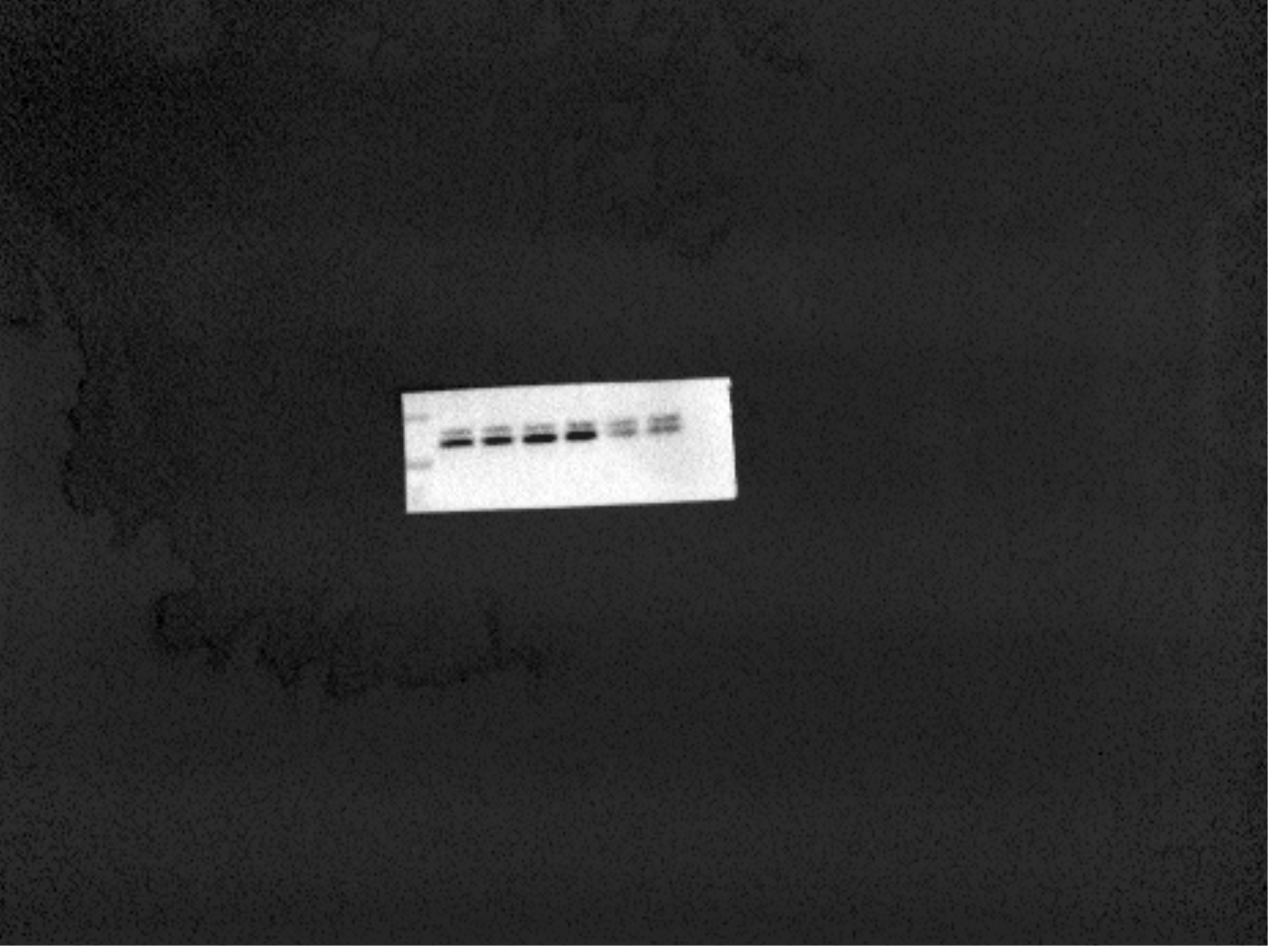


Fig 3B (MIAPaCa-2, SW1990)-α-tublin


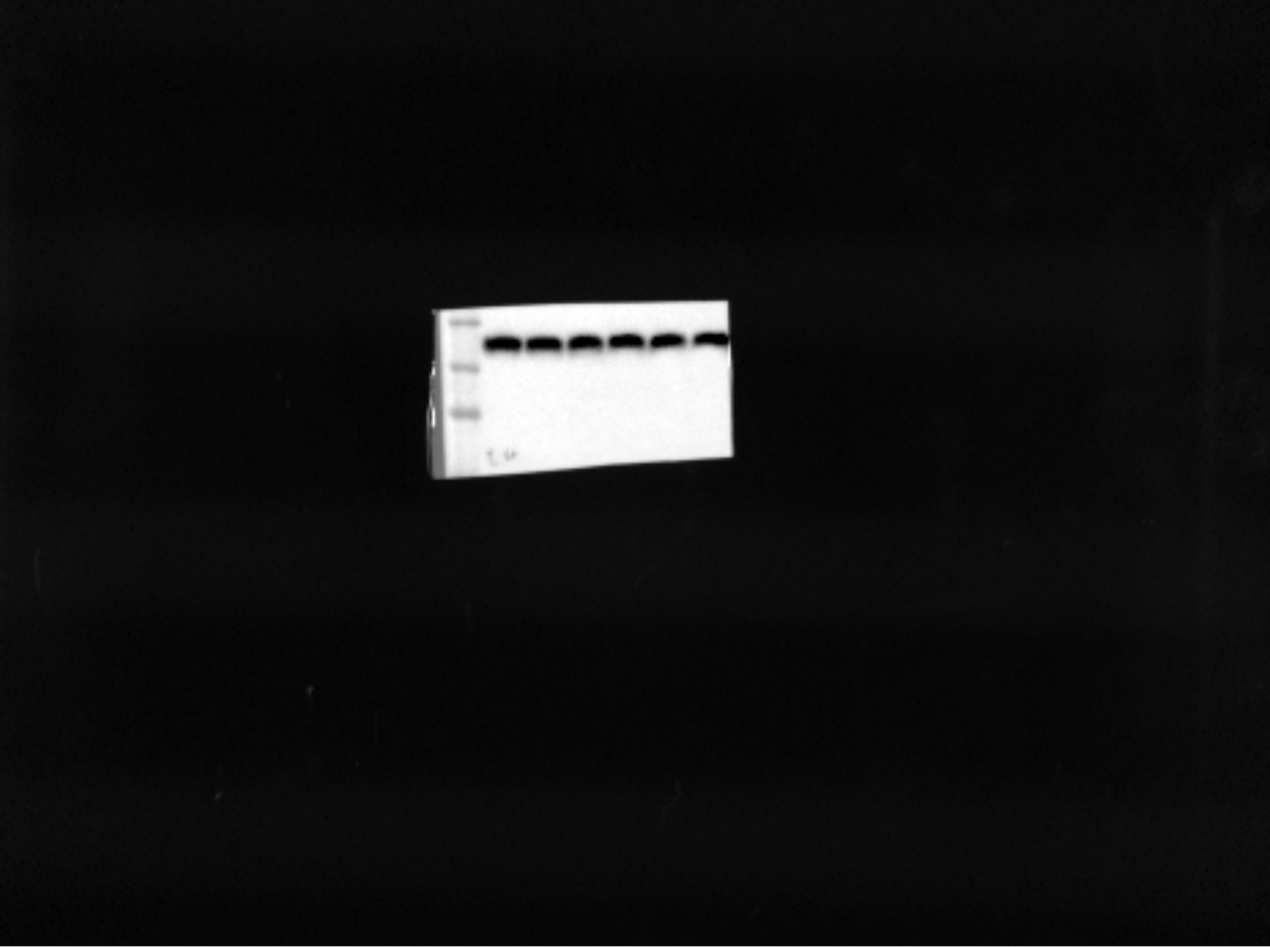


Fig 3B (Capan-2)-TMEM43


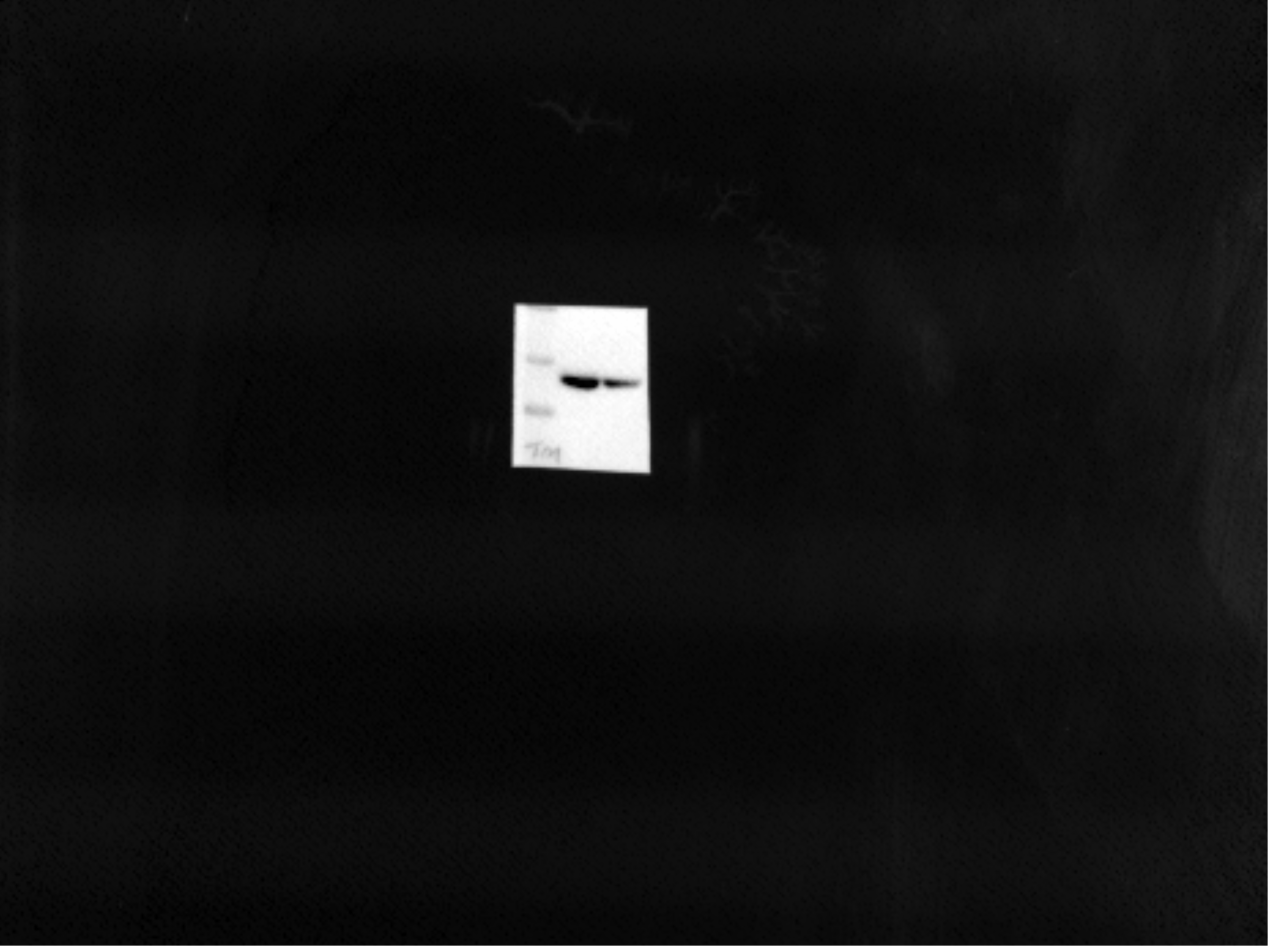


Fig 3B (Capan-2)-RAP2B


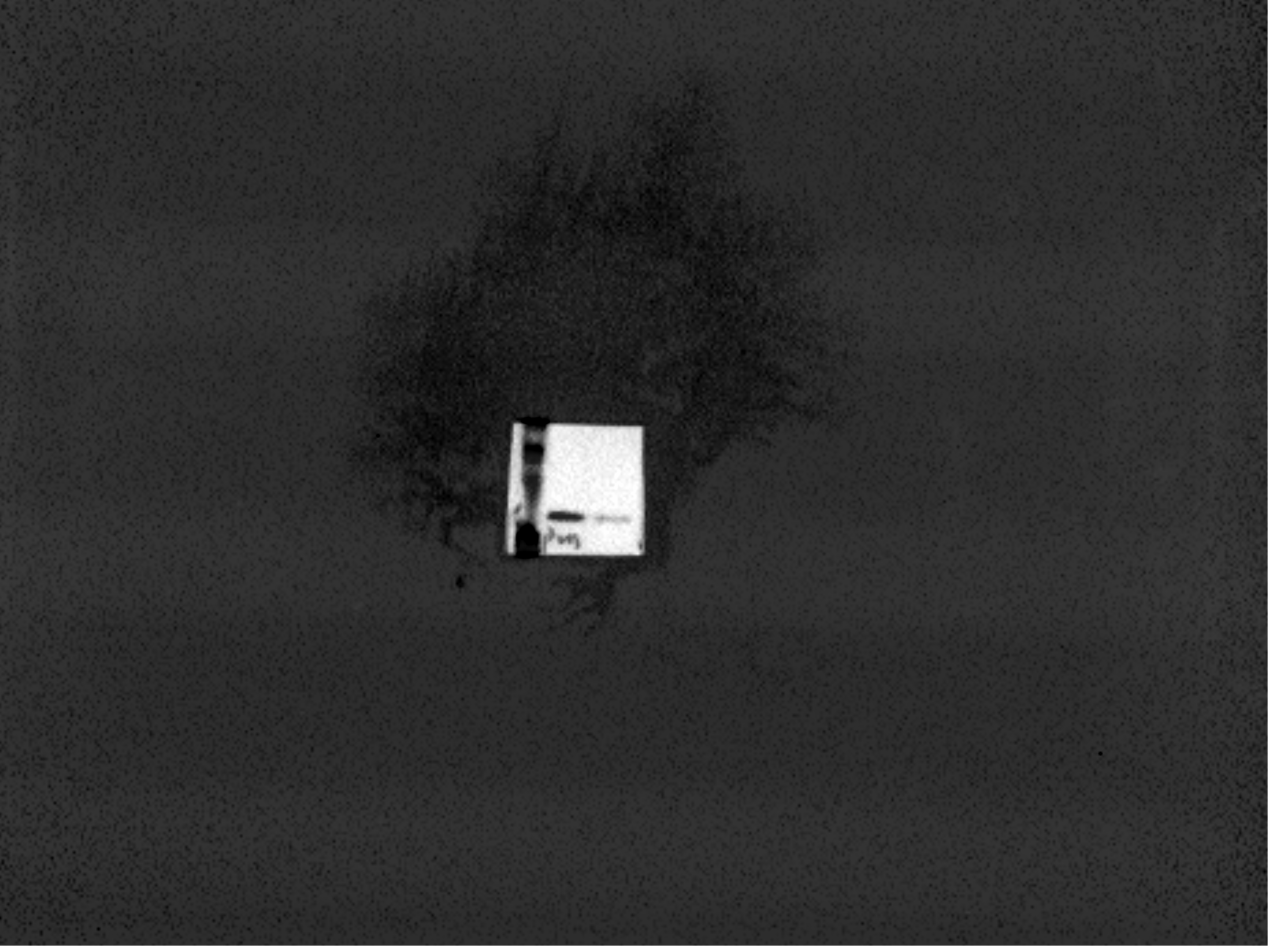


Fig 3B (Capan-2)-p-ERK


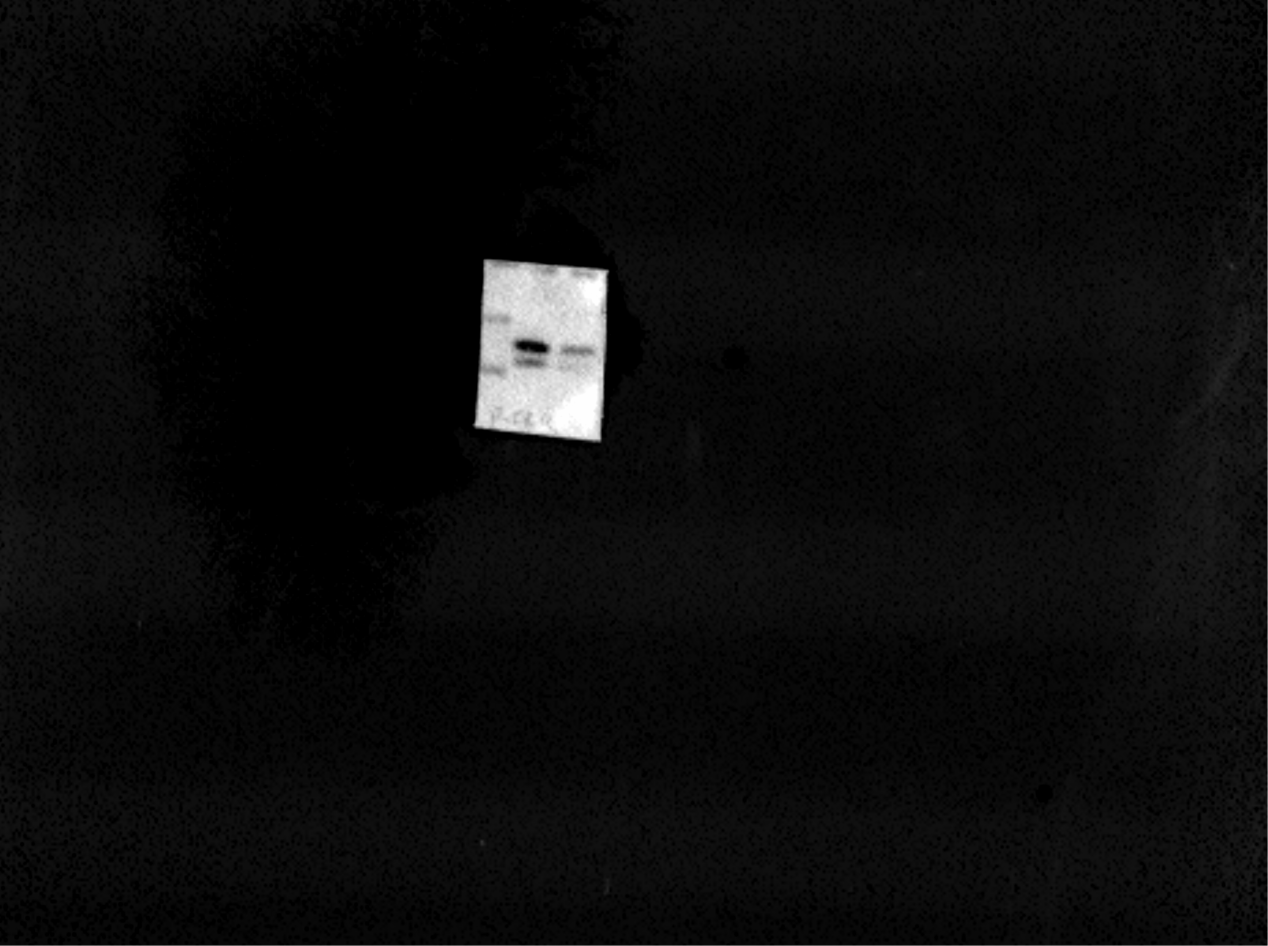


Fig 3B (Capan-2)-ERK


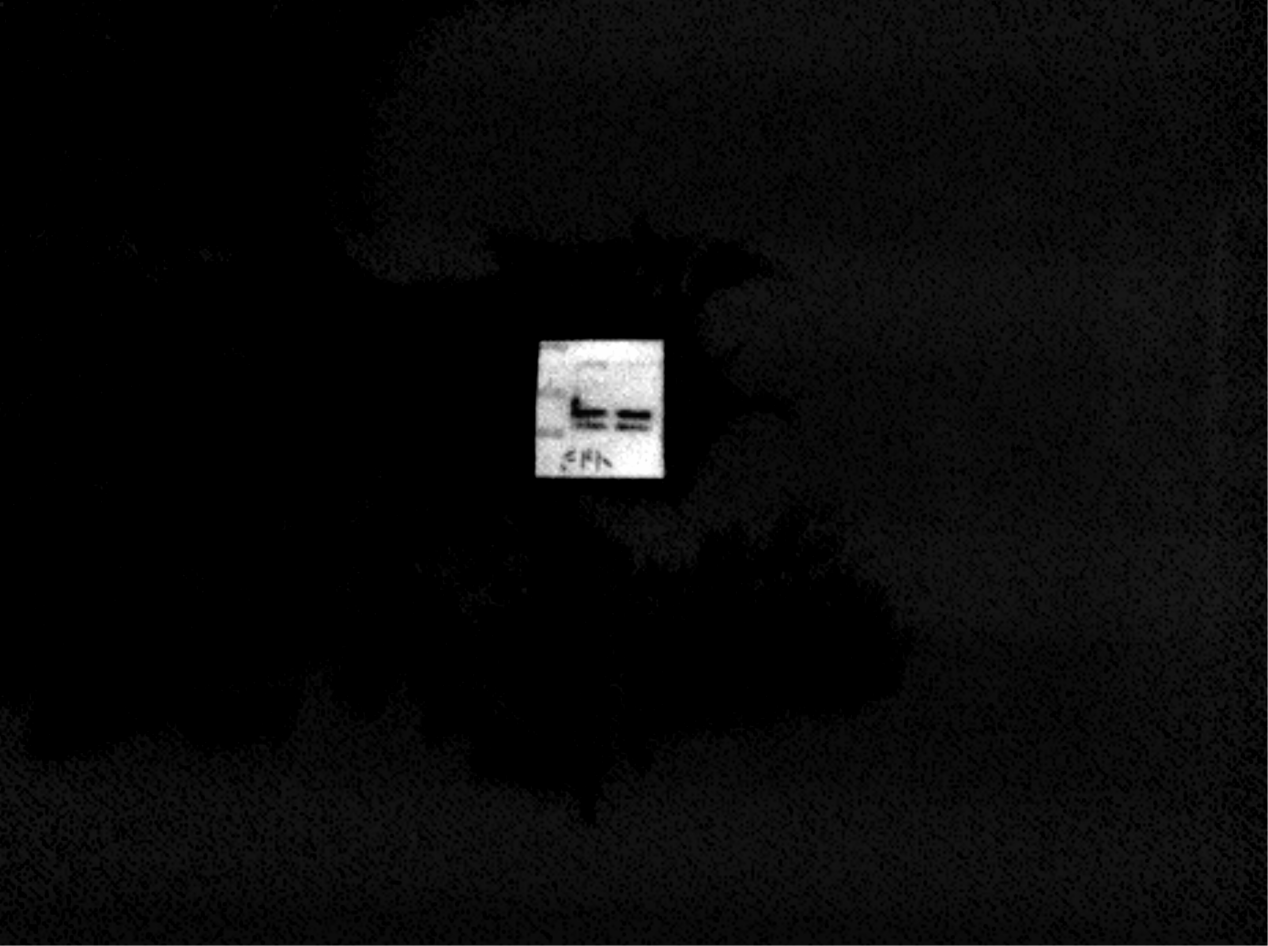


Fig 3B (Capan-2)-α-tublin


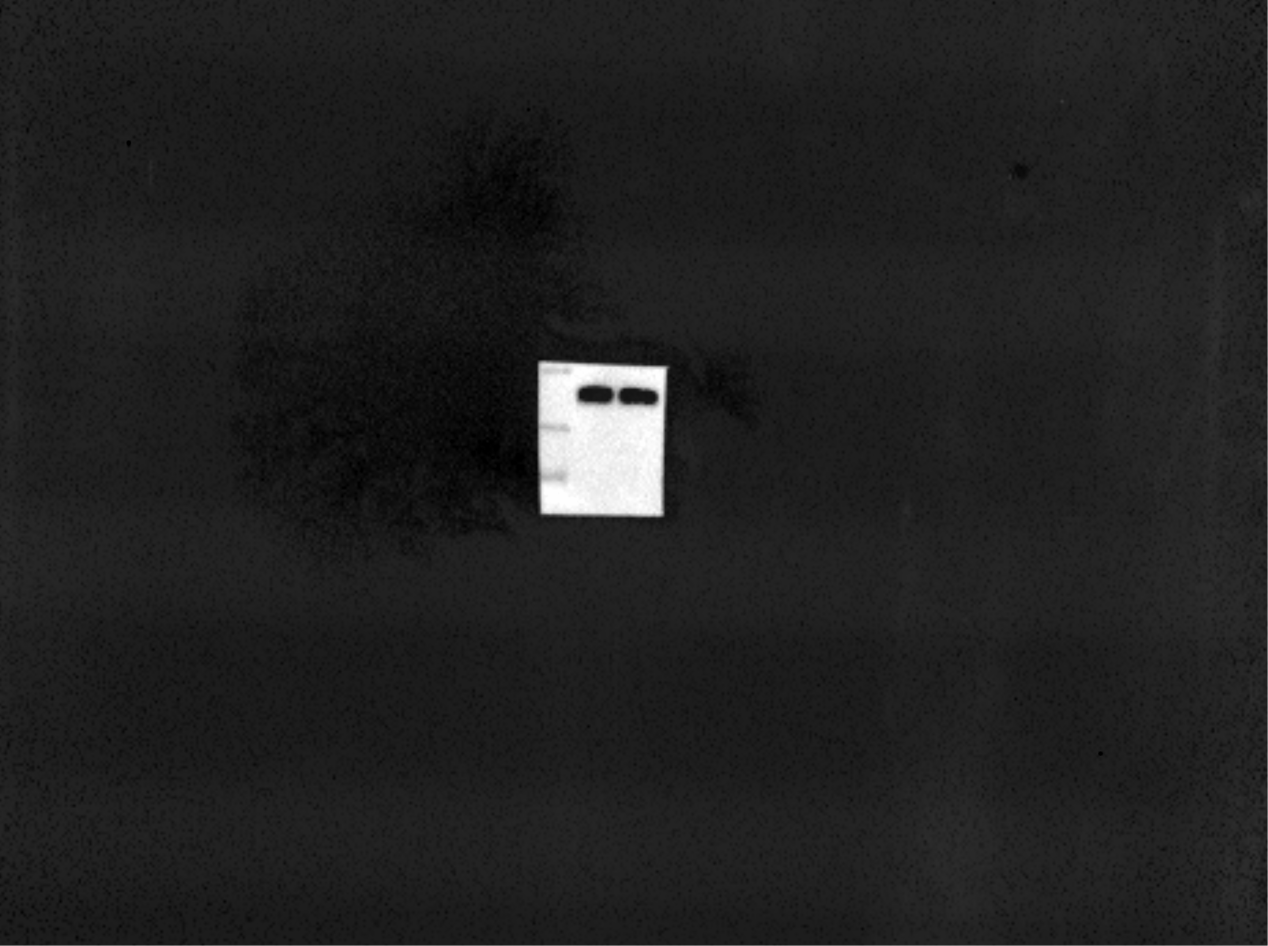


Fig 3C (MIAPaCa-2)-RAP2B


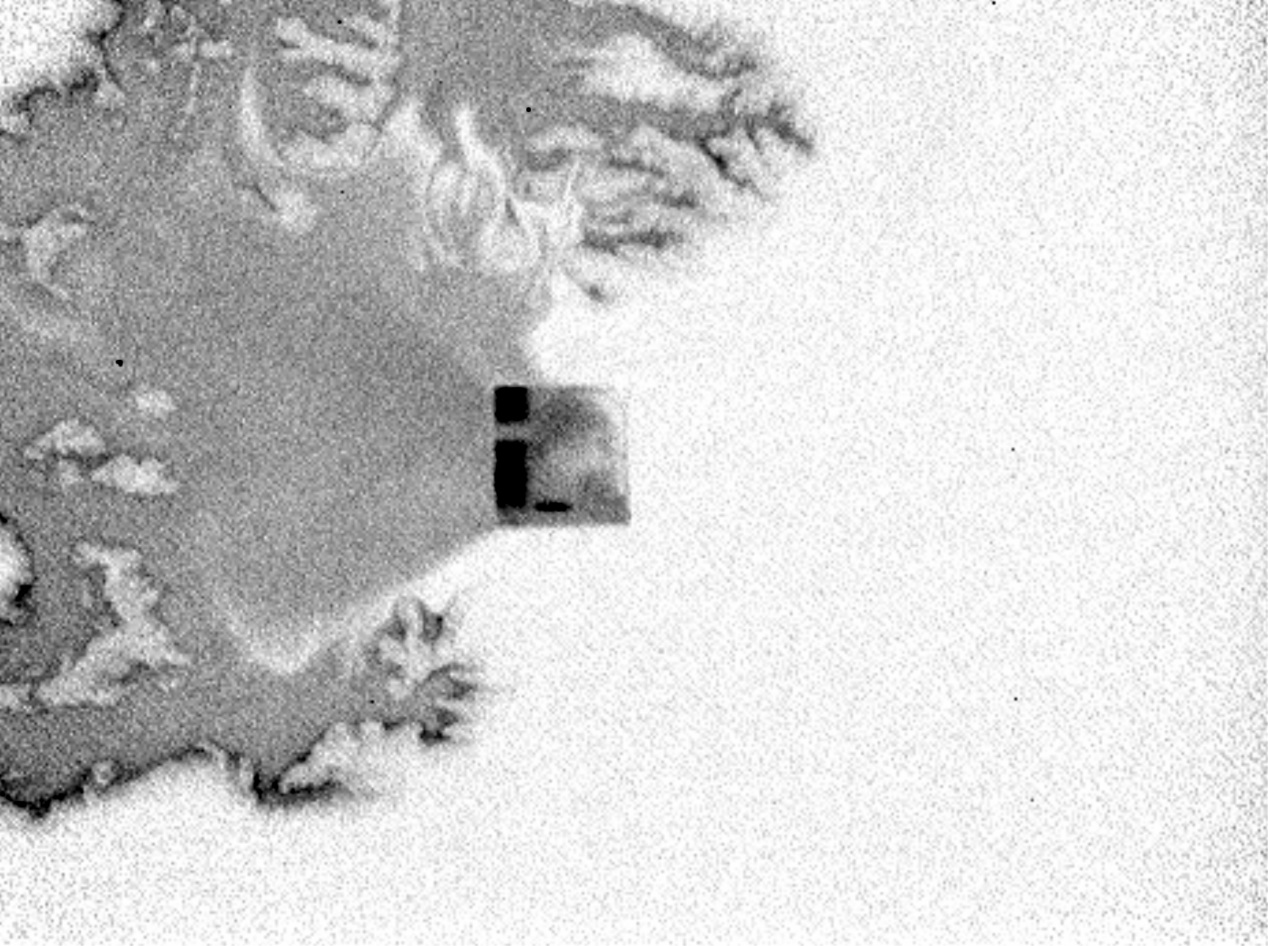


Fig 3C (MIAPaCa-2)-p-ERK


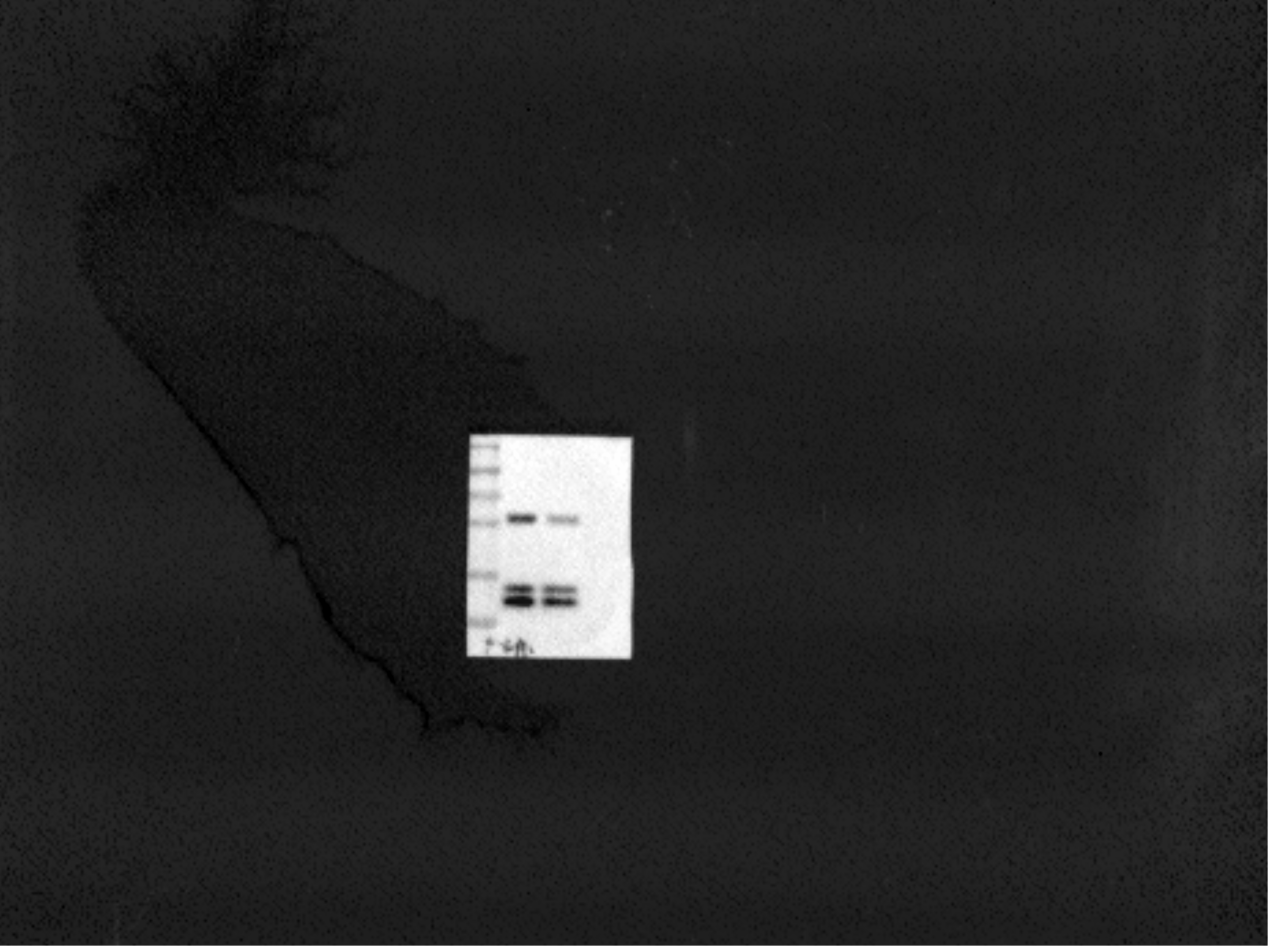


Fig 3C (MIAPaCa-2)-ERK


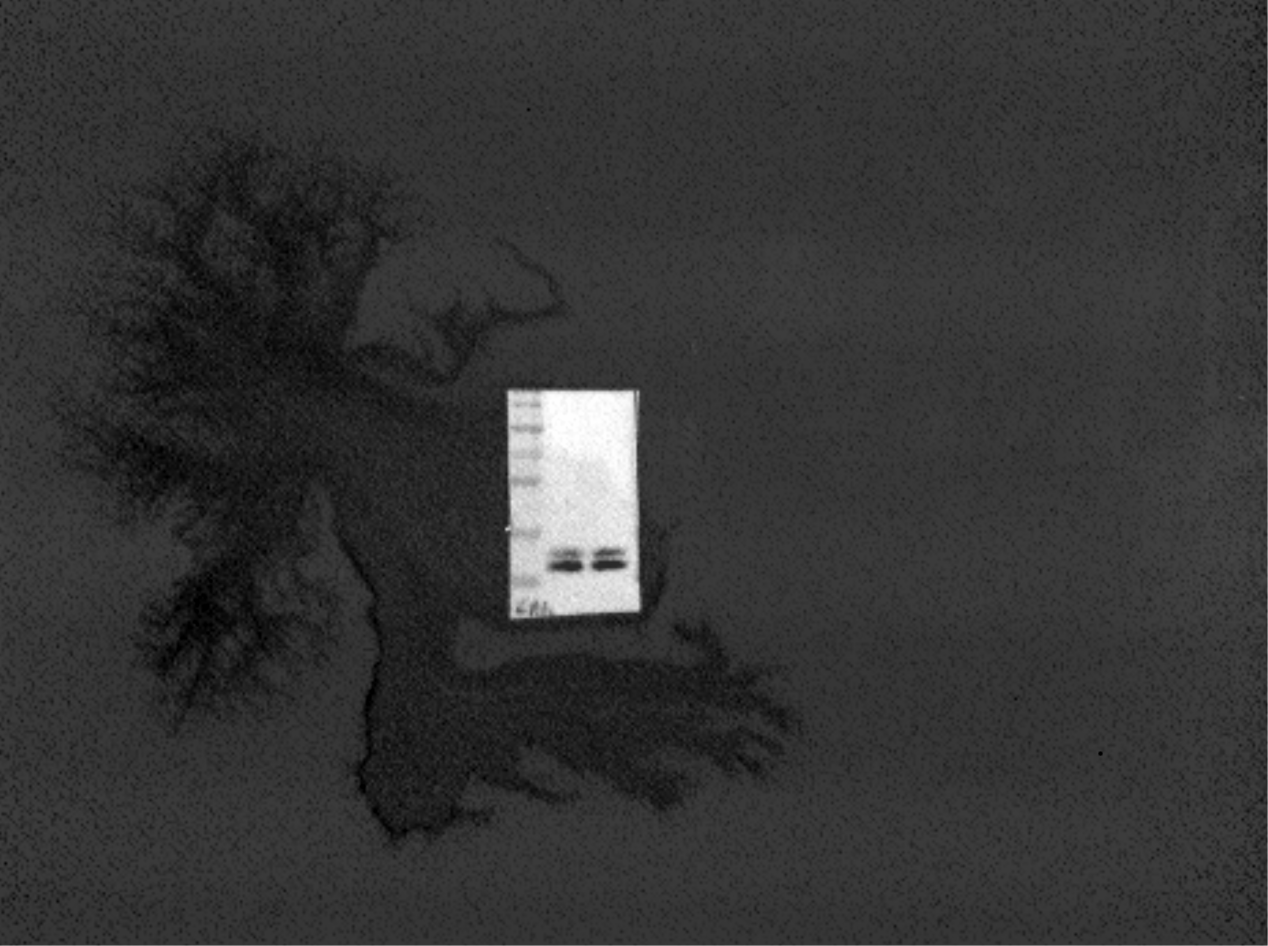


Fig 3C (MIAPaCa-2)-α-tublin


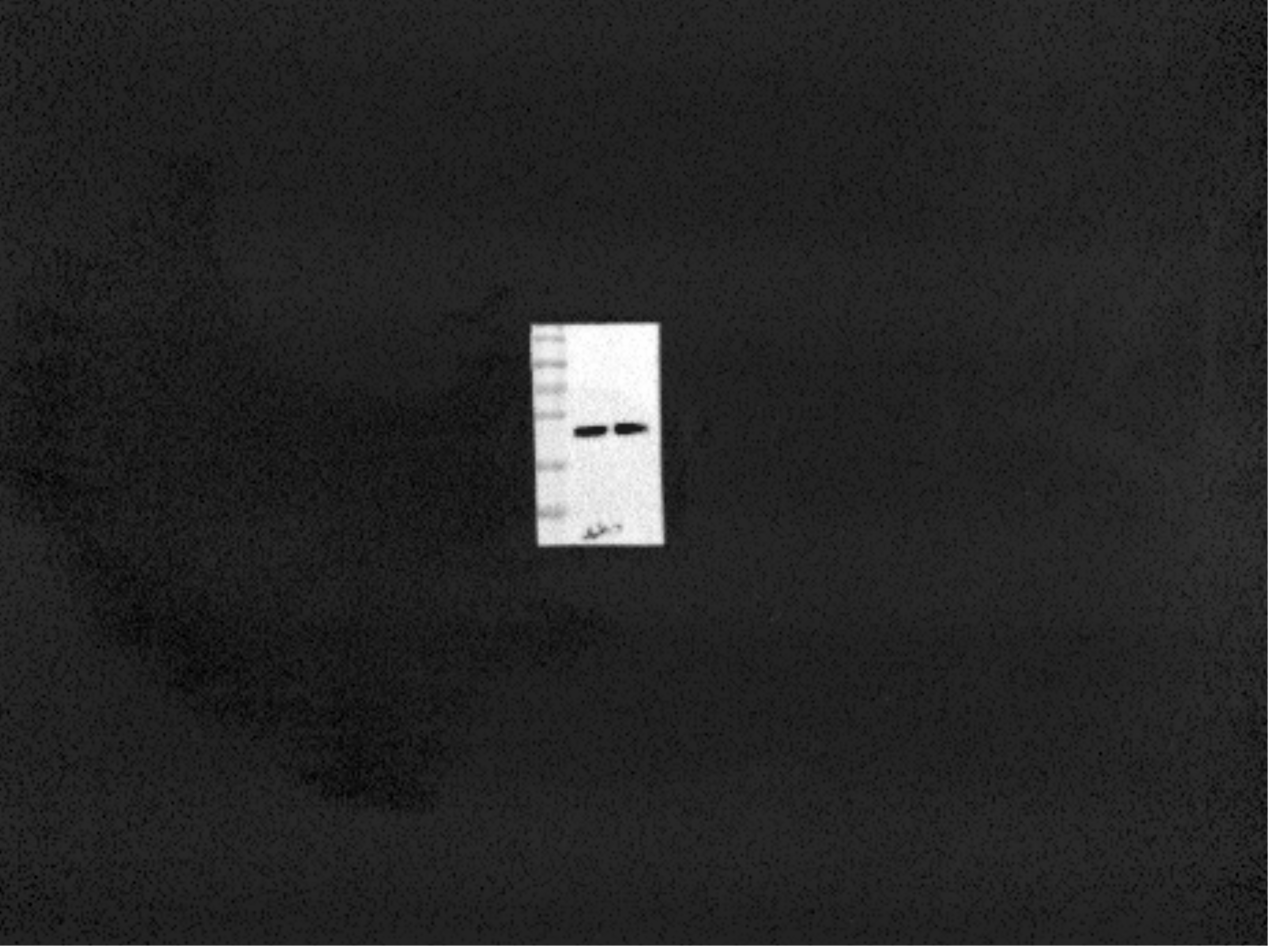


Fig 3C (SW1990)-RAP2B


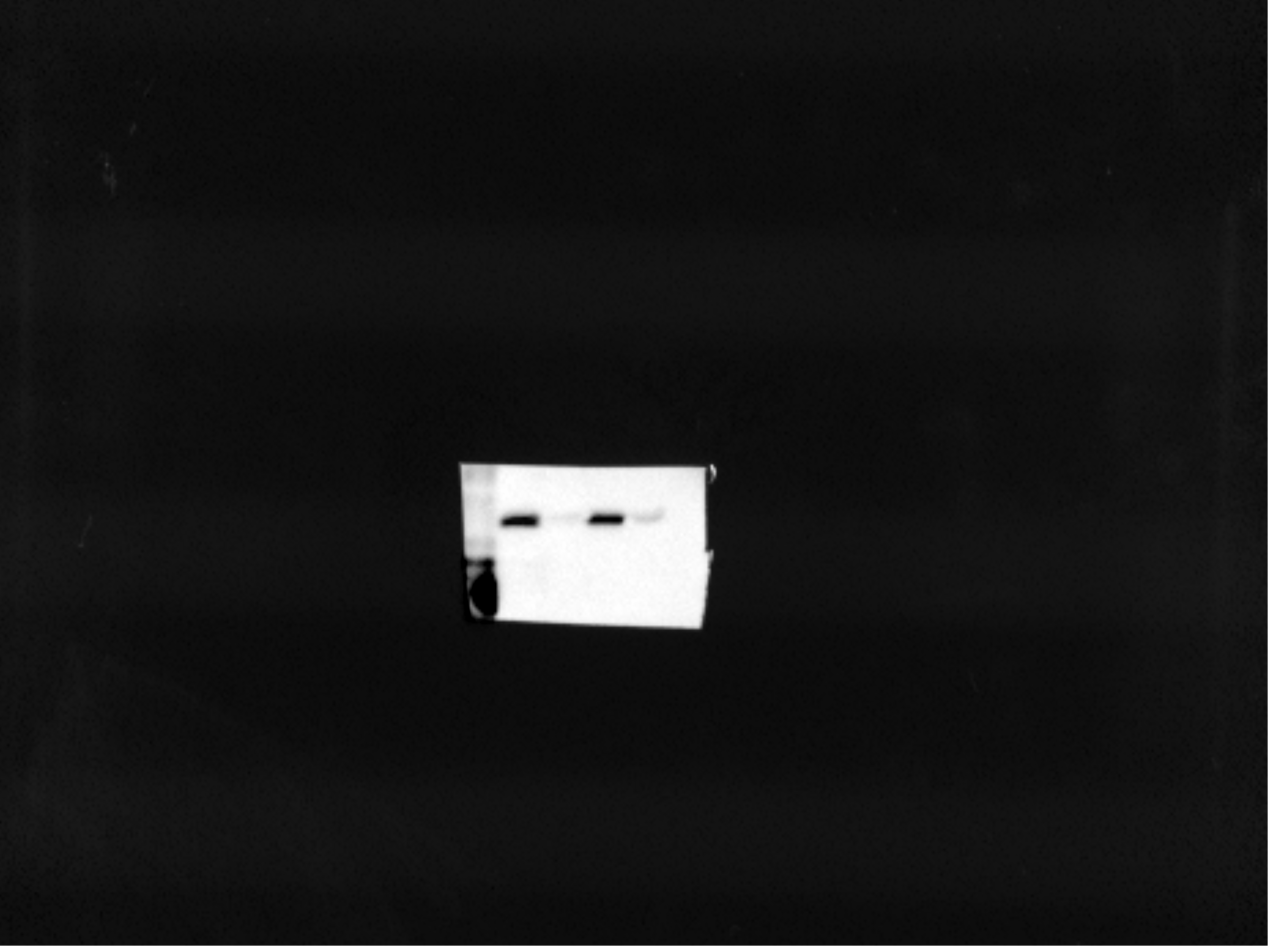


Fig 3C (SW1990)-p-ERK


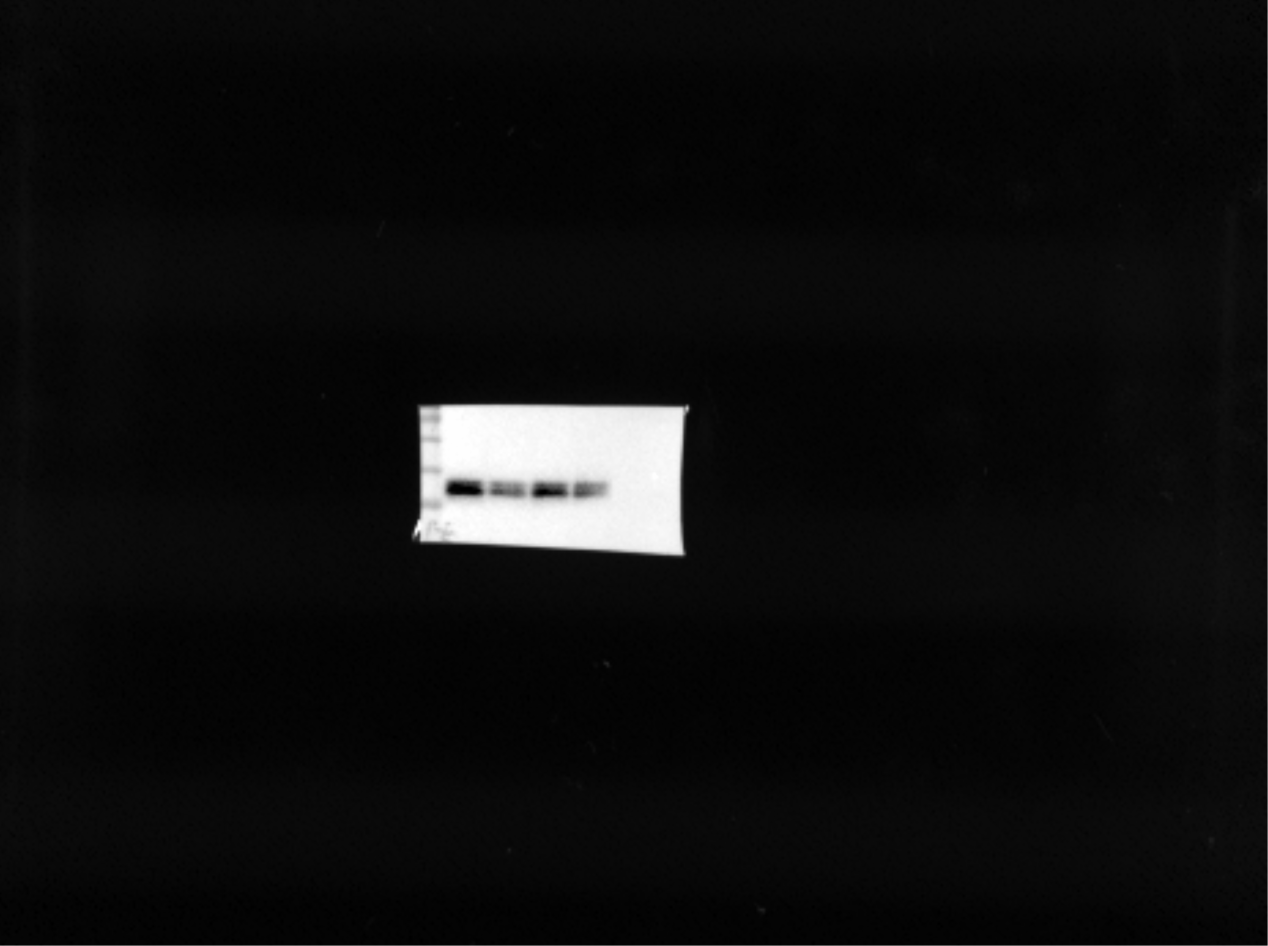


Fig 3C (SW1990)-ERK


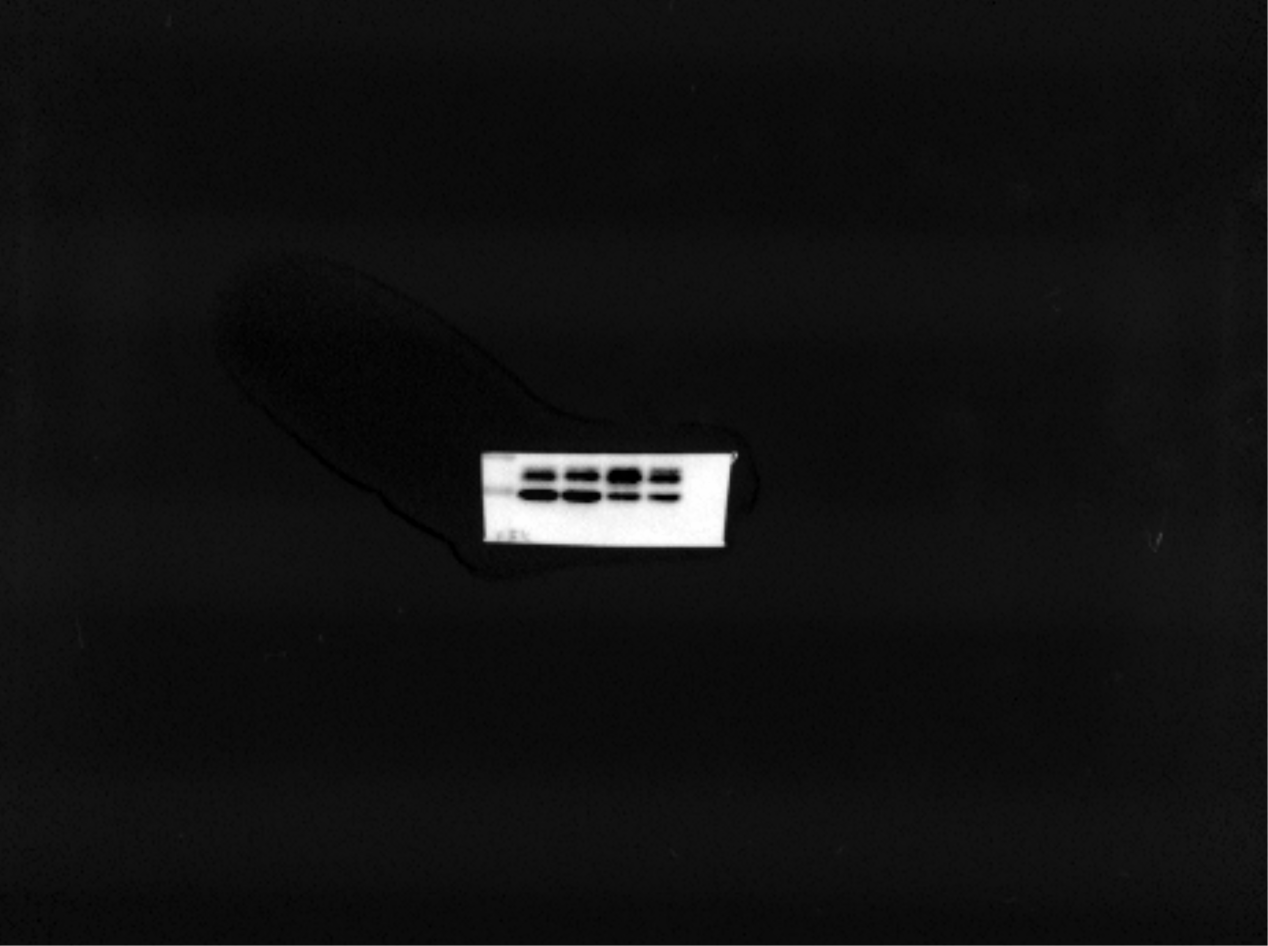


α-tublinα-tublin

Fig 3C (SW1990)-α-tublin


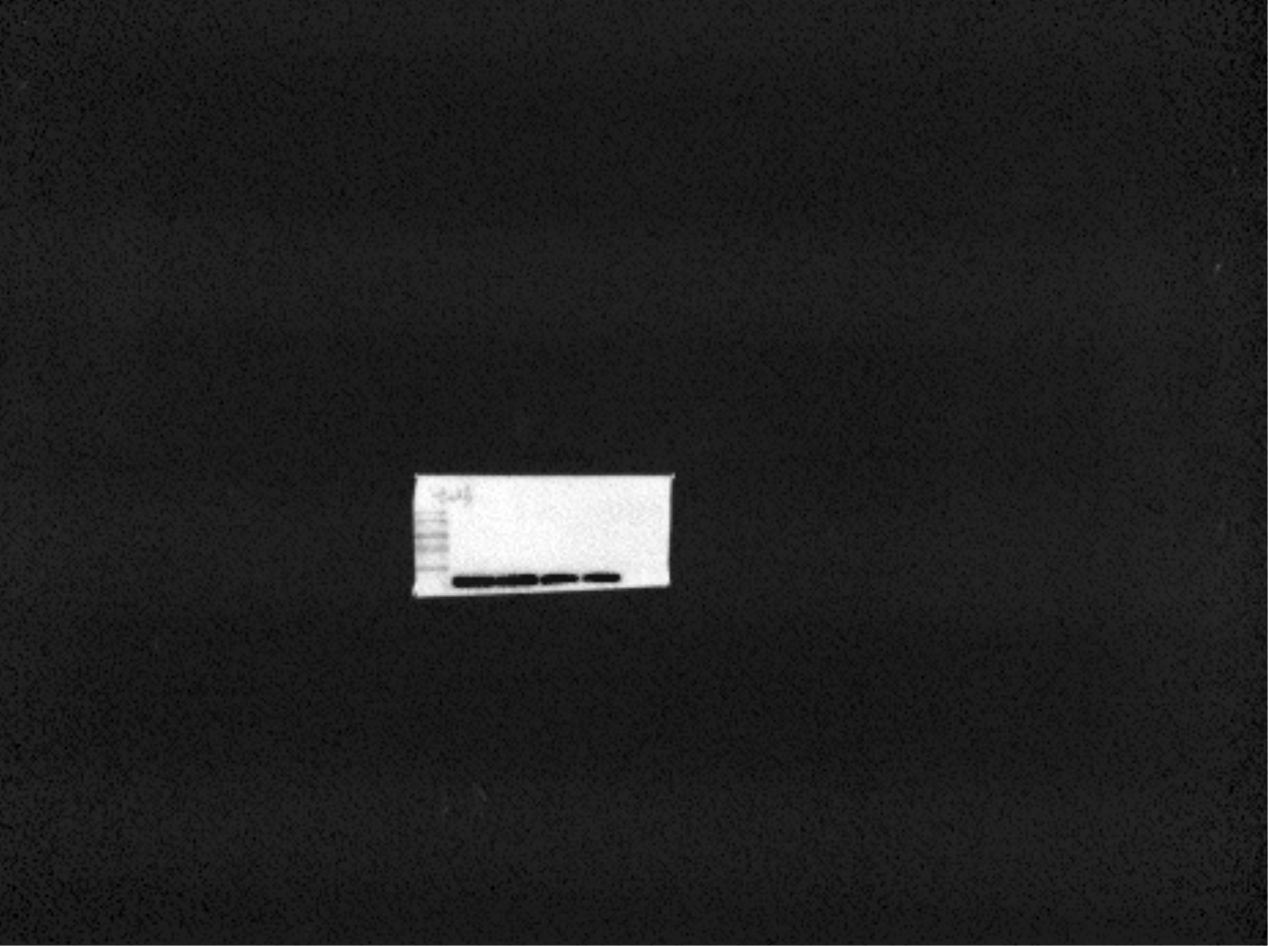


α-tublinα-tublin

Fig 3L-TMEM43


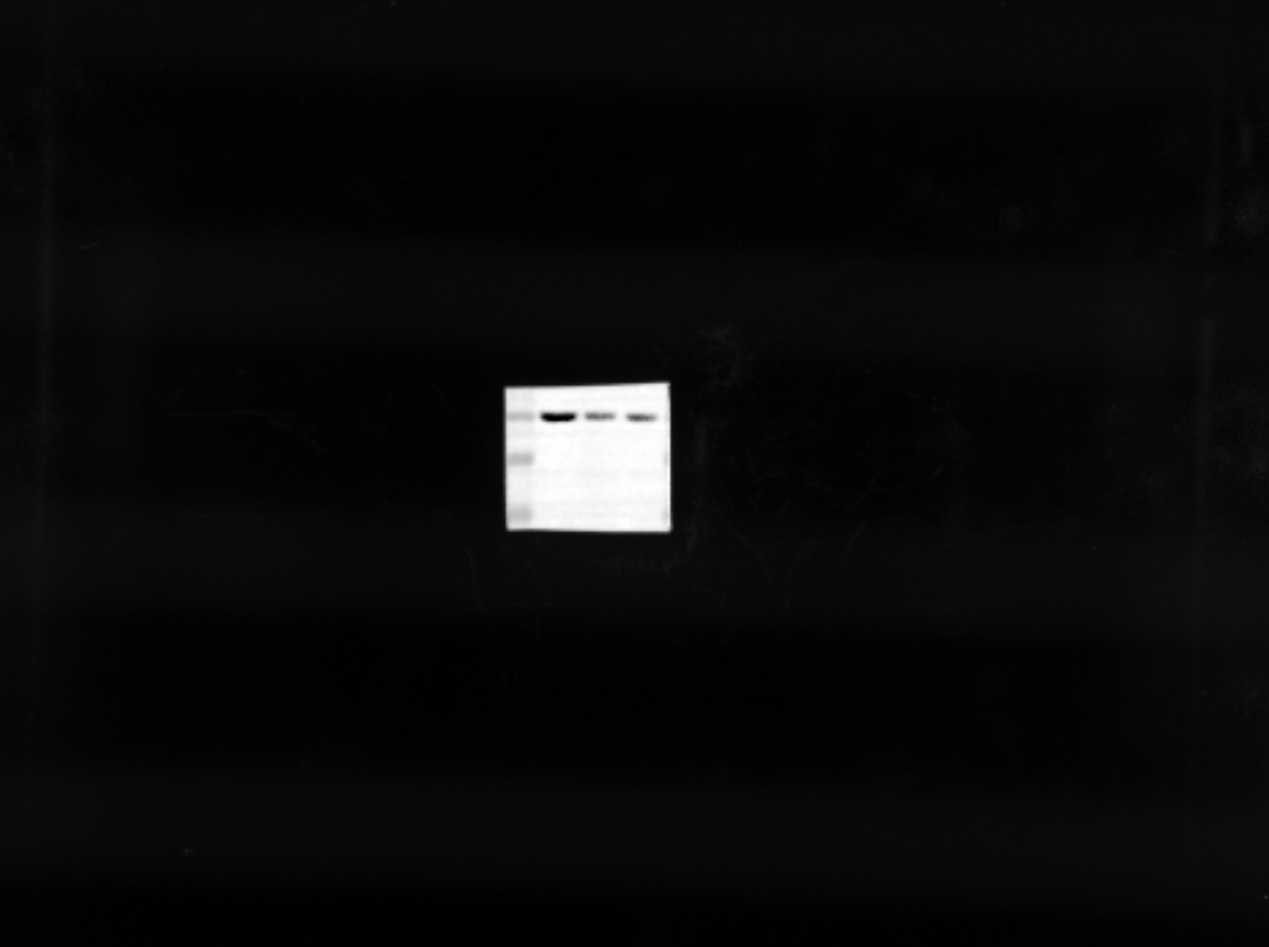


Fig 3L-RAP2B


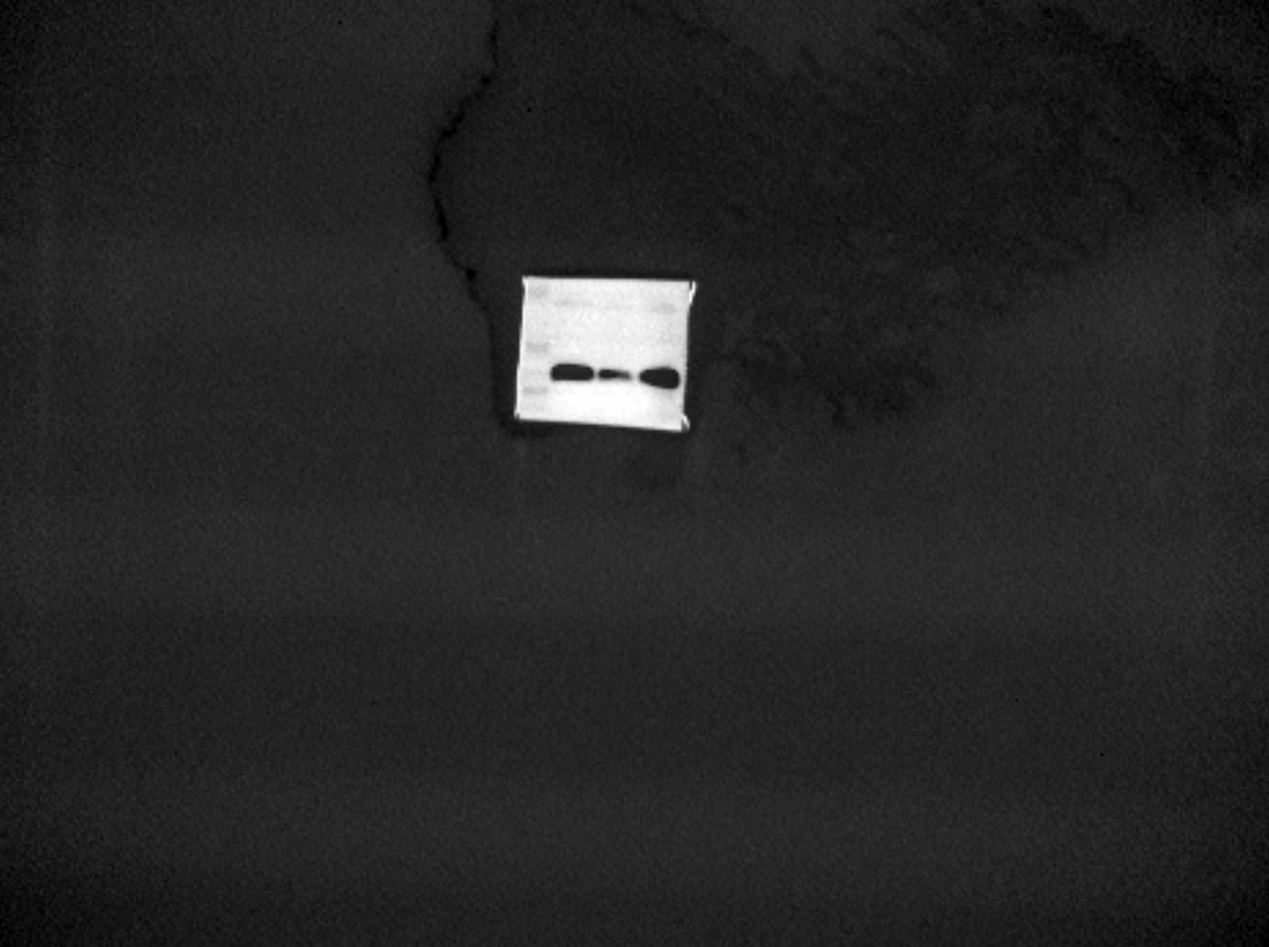


Fig 3L-p-ERK


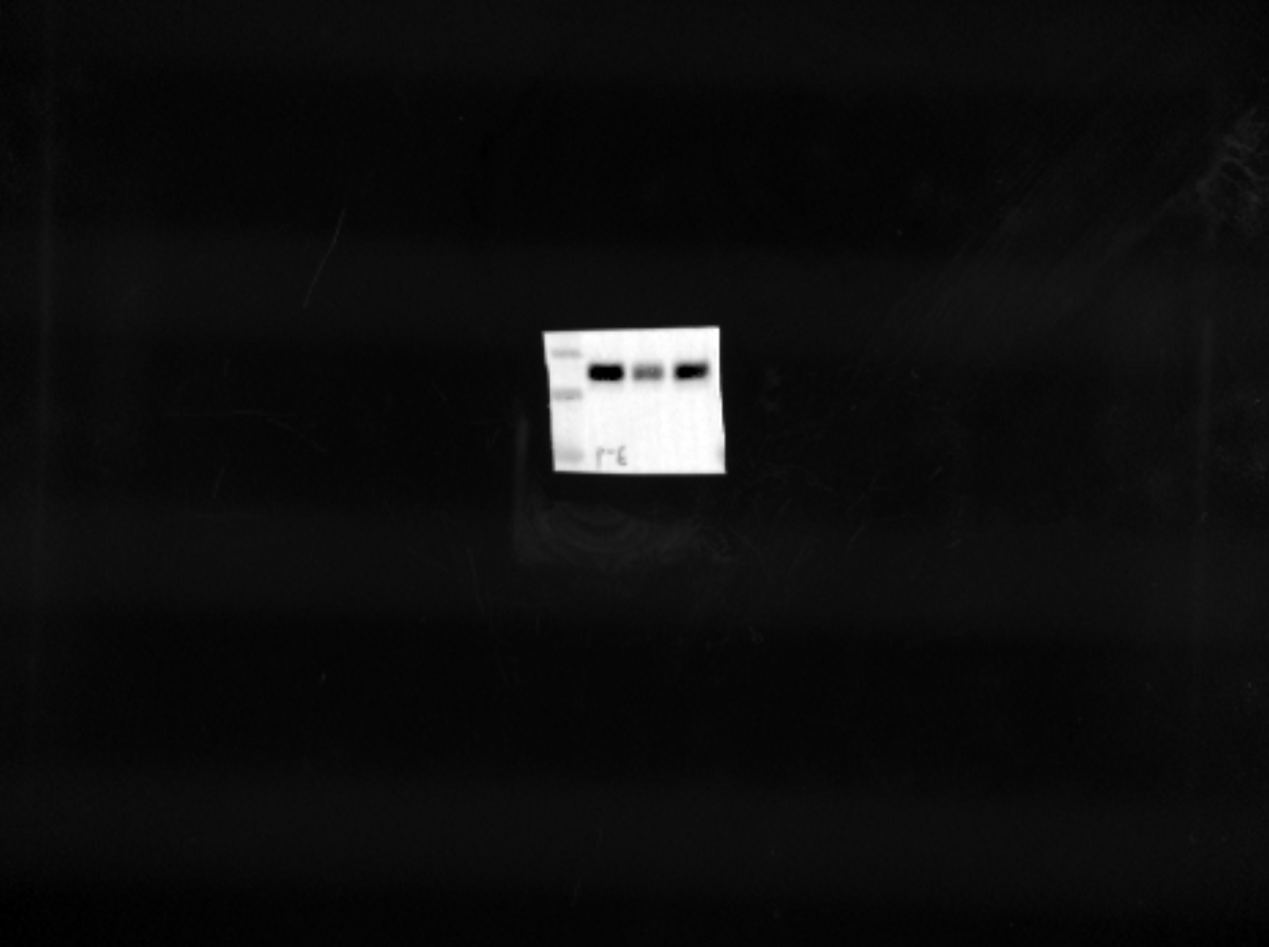


Fig 3L-ERK


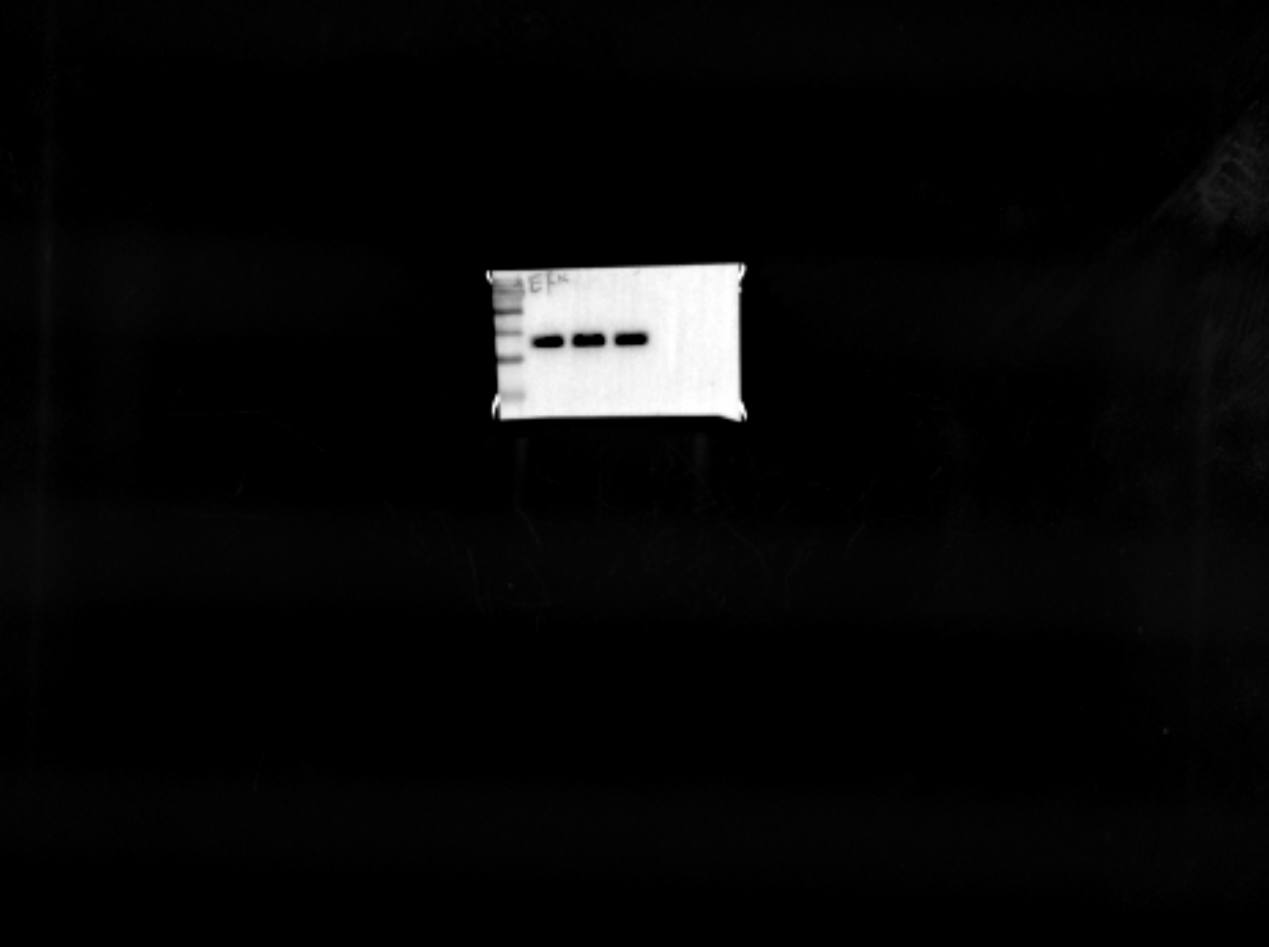


Fig 3L-tublin


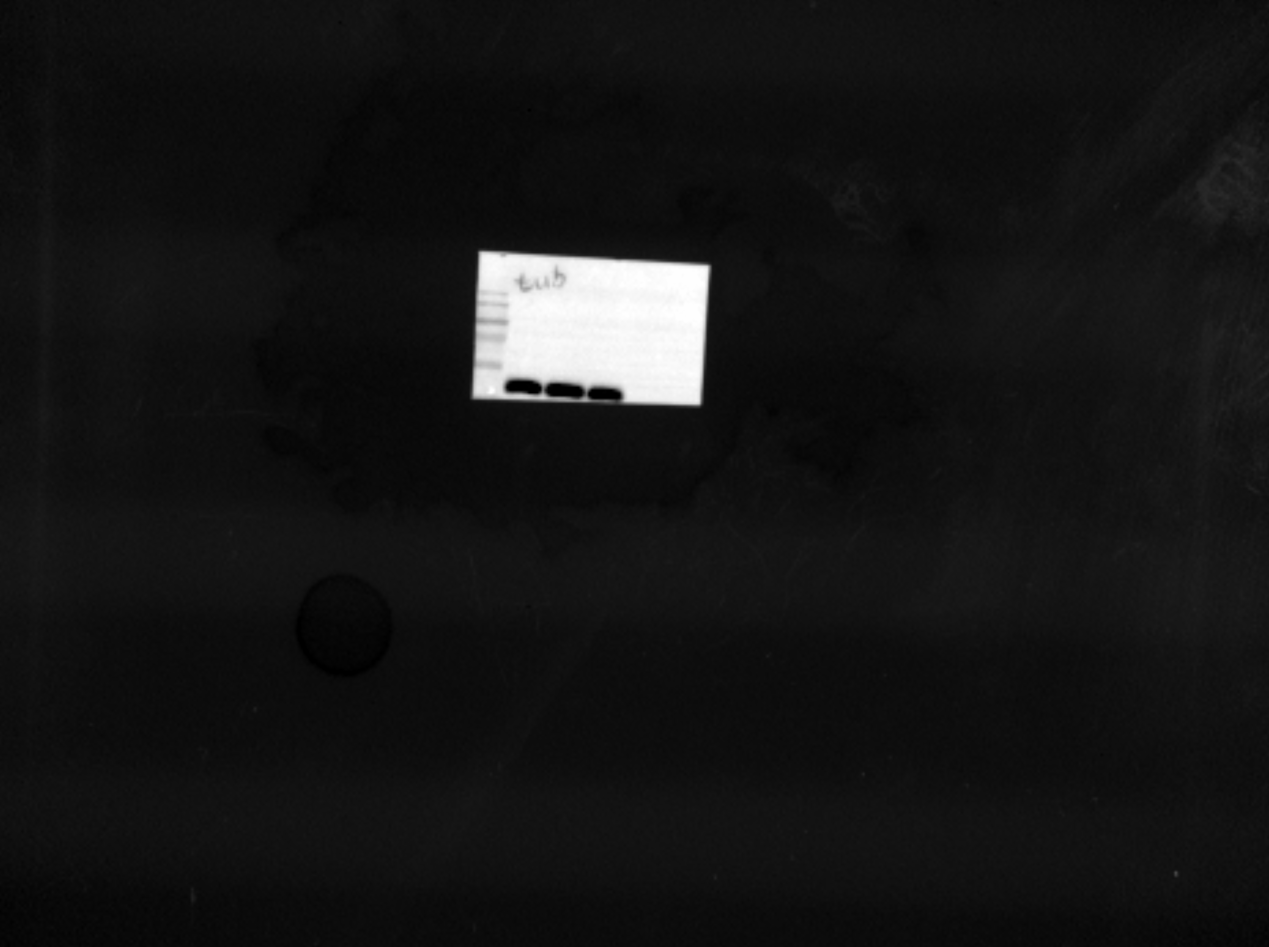


Fig 4D (IP-TMEM43)-TMEM43


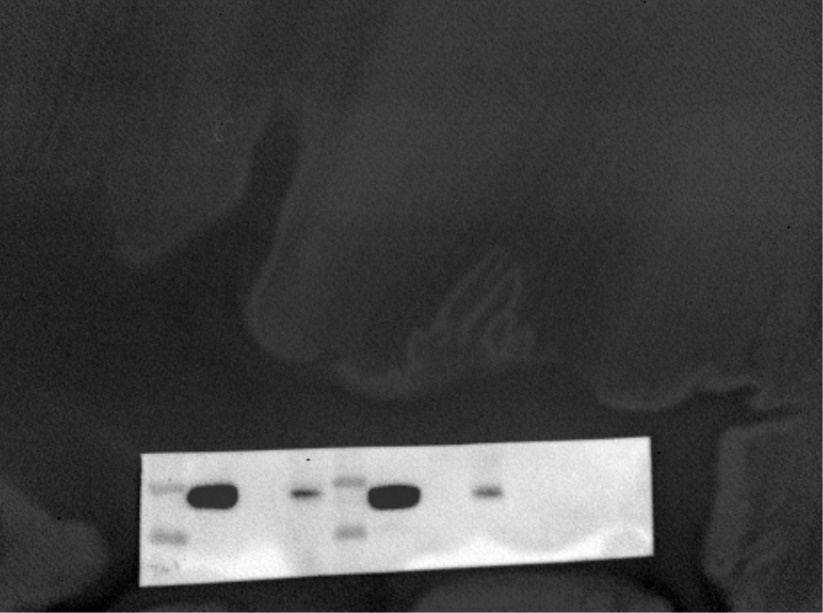


Fig 4D (IP-TMEM43)-PRPF3


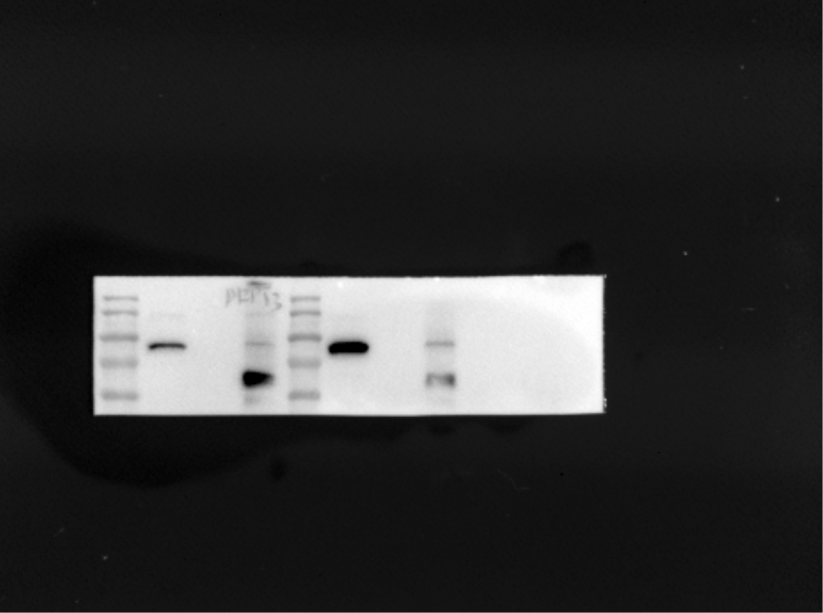


Fig 4D (MIAPaCa-2, IP-PRPF3)-TMEM43


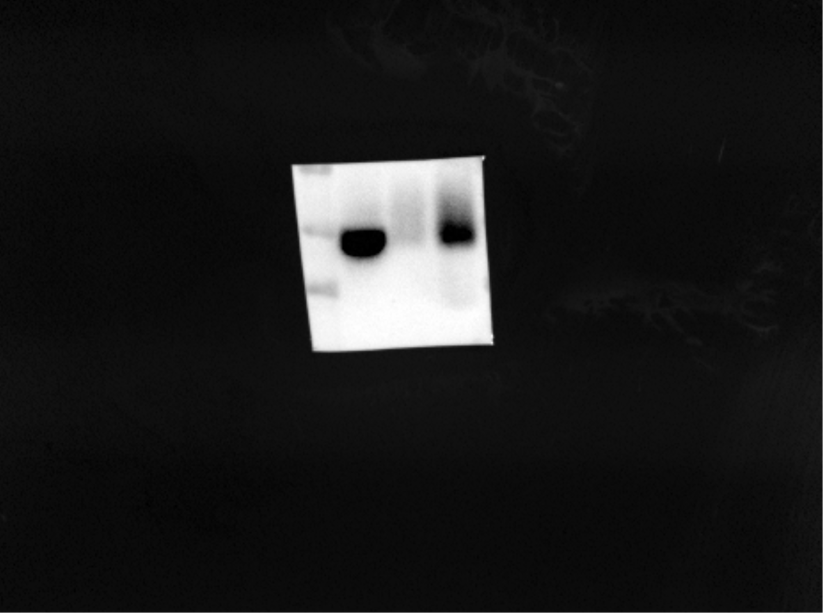


Fig 4D (MIAPaCa-2, IP-PRPF3)-PRPF3


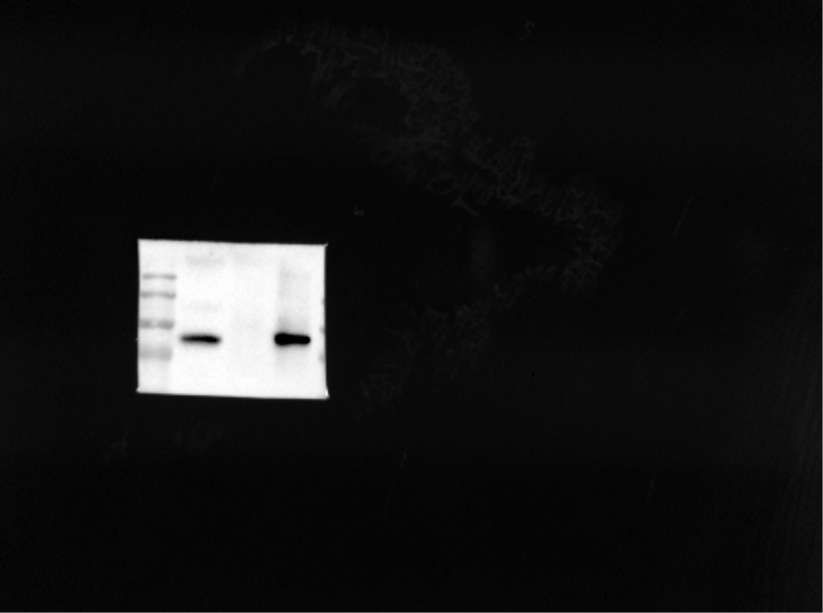


Fig 4D (SW1990, IP-PRPF3)-TMEM43


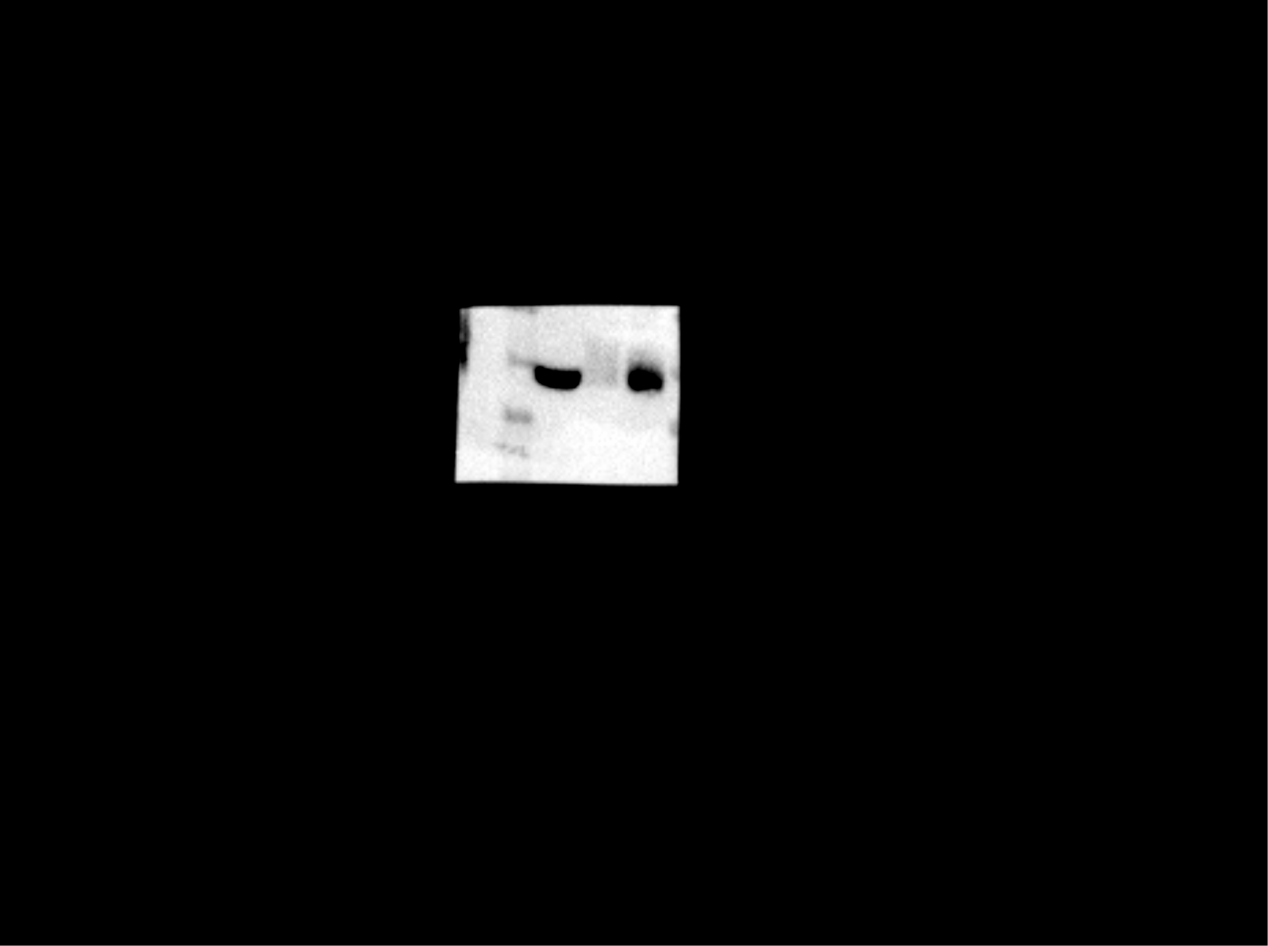


Fig 4D (SW1990, IP-PRPF3)-PRPF3


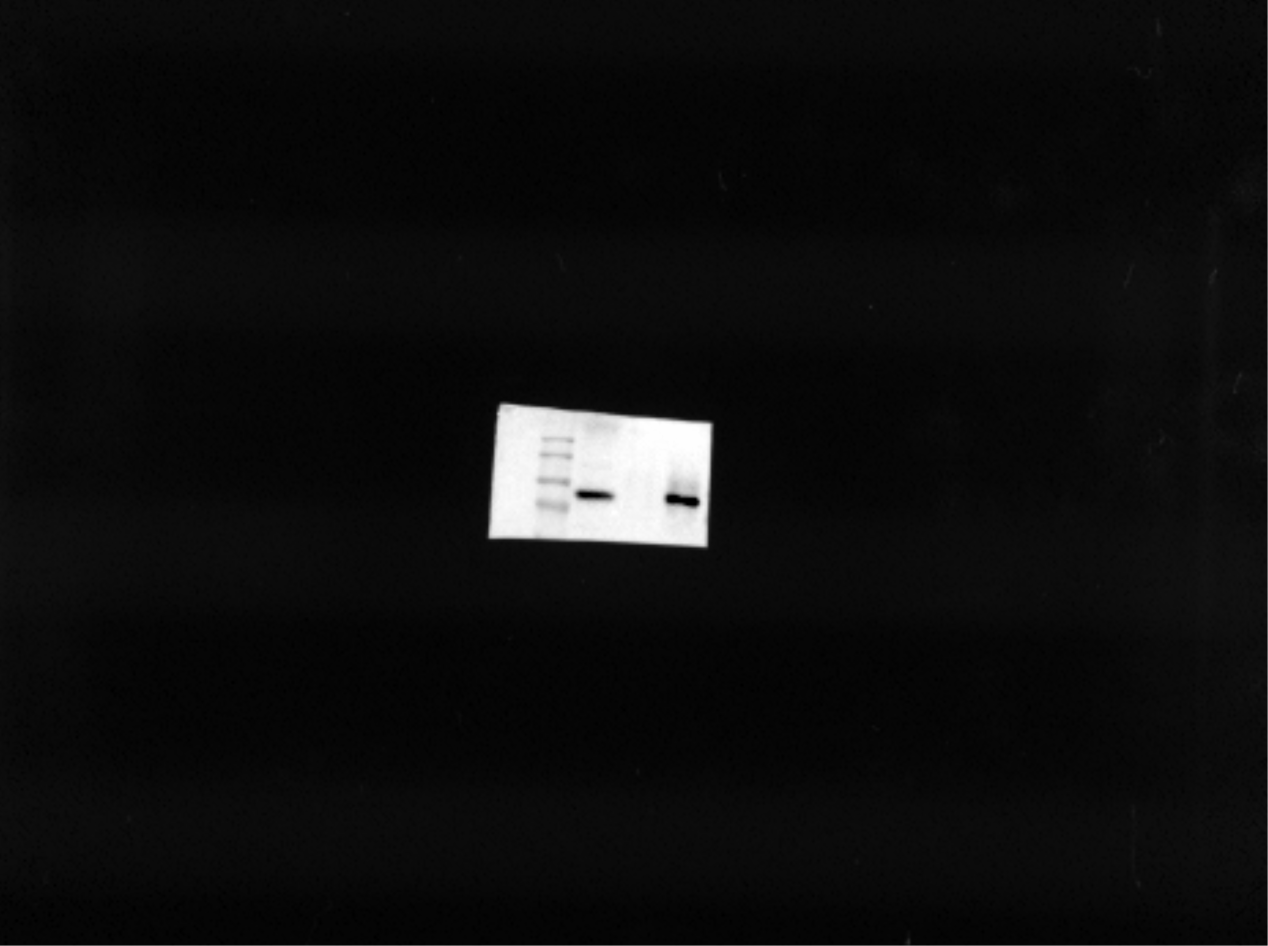


Fig 4E (IP-Flag)-Flag


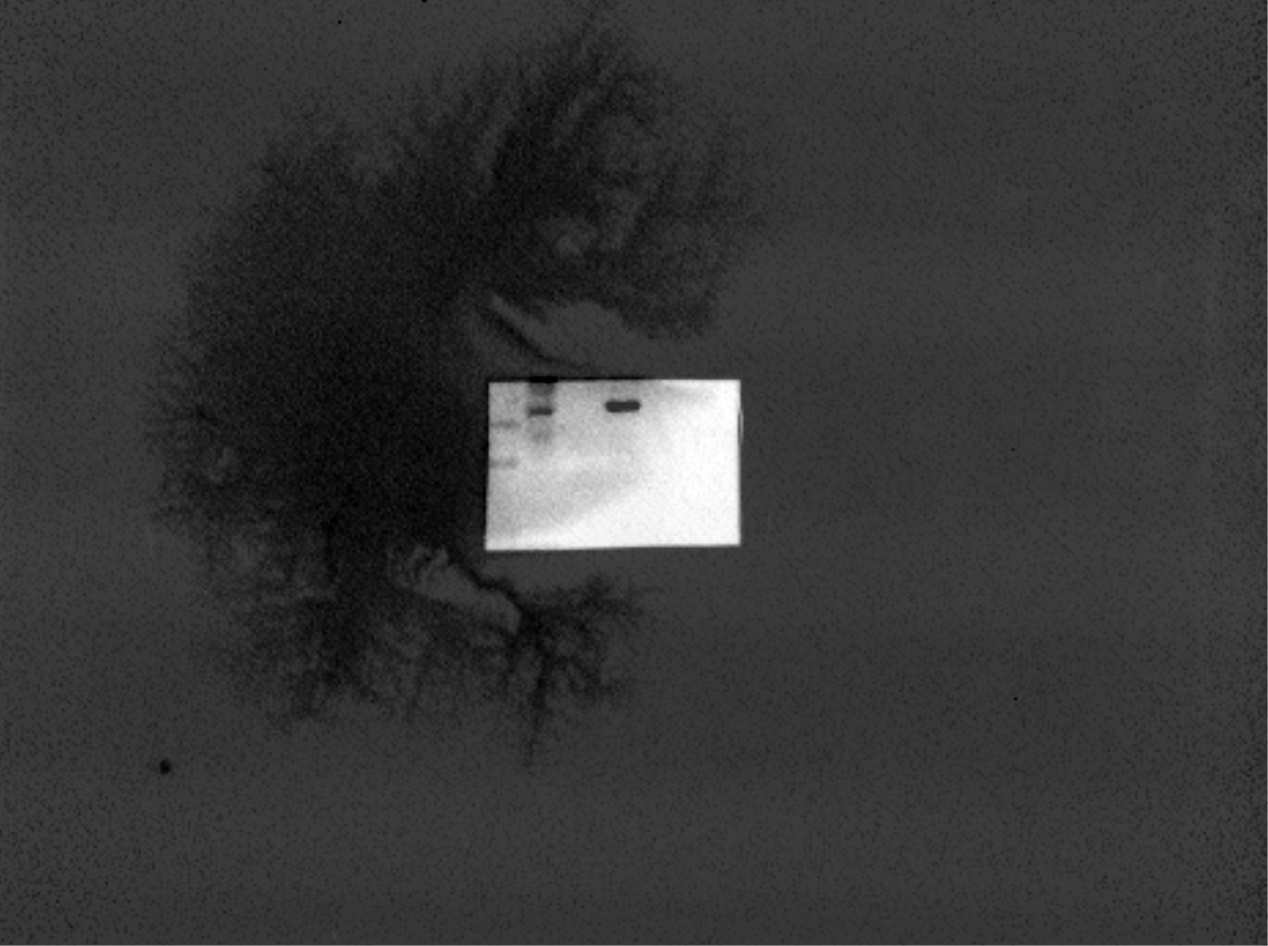


Fig 4E (IP-Flag)-HA


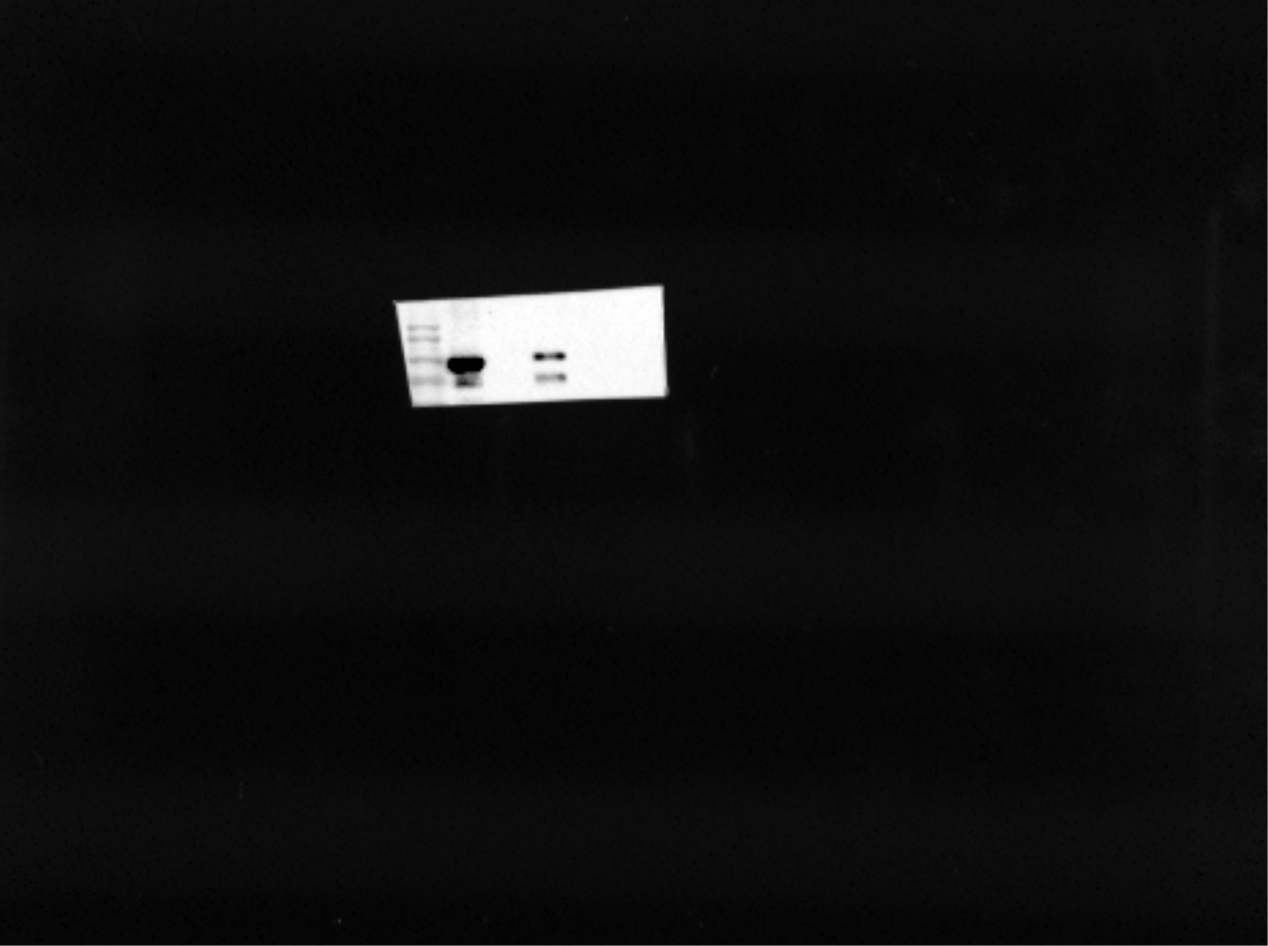


Fig 4E (IP-HA)-Flag


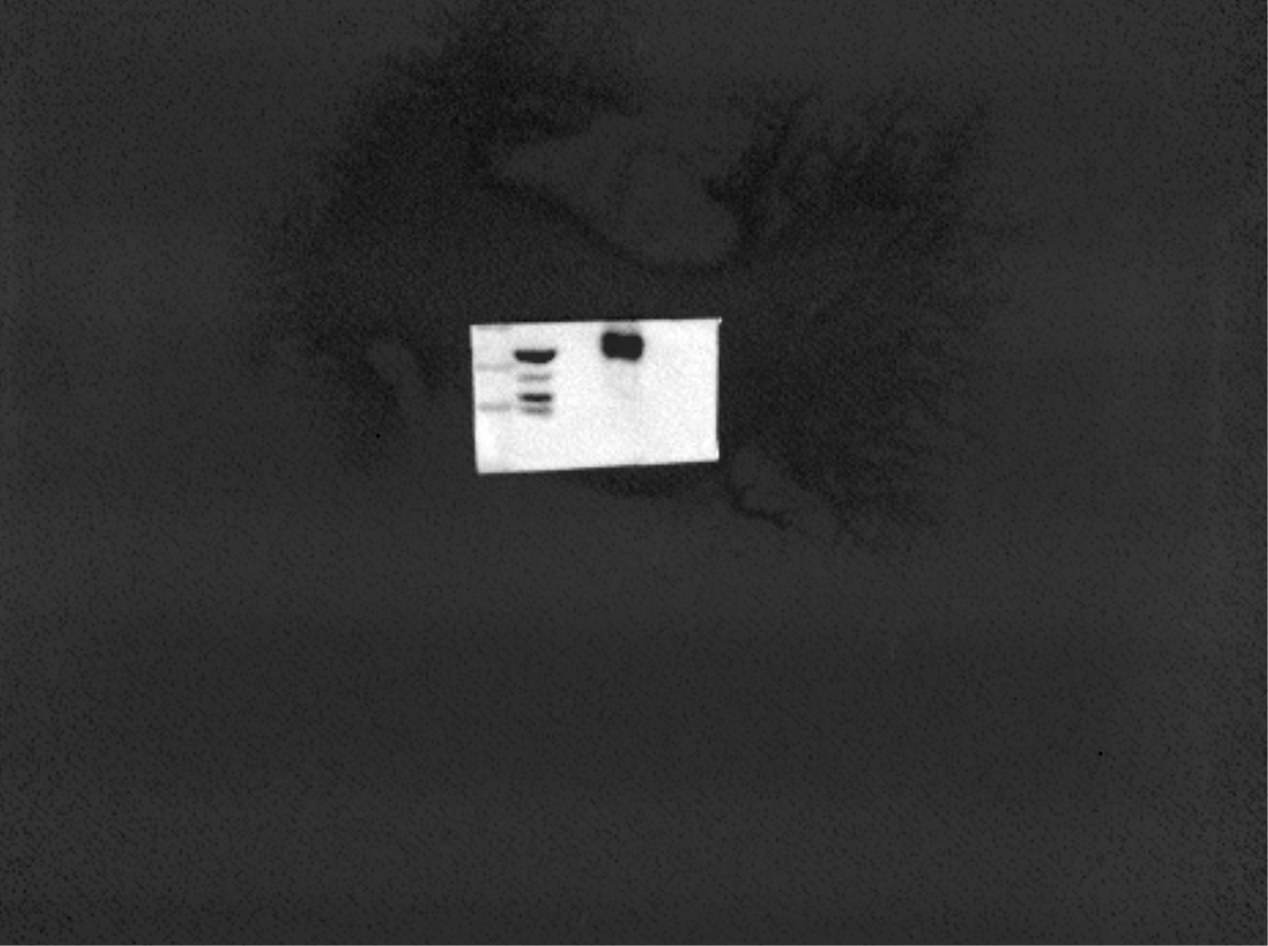


Fig 4E (IP-HA)-HA


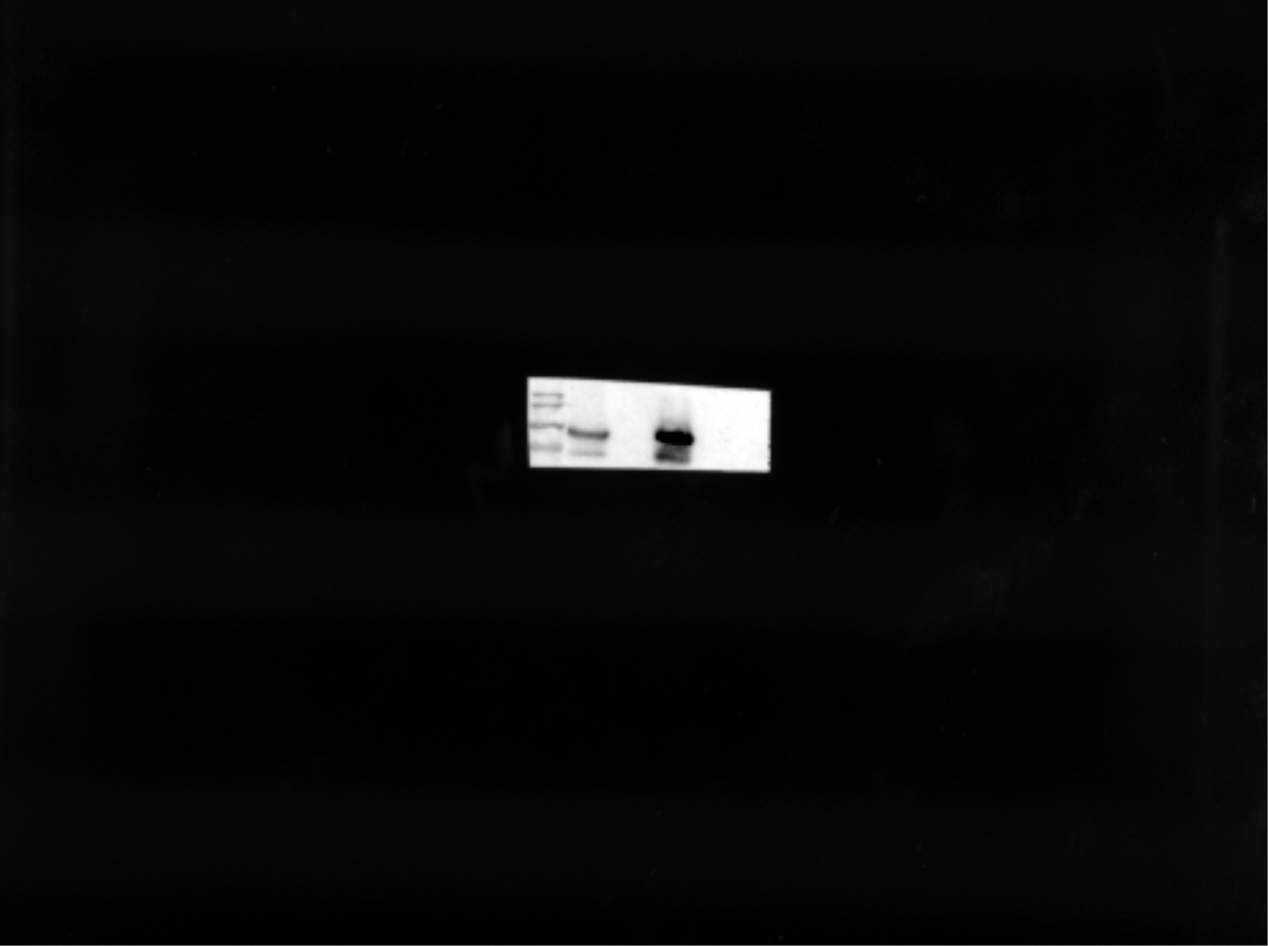


Fig 4G (IP)-HA


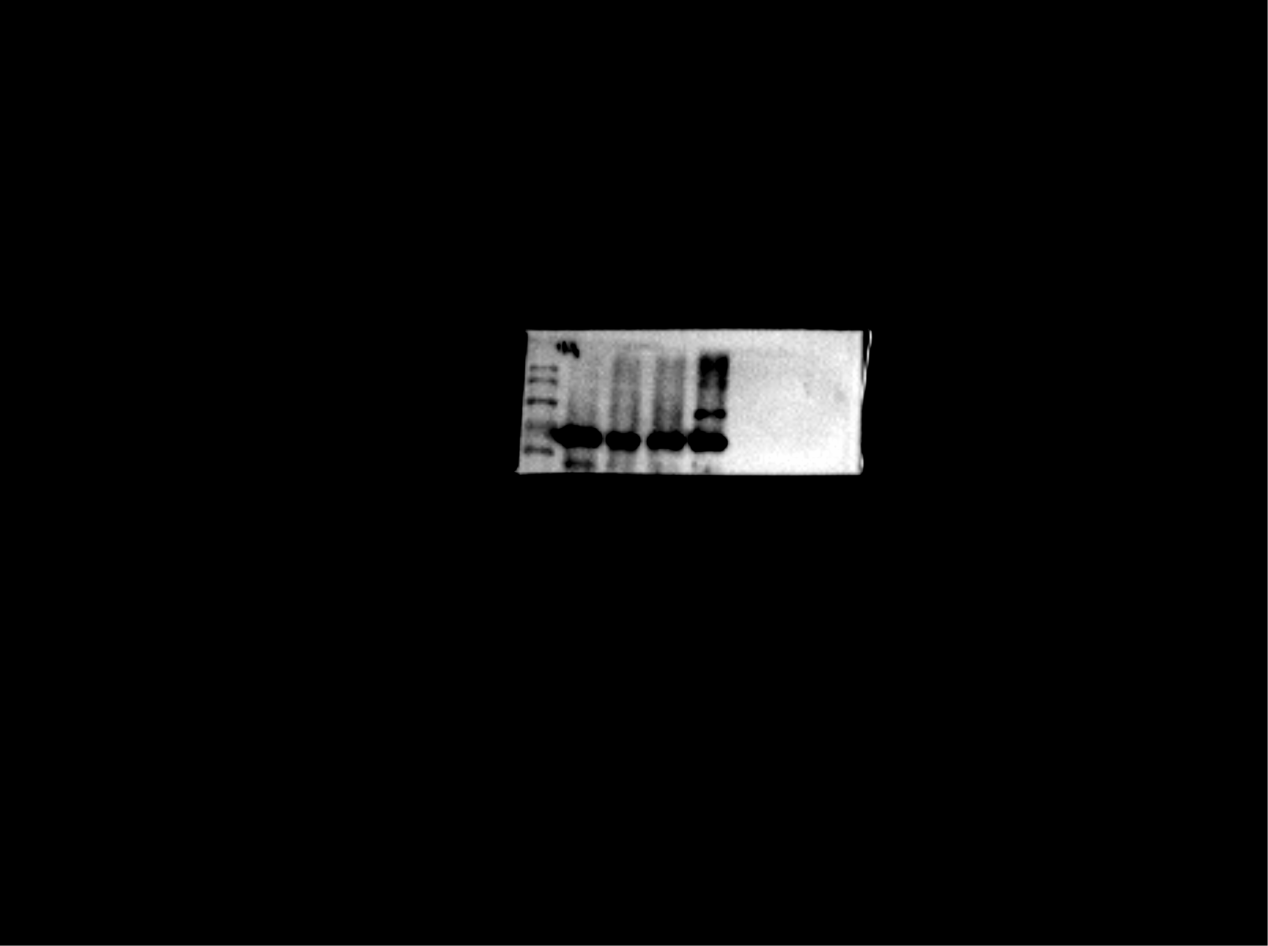


Fig 4G (IP)-Flag


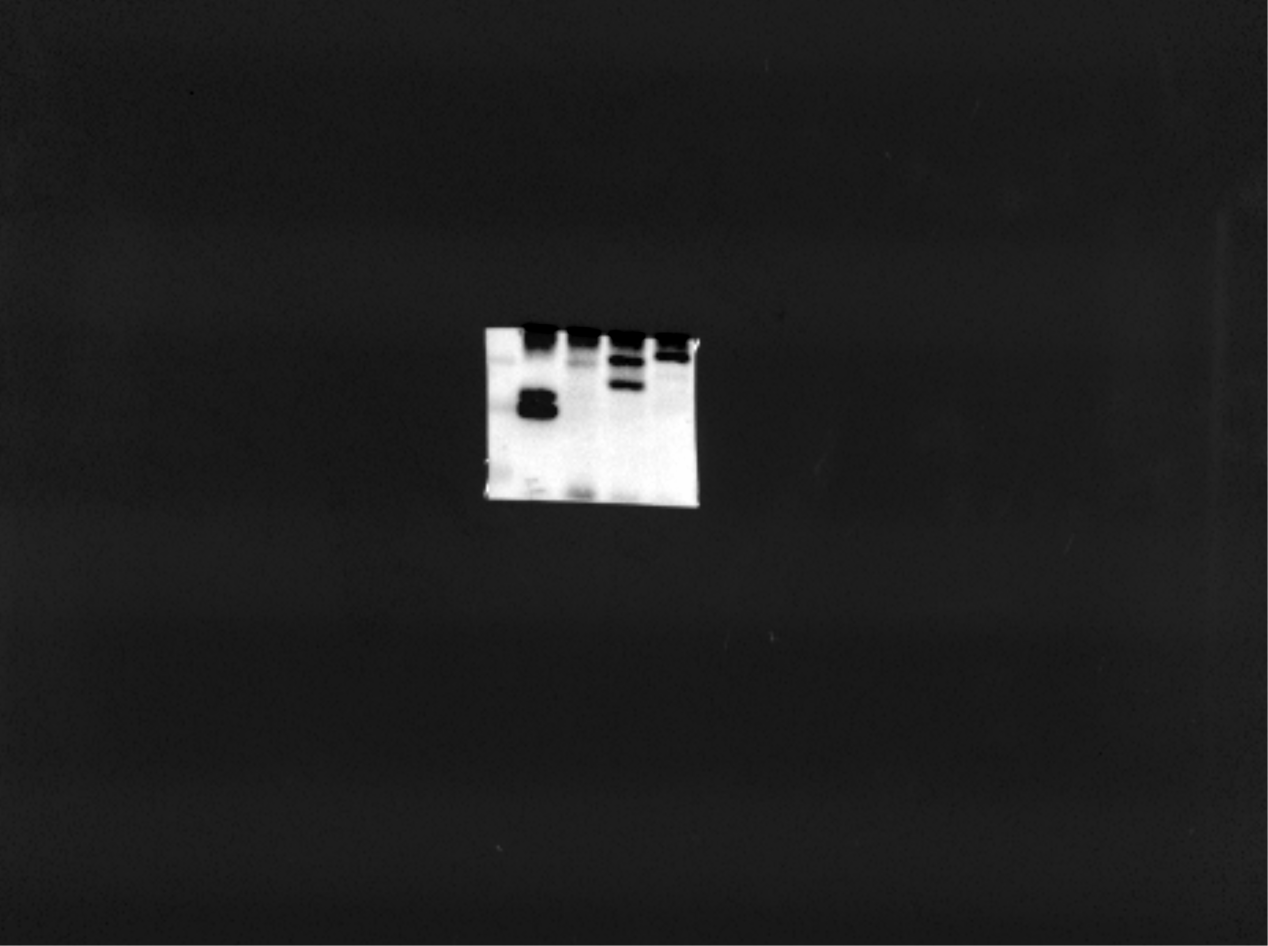


Fig 4G (Input)-Flag


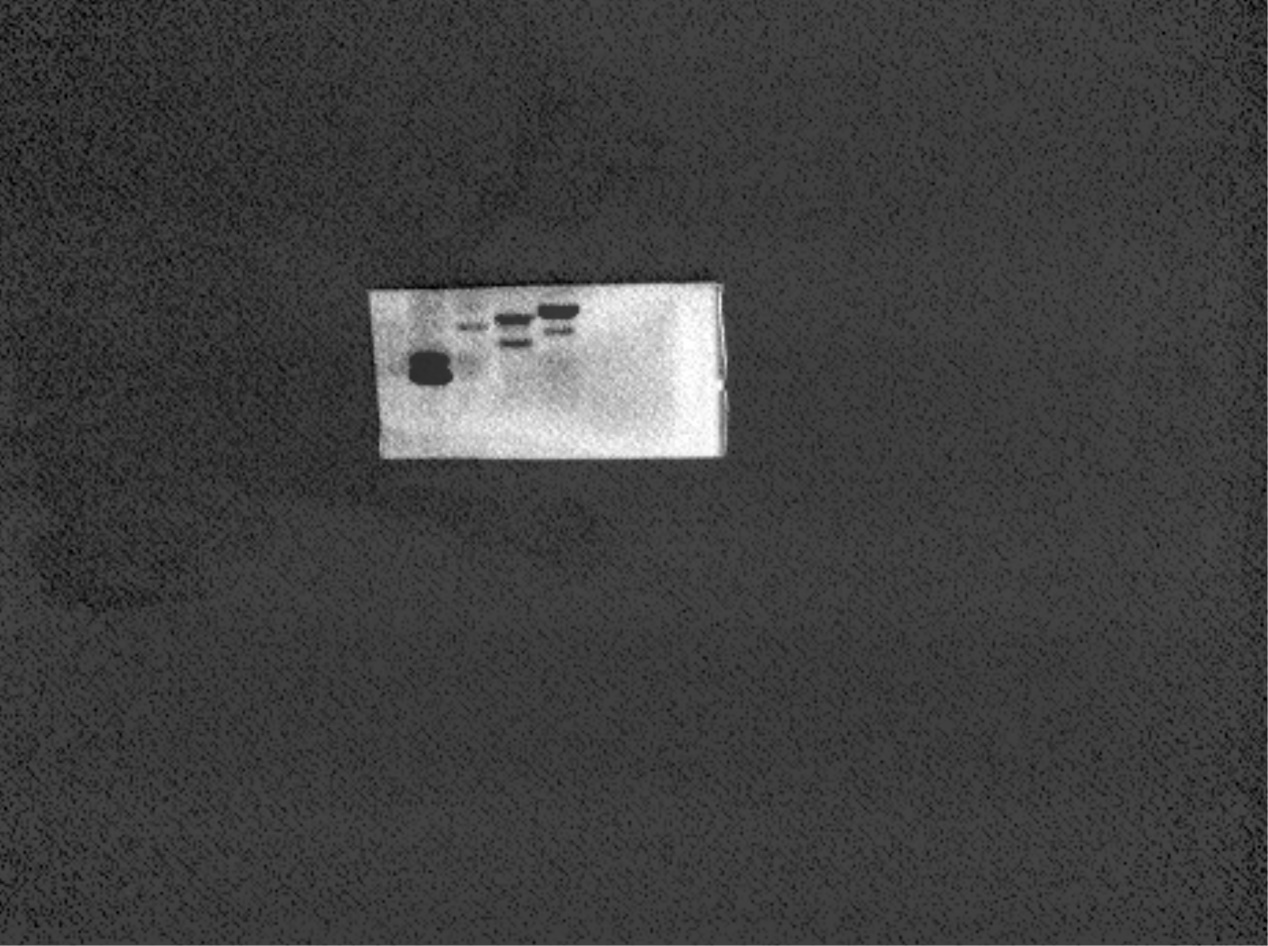


Fig 4G (Input)-HA


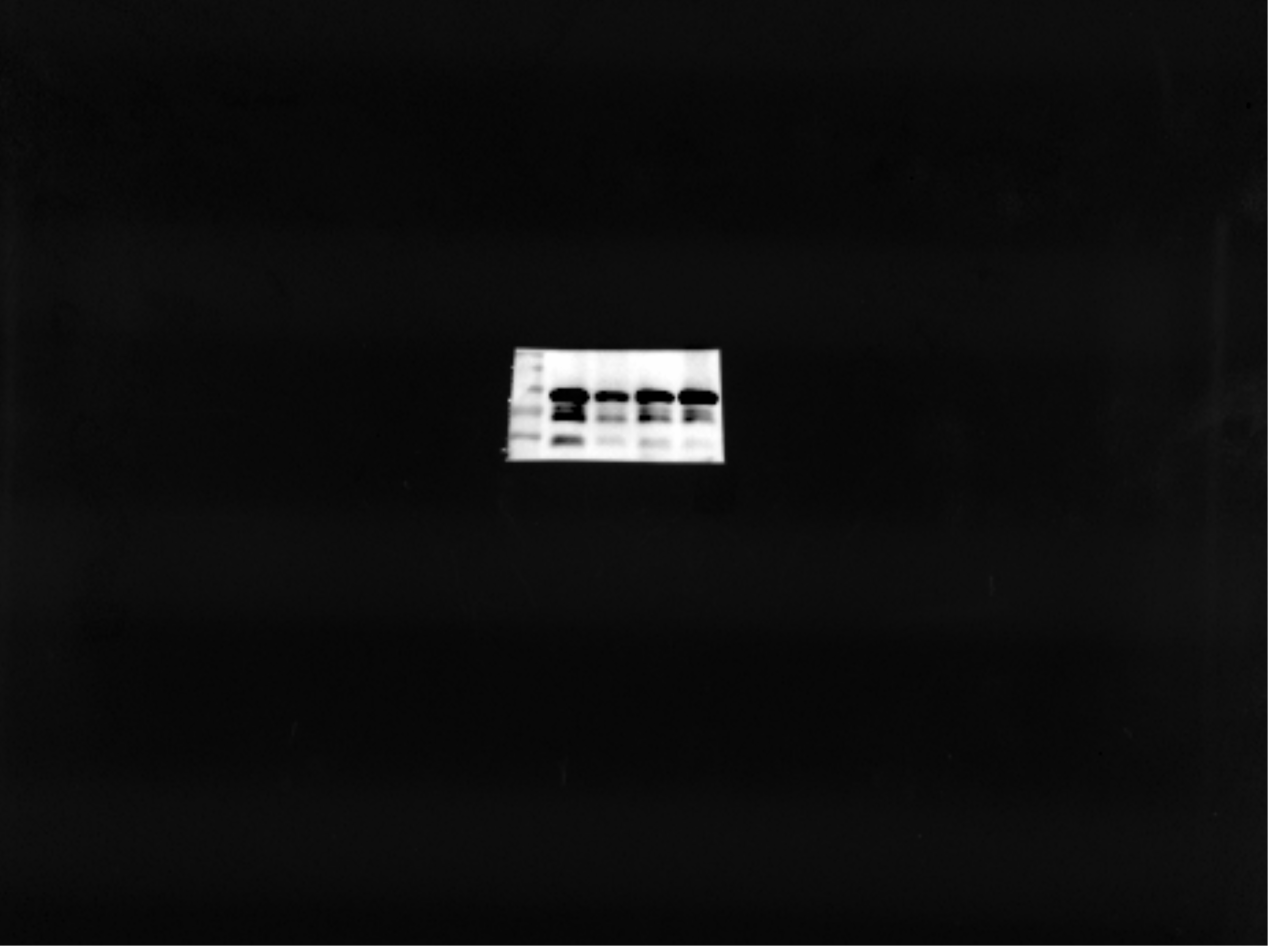


Fig 4G (Input)-α-tublin


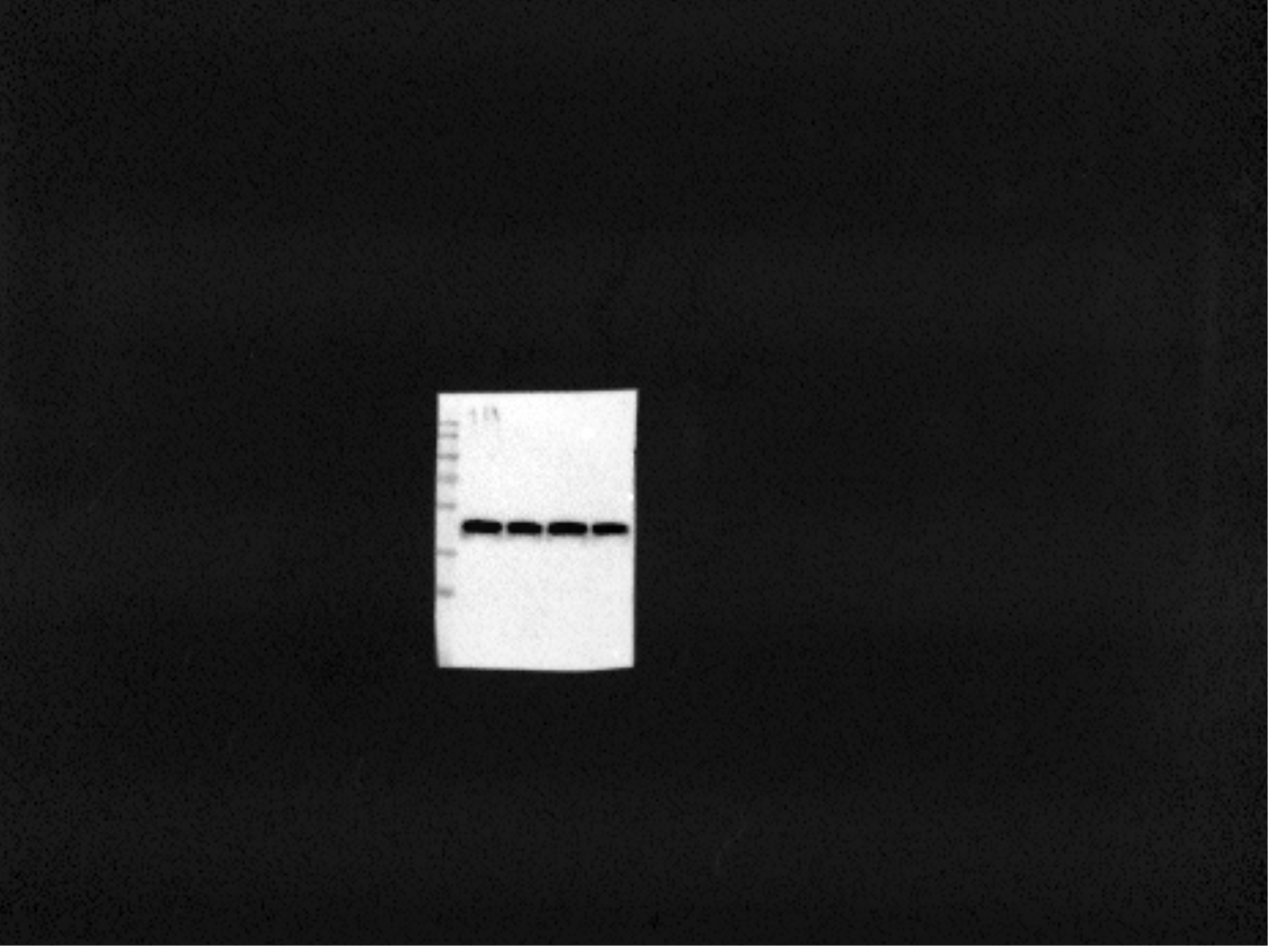


Fig 4H (MIAPaCa-2)-TMEM43


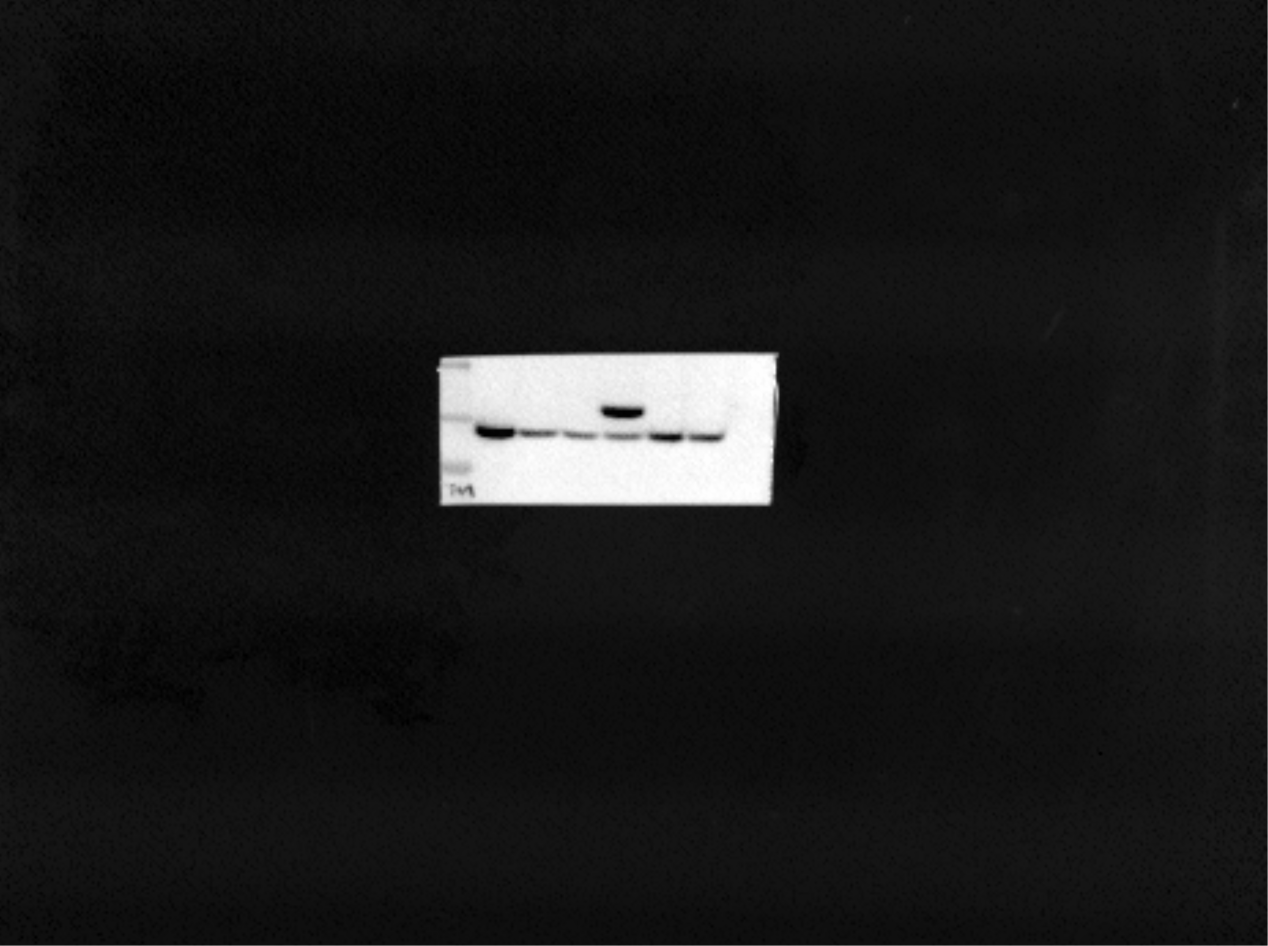


Fig 4H (MIAPaCa-2)-PRPF3


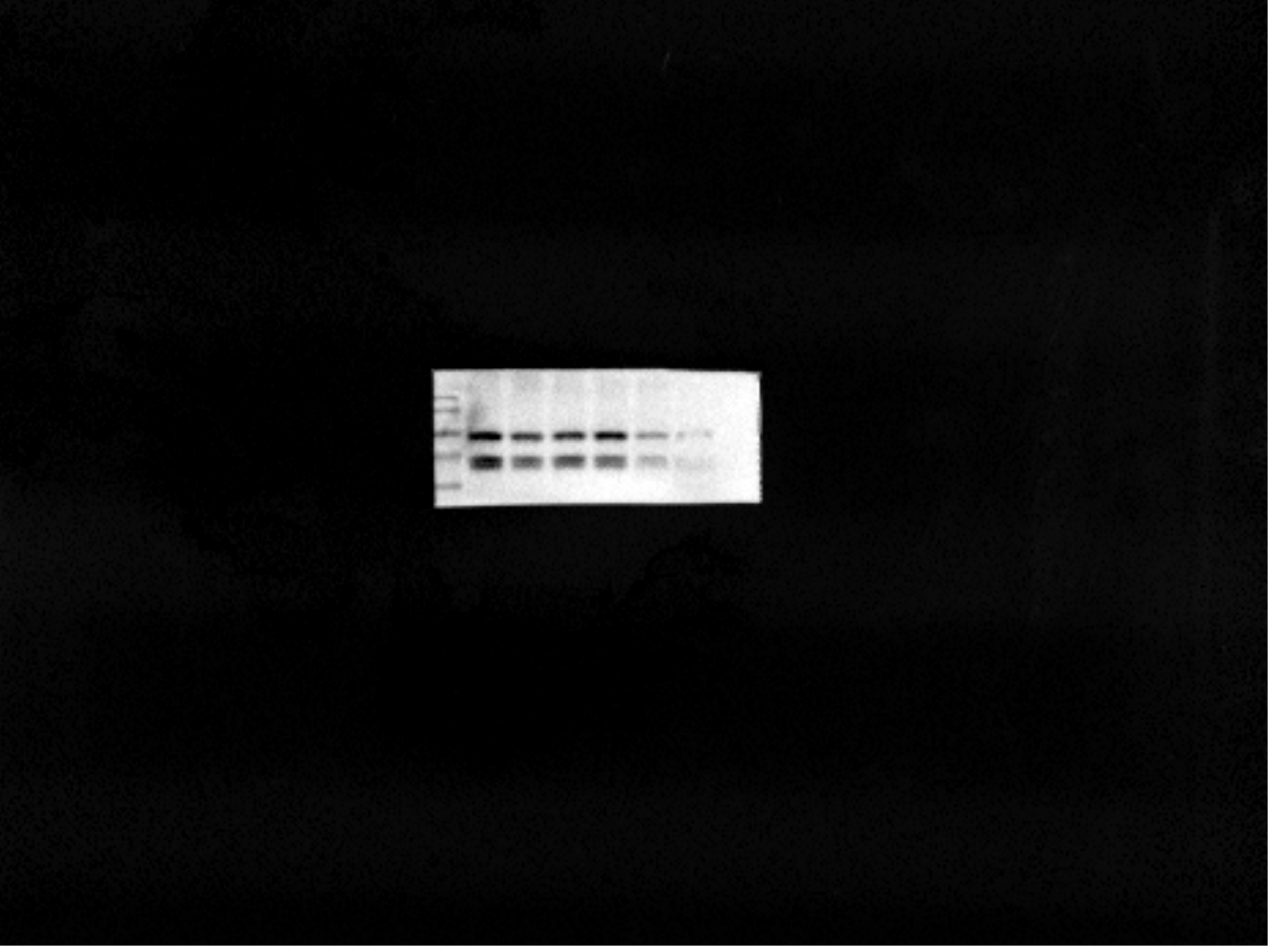


Fig 4H(MIAPaCa-2) -α-tublin


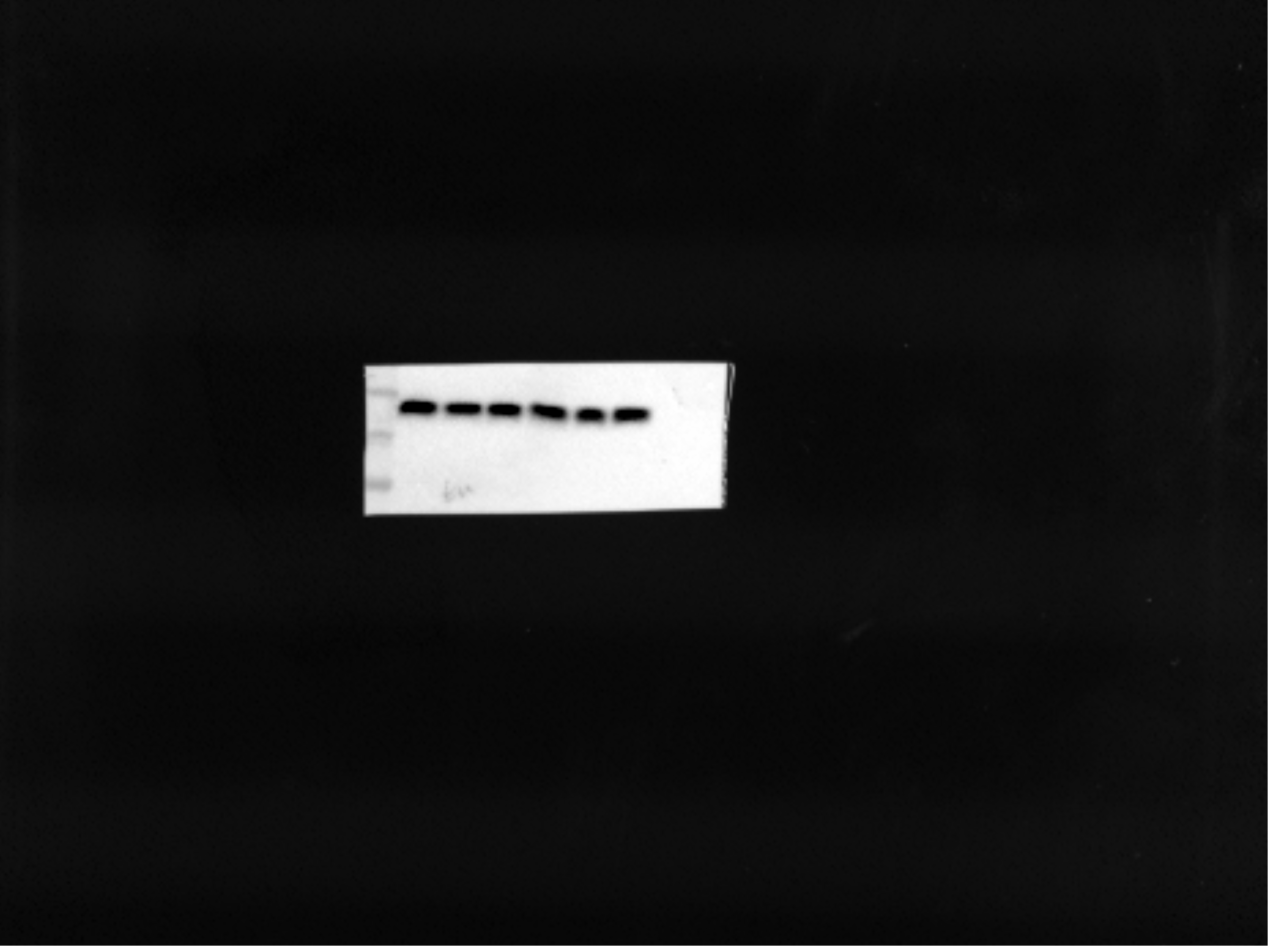


Fig 4H (SW1990)-TMEM43


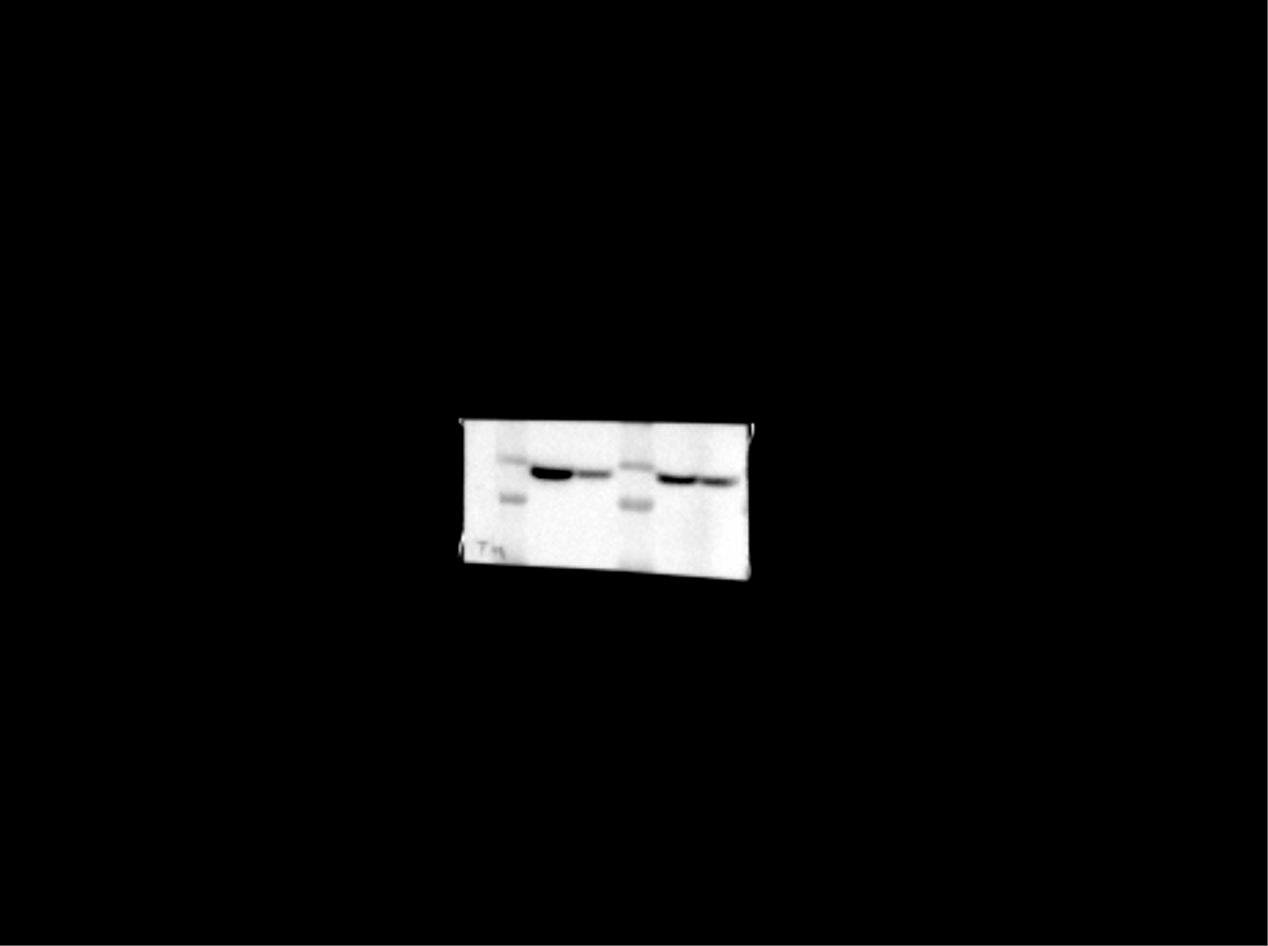


Fig 4H (SW1990)-PRPF3


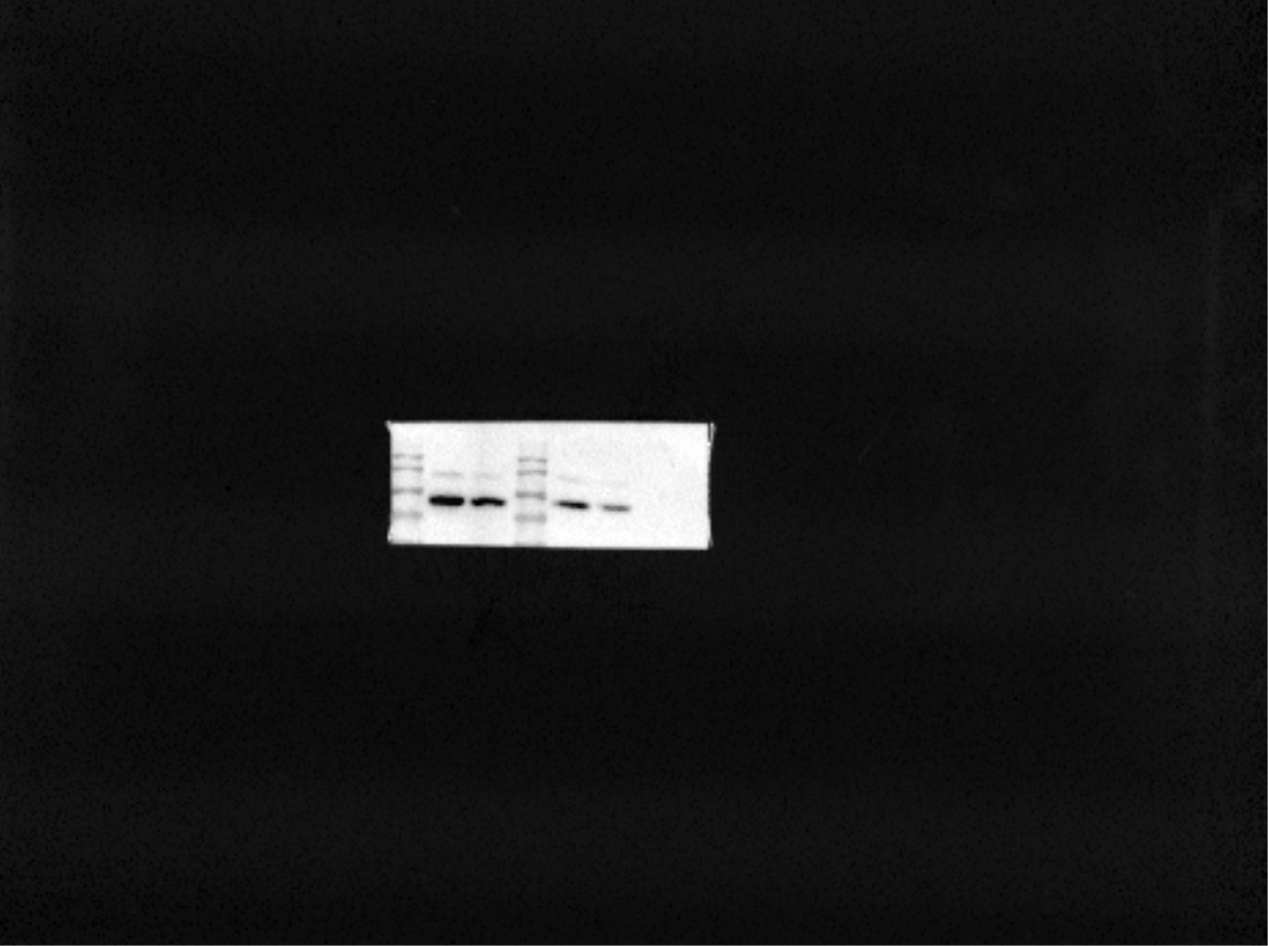


Fig 4H (SW1990) -α-tublin


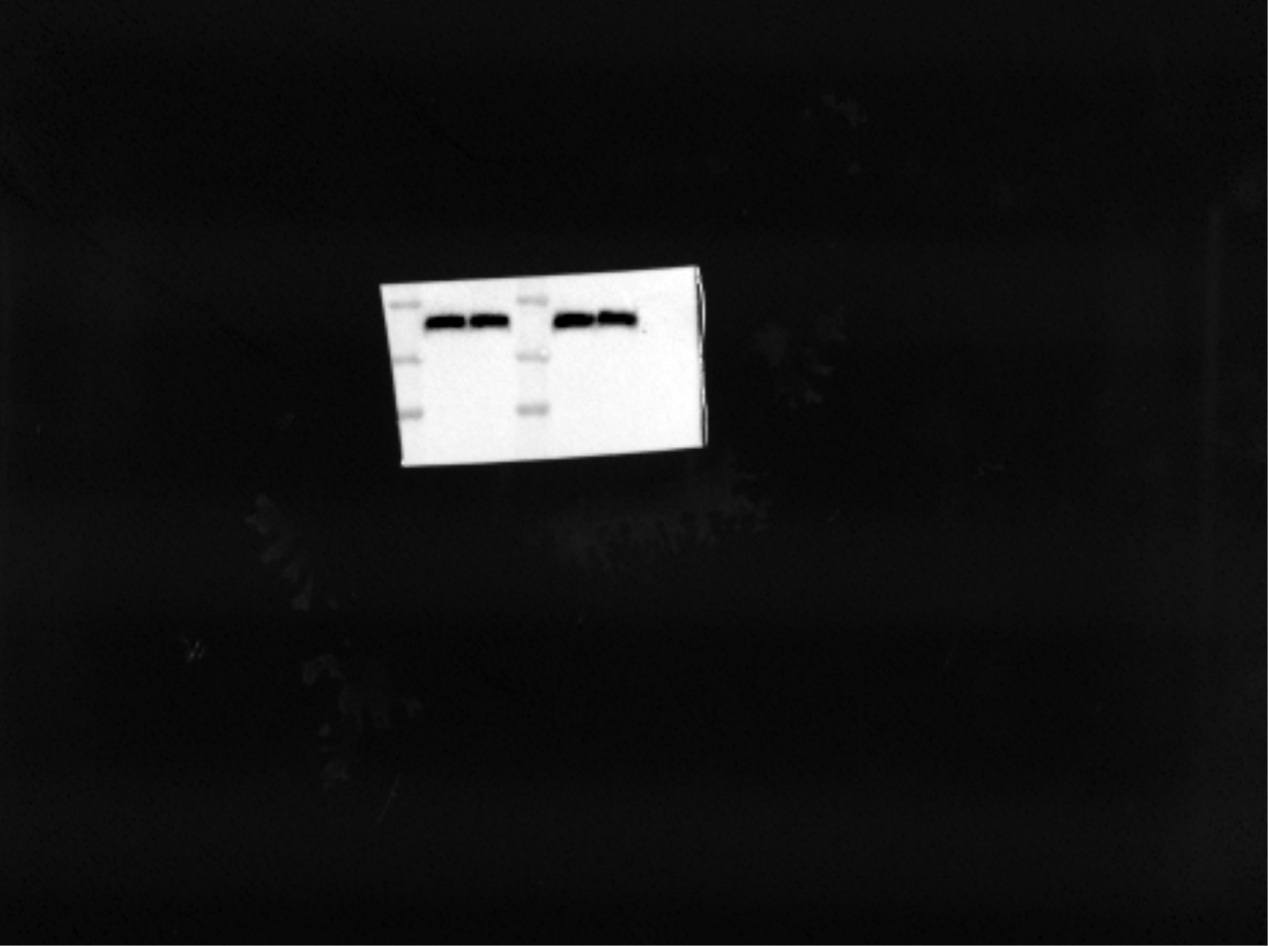


Fig 4J (MIAPaCa-2) –TMEM43


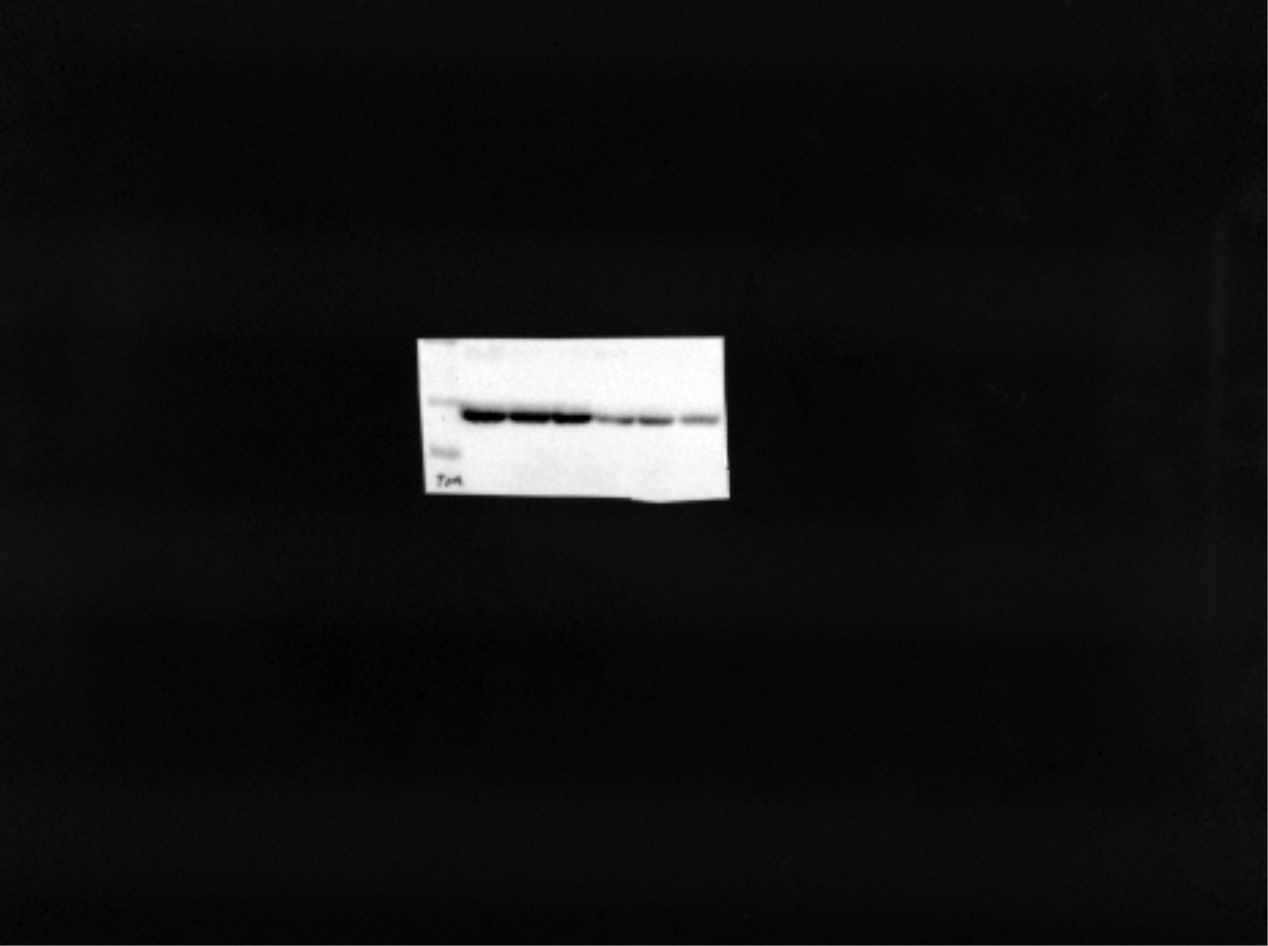


Fig 4J (MIAPaCa-2) –PRPF3


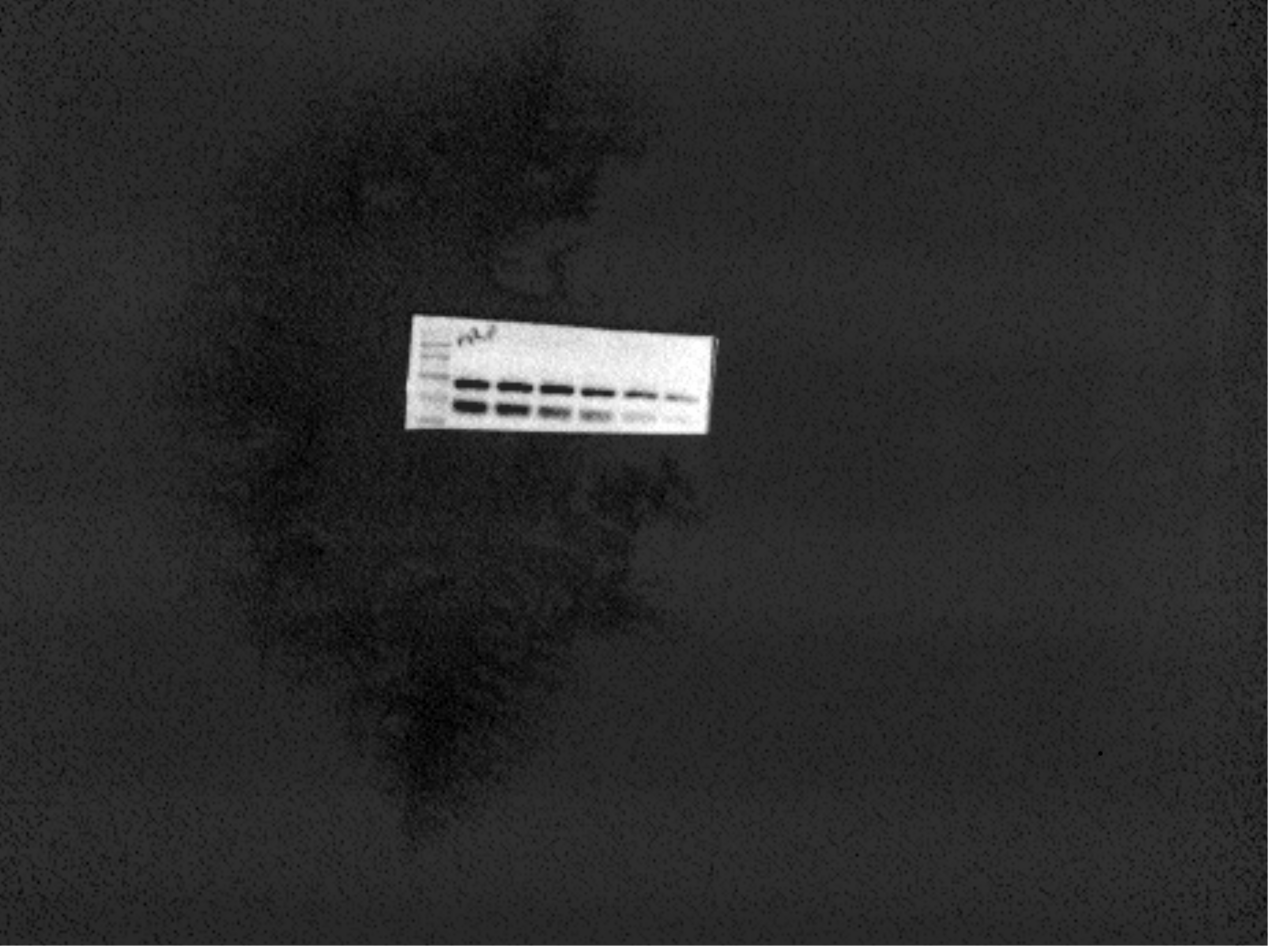


Fig 4J (MIAPaCa-2) –tublin


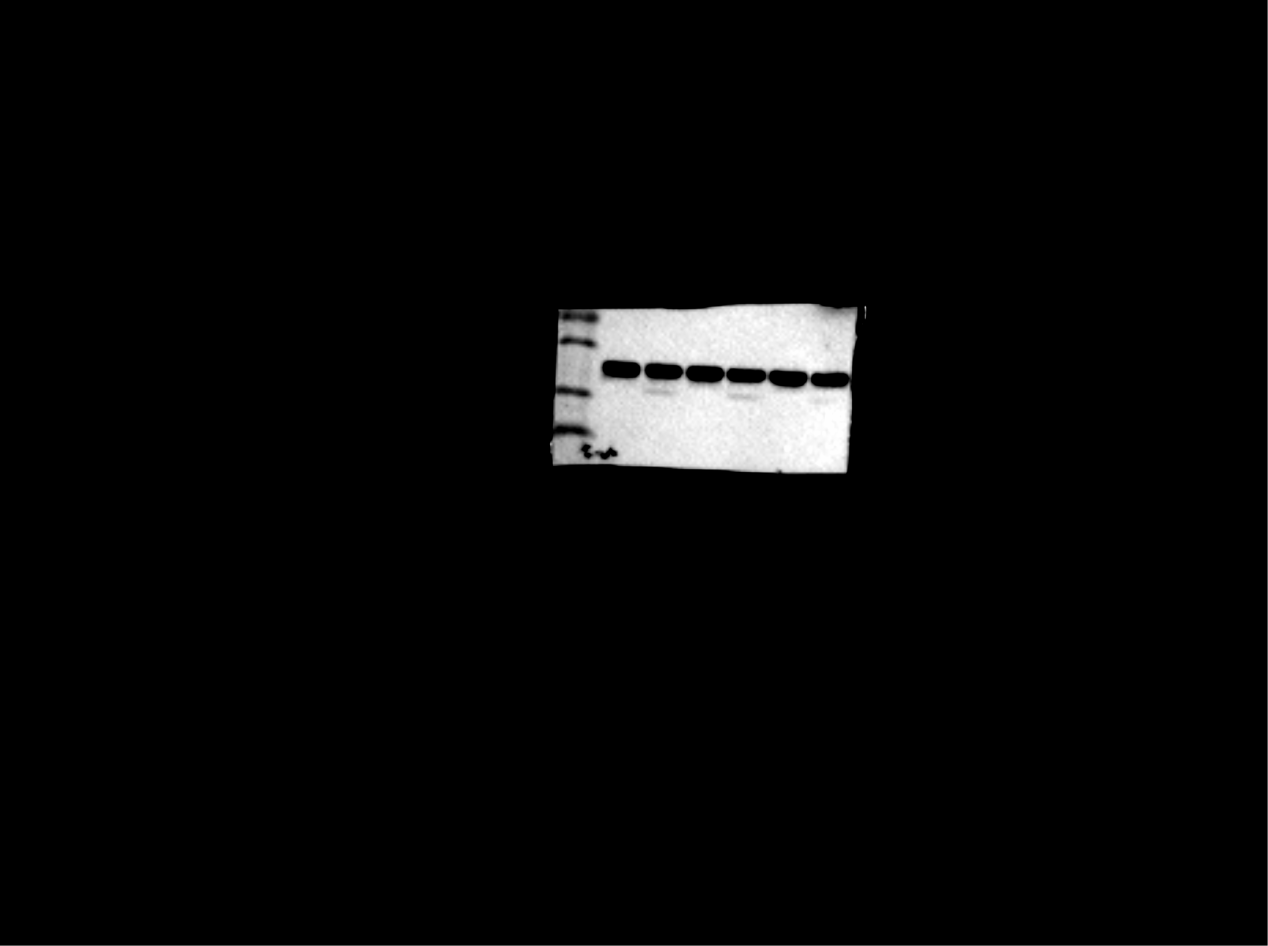


Fig 4J (SW1990) –TMEM43


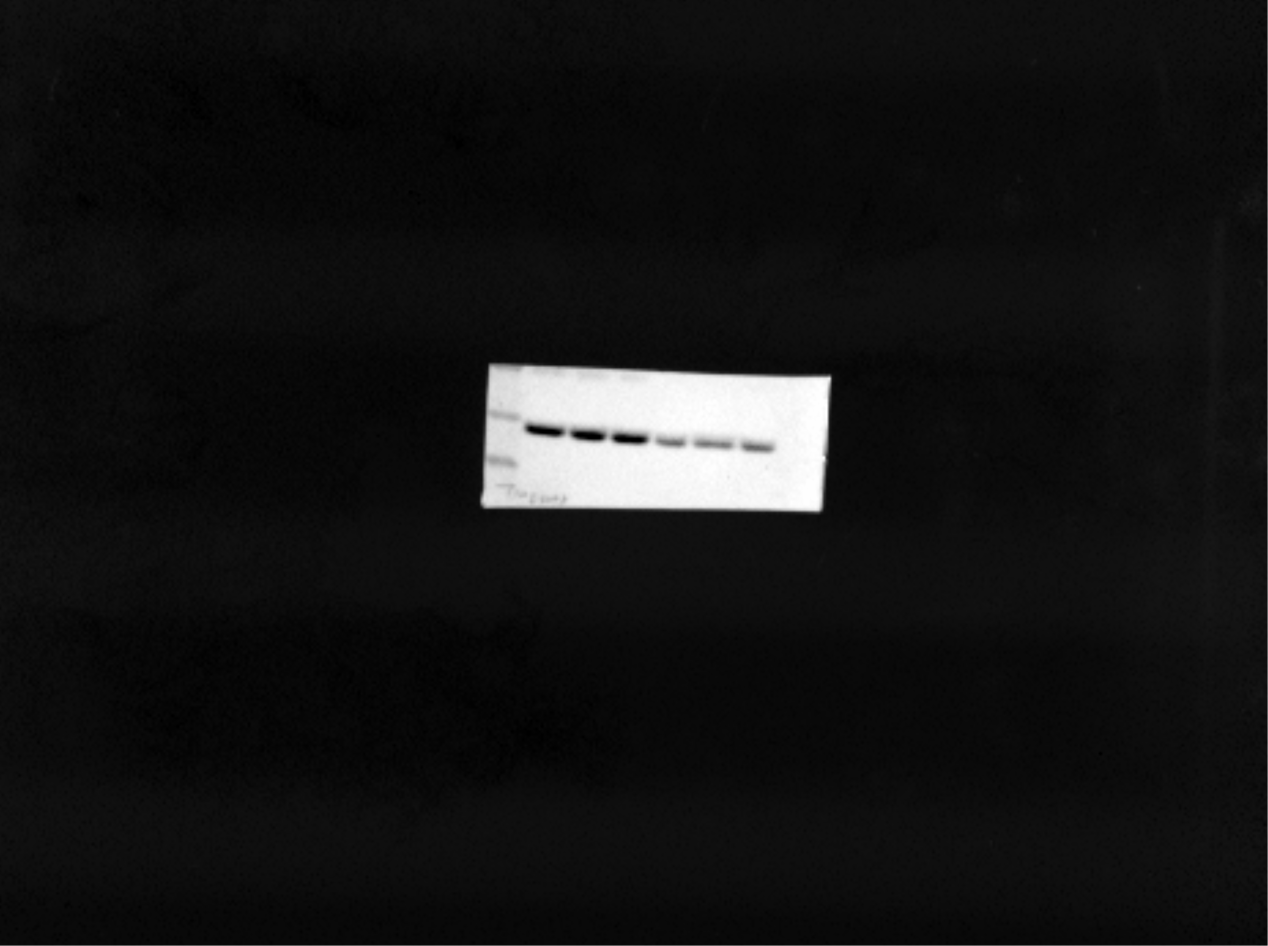


Fig 4J (SW1990) –PRPF3


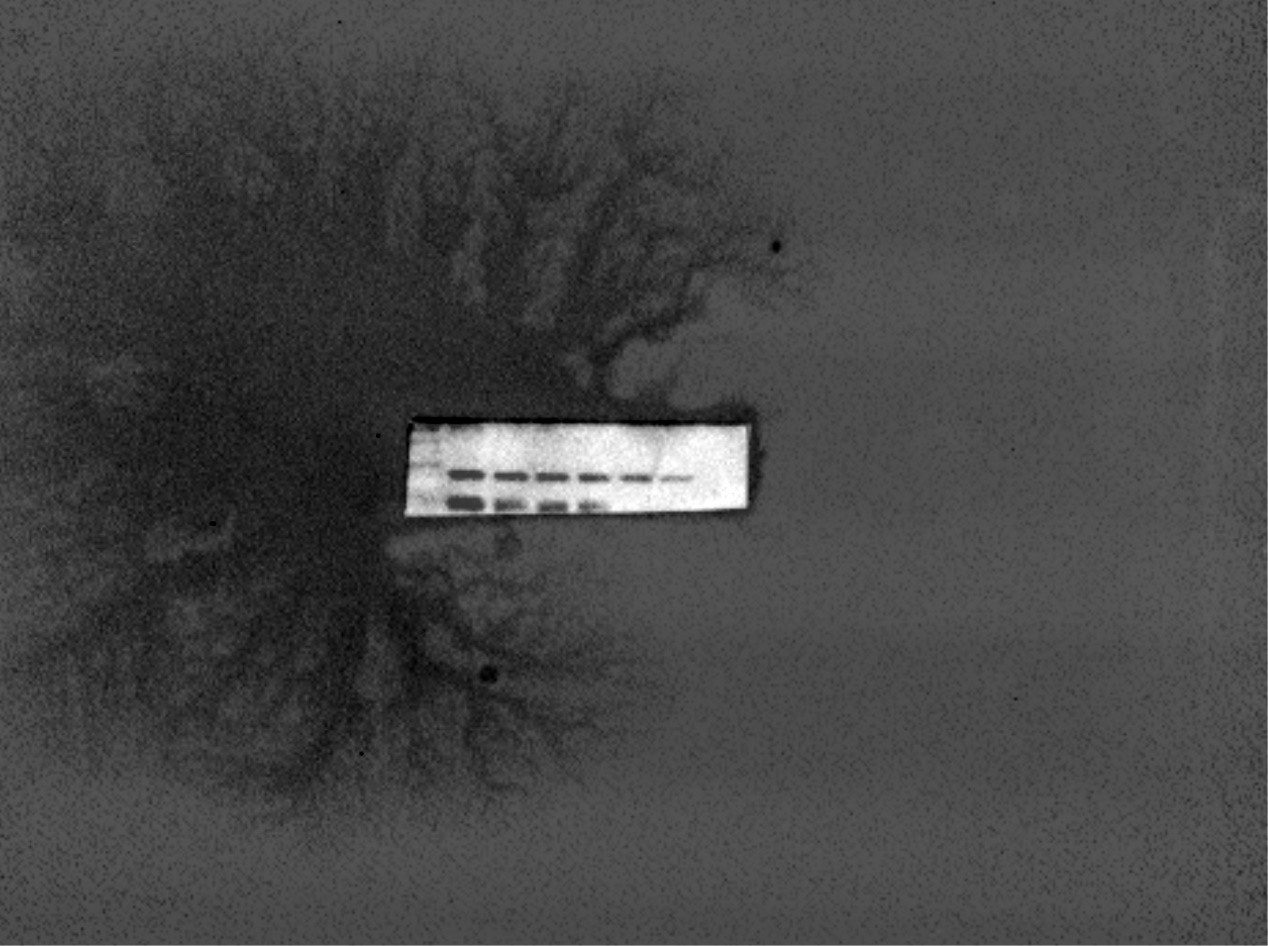


Fig 4J (SW1990) –tublin


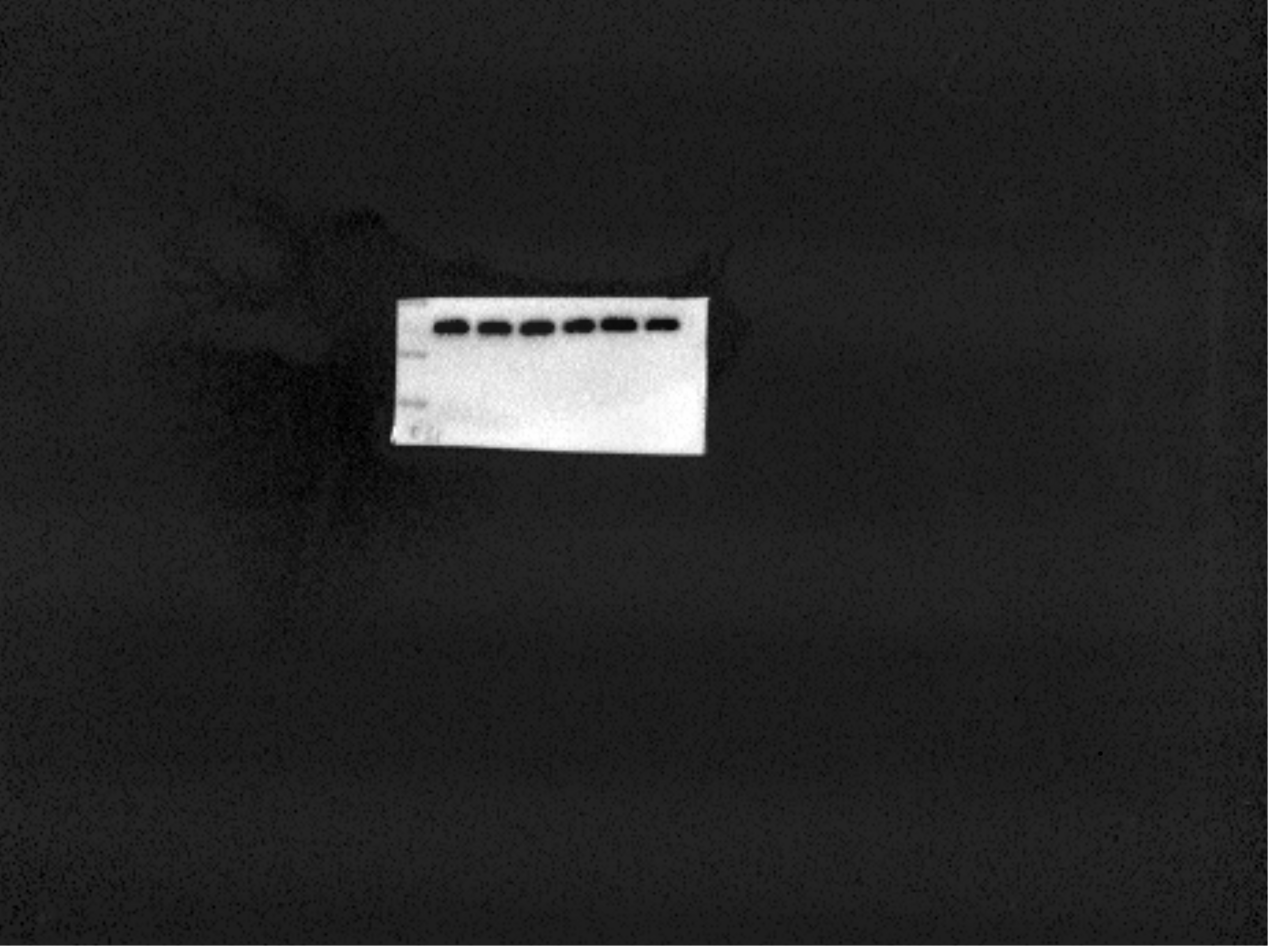


Fig 5I-PRPF3


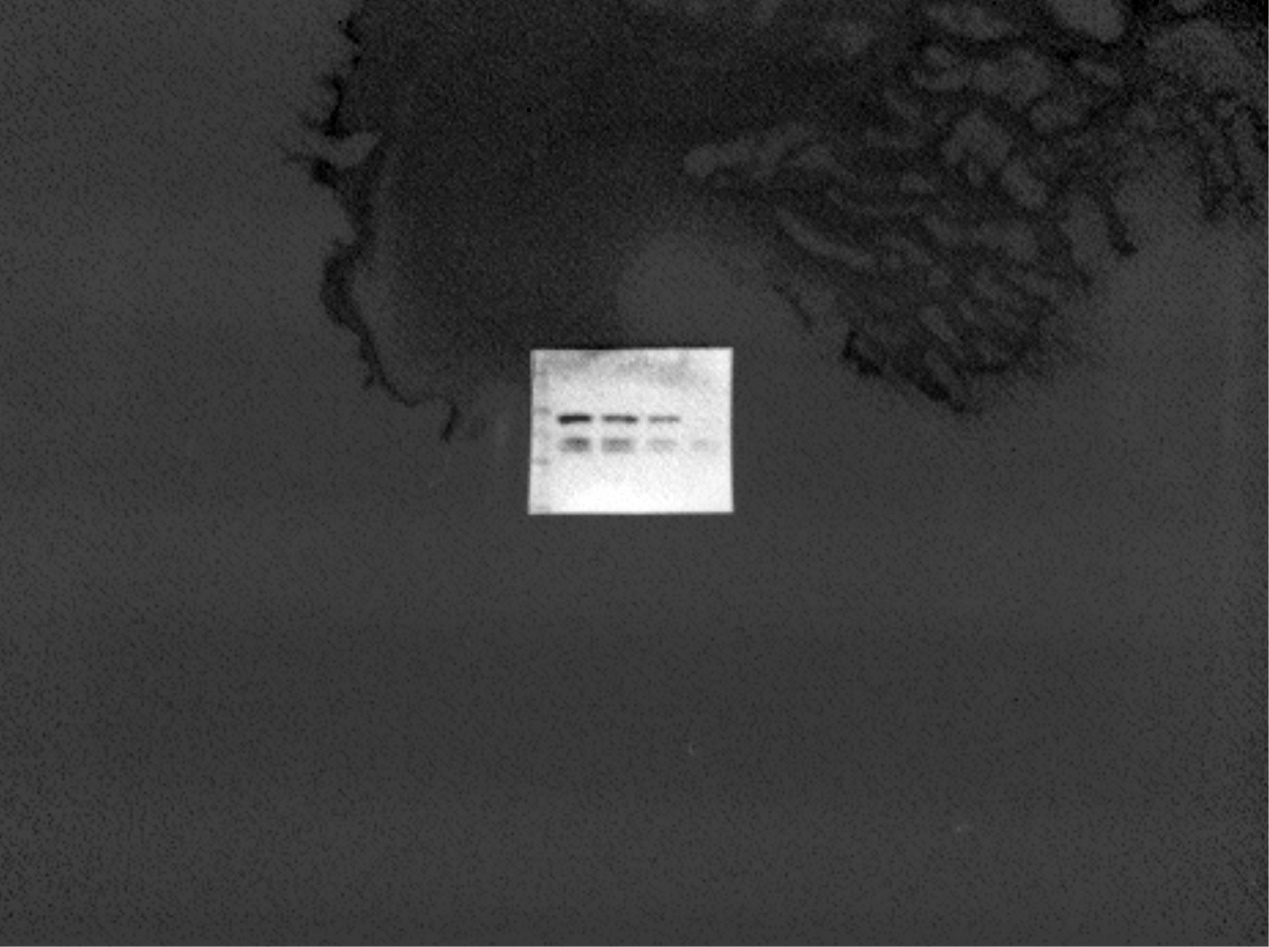


Fig 5I-RAP2B


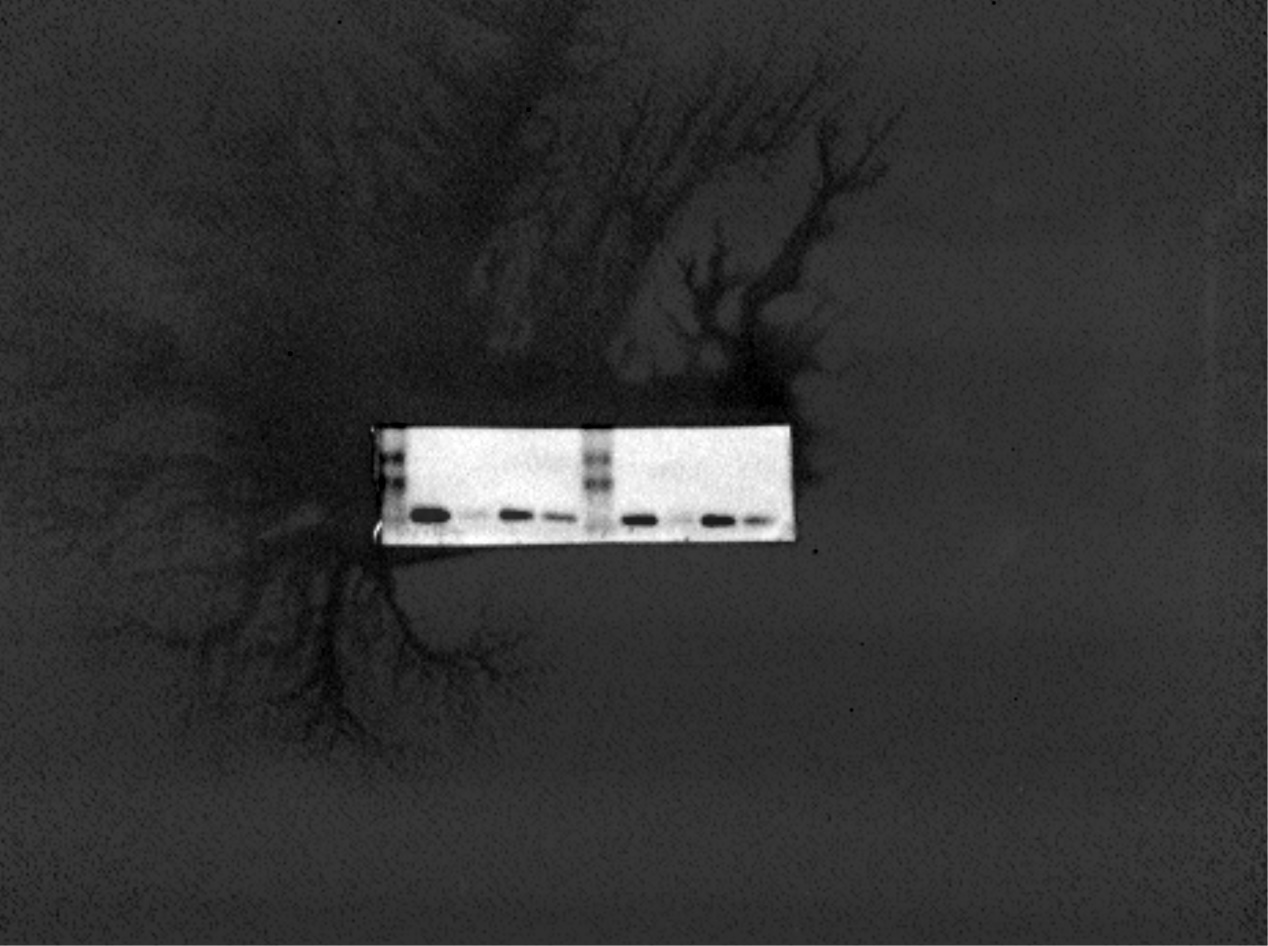


Fig 5I-p-ERK


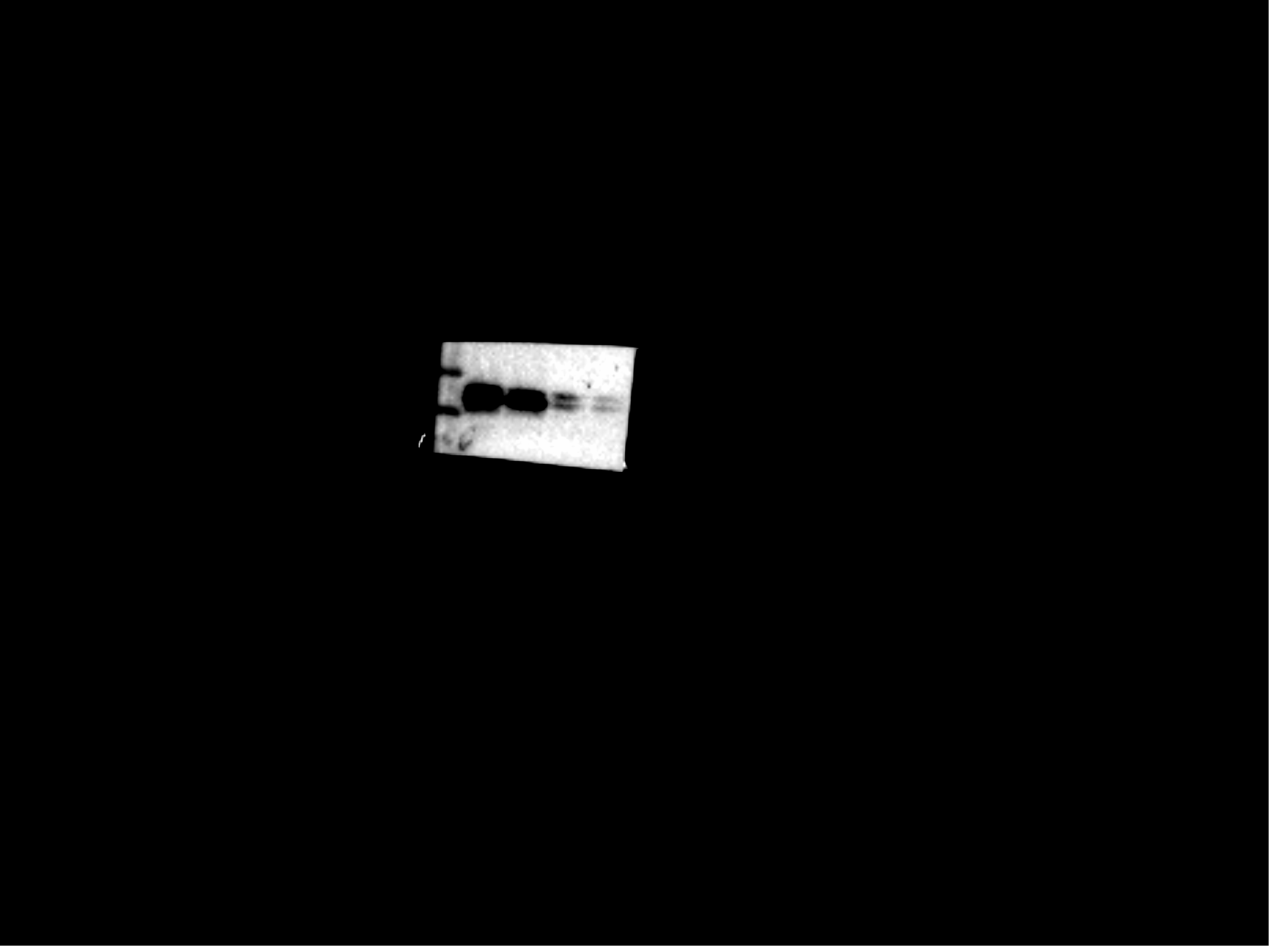


Fig 5I-ERK


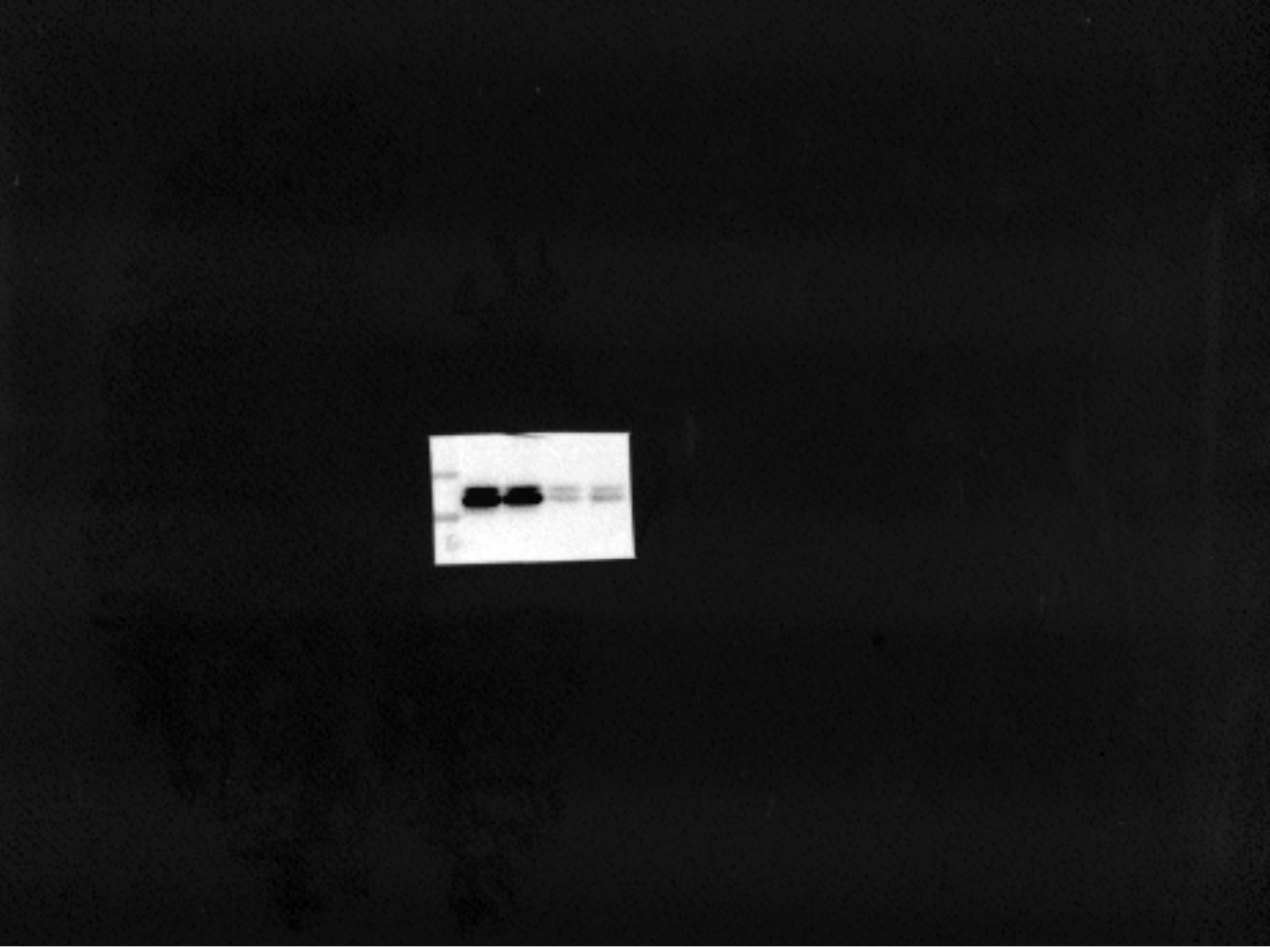


Fig 5I-tublin


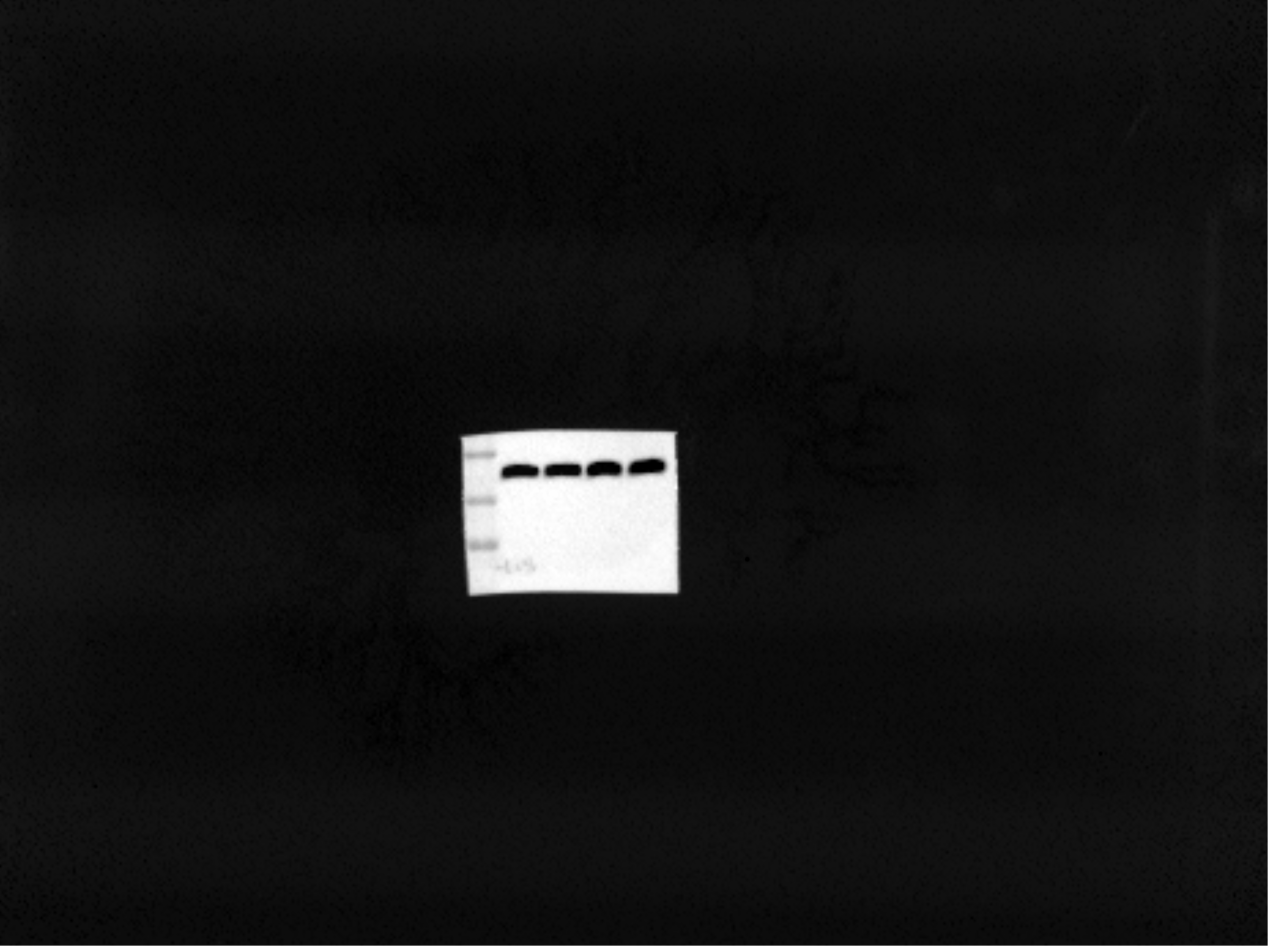


Fig 5J-PRPF3


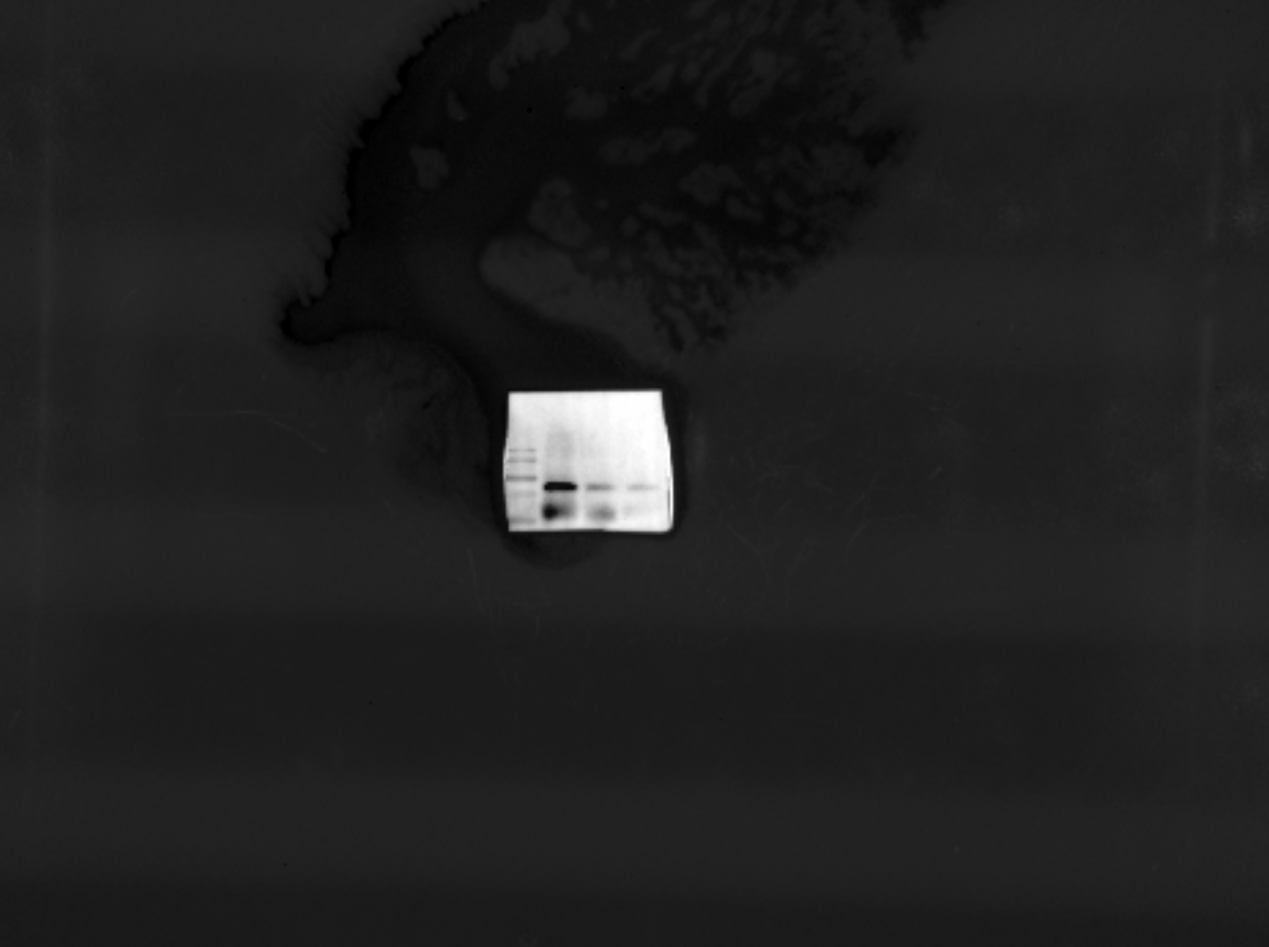


Fig 5J-RAP2B


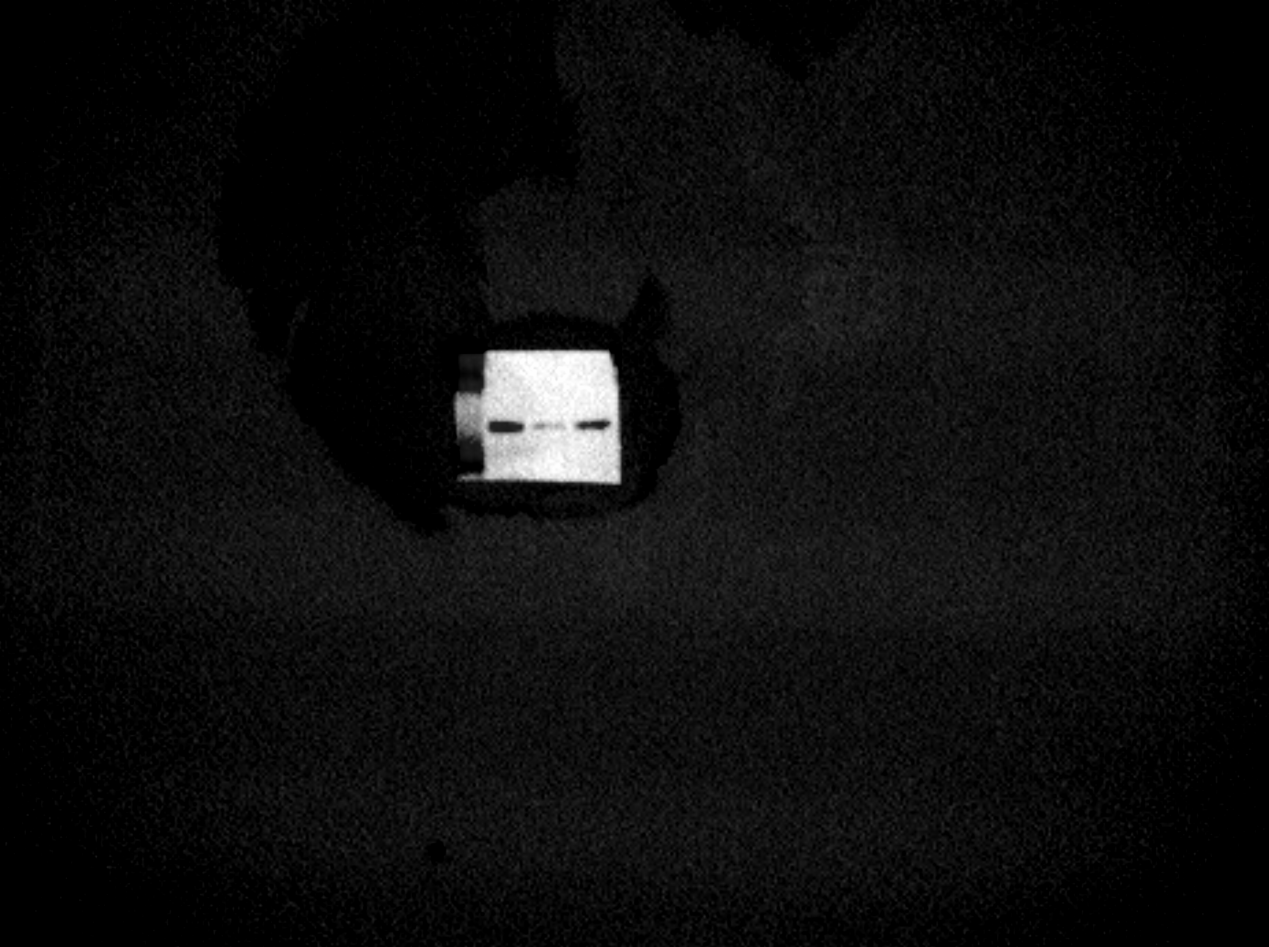


Fig 5J-p-ERK


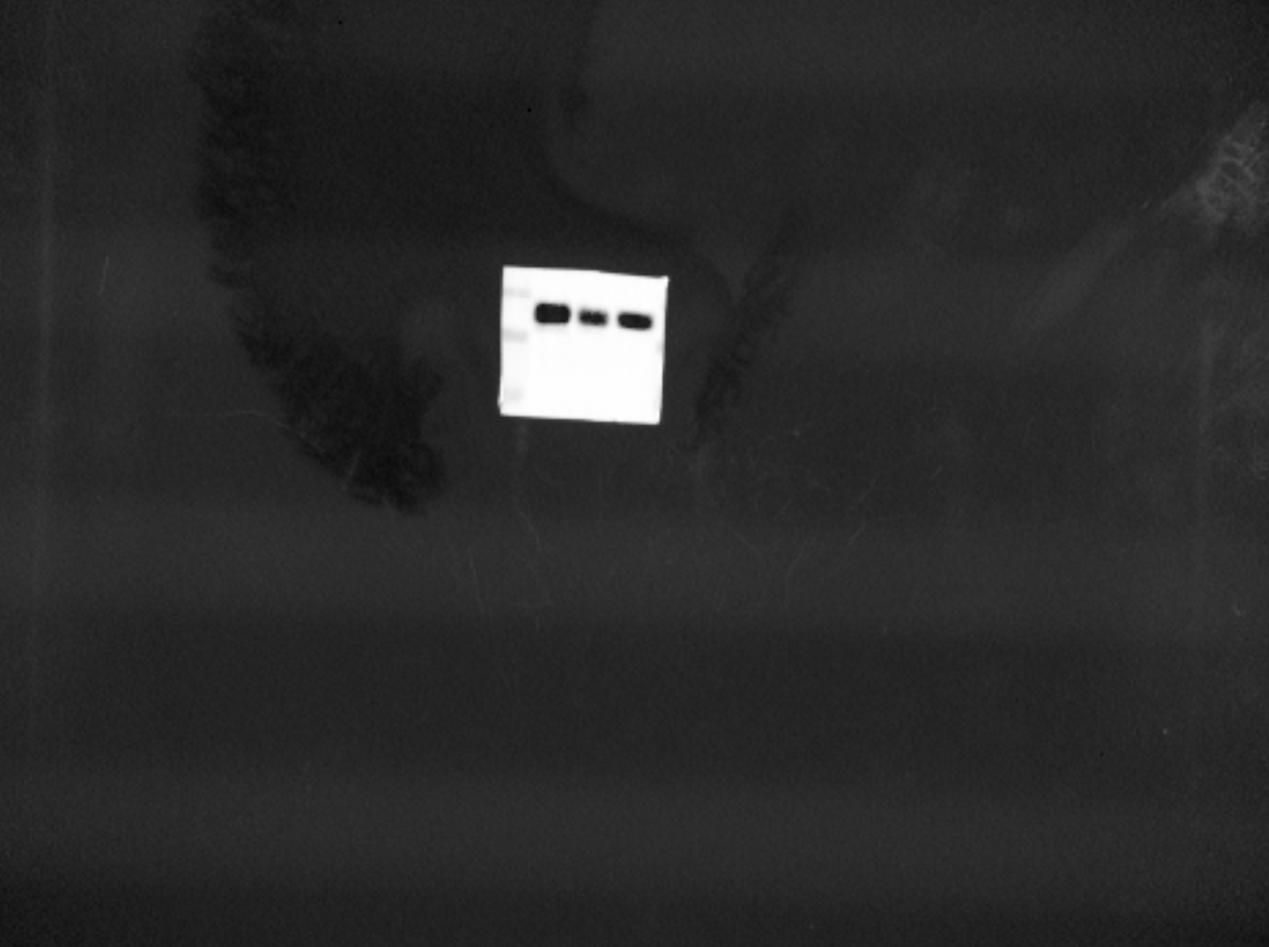


Fig 5J-ERK


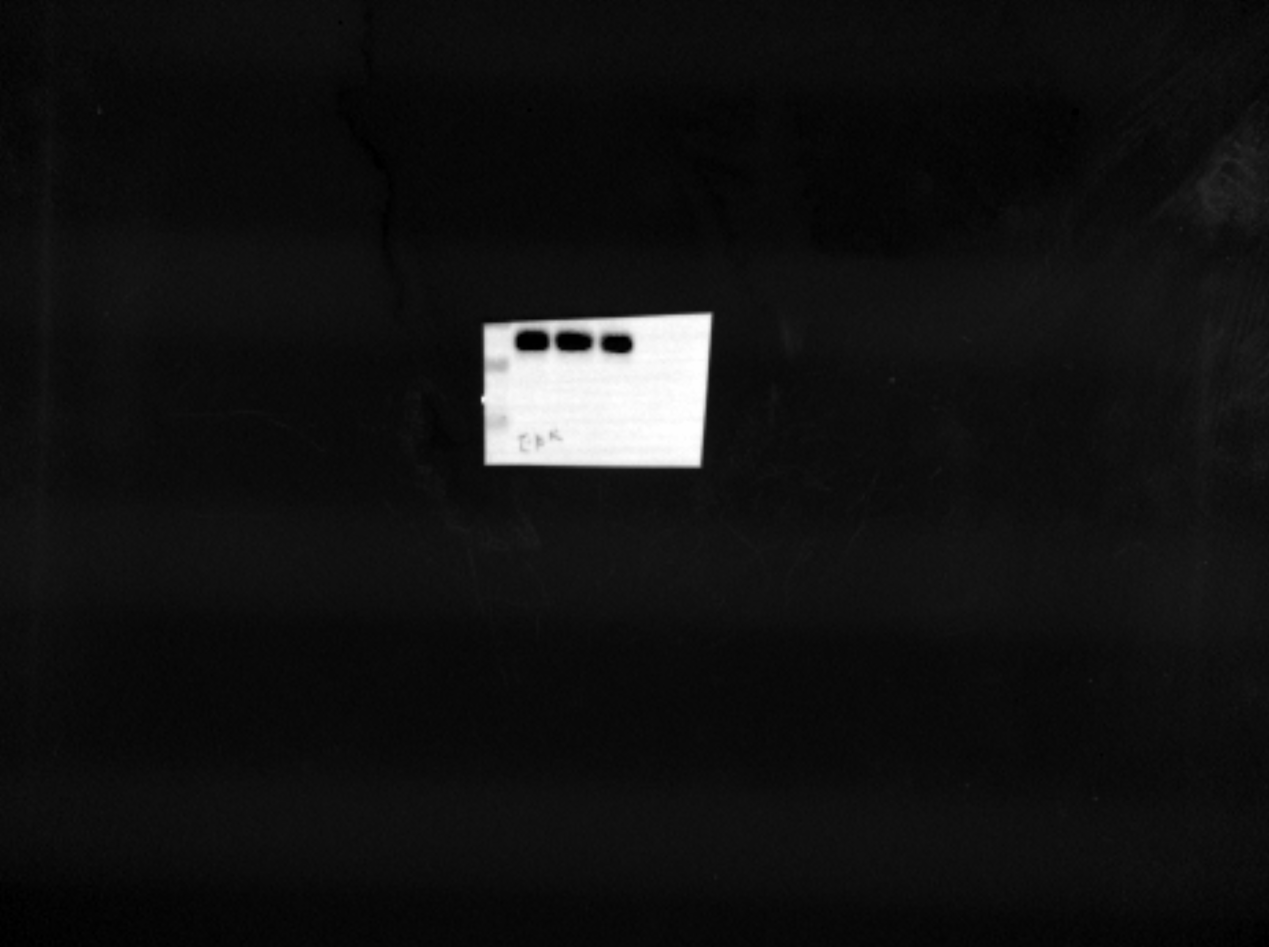


Fig 5J-tublin


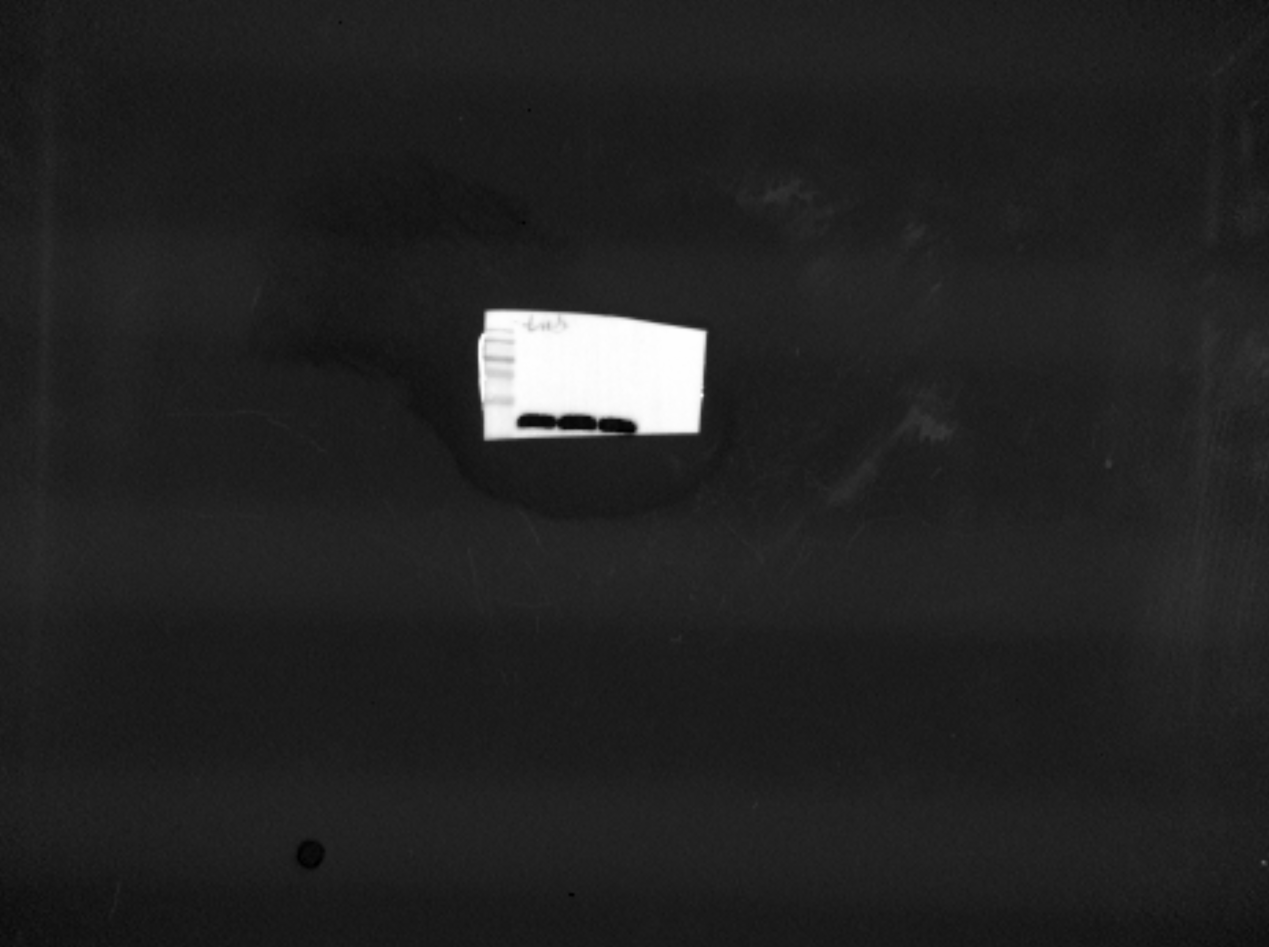


Supp Fig S1-Ub


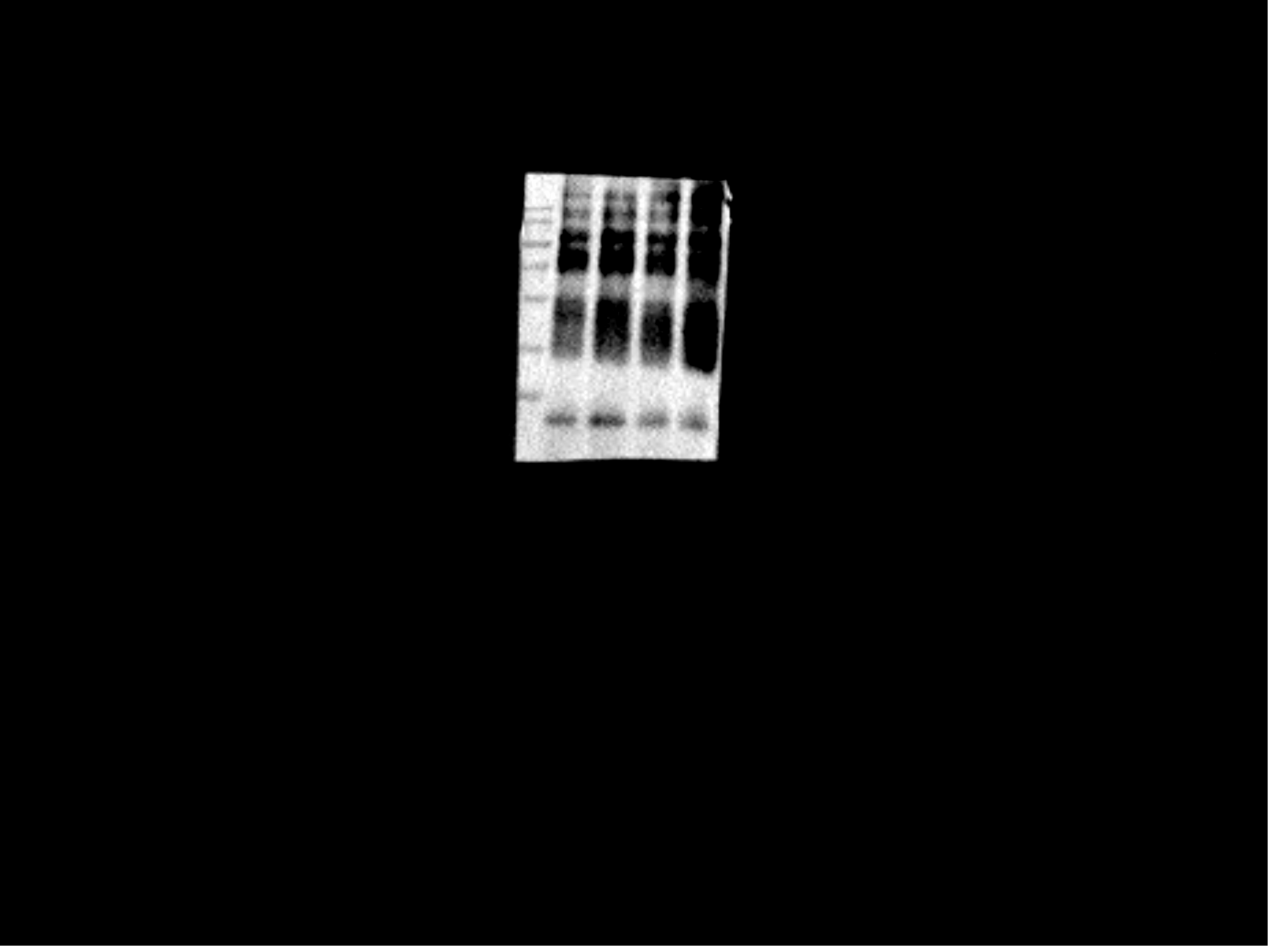


Supp Fig S1-PRPF3(IP)


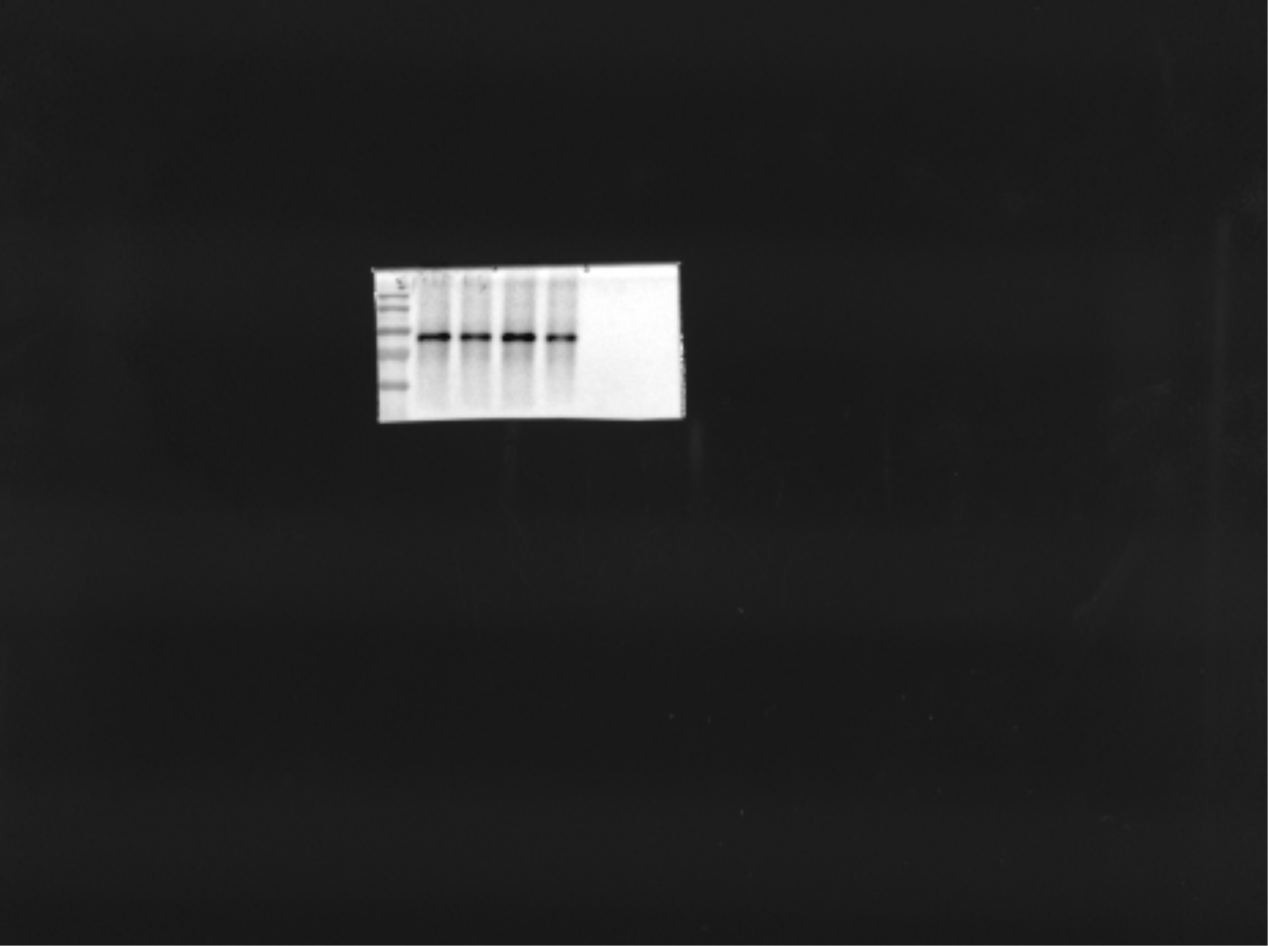


Supp Fig S1-TMEM43


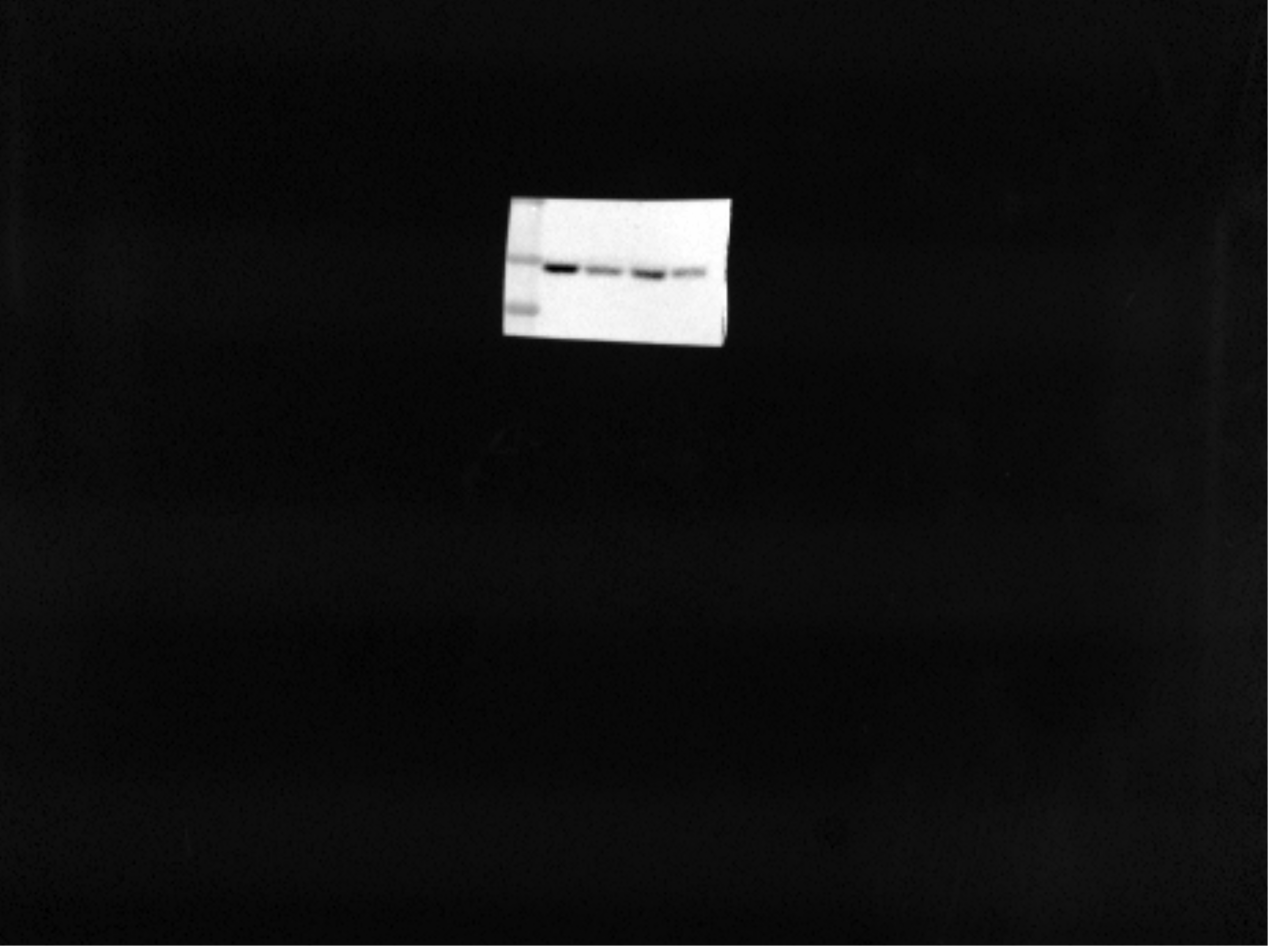


Supp Fig S1-PRPF3


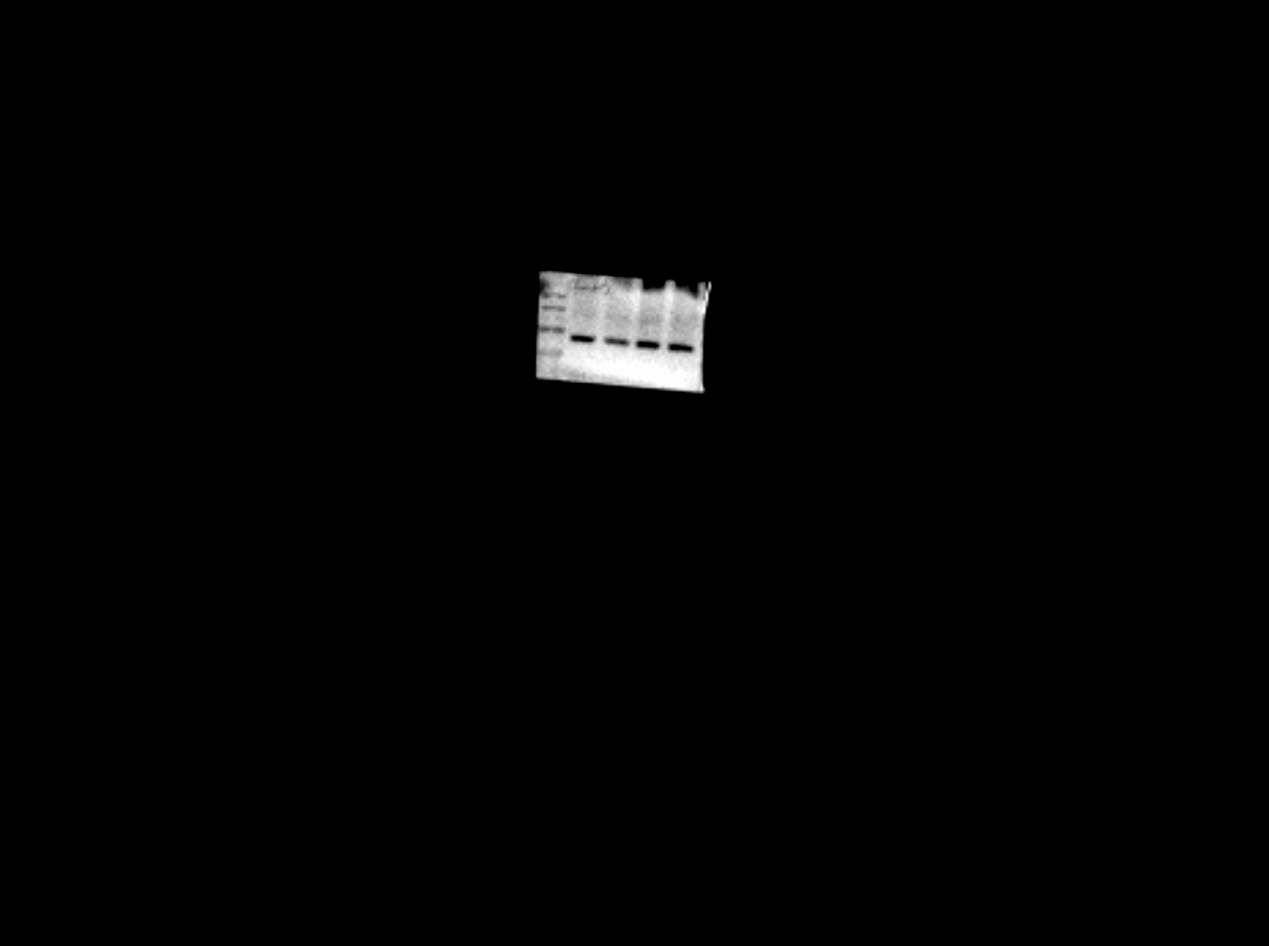


Supp Fig S1-tublin


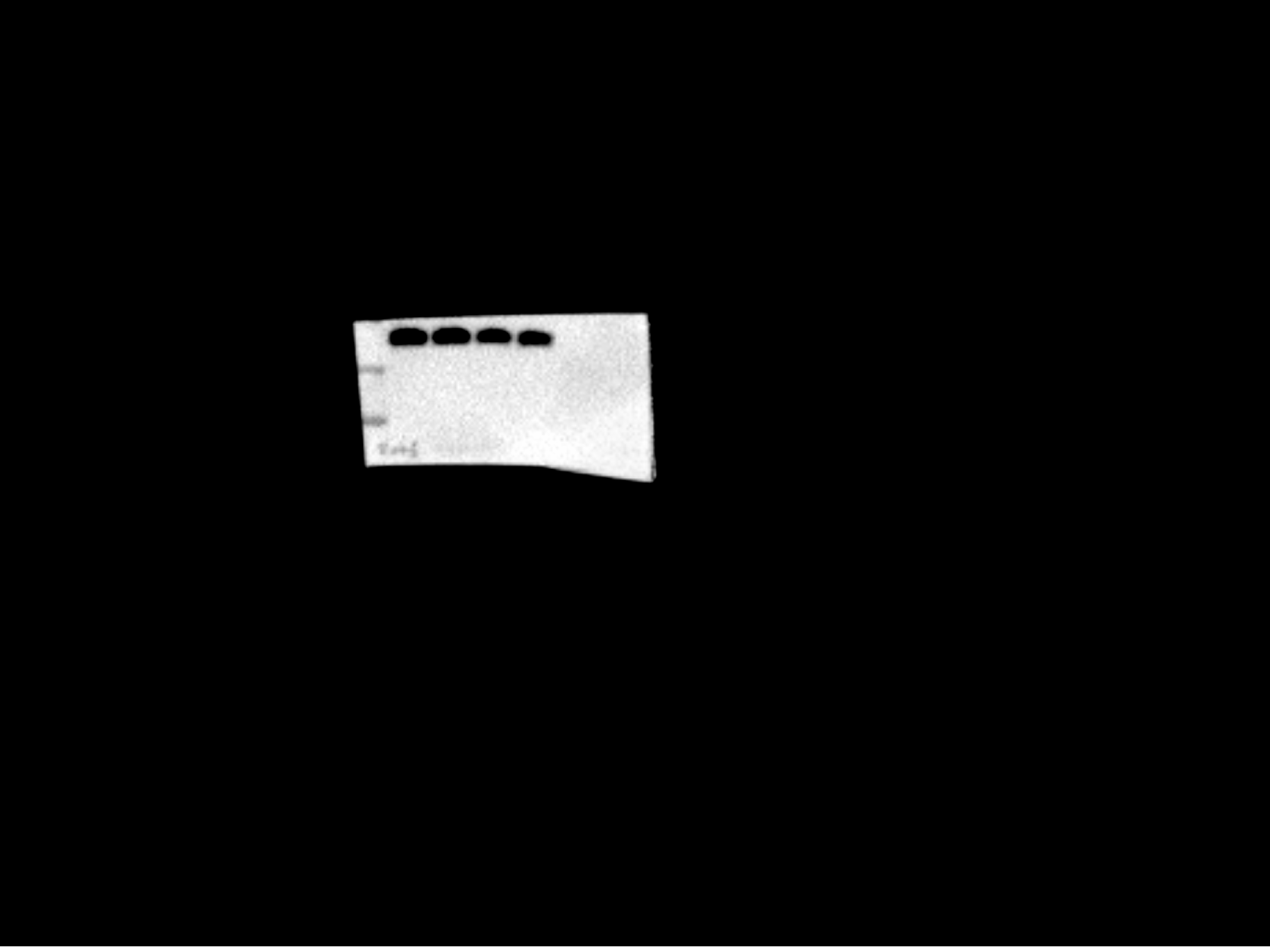

Supplement: Supplementary file 2 — Additional file 2. Original data. [file 11658_2022_321_MOESM2_ESM.docx]
